# Genome-wide meta-analyses reveal novel loci for verbal short-term memory and learning

**DOI:** 10.1038/s41380-022-01710-8

**Published:** 2022-08-16

**Authors:** Jari Lahti, Samuli Tuominen, Qiong Yang, Giulio Pergola, Shahzad Ahmad, Najaf Amin, Nicola J. Armstrong, Alexa Beiser, Katharina Bey, Joshua C. Bis, Eric Boerwinkle, Jan Bressler, Archie Campbell, Harry Campbell, Qiang Chen, Janie Corley, Simon R. Cox, Gail Davies, Philip L. De Jager, Eske M. Derks, Jessica D. Faul, Annette L. Fitzpatrick, Alison E. Fohner, Ian Ford, Myriam Fornage, Zachary Gerring, Hans J. Grabe, Francine Grodstein, Vilmundur Gudnason, Eleanor Simonsick, Elizabeth G. Holliday, Peter K. Joshi, Eero Kajantie, Jaakko Kaprio, Pauliina Karell, Luca Kleineidam, Maria J. Knol, Nicole A. Kochan, John B. Kwok, Markus Leber, Max Lam, Teresa Lee, Shuo Li, Anu Loukola, Tobias Luck, Riccardo E. Marioni, Karen A. Mather, Sarah Medland, Saira S. Mirza, Mike A. Nalls, Kwangsik Nho, Adrienne O’Donnell, Christopher Oldmeadow, Jodie Painter, Alison Pattie, Simone Reppermund, Shannon L. Risacher, Richard J. Rose, Vijay Sadashivaiah, Markus Scholz, Claudia L. Satizabal, Peter W. Schofield, Katharina E. Schraut, Rodney J. Scott, Jeannette Simino, Albert V. Smith, Jennifer A. Smith, David J. Stott, Ida Surakka, Alexander Teumer, Anbupalam Thalamuthu, Stella Trompet, Stephen T. Turner, Sven J. van der Lee, Arno Villringer, Uwe Völker, Robert S. Wilson, Katharina Wittfeld, Eero Vuoksimaa, Rui Xia, Kristine Yaffe, Lei Yu, Habil Zare, Wei Zhao, David Ames, John Attia, David A. Bennett, Henry Brodaty, Daniel I. Chasman, Aaron L. Goldman, Caroline Hayward, M. Arfan Ikram, J. Wouter Jukema, Sharon L. R. Kardia, Todd Lencz, Markus Loeffler, Venkata S. Mattay, Aarno Palotie, Bruce M. Psaty, Alfredo Ramirez, Paul M. Ridker, Steffi G. Riedel-Heller, Perminder S. Sachdev, Andrew J. Saykin, Martin Scherer, Peter R. Schofield, Stephen Sidney, John M. Starr, Julian Trollor, William Ulrich, Michael Wagner, David R. Weir, James F. Wilson, Margaret J. Wright, Daniel R. Weinberger, Stephanie Debette, Johan G. Eriksson, Thomas H. Mosley, Lenore J. Launer, Cornelia M. van Duijn, Ian J. Deary, Sudha Seshadri, Katri Räikkönen

**Affiliations:** 1 Department of Psychology and Logopedics, University of Helsinki, Helsinki, Finland; 2 Turku Institute of Advanced Studies, University of Turku, Turku, Finland; 3 Department of Biostatistics, Boston University, Boston, MA USA; 4 Lieber Institute for Brain Development, Johns Hopkins Medical Campus, Baltimore, MD USA; 5 Department of Basic Medical Science, Neuroscience, and Sense Organs, University of Bari Aldo Moro, Bari, Italy; 6 Department of Epidemiology, Erasmus MC University Medical Center, Rotterdam, The Netherlands; 7 Department of Mathematics and Statistics, Murdoch University, Murdoch, WA Australia; 8 Framingham Heart Study, Framingham, MA USA; 9 Department of Psychiatry and Psychotherapy, University of Bonn, Bonn, Germany; 10 German Center for Neurodegenerative Diseases, Bonn, Germany; 11 Cardiovascular Health Research Unit, Department of Medicine, University of Washington, Seattle, WA USA; 12 Human Genetics Center, School of Public Health, University of Texas Health Science Center at Houston, Houston, TX USA; 13 Human Genome Sequencing Center, Baylor College of Medicine, Houston, TX USA; 14 Centre for Genomic and Experimental Medicine, Institute of Genetics and Molecular Medicine, University of Edinburgh, Edinburgh, UK; 15 Usher Institute, University of Edinburgh, Edinburgh, UK; 16 Centre for Global Health Research, Usher Institute, University of Edinburgh, Edinburgh, UK; 17 Department of Psychology, Centre for Cognitive Ageing and Cognitive Epidemiology, University of Edinburgh, Edinburgh, UK; 18 Center for Translational and Computational Neuroimmunology, Columbia University Medical Center, New York, NY USA; 19 Translational Neurogenomics Laboratory, QIMR Berghofer Medical Research Institute, Brisbane, QLD Australia; 20 Survey Research Center, Institute for Social Research, University of Michigan, Ann Arbor, MI USA; 21 Department of Family Medicine, University of Washington, Seattle, WA USA; 22 Department of Epidemiology, University of Washington, Seattle, WA USA; 23 Department of Global Health, University of Washington, Seattle, WA USA; 24 Institute of Public Health Genetics, University of Washington, Seattle, WA USA; 25 Robertson Center for Biostatistics, University of Glasgow, Glasgow, UK; 26 McGovern Medical School, Brown Foundation Institute of Molecular Medicine, University of Texas Health Science Center at Houston, Houston, TX USA; 27 Department of Psychiatry and Psychotherapy, University Medicine Greifswald, Greifswald, Germany; 28 German Center for Neurodegenerative Diseases, Greifswald, Germany; 29 Channing Laboratory, Brigham and Women’s Hospital, Boston, MA USA; 30 Harvard School of Public Health, Boston, MA USA; 31 Icelandic Heart Assocation, Kopavogur, Iceland; 32 Faculty of Medicine, University of Iceland, Reykjavik, Iceland; 33 Translational Gerontology Branch, National Institute on Aging, Intramural Research Program, National Institutes of Health, Baltimore, MD USA; 34 School of Medicine and Public Health, University of Newcastle, Callaghan, NSW Australia; 35 Institute of Social and Preventive Medicine, University of Lausanne, Lausanne, Switzerland; 36 National Institute for Health and Welfare, Helsinki and Oulu, Oulu, Finland; 37 Hospital for Children and Adolescents, Helsinki University Hospital and University of Helsinki, Helsinki, Finland; 38 PEDEGO Research Unit, MRC Oulu, Oulu University Hospital and University of Oulu, Oulu, Finland; 39 Institute for Molecular Medicine Finland (FIMM), University of Helsinki, Helsinki, Finland; 40 Department of Public Health, University of Helsinki, Helsinki, Finland; 41 Department for Neurodegenerative Diseases and Geriatric Psychiatry, University of Bonn, Bonn, Germany; 42 Centre for Healthy Brain Ageing (CHeBA), School of Psychiatry, Faculty of Medicine, University of New South Wales, Sydney, NSW Australia; 43 Neuropsychiatric Institute, Prince of Wales Hospital, Sydney, NSW Australia; 44 Brain and Mind Centre, The University of Sydney, Sydney, NSW Australia; 45 School of Medical Sciences, University of New South Wales, Sydney, NSW Australia; 46 Department of Psychiatry, University of Cologne, Cologne, Germany; 47 Psychiatry Research, Zucker Hillside Hospital, Glen Oaks, NY USA; 48 Stanley Center for Psychiatric Research, Broad Institute, Cambridge, MA USA; 49 Helsinki Biobank, University of Helsinki Central Hospital, Helsinki, Finland; 50 Department of Economic and Social Sciences & Institute of Social Medicine, Rehabilitation Sciences and Healthcare Research, University of Applied Sciences Nordhausen, Nordhausen, Germany; 51 University of Leipzig, Leipzig, Germany; 52 LIFE Leipzig Research Center for Civilization Diseases, Leipzig, Germany; 53 Centre for Cognitive Ageing and Cognitive Epidemiology, University of Edinburgh, Edinburgh, UK; 54 Sunnybrook Health Sciences Centre, University of Toronto, Randwick, NSW Australia; 55 QIMR Berghofer Medical Research Institute, Brisbane, QLD Australia; 56 Department of Neurology, Sunnybrook Health Sciences Centre, University of Toronto, Toronto, ON Canada; 57 Laboratory of Neurogenetics, National Institute on Aging, Bethesda, MD USA; 58 Data Tecnica International, Glen Echo, MD USA; 59 Center for Neuroimaging, Department of Radiology and Imaging Sciences, Indiana University School of Medicine, Indianapolis, IN USA; 60 Center for Computational Biology and Bioinformatics, Indiana University School of Medicine, Indianapolis, IN USA; 61 Indiana Alzheimer Disease Center, Indiana University School of Medicine, Indianapolis, IN USA; 62 Clinical Research Design, IT and Statistical Support Unit, Hunter Medical Research Institute, New Lambton, NSW Australia; 63 Department of Developmental Disability Neuropsychiatry, School of Psychiatry, University of New South Wales, Sydney, NSW Australia; 64 Department of Psychological & Brain Sciences, Indiana University Bloomington, Bloomington, IN USA; 65 Institute for Medical Informatics, Statistics and Epidemiology, University of Leipzig, Leipzig, Germany; 66 LIFE Research Center for Civilization Diseases, University of Leipzig, Leipzig, Germany; 67 Department of Neurology, Boston University, Boston, MA USA; 68 Glenn Biggs Institute for Alzheimer’s & Neurodegenerative Diseases, University of Texas Health Sciences Center, San Antonio, TX USA; 69 Neuropsychiatry Service, Hunter New England Local Health District, Charlestown, NSW Australia; 70 Centre for Cardiovascular Sciences, Queen’s Medical Research Institute, Royal Infirmary of Edinburgh, University of Edinburgh, Edinburgh, UK; 71 School of Biomedical Sciences and Pharmacy, University of Newcastle, Callaghan, NSW Australia; 72 Hunter Medical Research Institute, New Lambton, NSW Australia; 73 Department of Data Science, University of Mississippi Medical Center, Jackson, MS USA; 74 Department of Epidemiology, University of Michigan, Ann Arbor, MI USA; 75 Institute of Social Research, Survey Research Center, University of Michigan, Ann Arbor, MI USA; 76 Institute of Cardiovascular and Medical Sciences, College of Medical, Veterinary and Life Sciences, University of Glasgow, Glasgow, UK; 77 Department of Internal Medicine, University of Michigan, Ann Arbor, MI USA; 78 Institute for Community Medicine, University Medicine Greifswald, Greifswald, Germany; 79 Section of Gerontology and Geriatrics, Department of Internal Medicine, Leiden University Medical Center, Leiden, The Netherlands; 80 Division of Nephrology and Hypertension, Mayo Clinic, Rochester, MN USA; 81 Department of Neurology and Alzheimer Center, VU University Medical Center, Amsterdam, The Netherlands; 82 Max Planck Institute for Human Cognitive and Brain Sciences, Leipzig, Germany; 83 Day Clinic for Cognitive Neurology, University Hospital Leipzig, Leipzig, Germany; 84 Interfaculty Institute for Genetics and Functional Genomics, Department Functional Genomics, University Medicine Greifswald, Greifswald, Germany; 85 Rush Alzheimer’s Disease Center, Rush University Medical Center, Chicago, IL USA; 86 Institute of Molecular Medicine, University of Texas Health Science Center at Houston, Houston, TX USA; 87 Department of Psychiatry, University of California, San Francisco, CA USA; 88 Department of Cell Systems & Anatomy, The University of Texas Health Science Center, San Antonio, TX USA; 89 Glenn Biggs Institute for Alzheimer’s & Neurodegenerative Diseases, University of Texas, San Antonio, TX USA; 90 University of Texas Health Sciences Center, Houston, NA US; 91 National Ageing Research Institute, Parkville, Melbourne, VIC Australia; 92 University of Melbourne, Academic Unit for Psychiatry of Old Age, St George’s Hospital, Melbourne, VIC Australia; 93 Dementia Collaborative Research Centre, University of New South Wales, Sydney, NSW Australia; 94 Division of Preventive Medicine, Brigham and Women’s Hospital, Boston, MA USA; 95 Harvard Medical School, Boston, MA USA; 96 MRC Human Genetics Unit, Institute of Genetics and Molecular Medicine, University of Edinburgh, Edinburgh, UK; 97 Department of Cardiology, Leiden University Medical Center, Leiden, The Netherlands; 98 Hofstra Northwell School of Medicine, Hempstead, NY USA; 99 Food and Drug Administration, Washington, DC USA; 100 Analytic and Translational Genetics Unit, Department of Medicine, Department of Neurology and Department of Psychiatry, Massachusetts General Hospital, Boston, MA USA; 101 The Stanley Center for Psychiatric Research and Program in Medical and Population Genetics, The Broad Institute of MIT and Harvard, Cambridge, MA USA; 102 Department of Epidemiology and Department of Health Services, University of Washington, Seattle, WA USA; 103 Kaiser Permanente Washington Heath Research Institute, Seattle, WA USA; 104 Institute of Social Medicine, Occupational Health and Public Health, University of Leipzig, Leipzig, Germany; 105 Institute of Primary Medical Care, University Medical Center Hamburg-Eppendorf, Hamburg, Germany; 106 Neuroscience Research Australia, Randwick, NSW Australia; 107 Kaiser Permanente Northern California, Division of Research, Oakland, CA USA; 108 Alzheimer Scotland Dementia Research Centre, University of Edinburgh, Edinburgh, UK; 109 Department of Developmental Disability Neuropsychiatry, School of Psychiatry, Faculty of Medicine, University of New South Wales, Sydney, NSW Australia; 110 Queensland Brain Institute, The University of Queensland, Brisbane, QLD Australia; 111 Centre for Advanced Imaging, The University of Queensland, Brisbane, QLD Australia; 112 Department of Neuroscience, Johns Hopkins University School of Medicine, Baltimore, MD USA; 113 Department of Psychiatry and Behavioral Sciences, Johns Hopkins University School of Medicine, Baltimore, MD USA; 114 McKusick-Nathans Institute of Genetic Medicine, Johns Hopkins University School of Medicine, Baltimore, MD USA; 115 Inserm, Bordeaux Population Health Research Center, team VINTAGE, UMR 1219, University of Bordeaux, Bordeaux, France; 116 Bordeaux University Hospital (CHU Bordeaux), Department of Neurology, Bordeaux, France; 117 Folkhälsan Research Center, Helsinki, Finland; 118 Department of General Practice and Primary Health Care, University of Helsinki, and Helsinki University Hospital, University of Helsinki, Helsinki, Finland; 119 Department of Obstetrics & Gynaecology, Yong Loo Lin School of Medicine, National University of Singapore and National University Health System, Helsinki, Singapore; 120 Department of Medicine, Division of Geriatrics, University of Mississippi Medical Center, Jackson, MS USA; 121 Laboratory of Epidemiology and Population Sciences, National Institute on Aging, Intramural Research Program, National Institutes of Health, Bethesda, MD USA; 122 Department of Public Health, Oxford University, Oxford, UK

**Keywords:** Genetics, Neuroscience

## Abstract

Understanding the genomic basis of memory processes may help in combating neurodegenerative disorders. Hence, we examined the associations of common genetic variants with verbal short-term memory and verbal learning in adults without dementia or stroke (*N* = 53,637). We identified novel loci in the intronic region of *CDH18*, and at 13q21 and 3p21.1, as well as an expected signal in the *APOE/APOC1/TOMM40* region. These results replicated in an independent sample. Functional and bioinformatic analyses supported many of these loci and further implicated *POC1*. We showed that polygenic score for verbal learning associated with brain activation in right parieto-occipital region during working memory task. Finally, we showed genetic correlations of these memory traits with several neurocognitive and health outcomes. Our findings suggest a role of several genomic loci in verbal memory processes.

## Introduction

The ability to focus attention and to encode, store, and recall information are not only imperative for survival but these memory-related cognitive processes also reflect healthy brain aging [1, 2]. Cognitive decline, especially episodic memory impairment, is a clinical hallmark and genetic endophenotype of several types of dementia, especially Alzheimer’s disease (AD) [3]. Understanding the genetic and molecular basis of inter-individual variation in normal memory function could improve precision in screening for dementias, and identify novel drug targets to support cognitive reserve, and to prevent and treat dementia.

Both episodic memory in cognitively normal individuals [3, 4] and AD [5] show moderate to high heritability in twin studies. Large-scale genome-wide association meta-analyses (GWAMAs) across several cohorts have identified over 30 genomic loci for AD [6], but GWAMAs for episodic memory among dementia-free adults have shown less consistent findings [7–17]. In the largest GWAMA of episodic memory, Davies et al. [17] did not find any significant genomic variants for visuo-spatial memory in the UK Biobank sample of 112,067 persons. As visuo-spatial encoding of information involves partially different brain networks compared to verbal encoding [18], genomic architecture of visuo-spatial memory and verbal memory may differ. Indeed, an earlier GWAMA from the CHARGE consortium showed that rs4420638 at 19q13.3 near the *APOE-APOC1-TOMM40* locus, that shows the largest known effects on AD [6], was associated with verbal long-term memory (delayed recall) in a sample of 29,076 persons [7]. There is ample evidence for differences in brain networks and thus, genetic networks, that are involved in long-term and short-term episodic memory processes [19]. A relatively small (*N* = 7486) genome-wide association study of immediate recall scores in tests of verbal episodic memory (verbal short-term memory; VSTM), however, detected the same *APOE-APOC1-TOMM40* locus [16]. GWAMAs with considerably larger sample sizes are needed to find novel loci beyond this locus.

Therefore, we examined if common genetic variants were associated with verbal episodic memory in adults of European ancestry without dementia or stroke in the Cohorts for Heart and Aging Research in Genomic Epidemiology (CHARGE) consortium. We operationalized VSTM as immediate recall scores in tests of verbal episodic memory and conducted a GWAMA in a sample of 53,637persons (32 cohorts). As verbal learning (VL) tasks may constitute a more sensitive marker of cognitive deficits than tests of VSTM without a learning component [20] and to our knowledge, only one small (*N* = 700) GWAMA for VL exists [15], we also examined genetic underpinnings of VL in 32,762 persons (19 cohorts). To assess the functional role of the identified variants, we analyzed fMRI activations during working memory performance and computed genomic associations.

## Results

The characteristics of the study cohorts, details of memory tests administered, genotyping quality control and genomic inflation factors are shown in Supplementary information S1–S4 and Supplement 1.

**Fig. 1:**
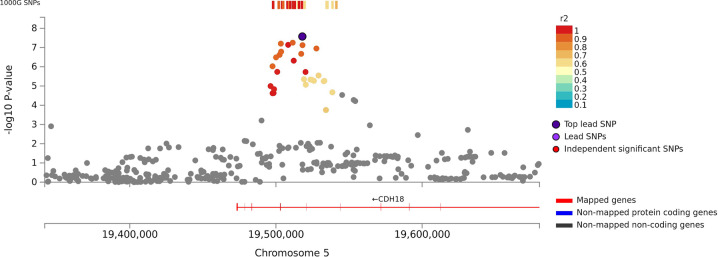
Regional plot of associations of SNPs at the 5p14.3 region with verbal short-term memory in the discovery sample (*N* = 44,874). Dots indicate p-values of SNPs and rs425724 in an intron of *CDH18* is marked in violet.

**Fig. 2:**
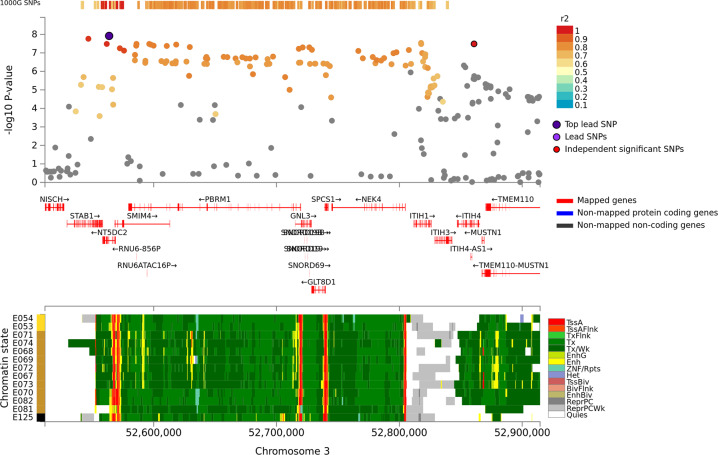
Regional plot of associations of SNPs at the 3q21 region with verbal learning in the discovery sample (*N* = 28,909; Upper panel). Dots indicate *p*-values of SNPs and the top lead SNP rs4687625 is marked in violet and another independent and significant SNP rs2276816 is marked in red. Lower panel indicates 15-core chromatin state in Roadmap brain-related tissues (E053-E082) and E125 ENCODE NH-A Astrocytes primary cells and shows that both significant SNPs are in transcriptionally active region.

**Fig. 3:**
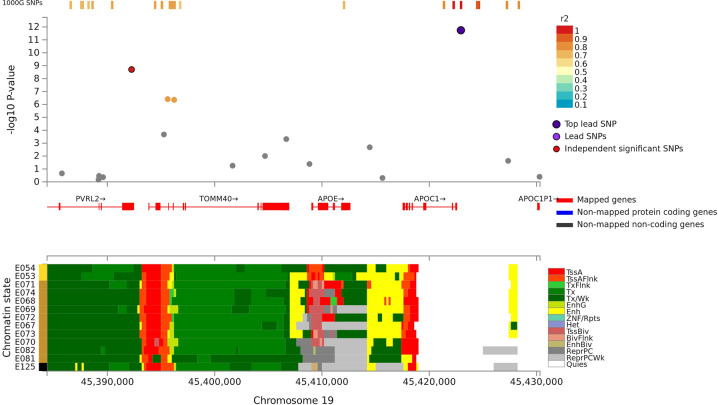
Regional plot of associations of SNPs at the 19q13.3 region with verbal learning (Upper panel). Dots indicate *p* values of SNPs and the top lead SNP rs4420638 is marked in violet and another independent and significant SNP rs6857 is marked in red. Lower panel indicates 15-core chromatin state in Roadmap brain-related tissues (E053-E082) and E125 ENCODE NH-A Astrocytes primary cells and shows that both significant SNPs are in or flanking transcriptionally active region.

Due to differences in verbal memory tests used in the different cohorts, we performed sample-size based meta-analyses using METAL [21]. All models were adjusted for age, sex, and population substructure. Table 1 shows results for the lead SNPs and Figs. 1–3 shows regional plots of genome-wide significant associations. Supplementary Figs. 1–7 show Manhattan plots of all genomic associations and Supplementary Table S1 shows all genome-wide significant (*p* < 5 × 10^−8^) and suggestive (5 × 10^−8^ ≥ *p* < 5 × 10^−6^) associations in the discovery sample.

**Table 1:** Meta-analyses results of lead SNPs for verbal short-term memory, paragraph recall, and verbal learning in discovery, replication, and combined samples (*N* = 53,637).

								Discovery cohorts					Replication cohorts					Discovery + Replication cohorts		
Pheno-type	SNP	Chr	Genomic position	EA/OA	EAF	Nearby gene	Predicted function	*N*	Z-score	*P*-val	Direction	Het *P*-val	*N*	Z-score	*P*-val	Direction	Het *P*-val	*N*^a^	Z-score	*P*-val
VL	rs4687625	3	52538758	t/c	0.38	NT5DC2	Intronic	28579	5.70	1.2E−08	+?+++++++++++−++++++++	0.97	3853	2.74	0.006	++	0.28	32432	6.3	3.1E−10
VL	rs2015971	3	52521860	t/c	0.38	STAB1	Intronic	28127	5.64	1.7E−08	+?−+++?++++++−++++++++	0.96	3853	2.70	0.007	++	0.23	31980	6.2	4.8E−10
VL	rs11711421	3	52536819	t/c	0.39	NT5DC2	Intronic	28579	5.53	3.3E−08	+?−++++++++++−++++++++	0.96	3853	2.55	0.01	++	0.29	32432	6.1	1.3E−9
VL	rs2276816	3	52835856	t/c	0.12	ITIH4	Synon	27896	5.53	3.3E−08	+++++++++−+−+?++++++++	0.53	3067	0.61	0.54	?+	1	30963	5.4	5.4E−8
VSTM	rs425724	5	19518050	c/t	0.12	CDH18	Intronic	44668	5.56	2.7E−08	?++++++++−−+++++++++++++++−	0.67	8763	2.04	0.04	+++++	0.84	53431	5.91	3.4E−09
VSTM: Paragraph	rs9528369	13	62216927	a/c	0.32		Intergenic	18098	−6.0	2.0E−09	?−−−−−−??−−−+	0.62	4293	−0.79	0.43	−+−	0.66	22391	−5.74	9.4E−9
VSTM	rs4420638	19	45422946	g/a	0.18	APOC1	3’down-stream	37556	−7.23	4.9E−13	?+−−−−−?−−−−−−??−?−−−−+−?−−	5.5E−05	8164	−2.89	0.004	−−?−+	1.4E−04	44317	−7.83	4.9E−15
VL	−“−	−“−	−“−	−“−	−“−	−“−	−“−	23575	−7.05	1.8E−12	−?+−−−−?−−−?−?−−−−−−+?	0.14	3067	0.56	0.58	?+	1	26642	−6.4	1.2E−10
VSTM: Paragraph	−“−	−“−	−“−	−“−	−“−	−“−	−“−	16216	−6.93	4.2E−12	?−−−−?−?−−−−−	4.4E−05	3694	−5.14	2.78E−07	−?−	0.37	19910	−8.47	2.5E−17
VL: Visual	−“−	−“−	−“−	−“−	−“−	−“−	−“−	15096	−6.0	3.1E−09	−−?−−−−−−−+									
VL	rs6857	19	45392254	t/c	0.16	PVRL2	3’utr	26849	−6.00	2.0E−09	−?−−−−−?−−−+−?−−−−−−++	2.2E−05	3067	0.63	0.53	?+	1	29916	−5.5	4.2E−8

All models were adjusted for sex, age, and population substructure and results reflect analyses in participants of European ancestry without dementia or stroke.

*VSTM* Verbal short-term memory, *VL* Verbal learning, *VSTM Paragraph* VSTM meta-analyses restricted to those cohorts with paragraph recall test, *VL Visual* meta-analyses restricted to those cohorts with VL test using visual presentation of the words, *EA* Effect allele, *OA* Other allele, *EAF* Effect allele frequency.

^a^*N* varies due to missing data.

For VSTM, we observed two significant associations in the discovery sample (*N* = 44,874): rs425724 (*p* = 2.7 × 10^−8^) within an intron of *CDH18* and rs4420638 (*p* = 4.9 × 10^−13^) downstream of *APOC1* at 19q13.3. Associations of both SNPs with VSTM were replicated in an independent sample at nominal significance (*p* values < 0.04; *N* = 8763).

For VL, we observed significant associations at the same 19q13.3 locus and at 3p21 in the discovery sample (*N* = 28,909). At the 19q13.3 locus the strongest associations were observed with rs4420638 (*p* = 1.8 × 10^−12^) and rs6857 (*p* = 2.0 × 10^−9^) that are in linkage disequilibrium (LD; *r*^2^: 0.45) with each other. The 3p21 locus harbors a large LD block in/near *NT5DC2, STAB1, ITIH1, ITIH4*, and *PBRM1*. Out of 14 SNPs showing a significant association at this locus, rs4687625, within an intron of *NT5DC2*, and a synonymous *ITIH4* variant rs2276816 were independently significant SNPs (*r*^2^: 0.12, distance: 297 kb). Three of the significant 3p21 SNPs (rs4687625, rs2015971, and rs11711421; all intronic to or near *NT5DC2*) showed nominally significant association with VL scores in an independent replication sample (*p* values < 0.01; *N* = 3853).

Despite some heterogeneity between the cohorts in 19q13.3 SNPs (rs4420638 and rs6857), no single cohort drove the results (Supplementary Figs. 7–13). We further examined with meta-regression if cohort-level characteristics influenced estimates of the association between these SNPs and memory test scores. Larger effect estimates in both 19q13.3 SNPs associated with smaller proportion of women in the cohort and rs4420638 effect estimates for VL associated with younger mean age of the cohort (Supplementary Table S16).

There were no other significant signals in the analyses combining discovery and replication cohorts (Supplementary Table S8).

### Analyses stratified by the type of the memory test

As in Debette et al. [7], we further meta-analyzed cohorts based on the specific type of memory test applied. In the analyses of VSTM, cohorts were classified into those with paragraph recall test data (13 cohorts, *N* = 19,420) and those with word list recall test data (14 cohorts, *N* = 25,454). In the analyses of VL, cohorts were classified into those with orally presented words (11 cohorts, *N* = 12,593) and those with visually presented words (11 cohorts, *N* = 16,191).

In the analyses restricted to cohorts with the VSTM paragraph recall tests, we observed a novel locus in an intergenic region at 13q21 (lead SNP rs9528369, *p* = 2.0 × 10^−9^) and a second locus at 19q13.3 (lead SNP rs4420638, *p* = 4.2 × 10^−12^). Additionally, rs4420638 showed a significant association with VL in those cohorts with visually presented words (*p* = 3.1 × 10^−9^). Of these results, we were able to replicate the association of rs4420638 with paragraph recall (*p* = 1.4 × 10^−4^) in an independent replication sample (*N* = 4293). There were no significant associations in the other stratified meta-analyses.

### Analyses adjusting for educational attainment

Following Debette et al. [7], we ran secondary analyses to test if associations were independent of education. All associations in the significant lead SNPs remained significant after further adjusting the models for educational attainment except that the associations of rs4687625 (*p* = 8.8 × 10^−7^) and rs2276816 (*p* = 5.3 × 10^−6^) at 3p21 with VL became only suggestively significant.

### Gene-based, gene-set, and gene property analysis results with MAGMA

Gene-based association analyses with MAGMA identified one gene for VSTM (*APOC1* at 19q13.3), 15 genes for VL (*SMIM4, STAB1, PBRM1, NEK4, NT5DC2, ITIH4, GNL3, ITIH1, MUSTN1, GLT8D1*, and *ITIH3* at 3p21; *CALN1* at 7q11*; TOMM40* and *APOC1* at 19q13.3; and *AGXT2* at 5p13), and two genes for paragraph recall (*APOC1* and *TOMM40* at 19q13.3) after Bonferroni correction for multiple testing (Supplementary Table S2 and Supplementary Fig. 21). We found no significant enrichment in gene-set analyses (Supplementary Table S3).

Gene-property analysis tests if tissue-specific expression is predictive of the association of the gene with the phenotype. These analyses indicate that genes with the highest expression levels in the pituitary and all available brain regions, except for the rostral intracranial portion of the spinal cord, were the same genes showing significant associations with VSTM and with paragraph recall, but not with VL (Supplementary Table S4).

### Functional analyses and colocalization

We identified potential functionality of SNPs showing significant associations with FUMA [22] (Supplementary Tables S5 and S6). Fourteen SNPs at the 3p21 locus that associated with VL are significant eQTLs for *POC1A, GNL3, GLYCTK, DUSP7, ITIH4, PPM1M*, and *GLT8D1* in putamen, cerebellum, frontal cortex, and/or hippocampus in the Genotype-Tissue Expression (GTeX) and in putamen, white matter, and/or hippocampus in the Brain eQTL Almanac (Braineac) database. Of these, rs2276816 is also a synonymous exonic SNP with a Combined Annotation Dependent Depletion (CADD) score indicating a potential functionally deleterious effect (CADD > 12.37) [23]. Additionally, rs1961958, that associated with VL, and rs11148561, that associated with paragraph recall, have high CADD scores. Moreover, 3p21 locus SNPs rs4687625, rs1961959, rs6798246, and rs3774355, that associated with VL, also may influence gene regulation as indicated by both eQTL data and transcription factor binding data (regulomeDB category 1f [24]). Roadmap 15-core chromatin states show that 3p21 and 19q13.3 loci are situated in transcriptionally active regions and rs6798246 flanks an active transcription start site in brain tissues (Figs. 2 and 3). Additionally, our methylation QTL (mQTL) and amyloid/tau accumulation PET analyses corroborate the functional role of the 3p21, 13q21 and 19q13.3 loci in the brain tissues. In the dorsolateral prefrontal cortex (DLPFC) samples of the Religious Orders Study and Rush Memory and Aging Project (ROSMAP) (*N* = 322), the top 3p21 SNPs associated with methylation levels of CpGs corresponding to *ITIH4, ITIH1, STAB1, NEK4, MUSTN1, DNAH1, TLR9, GNL3, SNORD69, TMEM110*, and *NT5DC2* (p(Benjamini-Hochberg false discovery rate [FDR]) < 0.01). Moreover, rs9528369 associated with a cg09367879 located in the open sea region in chromosome 13, and rs6857 associated with a CpG in the *APOE* (Supplementary Table S13). Both 19q13.3 SNPs marginally associated with tau accumulation in the precuneus, and rs4420638 also associated with overall amyloid accumulation in a Framingham Heart Study (FHS) sample of young adults with PET imaging (*N* = 183) (Supplementary Table S15). Chromatin-chromatin interaction analyses show that all genomic regions implicated in VSTM, VL, and paragraph recall showed significant interactions with other intra-chromosomal regions (Supplementary Figs. 14–19 and Supplementary Table S6). For example, the intronic *CDH18* region implicated in VSTM analyses interacts with the *CDH18* promoter region in the Roadmap Epigenomics Project brain tissue samples. In these same brain samples, the intergenic 13q21 region implicated in the paragraph recall analyses interacts with the promoter region of *TDRD3*. This same region also interacted with the *PCDH20* gene region in non-brain tissue samples.

Using S-PrediXcan [25], after Bonferroni correction for multiple testing we identified a single gene (*POC1A*) whose expression in the putamen was negatively associated with VL (Z = −5.02; *p* = 5.04 × 10^−7^) whereas no significant associations were observed for VSTM (Supplementary Table S7 and Supplementary Fig. 20).

**Fig. 4:**
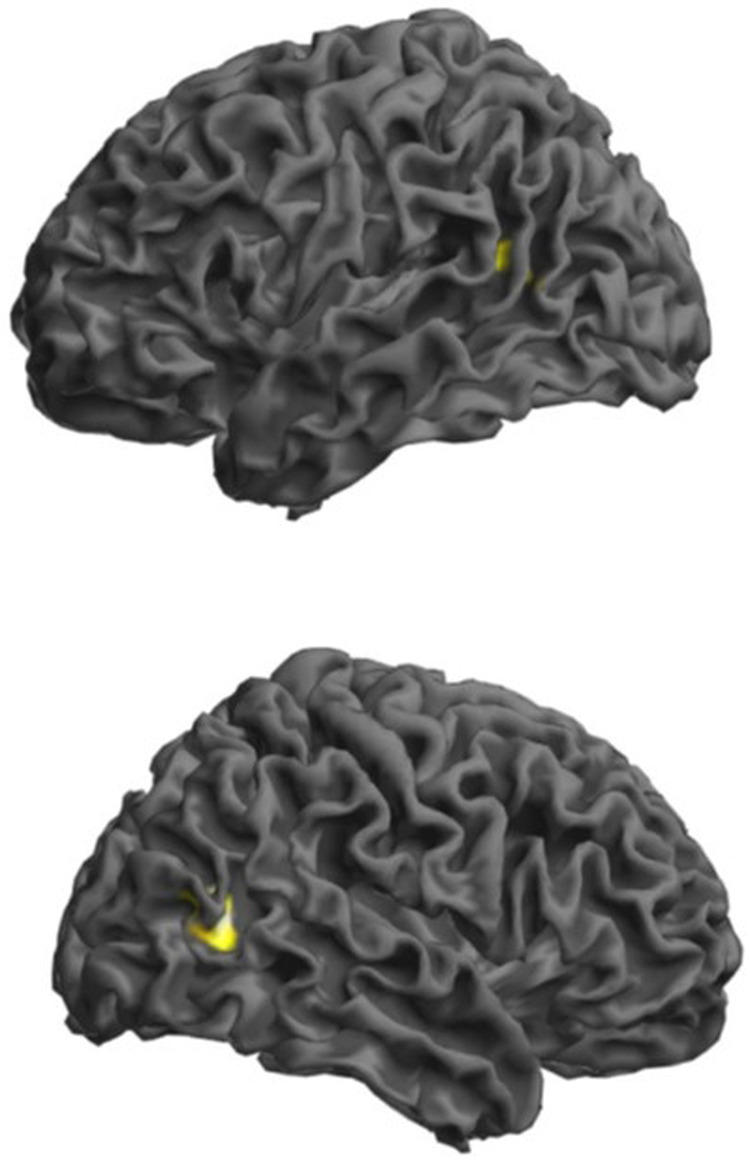
Associations of the polygenic score for verbal learning (PGS_VL_) with activation in the right BA19 during the 2-back working memory task in a sample of *N* = 435 healthy adults (upper panel left view and lower panel right view). Results are thresholded at peak-level *p* < 0.001 and masked for significantly increased activity during 2-back relative to 0-back. Rendered image illustrates clusters in which activity is negatively correlated with the PGS_VL_ (the right cluster survives correction for multiple comparisons at BA19; MNI coordinates *x* = 45, *y* = −64, *Z* = 10; FWE corrected *p* = 0.016). Left in the figure is left in the brain.

Finally, we tested with polygenic scores (PGSs) the overall association of VSTM (PGS_VSTM_) and VL (PGS_VL_) with brain activation assessed via fMRI during a working memory task in 435 healthy participants in the Clinical Brain Disorders Branch Sibling Study. The intermediate PGS_VL_ (SNP inclusion *p* value < 10^−4^) correlated negatively with activity in a right parieto-occipital cluster with a peak in BA19 (peak Z = 4.73; p_FWE_ = 0.016; 55 voxels; MNI coordinates *x* = 45; *y* = −64; *z* = 10; Fig. 4). At a lower *p* < 0.001 (uncorrected) threshold, a symmetric cluster was significant on the left with a peak in BA39 (peak Z = 3.55; 24 voxels; MNI coordinates *x* = −45; *y* = −58; *z* = 13; Fig. 4). No results survived correction for multiple comparisons using the PGS_VSTM._

### Protein-protein interactions

We investigated protein-protein interactions with DAPPLE [26] and results are presented in Supplementary Table 12. Fourteen, 30, and 11 proteins were included in the network construction for VSTM, VL, and paragraph recall, respectively, but six, 16, and two proteins were present in direct or indirect networks, respectively. None of the network parameters were significant. In the analyses of single proteins, SYT9 and NRXN1 were significant for VSTM (*p* = 0.006), ZFAND5, GRIK2, and ZC3H18 were nominally significant for VL (*p* = 0.018–0.05), and PRLHR was nominally significant for paragraph recall (*p* = 0.044).

### Genetic correlation analyses

**Fig. 5:**
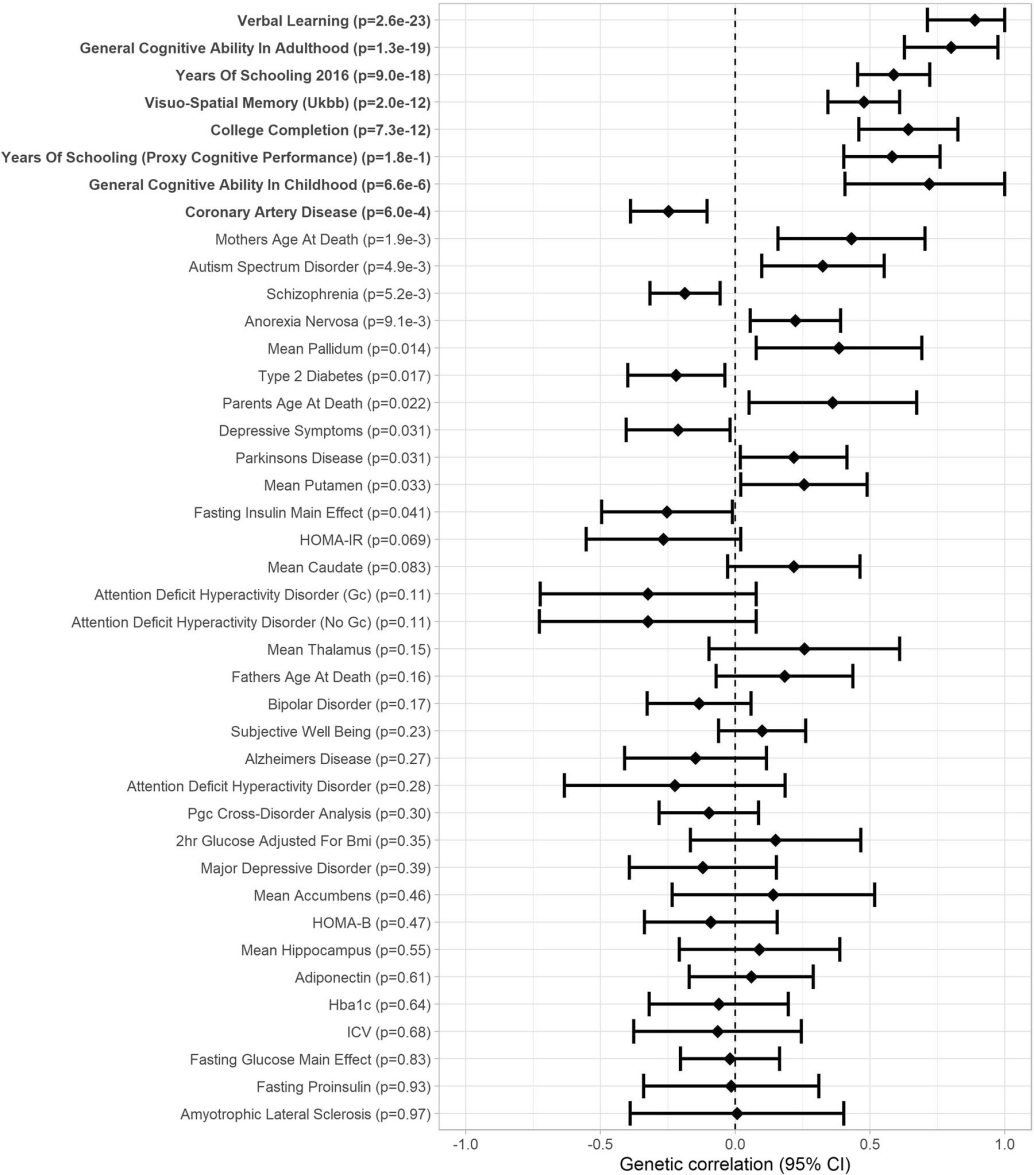
Forest plot of genetic correlations between verbal short-term memory and 46 traits related to cognitive abilities or health (Genetic correlation [95% confidence interval]; significant genetic correlations after FDR correction in boldface).

**Fig. 6:**
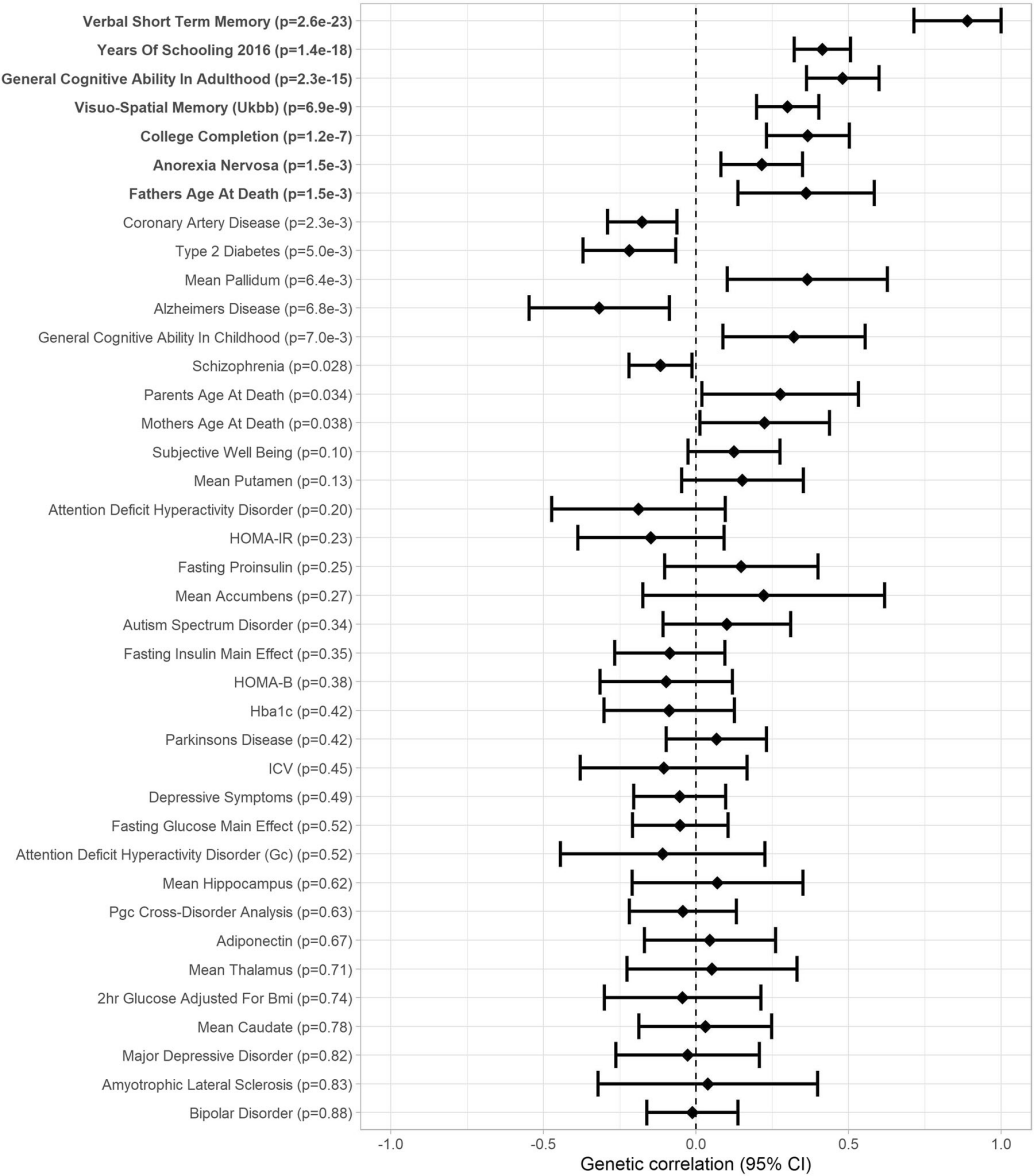
Forest plot of genetic correlations between verbal learning and 46 traits related to cognitive abilities or health (Genetic correlation [95% confidence interval]; significant genetic correlations after FDR correction in boldface).

We used LDHub [27] for analyses of SNP-based heritability and genetic correlations. In the cohorts that could pool individual participant data (16 cohorts *N* = 26,977 in VSTM and 15 cohorts *N* = 25,180 in VL), SNP-based heritability was 0.06 (SE: 0.02) and 0.18 (SE: 0.02) for VSTM and VL, respectively. Genetic correlations between VSTM, VL, and health-related phenotypes are presented in Figs. 5, 6 and in Supplementary Table S11. After FDR correction, VSTM and VL showed positive genetic correlation with each other (*r*_g_ = 0.89, *p* = 2.6 × 10^−23^) and with general cognitive ability (GCA; *r*_g_ > 0.44, *p* < 2.3 × 10^−16^) in adults (and VSTM also with GCA in childhood, *r*_g_ > 0.72, *p* < 7.3 × 10^−6^), visuo-spatial memory in the UK Biobank (*r*_g_ > 0.30, *p* < 6.9 × 10^−9^), years of schooling (*r*_g_ > 0.41, *p* < 1.4 × 10^−18^), and college completion (*r*_g_ > 0.37, *p* < 1.2 × 10^−7^). In addition, VSTM showed negative genetic correlation with coronary artery disease (*r*_g_ = −0.25, *p* = 6.0 × 10^−4^), and VL showed positive genetic correlation with anorexia nervosa (*r*_g_ = 0.37, *p* = 1.2 × 10^−7^) and father’s age at death (*r*_g_ = 0.36, *p* = 1.5 × 10^−8^).

### Consistency of findings with earlier studies

As our results might reflect genetic effects on more general cognitive abilities, we also show the GWAS results for visuo-spatial memory test scores in the UK Biobank sample (*N* = 336,881; http://www.nealelab.is/uk-biobank) and Davies et al (2018) [28] GWAMA results for GCA in the Supplemental Table 1. Only SNPs in 3p21 showed significant association with GCA implying that associations between *CDH18*, 13q21, and 19q13.3 SNPs with VSTM and VL are not secondary to the effect of this loci on GCA or general memory processes, but may show specificity to verbal episodic memory. However, as the UK Biobank memory test has showed low test-retest reliability, these results need to be interpreted with caution [29]. Further, we examined if the top SNPs of this study also linked with brain structure [30–32] and function [33] in previous GWA studies (Supplementary Table S14). We noticed that all our 3p21 top SNPs were associated with smaller intracranial volume and larger alpha oscillation during rest and both 19q13.3 (*APOE*-*TOMM40-APOC1*) SNPs linked with smaller volumes of hippocampus, amygdala, and nucleus accumbens.

Finally, in Supplementary Table S9 we show that of the top candidate SNPs for episodic verbal memory phenotypes (e.g., in *KIBRA* [10], *CTNNBL1* [9], *SCN1A* [8], and *FASTKD2* [11]) [7, 9–16], our meta-analyses showed at least suggestive signals only at the *APOE*-*TOMM40-APOC1* complex (rs4420638, rs2075650, rs6857, and rs157582).

## Discussion

We studied if common genetic variants associated with VSTM and VL in 53,637 adults without history of stroke or dementia within the CHARGE consortium. We identified four novel loci for VSTM/VL. The top SNPs showed wide range of functional properties in the brain tissues: Some were eQTLs, meQTLs, or associated with tau or amyloid accumulation in the brain, and an aggregate polygenic score for VL associated with working memory activity in the right parieto-occipital cortex.

The first novel peak for VSTM locates at 5p14.3 and encompasses rs425724, an intronic SNP within *CDH18* (aka *CDH14* and *CDH24*) as the lead SNP. Functional effects of rs425724 remain poorly known, but Hi-C chromosomal interaction tests suggest that it may influence regulation of *CDH18* expression. *CDH18* is specifically expressed in the brain [34] and it belongs to the Type II classic cadherin family, which is involved in neuronal cell-adhesion [35]. Cadherins are critically important in the development of cells and synapses early in life, and in maintaining neuronal and synaptic structure in mature synapses [36]. Cadherins are also suggested to play a central role in synaptic plasticity in general, and in long-term potentiation (LTP), the molecular basis of learning and memory, in particular [37, 38]. Cadherin-related alterations in LTP have been demonstrated in pharmacological, gene knockout, and RNAi experiments [39, 40], but little is known about the role of genomic variation in cadherin genes in memory processes in humans. We report that rs425724 may affect specifically processing of verbal information. Interestingly, a variant in *CDH13* associated with *verbal* but not spatial working memory in patients with ADHD [41], pointing again towards modality specificity. Some studies exist linking cadherin genes with neurodevelopmental outcomes (Supplement 1).

We also discovered a new locus for VL in 3p21 containing 14 SNPs in high LD in a ~300 kb region that showed significant associations with VL. Of these variants, we replicated rs4687625 and rs2015971, both intronic to *NT5DC2*, and rs2015971, which is intronic to *STAB1*. This locus harbors several genes and gene-based analyses implicated 11 genes (*NT5DC2, STAB1, ITIH1, ITIH4*, *PBRM1, SMIM4*, *NEK4, GLT8D1, ITIH3, MUSTN1*, and *GNL3*). We identified several potentially functional variants at this locus. All significant 3p21 SNPs are either intronic or exonic, are significant eQTLs and mQTLs in brain tissues, and link with brain intracranial volume [30] and alpha oscillation [33] in the previous studies. Some are also considered deleterious or regulatory. Moreover, the locus is in a transcriptionally active region and, finally, SNP associations of 3p21 variants with VL colocalized with imputed expression of *POC1A* in the putamen. The putamen is part of a cortico-striatal loop and it receives input from different parts of the cortex and projects back to the cortex via the globus pallidus and thalamus. Traditionally it has been linked with motor control functions, but recently both neuroimaging studies [42, 43] and studies on effects of focal lesions [44] have suggested an additional role in memory functions. Prior studies have associated SNPs at 3p21 locus with various neurodevelopmental outcomes, such as GCA [28] and schizophrenia [45], but causal variant(s) are not known and in the studies with functional analyses, no specific gene has been conclusively shown to account for the many association findings at this locus (Supplement 1). Interestingly, a recent study reported an association between *GLT8D1*-variant rs6795646 and working memory in healthy Chinese persons [46].

We observed a third novel locus in the intergenic region in 13q21 in meta-analyses of discovery sample cohorts with paragraph recall tests to measure VSTM. The lead SNP was rs9528369 and the locus harbors 36 other significant SNPs. Again, the causal SNP or gene underlying this association is not known, but earlier studies point towards influences of this locus on language processing [47] and educational attainment [48] (Supplement 1). In line with this, rs9528369 showed no association with visuo-spatial memory test performance in the UK Biobank sample (http://www.nealelab.is/uk-biobank). Functional influences of this locus remain poorly understood, but rs9528369 was a mQTL in the dorsolateral prefrontal cortex and Hi-C analyses of this study showed chromatin-chromatin interactions with the promoter region of *TDRD3* in brain tissue and *PCDH20* in other tissues. TDRD3 is part of the TOP3beta-TDRD3-FMRP complex, and *TOP3beta* deletion was recently linked with schizophrenia, cognitive impairment, and learning difficulties [49], while lack of FMRP causes the Fragile X syndrome characterized by severe learning deficits and mental retardation.

In line with Debette et al. [7] in the GWAS for long-term verbal memory, we showed that rs4420638 in the *APOE*-*TOMM40-APOC1* locus at 19q13.3 is associated consistently with VSTM, especially paragraph recall, and overall VL and visually presented VL test scores. Also, rs6857 associated with VL. It is near *PVRL2* and locates ~30 kb downstream from rs4420638 and is in LD with rs4420638. Both significant SNPs are located near transcriptionally active region, associate with tau accumulation, and with the size of the memory-relevant regions (e.g., hippocampus) [31, 32]. Prior studies have linked many SNPs in this locus with a variety of cognitive outcomes and dementias although not previously with VSTM or VL in cognitively normal adults (Supplement 1) [6, 7, 16]. These various signals may merely reflect an impact of genetic variation at the *APOE* locus or suggest that additional genes in this region are involved in episodic memory, but this distinction requires functional studies; the strong LD in this region precludes further conclusions based solely on genetic association studies.

We also showed gene-level associations and significant enrichment with genes expressed widely in the brain, especially in the cerebellum and the frontal cortex for VSTM, and the cerebellum and striatal nuclei for paragraph recall - a pattern that parallels one shown recently for the GCA [28]. Gene-based analyses implicated *AGXT2* and *CALN1* for VL, while analyses of protein-protein interactions implicated synaptic proteins previously associated with Alzheimer disease biology, *SYT9* and *NRXN1* for VSTM; *ZFAND5*, *GRIK2*, and *ZC3H18* for VL; and *PRLHR* for paragraph recall. There is some evidence that *AGXT2, CALN1, NRXN*, and *GRIK2* may influence neurodevelopmental outcomes (Supplement 1).

Previous fMRI studies on short-term word list recall associated performance with a network of brain regions including the medial temporal lobe, superior temporal gyrus, medial and inferior parietal cortex, and dorsolateral prefrontal cortex [50, 51]. Within this network, joint analysis of episodic and working memory tasks observed the involvement of the prefrontal cortex, supplementary motor area, and bilateral ventral posterior parietal cortex spanning into the extrastriate cortex [52]. Consistently, here we show that a polygenic score for VL associated with activity in the posterior parietal and extrastriate cortex during the N-back fMRI task. This association was not due to years of education. This visual association area is active during recognition memory [53, 54]. The association had a negative direction, consistent with N-back performance data which correlate negatively with frontoparietal network activity in healthy individuals. [55, 56]

The heritability estimates of ~6% for VSTM and 18% for VL are in line with a recent phenome-wide study that showed SNP-based estimates between 6% and 11% for visuo-spatial memory in the UK Biobank [57]. Moreover, our estimates are in line with a twin study showing lower estimates for VSTM than for VL [4]. In our study, VSTM and VL showed strong positive genetic correlations with each other and with GCA in adulthood, completion of college, and years of schooling, consistent with recent findings from the UK Biobank [58]; and VSTM with childhood GCA and VL with anorexia nervosa and father’s age at death. VSTM also showed negative genetic correlation with coronary artery disease, in agreement with a previous study showing a negative association between a polygenic risk score for cardiovascular disease and verbal short-term memory [59]. To our knowledge, no previous studies have suggested a shared genetic background between verbal episodic memory and anorexia nervosa. However, anorexia nervosa shows positive genetic correlation with years of education and attending college [60] and children born to mothers with anorexia nervosa have shown increased working memory capacity [61].

There are limitations to our study. Heterogeneity in the testing methods and phenotypes across cohorts may have hindered our ability to find associations. Since majority of the samples (91.2% for VSTM and 93.3% for VL) were imputed against the HapMap2 reference panel resulting in ~2.5 Million SNPs in the meta-analyses, re-analyses with higher resolution genotyping is warranted. Moreover, despite reporting GWAMA results of the largest sample with VSTM and VL, our study is still underpowered to detect all genomic variation related to verbal episodic memory and larger studies are needed. Finally, as VSTM and VL showed strong genetic correlation with GCA, it is possible that our results reflect genomic influences on GCA. However, there are several lines of evidence against this: of several cognitive abilities, memory has shown largest unique genetic variance [62], adjusting for educational attainment only marginally altered our results, and finally, of our lead SNPs only those in a highly pleiotropic region at 3q21 were implicated in the recent GWAS for GCA [28].

To sum up, we report the results of the largest GWAMA of verbal episodic memory. We show novel genome-wide significant associations between common SNPs in four loci, *CDH18*, 3p21, 13q21, and 19q13.3, and VSTM and VL, and link combined polygenic variation for VL with brain activity during working memory task in the parieto-occipital cortex. Whereas many SNPs in these loci, especially in 3p21 and in 19q13.3, have been linked to other neurocognitive outcomes and show functional significance and associations with brain structure and function, their exact biological role needs to be studied further. We also show moderate SNP-based heritability and high genetic correlation of these memory traits and GCA, as well as coronary artery disease and anorexia nervosa suggesting some shared biology. These results improve our understanding of the biology underlying learning and memory and could lead to improved risk stratification scores and new drug targets for preserving memory, and preventing or treating dementias.

## Online methods

### Participants

This study comprised 37 cohorts and 53,637 adult participants (age > 18 years) of European descent brought together by the Cohorts for Heart and Aging Research in Genomic Epidemiology (CHARGE) consortium. Exclusion criteria included clinical stroke and any form of prevalent dementia.

The discovery sample comprised 44,874 participants from 27 cohorts for VSTM and 28,909 participants from 22 cohorts for VL. Replication samples comprised 8763 participants (five cohorts) and 3853 participants (two cohorts) for VSTM and VL, respectively. All studies were approved by their institutional ethics review committees and all participants provided written informed consent. Characteristics of the study cohorts are shown in Supplementary information Table 1 and Supplement 1.

### Phenotypes

All verbal memory tests are standardized and validated and have shown psychometrically adequate properties. Cognitive tests were administered by trained personnel following standardized protocols and blind to genetic information. To assess VSTM, cohorts administered either word list tests, e.g., the California Verbal Learning Test (CVLT), or paragraph tests, e.g., the Paragraph/Story recall test in the Wechsler Memory Scale (WMS) test battery, with immediate recall (Supplement 1 and Supplementary information Table 3). In all tests, participants were asked to recall as many words or story elements as possible immediately after their presentation.

In addition, some of the word list tests, e.g., CVLT, RAVLT, and CERAD, included assessment of VL. In these tests, the recalled material was presented, either orally or visually, and recalled more than once, hence the tests are tapping into the ability to learn across trials. In these tests, the first round of recall was also used in the VSTM analyses. Thus, these cohorts contributed both to the VL meta-analyses and to the VSTM meta-analyses.

We decided a priori to run meta-analyses combining all cohorts with verbal episodic memory tests with immediate recall (VSTM) and another meta-analyses across cohorts that administered tests of verbal learning with immediate recall (VL). Following Debette et al. [7] we also ran additional meta-analyses combining only the cohorts that administered similar tests. In these meta-analyses, we combined cohorts with word list tests with immediate recall (VSTM word list), paragraph tests with immediate recall (VSTM paragraph recall), verbal learning tests with orally presented material (VL orally presented words), and finally, verbal learning tests with visually presented words (VL visually presented words).

### Genotyping, QC, and imputation

Genome-wide genotyping was conducted in each cohort on several platforms following manufacturer protocols. Quality control was performed independently for each study. In addition, each group performed genotype imputation with appropriate software using the HapMap Phase II release 22 reference panel (70% of the cohorts) or 1000 Genomes, Phase 1, Release v3 panel. To harmonize the datasets, we updated the SNP IDs in those cohorts with HapMap Phase II imputation to match 1000 genomes, phase 1, release v3 panel (hg 19) by using LiftOver tool. Imputation quality scores for each SNP were obtained from IMPUTE (“proper_info”) or MACH (“rsq_hat”). Details on the genotyping are presented in Supplementary Information Table 2.

### Cohort-level genome-wide association analyses

Each cohort applied multiple linear regressions with additive genetic effect models to test for phenotype-genotype association using ~2.5 million genotyped and/or imputed autosomal SNPs (cohorts with HapMap II imputation) and 10–12 million SNPs in cohorts with 1000 genomes, phase 1 imputation. In our primary model, we adjusted for sex, age, population substructure, and study-specific covariates if deemed appropriate such as clinical center for multi-center cohorts. Furthermore, in family-based studies we fitted familial relationships, if necessary. In the secondary model, we adjusted for primary model covariates and educational attainment.

### Meta-analyses and detection of genomic risk loci

We performed quality control of the cohort-level summary statistics before the meta-analyses with the QCGWAS R package, version 1.0–8 [63], in the cohorts with HapMap II imputed data and EasyQC version 9.0 [64] in the cohorts with 1000 Genomes imputed data. We conducted the meta-analyses using METAL software [21]. We used the sample-size weighting and fixed effect model approach. We ran meta-analyses first separately in the discovery and replication samples and then in the combined sample including both discovery and replication cohorts. At the meta-analysis stage, we filtered out SNPs with low minor allele frequency (MAF <1%), poor imputation quality (proper_info <0.4 for IMPUTE and rsq_hat <0.3), or small sample size in the meta-analyses (*N* < 4000). We applied genomic control correction. A threshold of *p* < 5 × 10^−8^ was pre-specified as genome-wide significant, while a threshold of *p* < 1 × 10^−6^ was considered suggestive genome-wide significant. We used lambda values and quantile–quantile (Q-Q) plots of observed versus expected –log10(*P* value) to examine the genome-wide distribution of *P* values for signs of excessive false positive results. Genomic inflation factors are shown in Supplementary Information Table 4.

We applied FUnctional Mapping and Annotation of genetic associations (FUMA) [22] with default values to detect individual significant SNPs (*p* < 5 × 10^−8^ and independent of other genome wide significant SNPs at *r*^2^ < 0.6) and corresponding genomic risk loci (independent significant SNPs with *r*^2^ ≥ 0.1 and distance <250 kb are assigned to the same genomic risk locus) based on the meta-analysis results.

We also report associations on visuo-spatial memory test scores (variable #399, “Number of incorrect matches in round”) in the UKBiobank sample (*N* = 336,881; http://www.nealelab.is/uk-biobank) and on GCA in the Davies et al. [28] for those SNPs showing at least suggestively significant results (*p* < 5 × 10^−6^) in our discovery cohort.

### Functional annotation

For each of the SNPs showing a significant genome-wide signal, we derived several indices suggesting functionality using FUMA [22]: a) annotations with ANNOVAR [65] and the Ensembl genes build 85; b) CADD (http://cadd.gs.washington.edu/) scores that reflect deleteriousness of variants computed by integrating 63 functional annotations and applying a cut-off score of 12.37 as previously suggested (in general the higher the CADD score the more deleterious the variant is likely to be) [23]; c) regulome DB scores indicating the level of evidence for a variant to be a regulatory element, with lower scores indicating stronger evidence [24]; d) 15-core chromatin states for 127 epigenomes as characterized by ChromHMM v1.10 derived from 5 chromatin markers (H3K4me3, H3K4me1, H3K36me3, H3K27me3, H3K9me3) [66]; e) significant brain-related eQTLs defined as FDR (gene *q*-value) ≤ 0.05, using eQTL information on gene expression in 13 brain tissues obtained from GTEx v7 (http://www.gtexportal.org/home/) [67, 68] and 10 brain tissues obtained from Braineac (http://www.braineac.org/) [69] databases; f) chromatin-chromatin interactions (using pre-processed significant loops filtered at FDR 0.05 (https://www.ncbi.nlm.nih.gov/geo/query/acc.cgi?acc=GSE87112) [70] between independent significant SNPs and gene promoter regions (predicted using DNase peaks and core 15-state chromatin state model (http://egg2.wustl.edu/roadmap/web_portal/DNase_reg.html#delieation) in Roadmap Epigenomics Project brain tissues (E007, E009, E010, E053, E054, E067, E068, E069, E070, E071, E072, E073, E074, E081, E082) [71].

Additionally, we tested if the top SNPs reaching genome-wide significance associated with i) methylation levels in the dorsolateral prefrontal cortex (DLPFC) in the participants of the ROSMAP cohort (*N* = 322) and ii) brain amyloid and tau burden in a sample of 183 persons from the Framingham Heart Study (FHS) Third Generation cohort (mean age 46 ± 8years, 44% women) who underwent positron emission tomography (PET) imaging (Please see Supplement 1 for methods).

### Gene-based, gene-set, and gene property analyses

We performed gene-based association analysis with MAGMA (v1.6) [72] with default settings as implemented in FUMA [22]. SNPs were assigned to protein coding genes obtained from Ensembl build 85. We applied Bonferroni correction and genome-wide significance was set at 2.777 × 10^−6^ (0.05/18,007).

We also performed MAGMA (v1.6) [72] competitive gene-set analysis, using the results of the gene-based analyses, to examine whether genes in a gene-set are more strongly associated with VSTM and VL than other genes. A total of 10,655 gene sets (curated gene sets: *N* = 4738, GO terms: *N* = 5917) from MsigDB v6.1 [73] were used. We applied Bonferroni correction and genome-wide significance was set at 4.69 × 10^−6^ (0.05/10,655).

In addition, we performed MAGMA tissue expression analysis as implemented in FUMA with default settings and GTEx v7 gene expression data. This test examines the (positive) relationship between highly expressed genes in a specific tissue and genetic associations with those phenotypes showing significant genes (VSTM, VL, and VSTM tests with paragraph recall).

### S-PrediXscan analyses

We used S-PrediXcan [25] to integrate eQTL information with GWAS summary statistics to identify genes for which genetically predicted expression levels are associated with VSTM and VL. We used expression weights derived from 13 brain tissues in the GTEx v7 database and LD information from the 1000 Genomes Project Phase 3 [74]. These data were processed with beta values and standard errors from the VSTM and VL GWAS to estimate the expression-GWAS association statistic. We used a transcriptome-wide significance threshold of *p* < 1.10 × 10^−6^, which is the Bonferroni-corrected threshold when adjusting for all brain tissues and genes and visualized the colocalization (if any) with locus compare plot (http://locuscompare.com/ /accessed 17.5.2019).

### PGS_VSTM_, PGS_VL,_ and brain activity during 2-Back working memory task

To compute the short-term memory (PGS_VSTM_) and verbal learning (PGS_VL_) polygenic scores, we obtained betas associating allele dose with performance for 115,414 and 57,689, respectively, linkage disequilibrium-independent (*R*^2^ < 0.1) index SNPs. We then computed a weighted sum of the cumulative SNP effects by summing the imputation probability for the reference allele of the index SNP, weighted by the effect size of association with performance, at each independent locus across the genome, as described elsewhere [75]. We analyzed fMRI data of 435 healthy adult (≥18 years) volunteers of Caucasian ancestry who participated in the Clinical Brain Disorders Branch Sibling Study of schizophrenia (Supplement 1). Participants were genotyped according to standard procedures. In the PGS, we included SNPs at whole-genome (*p* = 5 × 10^−8^), intermediate (*p* = 10^−4^), and nominal significance levels (*p* = 0.05). Participants performed the N-back task during fMRI (block design version: 2-Back vs. 0-Back, lasting 240 s) working memory (WM) task. This task is widely used in imaging genetics studies [76–78]. fMRI data collection, pre-processing, and analysis followed standard procedures (Supplement 1) [79]. We used SPM12 to perform multiple regression analyses using PGSs as predictors. We report results surviving p_FWE_ < 0.05 threshold at whole brain level masked by task activity with a minimum cluster extent of 10 voxels (Supplement 1). Results are illustrated at *p* < 0.001 (uncorrected) in Fig. 4.

### Protein-protein interactions with DAPPLE

We investigated a possible causal role for genes at the loci associated with VSTM and VL by searching for physical connections between proteins encoded by genes within these loci. The hypothesis is that causal genetic variants are likely to affect common mechanisms and these mechanisms may be revealed by these protein-protein interaction (PPI) networks. We performed the analyses using Disease Association Protein-Protein Link Evaluator (DAPPLE) [26] in GenePattern. DAPPLE searches for PPI in the InWeb database and assigned a probabilistic score. The InWeb database collects PPI data reported in the literature from numerous sources including IntAct, Reactome, the Molecular Interaction Database (MINT), the Biomolecular Interaction Network Database (BIND) and the Kyoto Encyclopaedia of Genes and Genomes (KEGG). DAPPLE constructs PPI networks where proteins are nodes and interactions in the InWeb databases are edges connecting the nodes. Input SNPs are those associated with memory phenotypes at *p* value < 0.10 and minor allele frequency >0.05. Genes harboring any of the input SNPs or those in LD (*r*^2^  >  0.5) with the input SNPs, or located within the closest recombination hotspots plus 50 kb are identified. Proteins coded by these genes are used to construct an interaction network. Four parameters are estimated for the observed network: (1) number of edges in the direct network; (2) the average number of proteins with which each seed protein directly interacts; (3) the average number of proteins with which each seed protein indirectly interacts; (4) the average number of seed proteins bound by common interactor (CI) proteins. The distributions of these estimates are then enumerated via 20,000 permutations by randomly reassigning proteins of the same binding degree (i.e., the total number of interactions a protein has in the InWeb database) as the proteins in the observed network to each node. Individual seed proteins are then scored based on their presence in direct and indirect networks. The significance of these scores are evaluated in the same permutation procedure and Bonferroni-corrected for the number of possible candidate proteins from each locus to prioritize genes (pcorr < 0.05).

### Genetic correlation analyses

We used LDscore (LDSC) regression as implemented in LD Hub [27] to estimate the degree of overlap between the polygenic architecture of the traits. We estimated genetic correlations between verbal episodic memory traits and traits that may be phenotypically linked with memory (categories: Neurological, Psychiatric, Brain volume, Aging, Cognitive, Education, Cardiometabolic, and Glycemic). In these analyses, we excluded the American cohorts as their consent precluded the use of their data to examine an association with education. Therefore, sample size was 26,977 in the analyses of genetic correlation with VSTM and 25,180 in the analyses of genetic correlation with the VL. We used FDR correction to account for multiple comparisons. Heritability z-scores were 4.9 and 7.4 for VSTM and VL, respectively, suggesting that the datasets for both traits are suitable for LDSC analyses.

## Supplementary information

Supplementary tables S1-S6

Supplementary figures

Supplementary cohort information tables S1-S4

Supplementary materials

## Data Availability

Code of the primary statistical analyses can be obtained from the corresponding author.
