# Supplementary material for: Genome-wide meta-analyses reveal novel loci for verbal short-term memory and learning
Source: Mol Psychiatry. Author manuscript; Available in PMC 2022 Dec 12. (PMC9734053; doi:10.1038/s41380-022-01710-8)
Supplement: Supplementary_figures [file NIHMS1840537-supplement-Supplementary_figures.docx]

**Supplementary Figures**


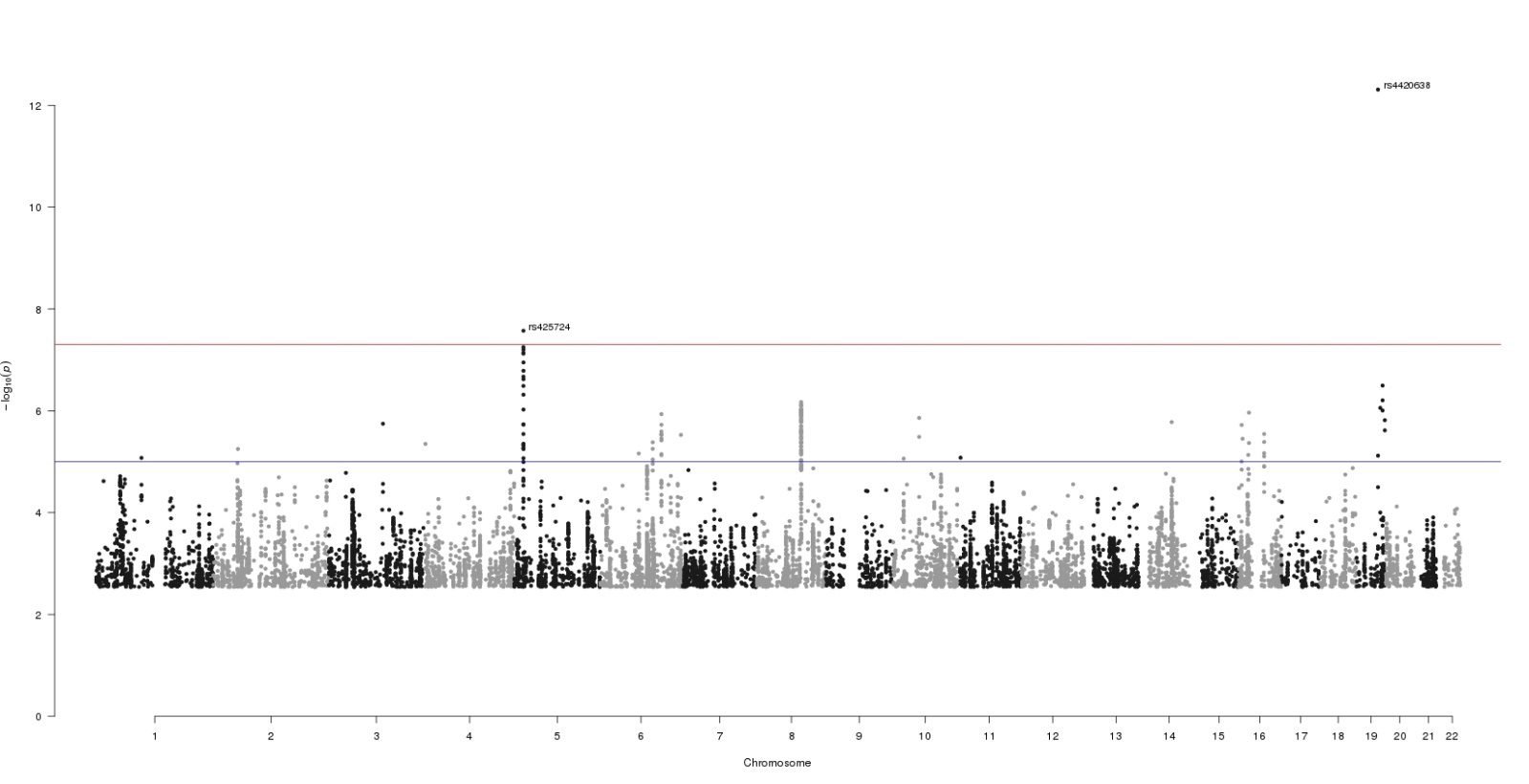


**Supplementary Figure 1:** The Manhattan plot for the genome-wide association meta-analysis of verbal short-term memory (VSTM) with 26 cohorts (N=44,874) of European descent. The −log10(P) values for the association tests (two-tailed) are shown on the y axis and the chromosomes are ordered on the x axis. Two genetic loci surpassed the genome-wide significance threshold (−log10(P) > 7.3; indicated by the red line and rs-number of the lead SNP). The black and grey colors differentiate adjacent chromosomes.

**
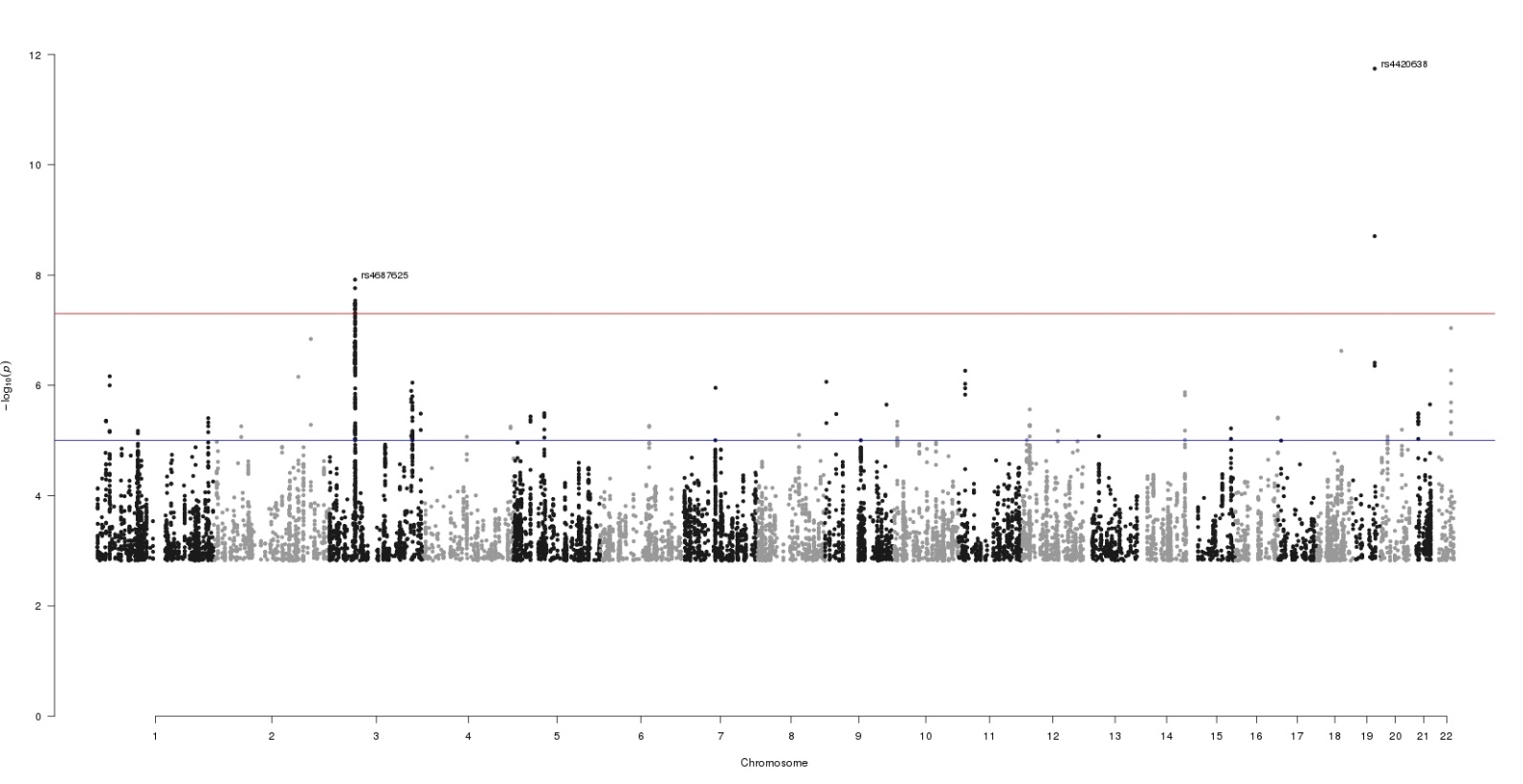
**

**Supplementary Figure 2:** The Manhattan plot for the genome-wide association meta-analysis of verbal learning (VL) with 17 cohorts (N=28,909) of European descent. The −log10(P) values for the association tests (two-tailed) are shown on the y axis and the chromosomes are ordered on the x axis. Two genetic loci surpassed the genome-wide significance threshold (−log10(P) > 7.3; indicated by the red line and rs-number of the lead SNP). The black and grey colors differentiate adjacent chromosomes.

**
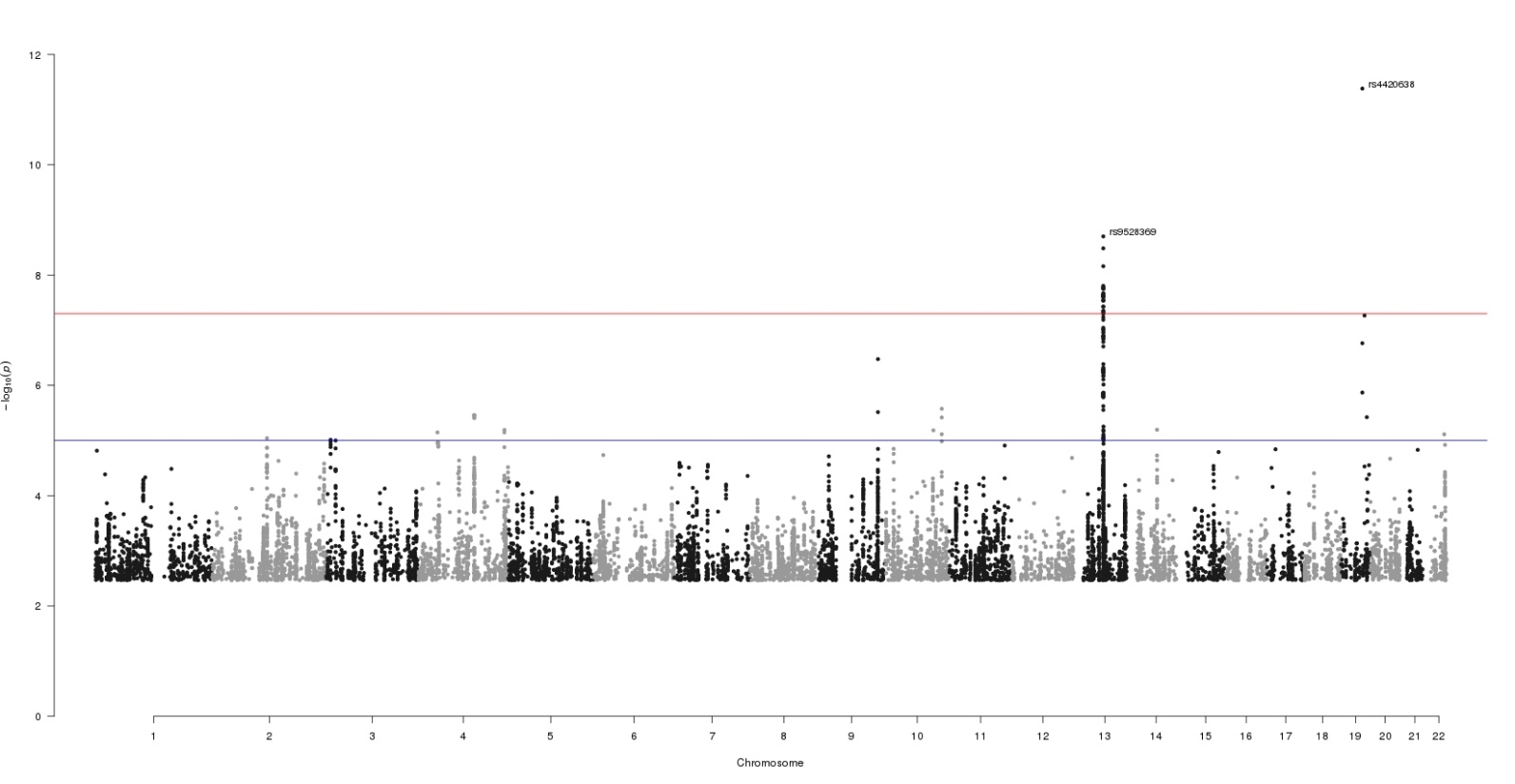
**

**Supplementary Figure 3:** The Manhattan plot for the genome-wide association meta-analysis of verbal short-term memory: paragraph recall tests with 13 cohorts (N=19,420) of European descent. The −log10(P) values for the association tests (two-tailed) are shown on the y axis and the chromosomes are ordered on the x axis. Two genetic loci surpassed the genome-wide significance threshold (−log10(P) > 7.3; indicated by the red line and rs-number of the lead SNP). The black and grey colors differentiate adjacent chromosomes.

**
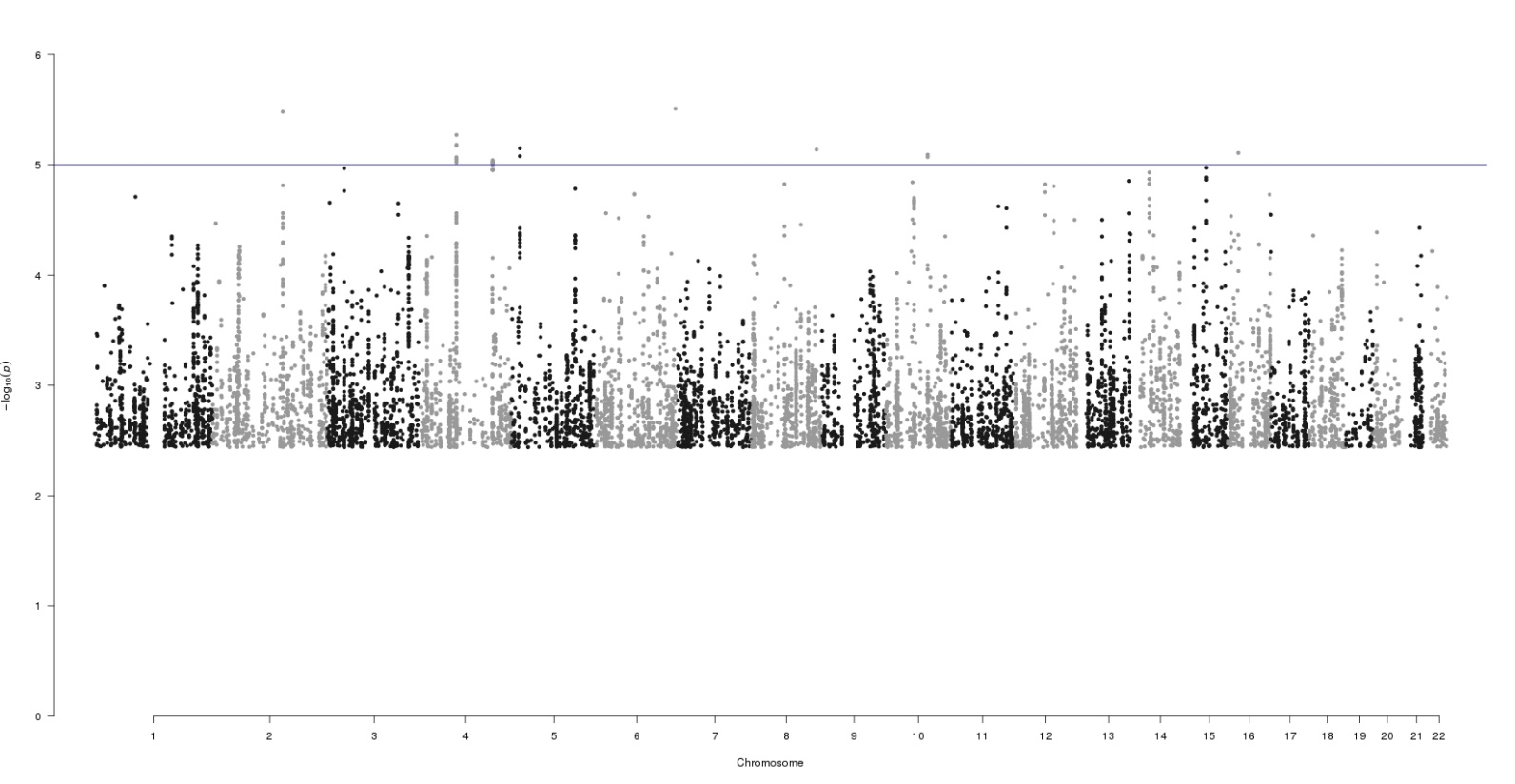
**

**Supplementary Figure 4:** The Manhattan plot for the genome-wide association meta-analysis of verbal short-term memory: word list recall tests with 14 cohorts (N=25,454) of European descent. The −log10(P) values for the association tests (two-tailed) are shown on the y axis and the chromosomes are ordered on the x axis. No genetic loci surpassed the genome-wide significance threshold (−log10(P) > 7.3). The black and grey colors differentiate adjacent chromosomes.


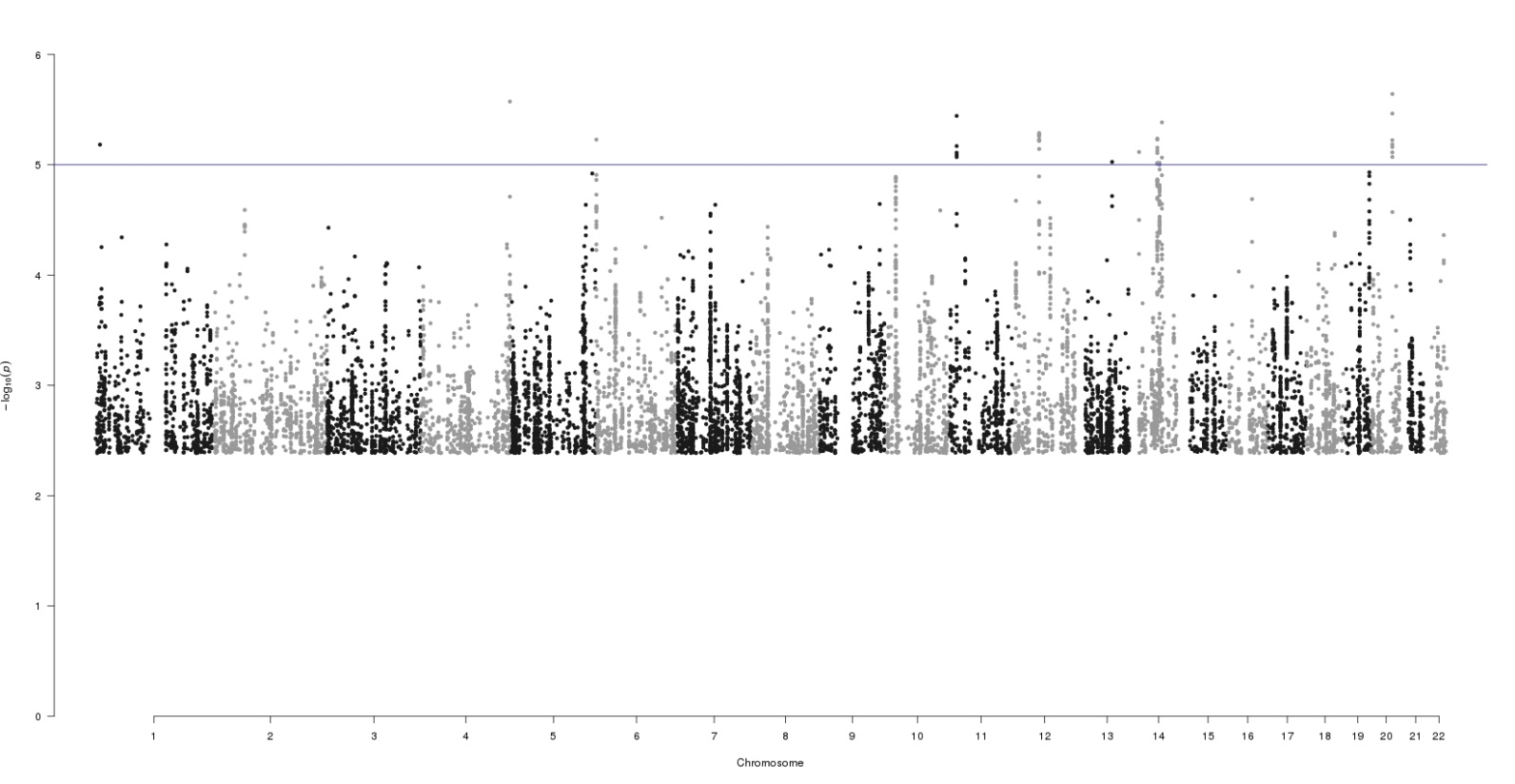


**Supplementary Figure 5:** The Manhattan plot for the genome-wide association meta-analysis of verbal learning: verbally presented content with 11 cohorts (N=12,593) of European descent. The −log10(P) values for the association tests (two-tailed) are shown on the y axis and the chromosomes are ordered on the x axis. No genetic loci surpassed the genome-wide significance threshold (−log10(P) > 7.3). The black and grey colors differentiate adjacent chromosomes.

**
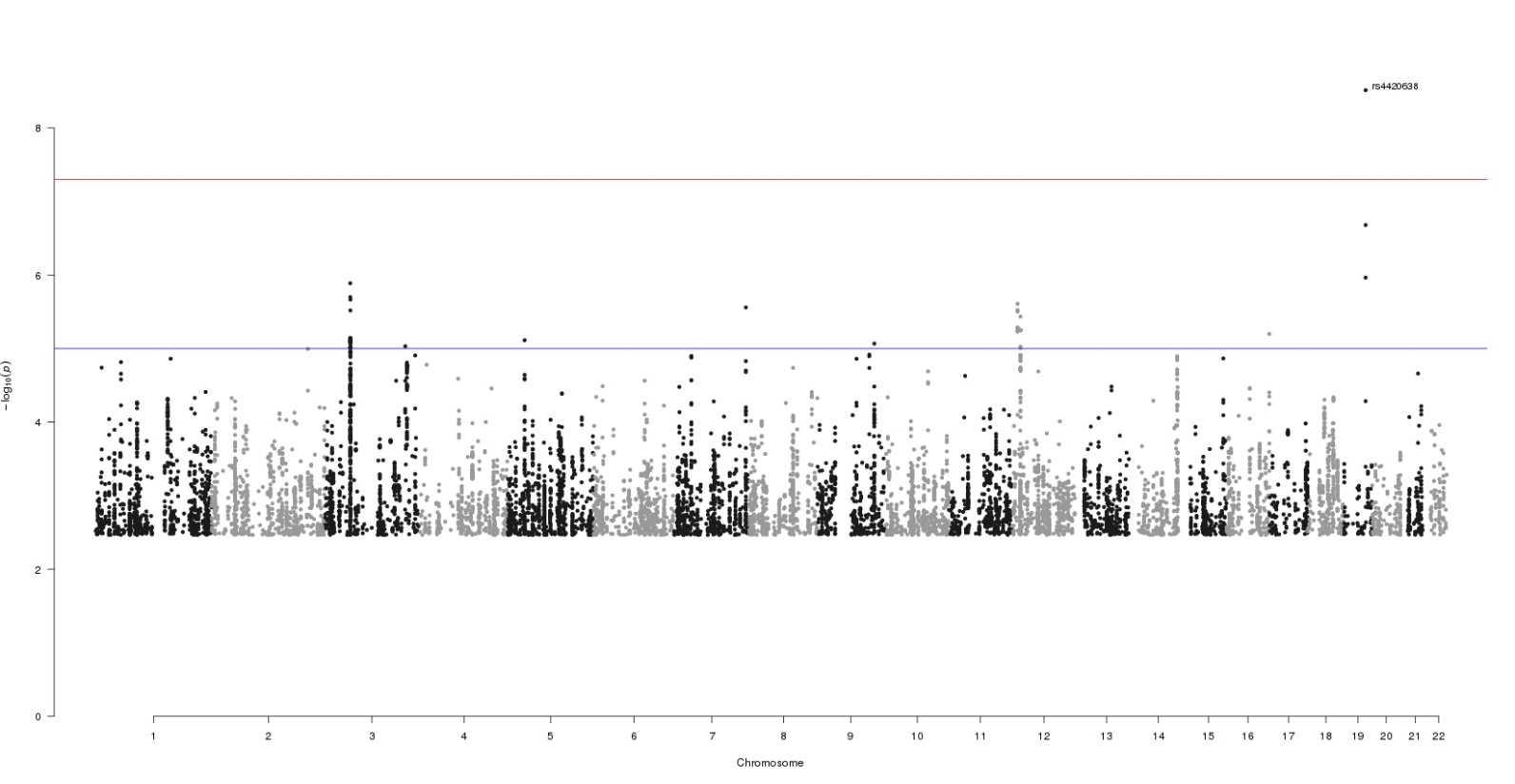
**

**Supplementary Figure 6:** The Manhattan plot for the genome-wide association meta-analysis of verbal learning: visually presented content with 11 cohorts (N=16,191) of European descent. The −log10(P) values for the association tests (two-tailed) are shown on the y axis and the chromosomes are ordered on the x axis. One genetic loci surpassed the genome-wide significance threshold (−log10(P) > 7.3; indicated by the red line and rs-number of the lead SNP). The black and grey colors differentiate adjacent chromosomes.


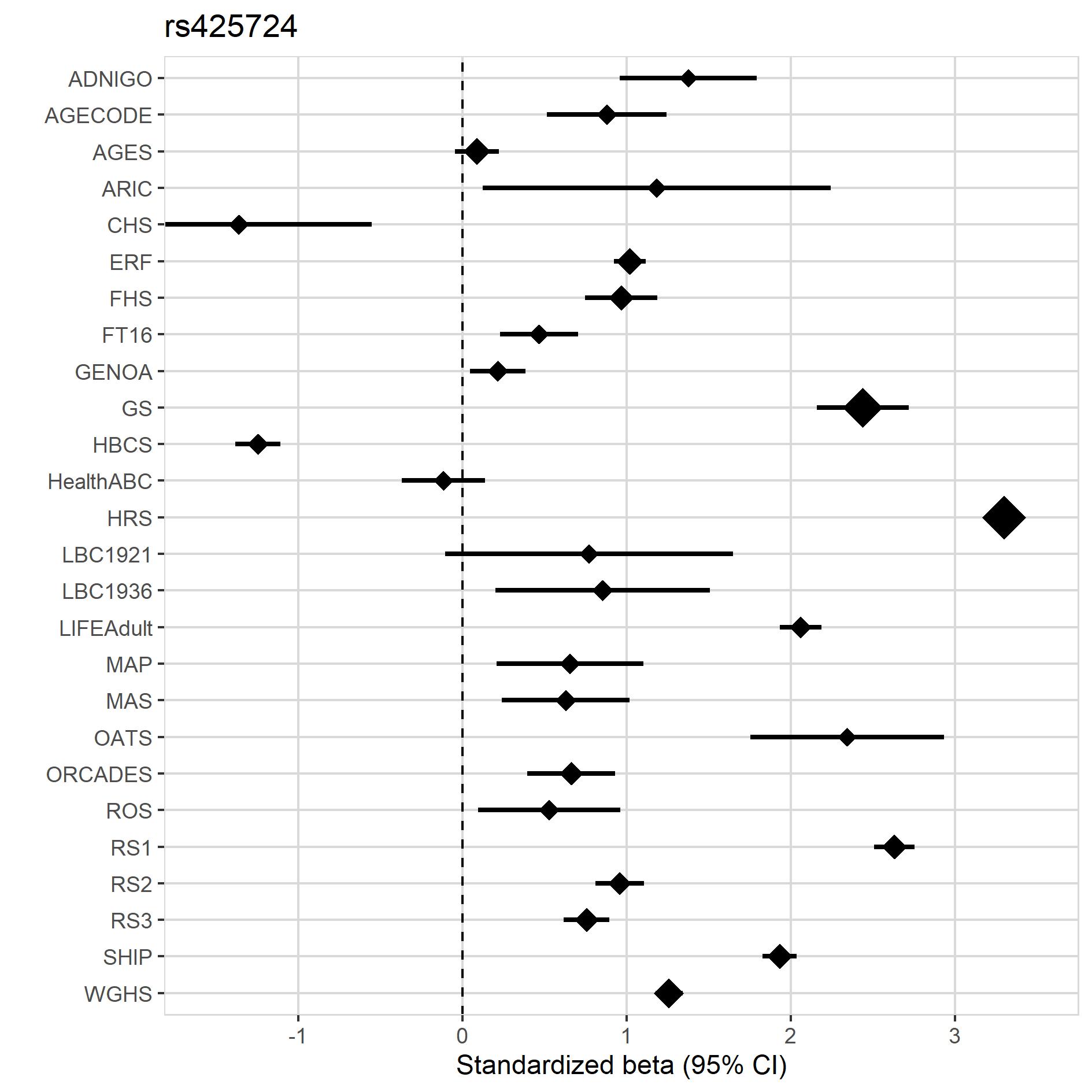


**Supplementary Figure 7:** Forest plot of associations between rs425724 and verbal short-term memory across cohorts. Diamonds denote standardized betas (95% confidence interval [95% CI]) and size of the diamonds reflect sample size.


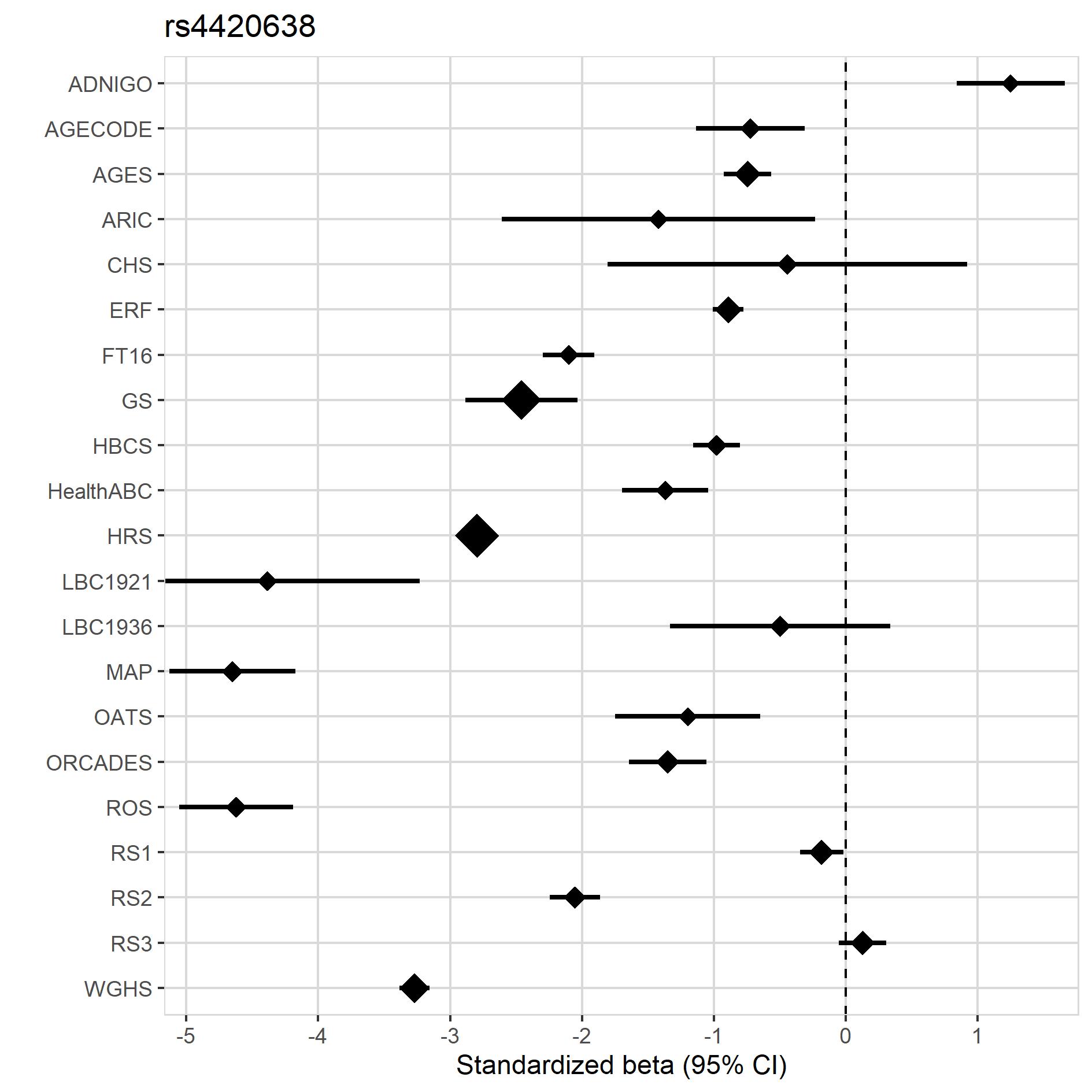


**Supplementary Figure 8:** Forest plot of associations between rs4420638 and verbal short-term memory across cohorts. Diamonds denote standardized betas (95% confidence interval [95% CI]) and size of the diamonds reflect sample size.


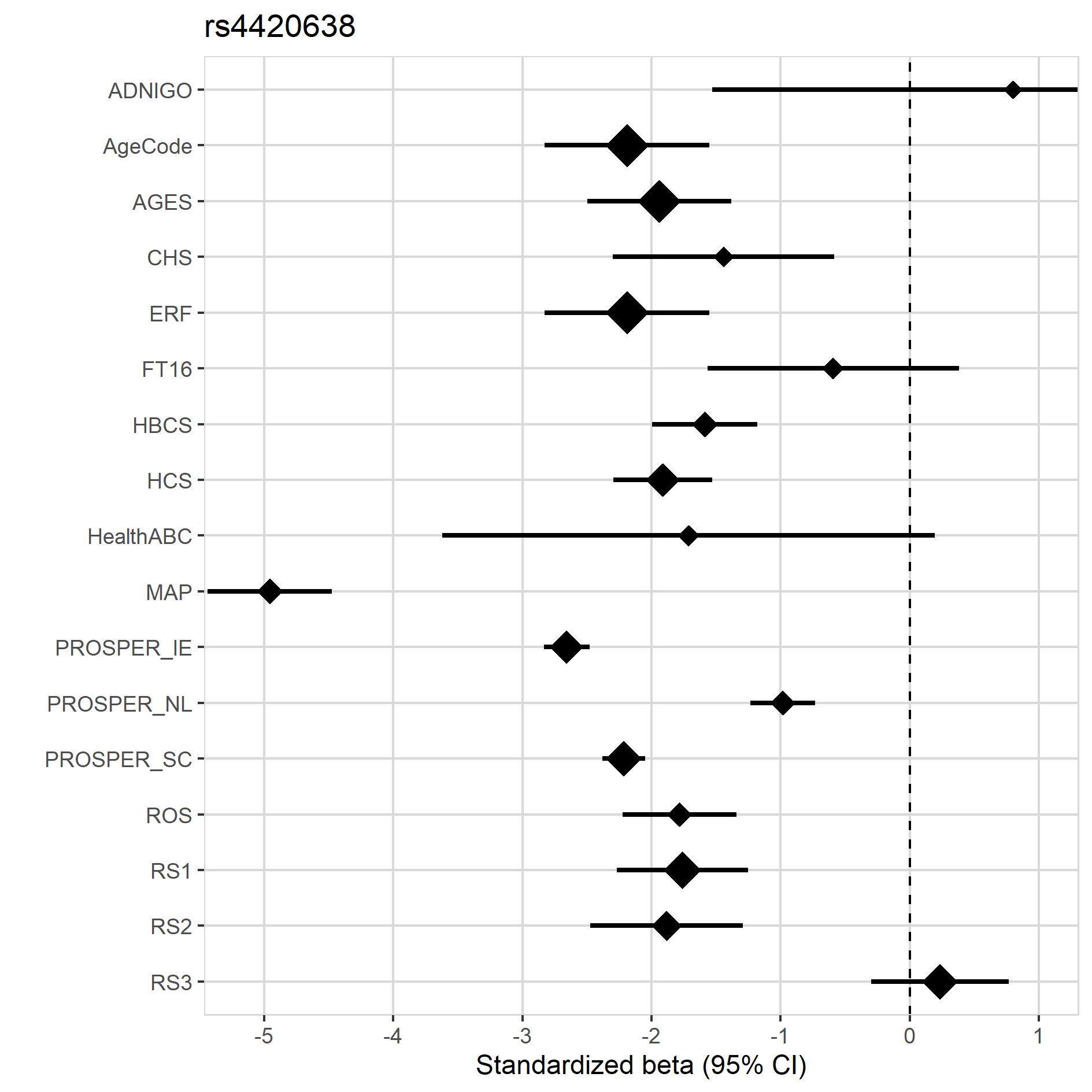


**Supplementary Figure 9:** Forest plot of associations between rs4420638 and verbal learning across cohorts. Diamonds denote standardized betas (95% confidence interval [95% CI]) and size of the diamonds reflect sample size.


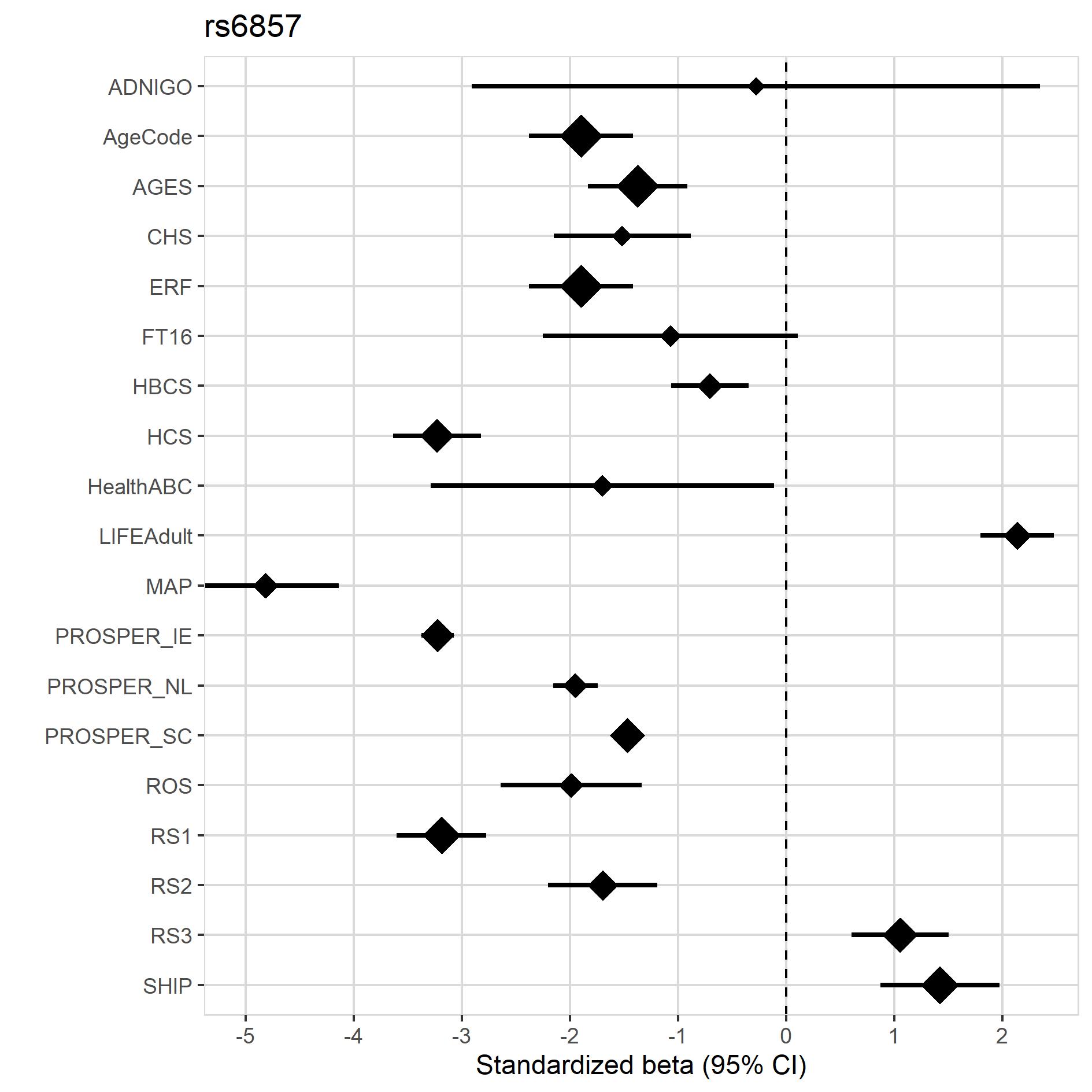


**Supplementary Figure 10:** Forest plot of associations between rs6857 and verbal learning across cohorts. Diamonds denote standardized betas (95% confidence interval [95% CI]) and size of the diamonds reflect sample size.


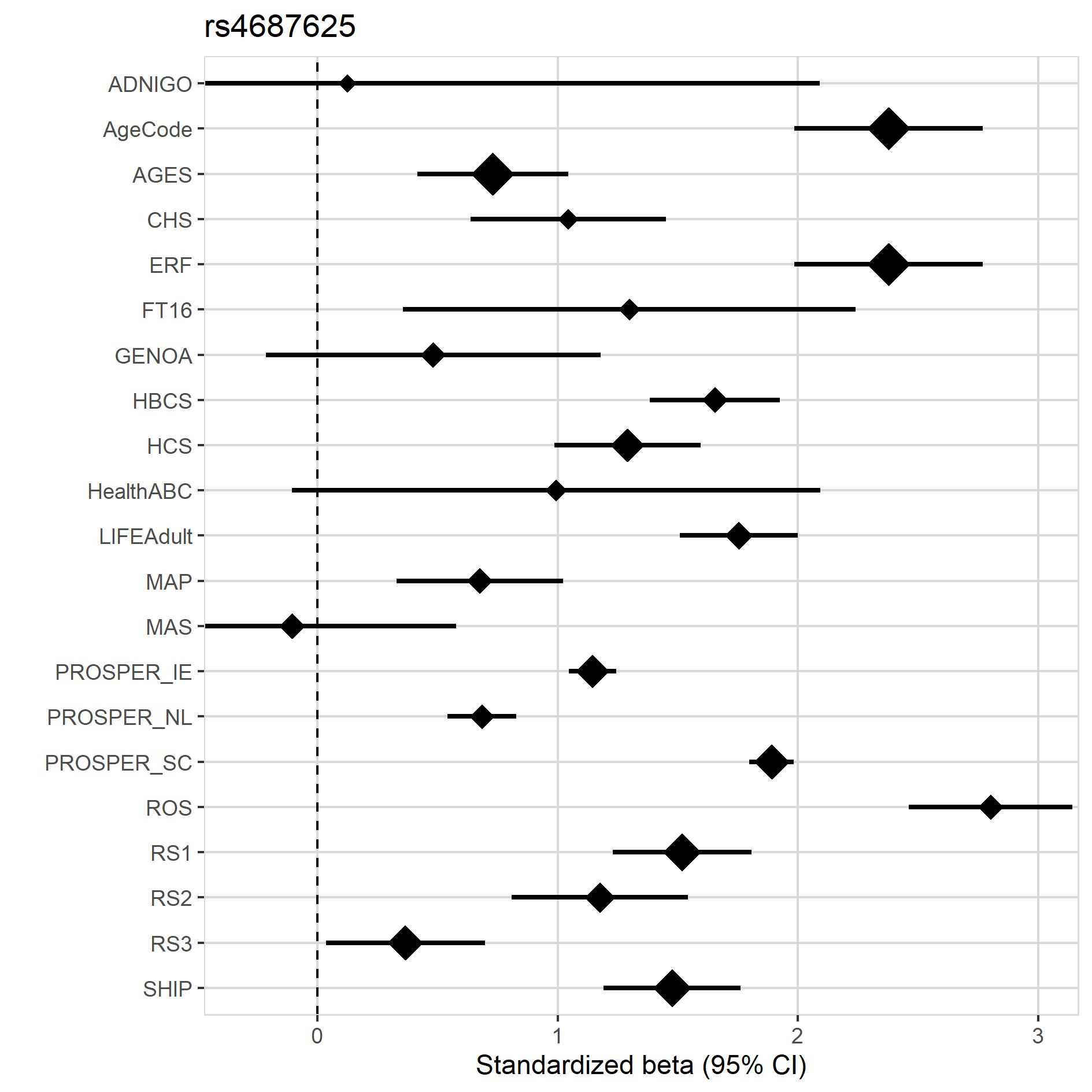


**Supplementary Figure 11:** Forest plot of associations between rs4687625 and verbal learning across cohorts. Diamonds denote standardized betas (95% confidence interval [95% CI]) and size of the diamonds reflect sample size.


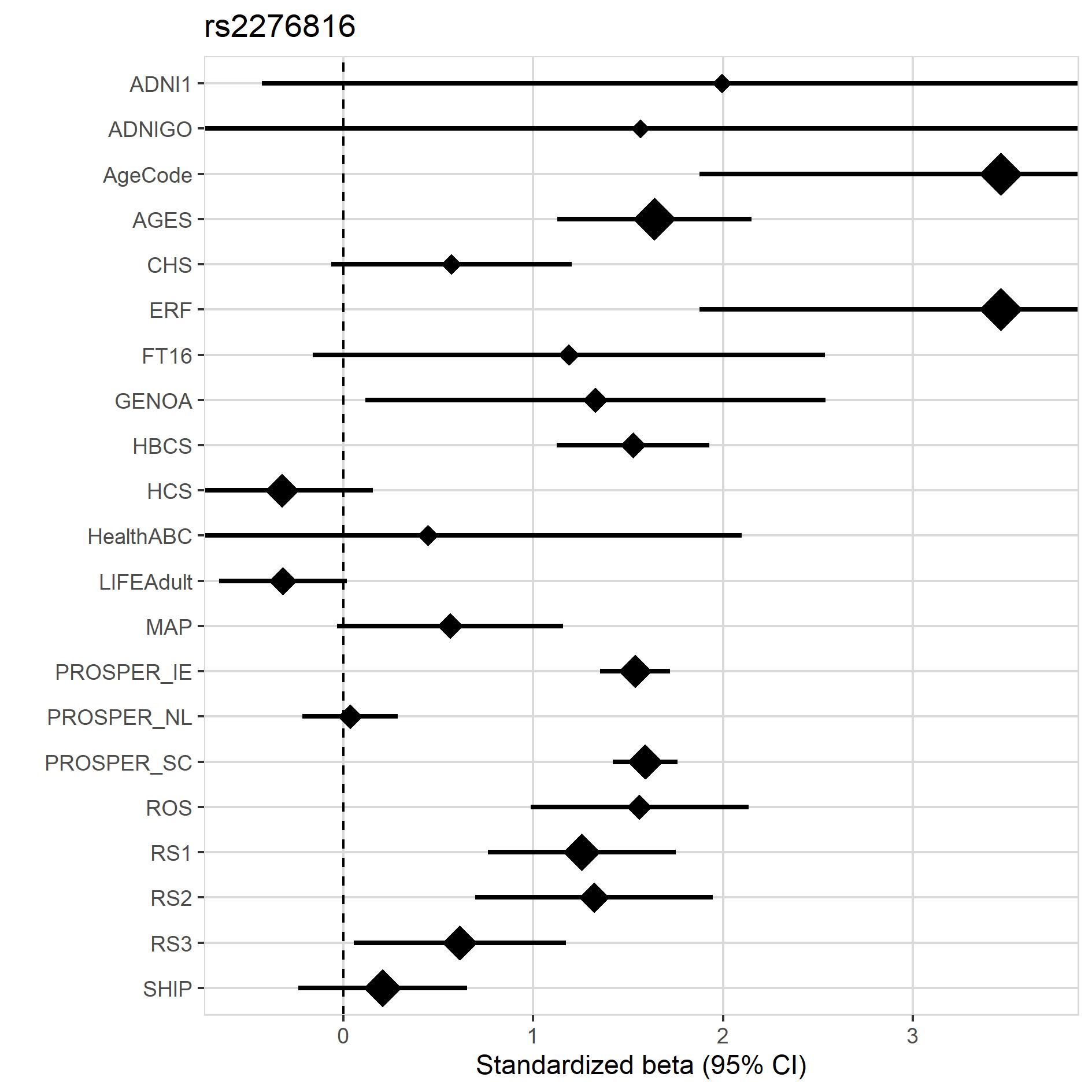


**Supplementary Figure 12:** Forest plot of associations between rs2276816 and verbal learning across cohorts. Diamonds denote standardized betas (95% confidence interval [95% CI]) and size of the diamonds reflect sample size.


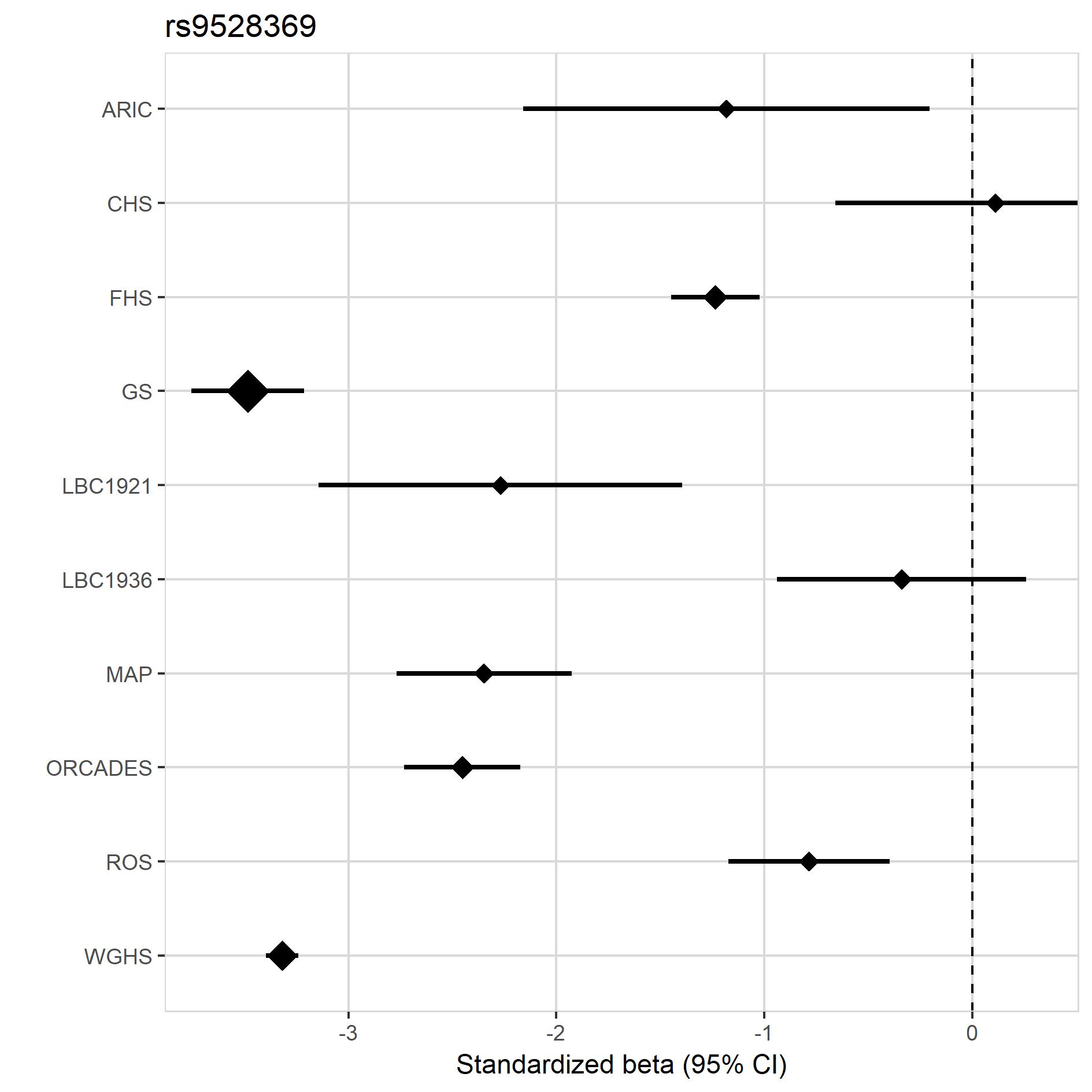


**Supplementary Figure 13:** Forest plot of associations between rs9528369 and verbal learning across cohorts. Diamonds denote standardized betas (95% confidence interval [95% CI]) and size of the diamonds reflect sample size.


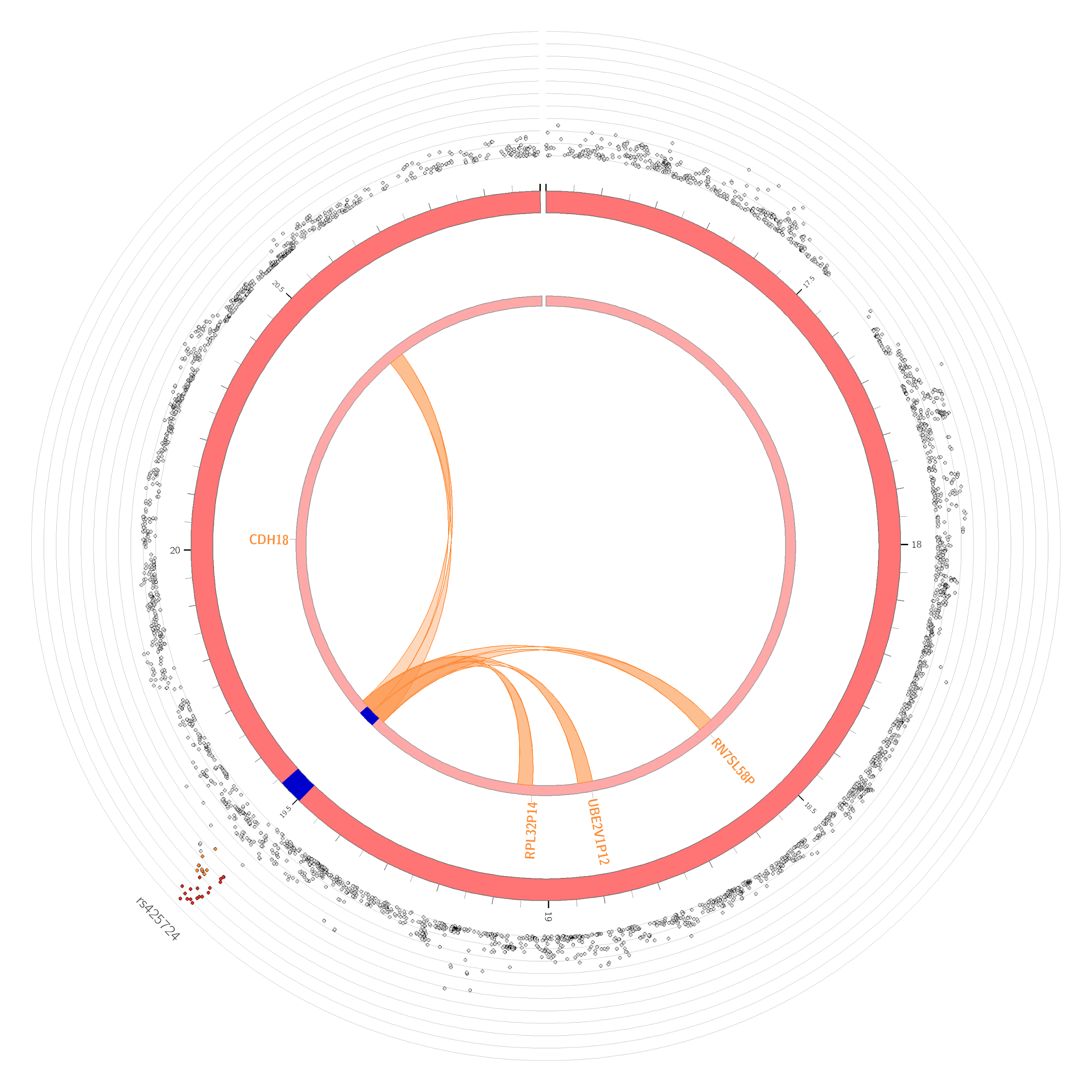


**Supplementary Figure 14:** Circos plot of chromosome 5 region implicated in verbal short-term memory. On the outer rim, there is Manhattan plot with rsID of the lead SNP(s) and genomic risk locus marked in blue. Chromatin interactions with mapped genes are marked in orange.


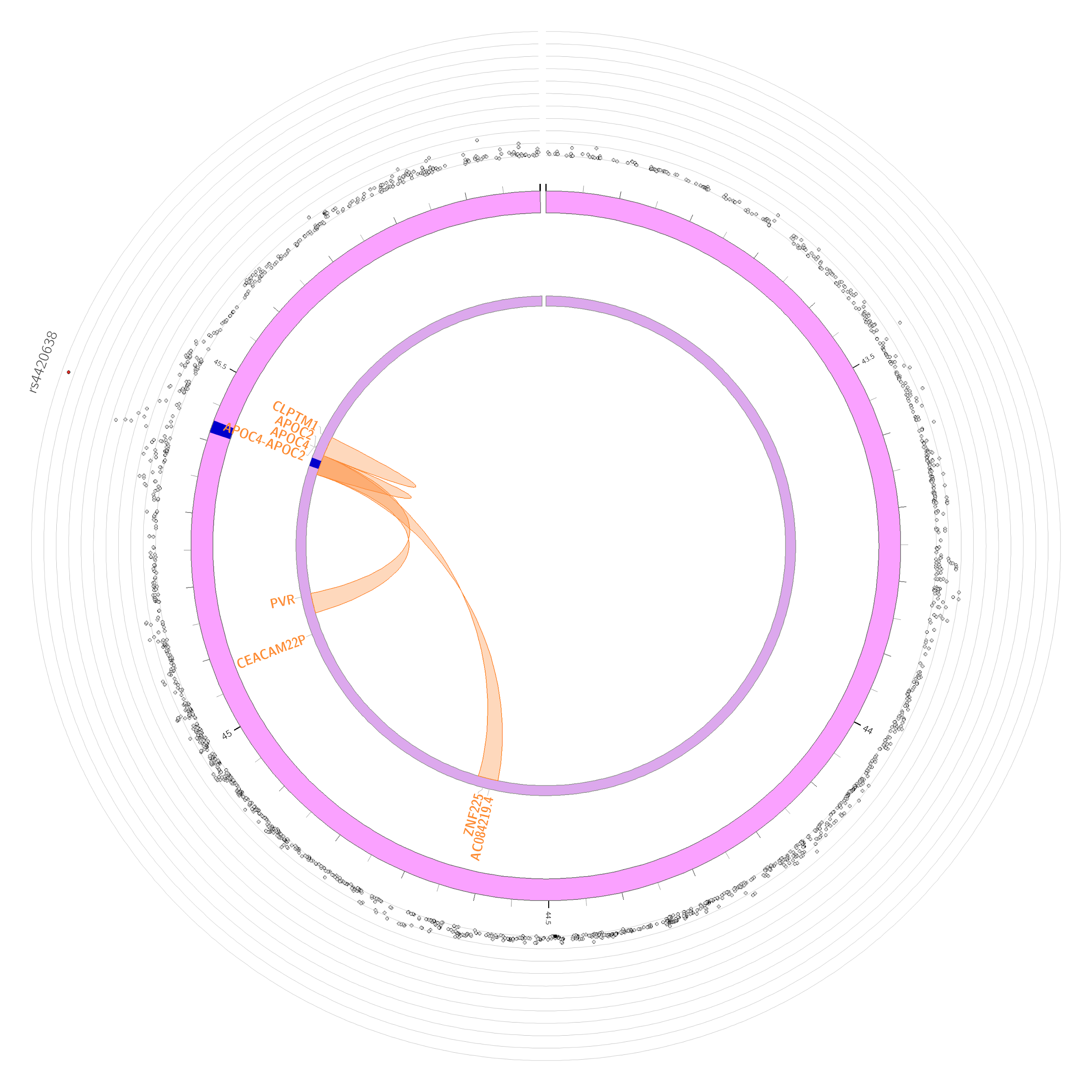


**Supplementary Figure 15**: Circos plot of chromosome 19 region implicated in verbal short-term memory. On the outer rim, there is Manhattan plot with rsID of the lead SNP(s) and genomic risk locus marked in blue. Chromatin interactions with mapped genes are marked in orange.

.


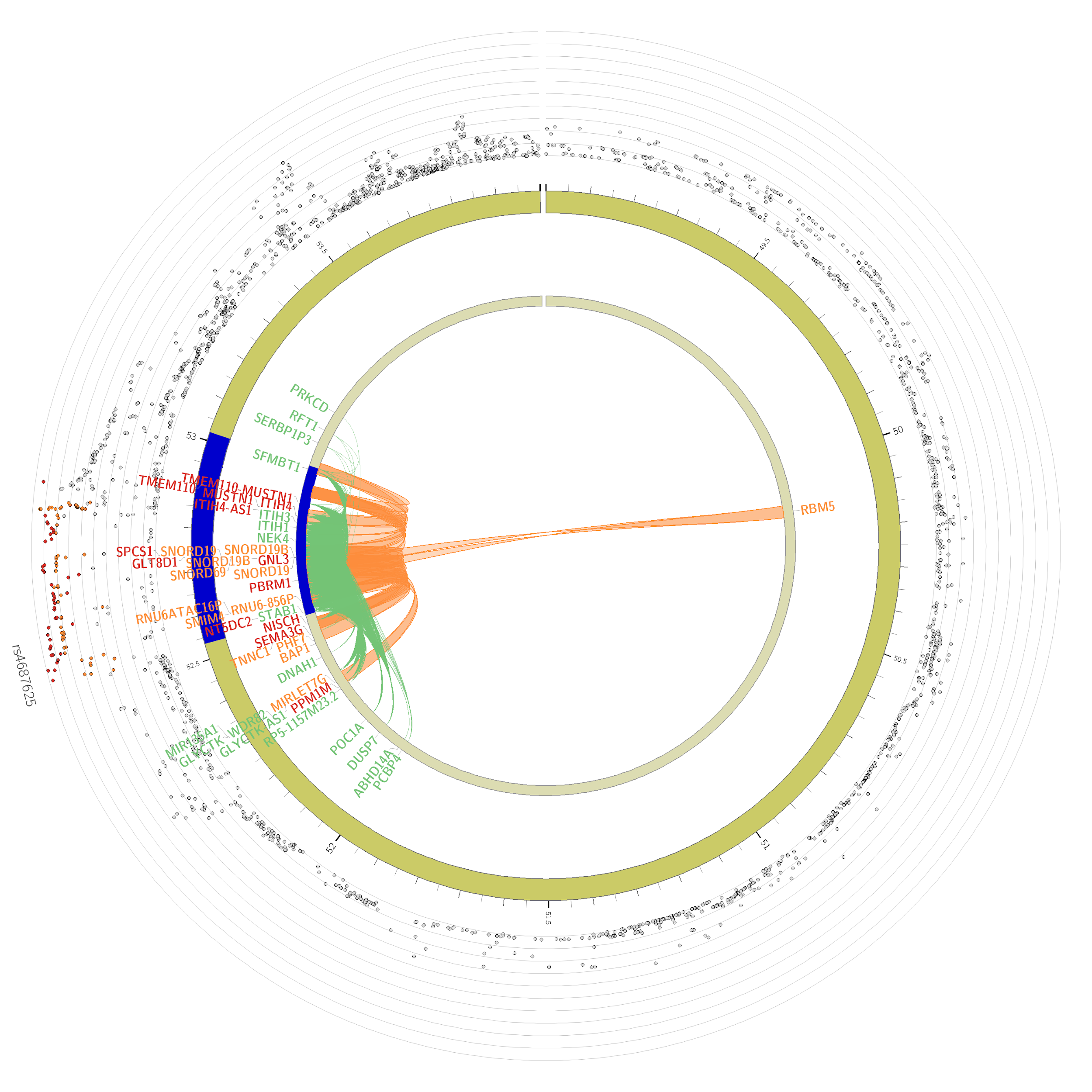


**Supplementary Figure 16**: Circos plot of chromosome 3 region implicated in verbal learning. On the outer rim, there is Manhattan plot with rsID of the lead SNP(s) and genomic risk locus marked in blue. Chromatin interactions with mapped genes are marked in orange and eQTLs with mapped genes are marked in green. Genes in red are mapped in both chromatin interaction and eQTL analyses.

**
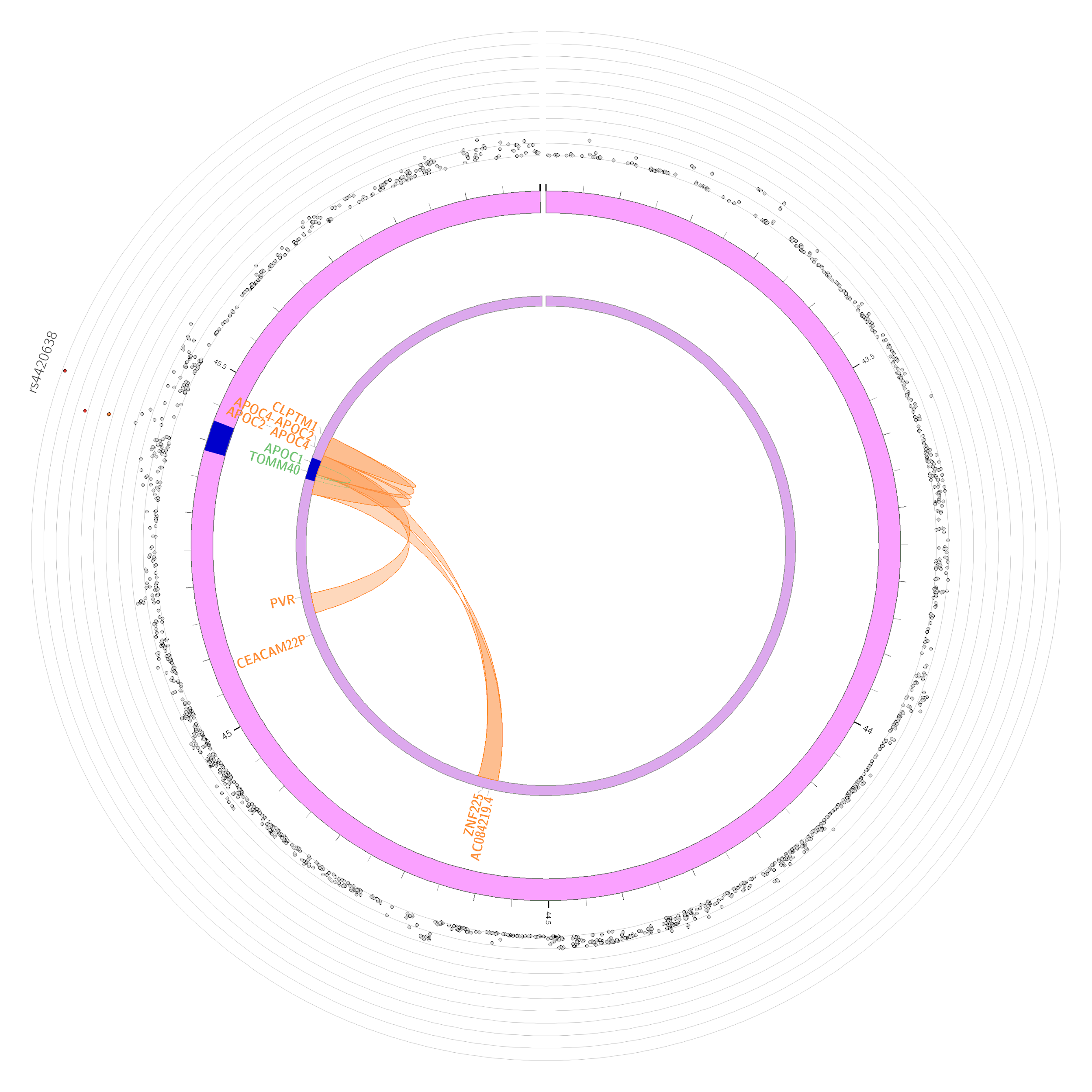
Supplementary Figure 17:** Circos plot of chromosome 19 region implicated in verbal learning. On the outer rim, there is Manhattan plot with rsID of the lead SNP(s) and genomic risk locus marked in blue. Chromatin interactions with mapped genes are marked in orange and eQTLs with mapped genes are marked in green.


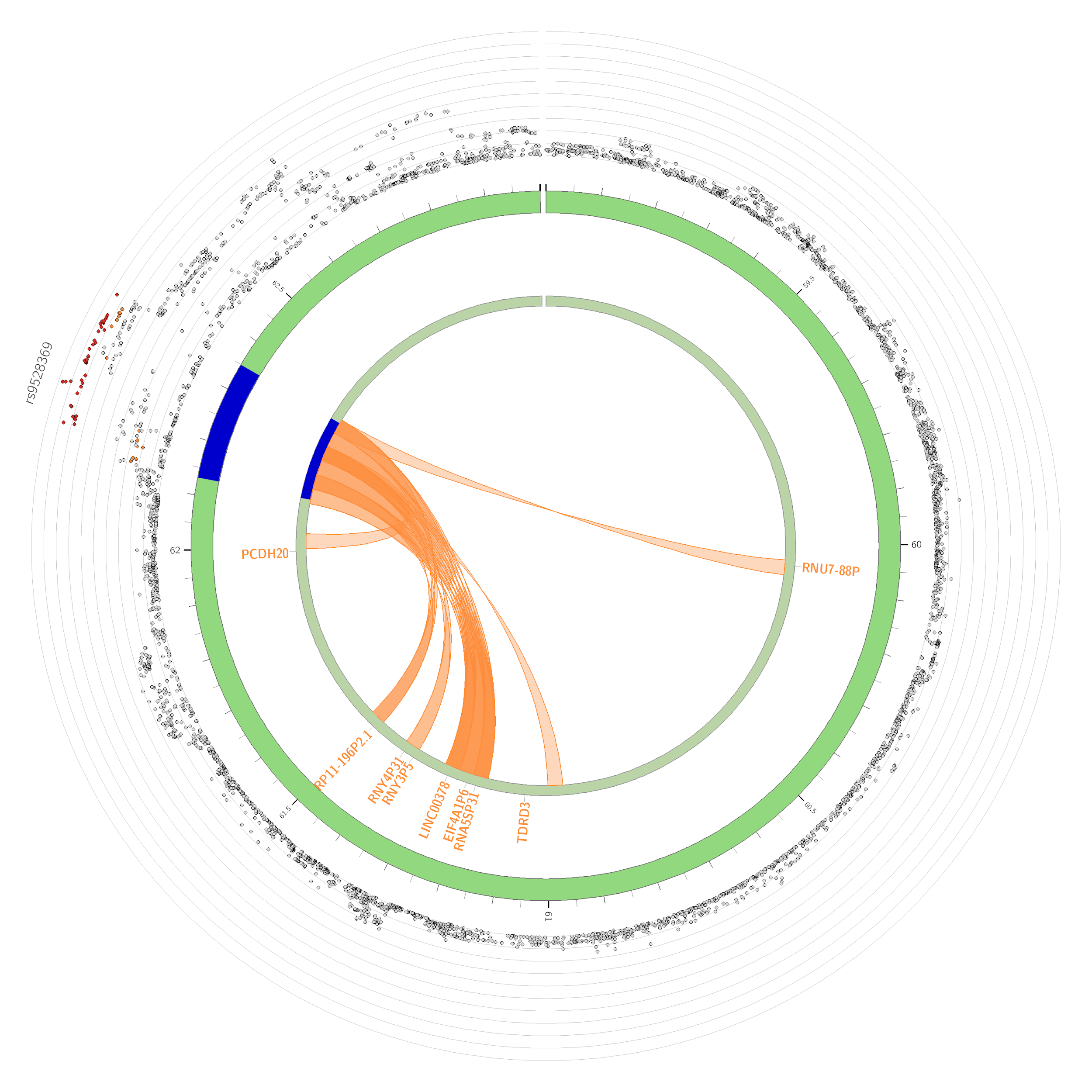


**Supplementary Figure 18**: Circos plot of chromosome 13 region implicated in paragraph recall. On the outer rim, there is Manhattan plot with rsID of the lead SNP(s) and genomic risk locus marked in blue. Chromatin interactions with mapped genes are marked in orange.


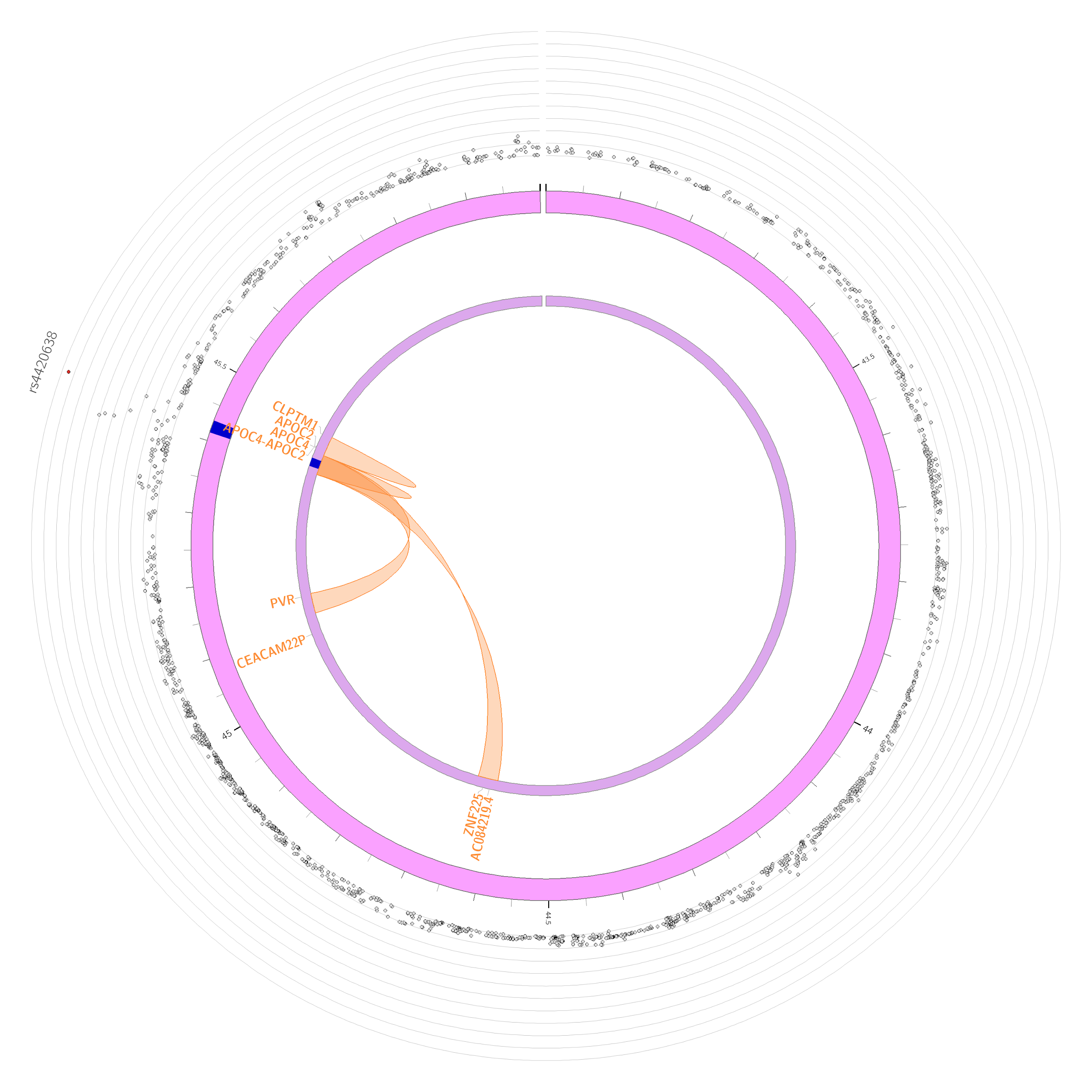


**Supplementary Figure 19:** Circos plot of chromosome 19 region implicated in paragraph recall. On the outer rim, there is Manhattan plot with rsID of the lead SNP(s) and genomic risk locus marked in blue. Chromatin interactions with mapped genes are marked in orange.


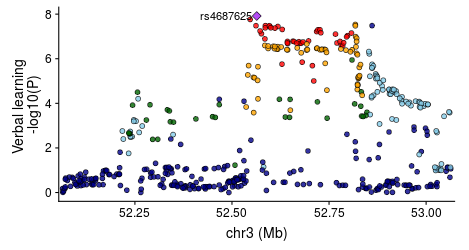


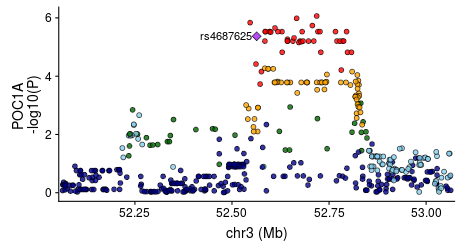


b

c

a


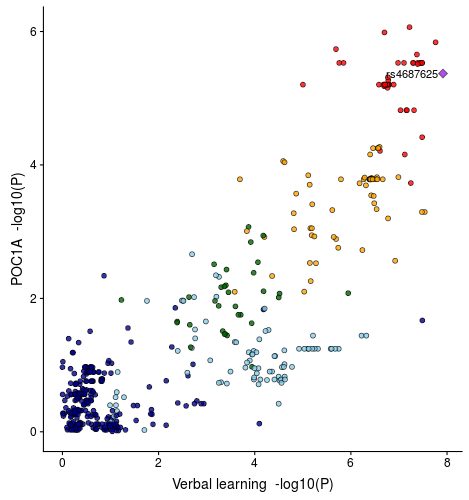


**Supplementary Figure 20**: Colocalization (a) between POC1A locus in verbal learning GWAS (b) results and *POC1A* eQTL in putamen (c). The eQTL p-values were extracted from the GTEx v7 Putamen (n = 124) based on a linear regression model.


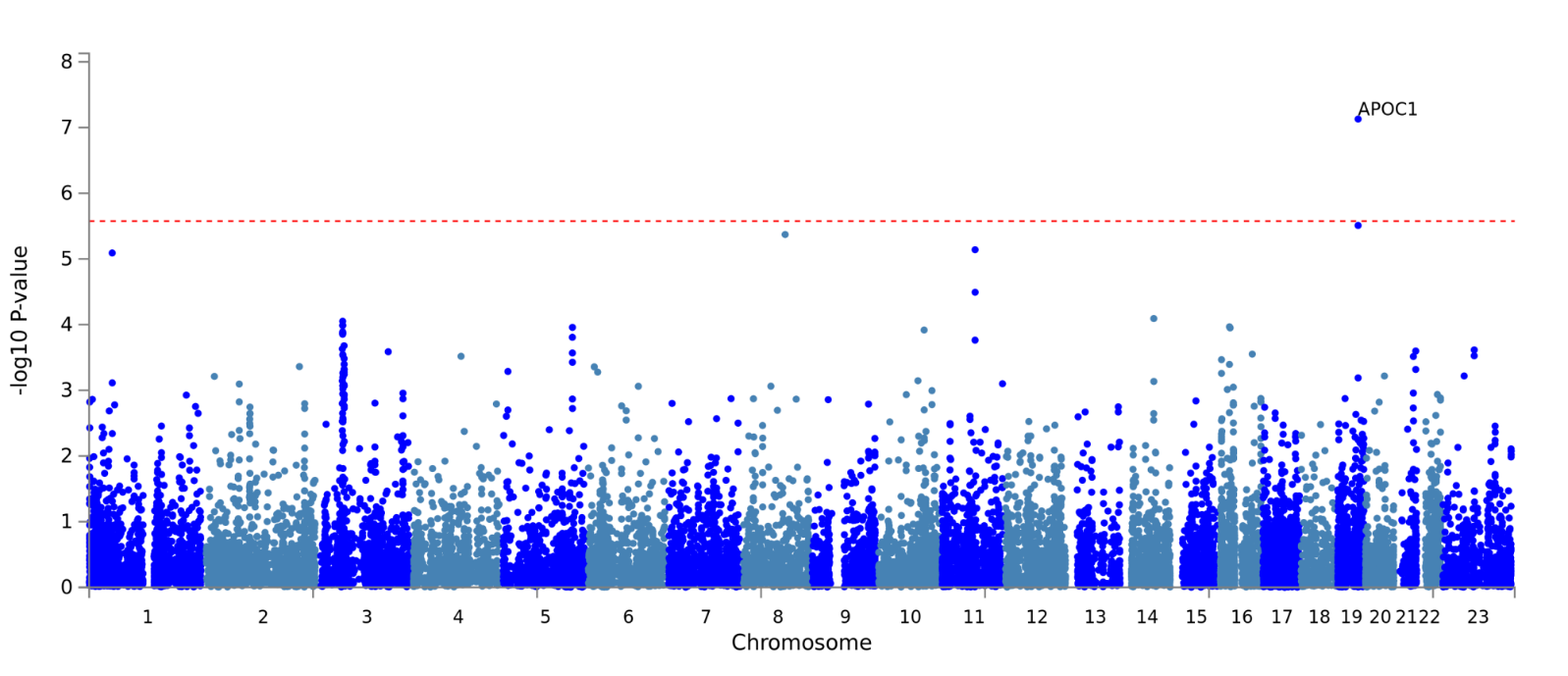


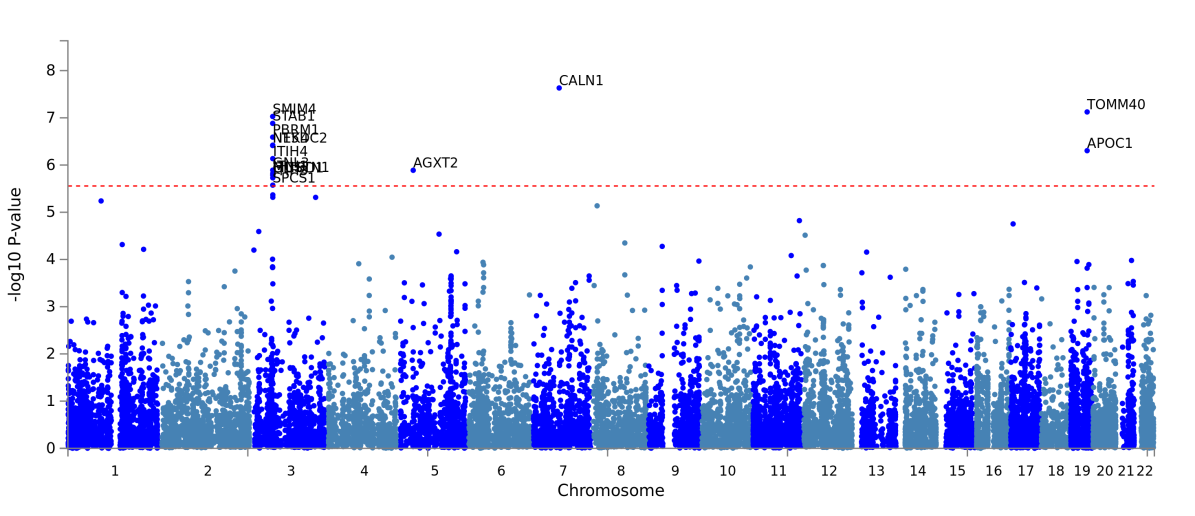


**Supplementary Figure 21**: The Manhattan plot for the gene-based analyses of verbal short-term memory (upper panel) and verbal learning (lower panel). The −log10(P) values for the association tests (two-tailed) are shown on the y axis and the chromosomes are ordered on the x axis. One and fifteen genetic loci surpassed the genome-wide significance threshold (−log10(P) > 5.33; indicated by the red dotted line) for verbal short-term memory (upper panel) and verbal learning (lower panel), respectively. The dark blue and light blue colors differentiate adjacent chromosomes.


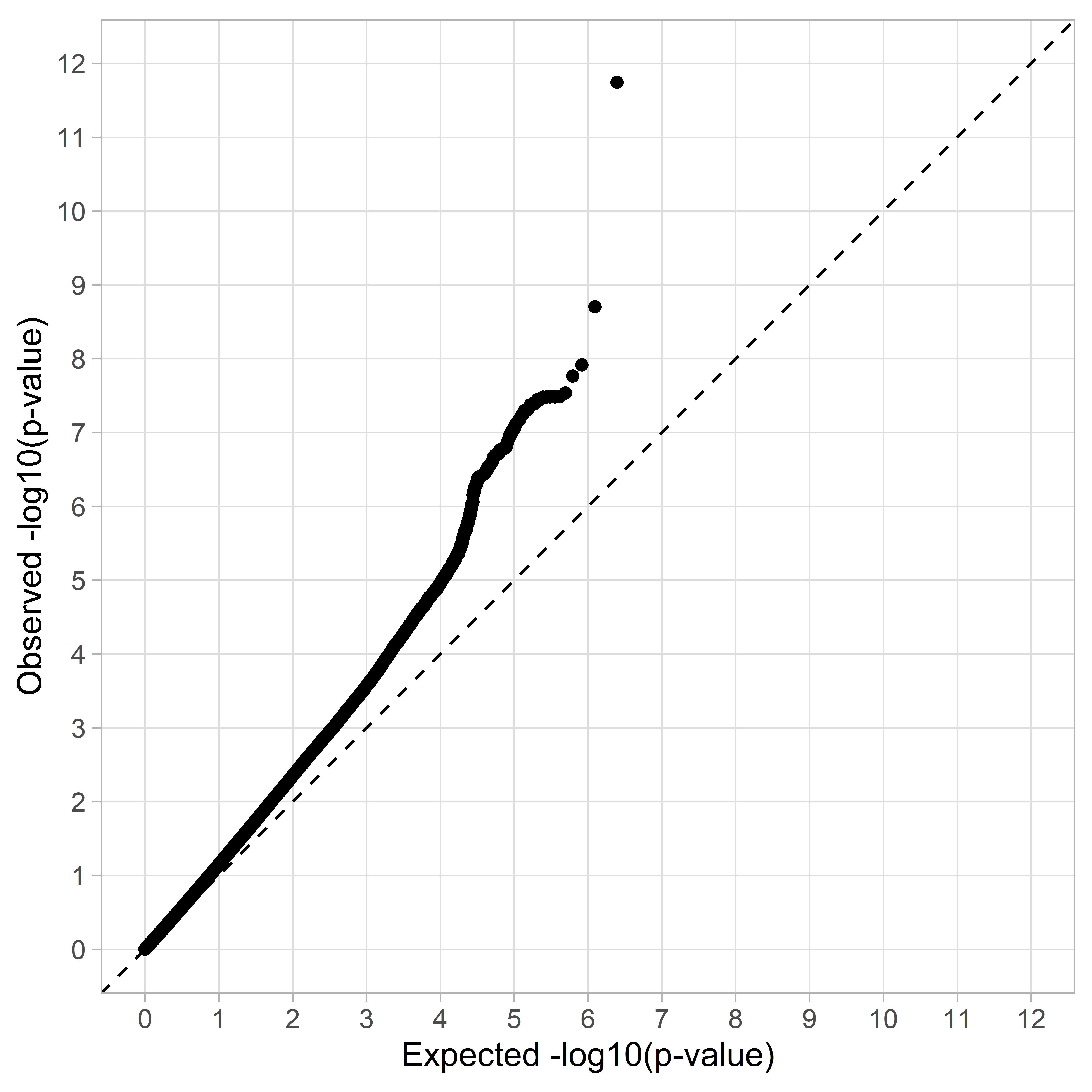


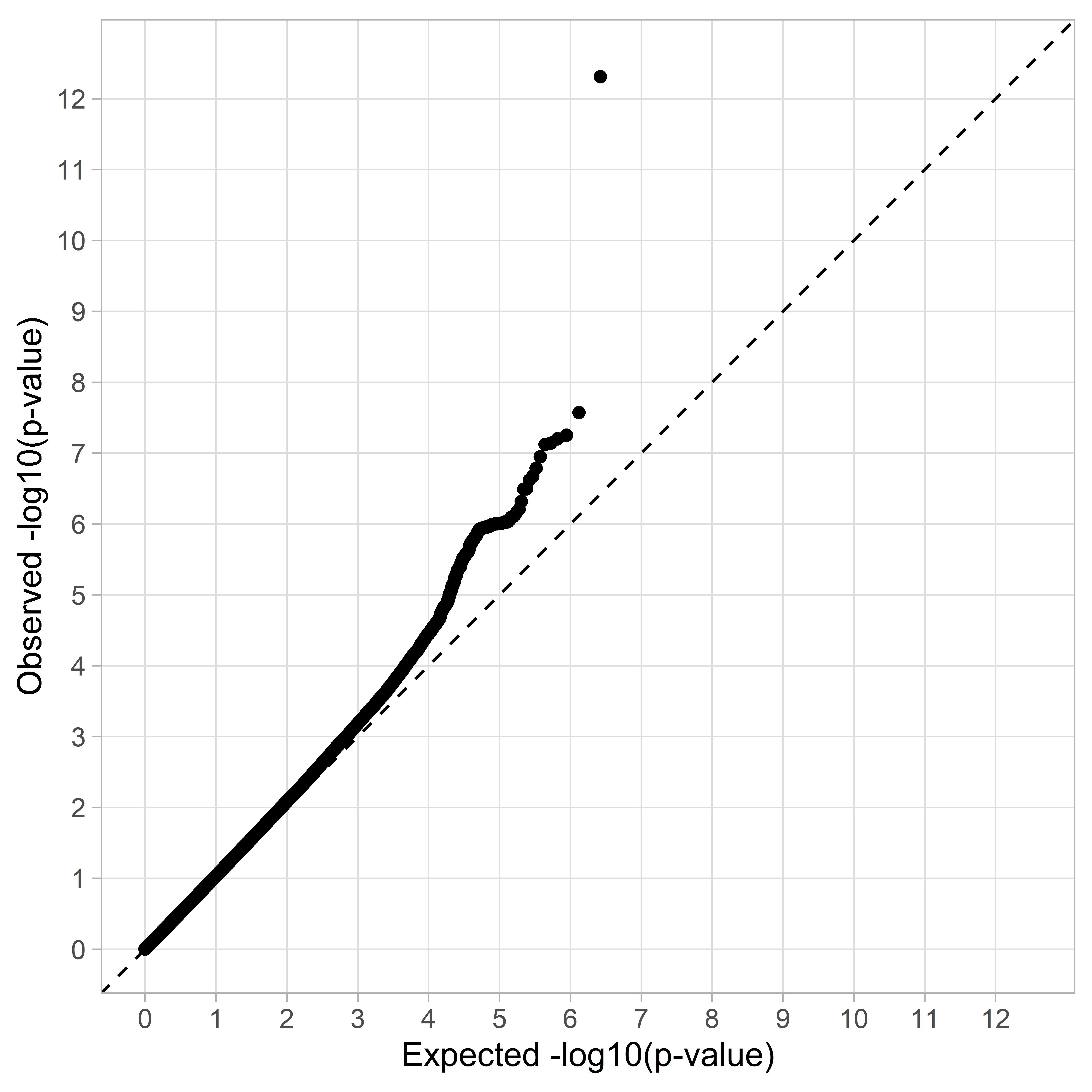


**Supplementary Figure 22:** QQ plots of Model 1 GWAMA results of discovery cohorts for VSTM (upper) and VL (lower).

**Supplementary Figure 23:** QQ plots of discovery cohort GWAMA (Model 1) for VSTM cohorts with paragraph recall test data (upper left panel) and those with word list recall test data (upper right panel), VL cohorts with orally presented words (lower left panel) and those with visually presented words (lower right panel).


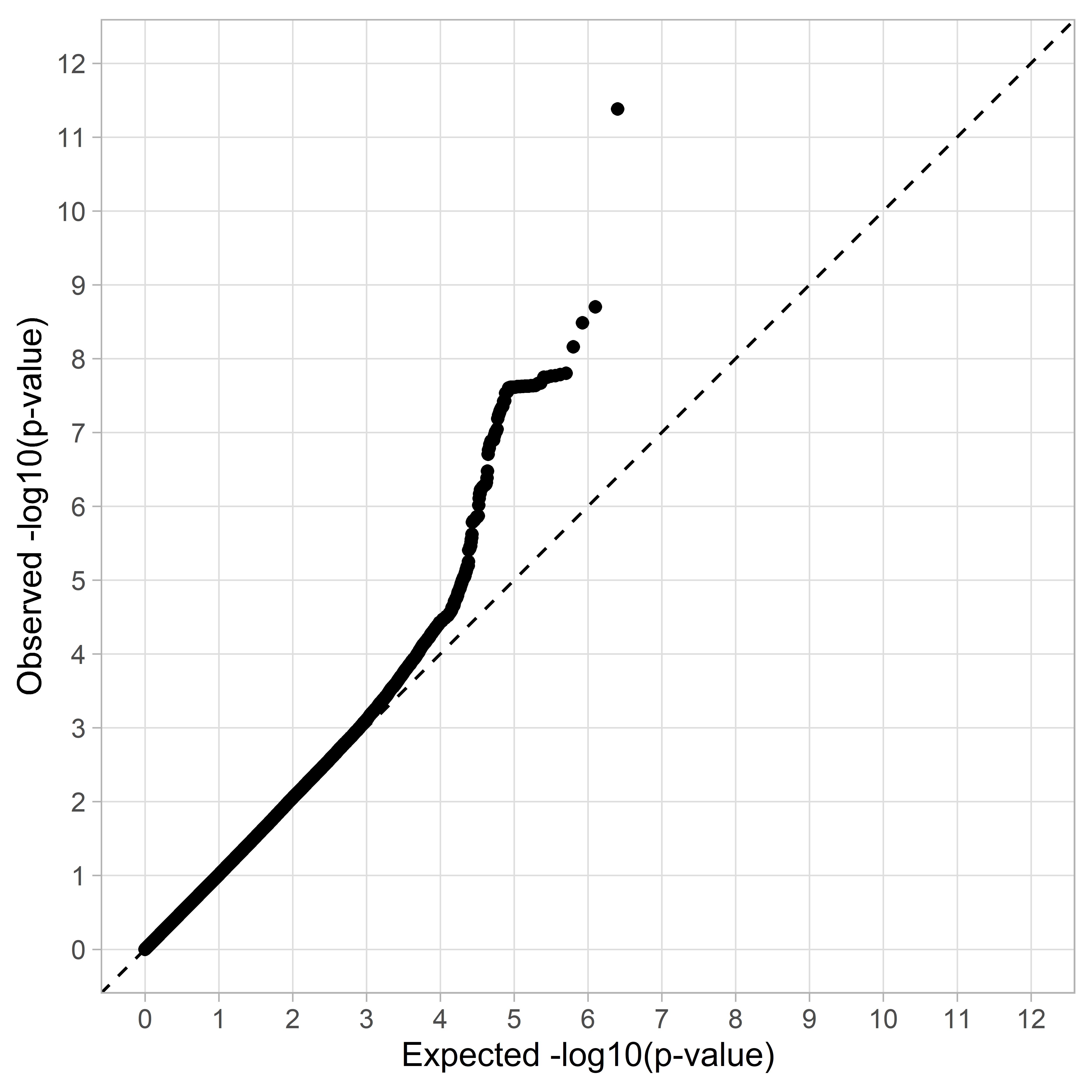


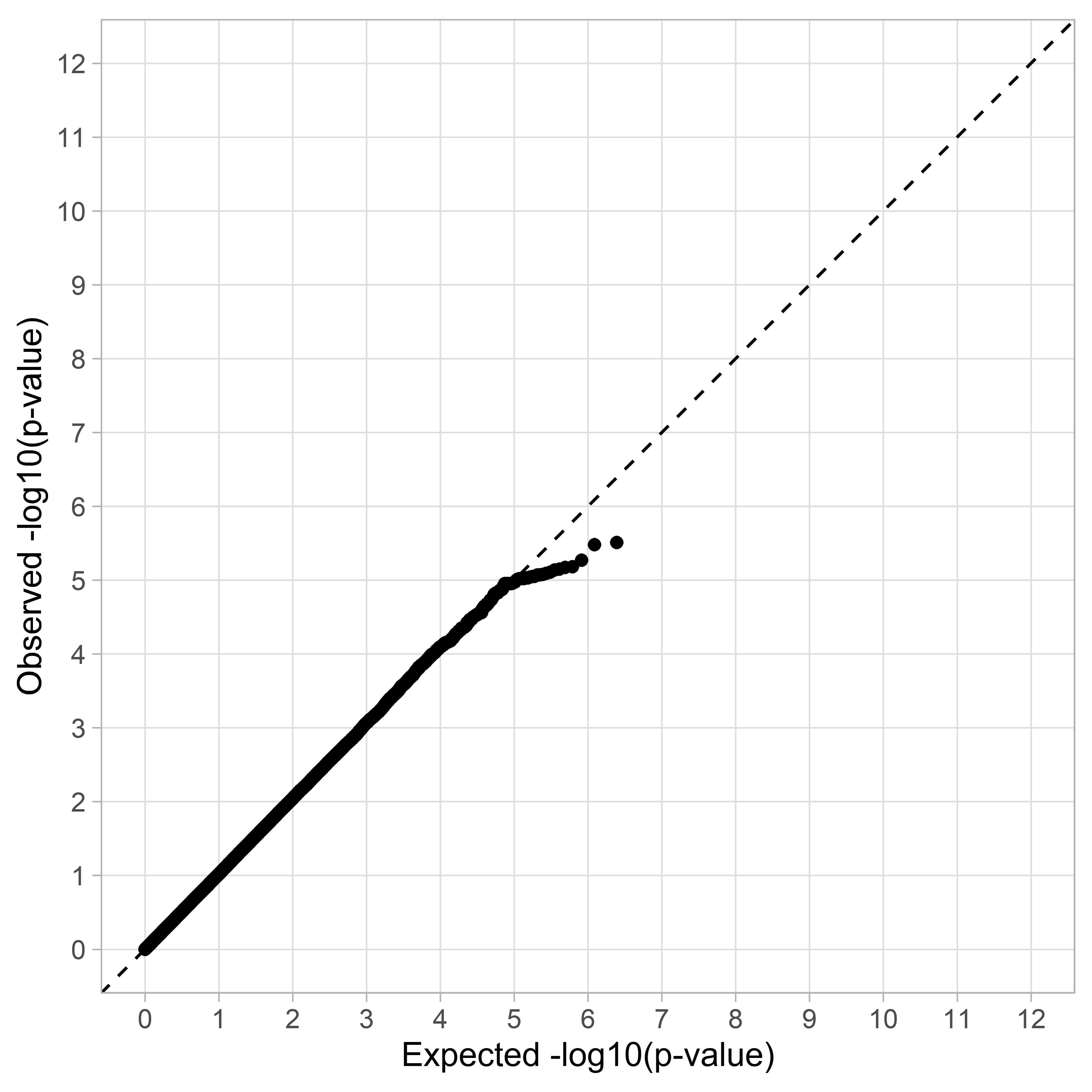


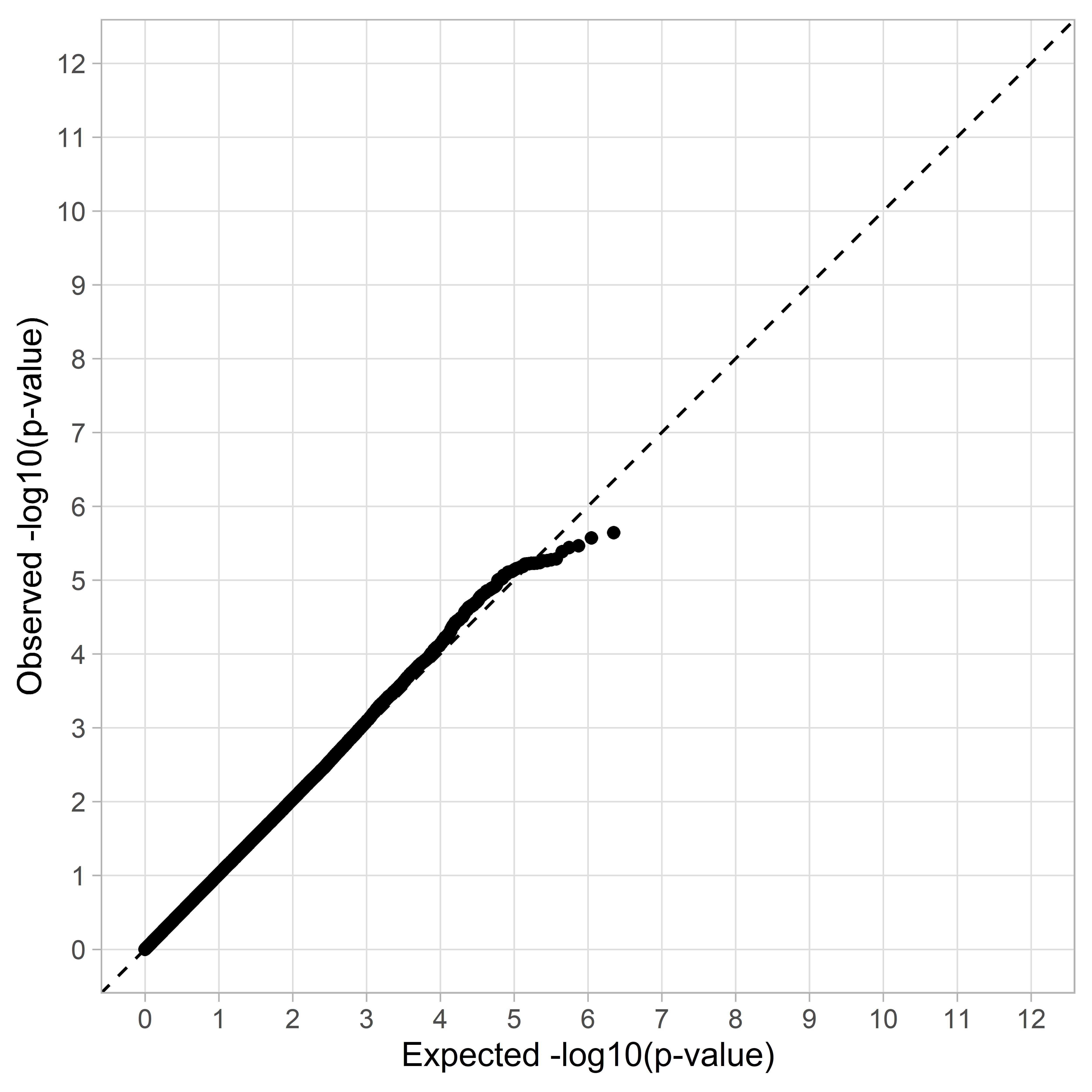


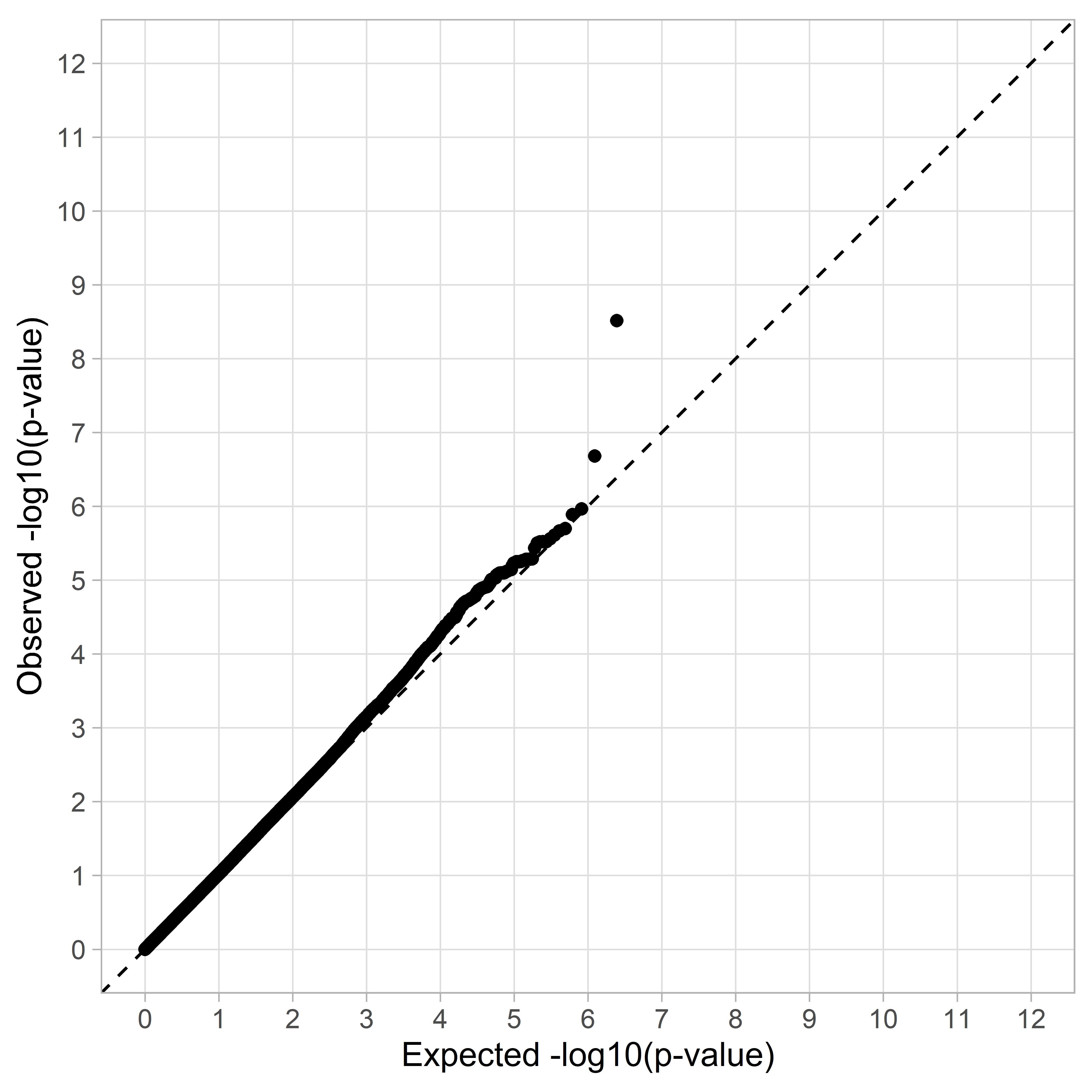


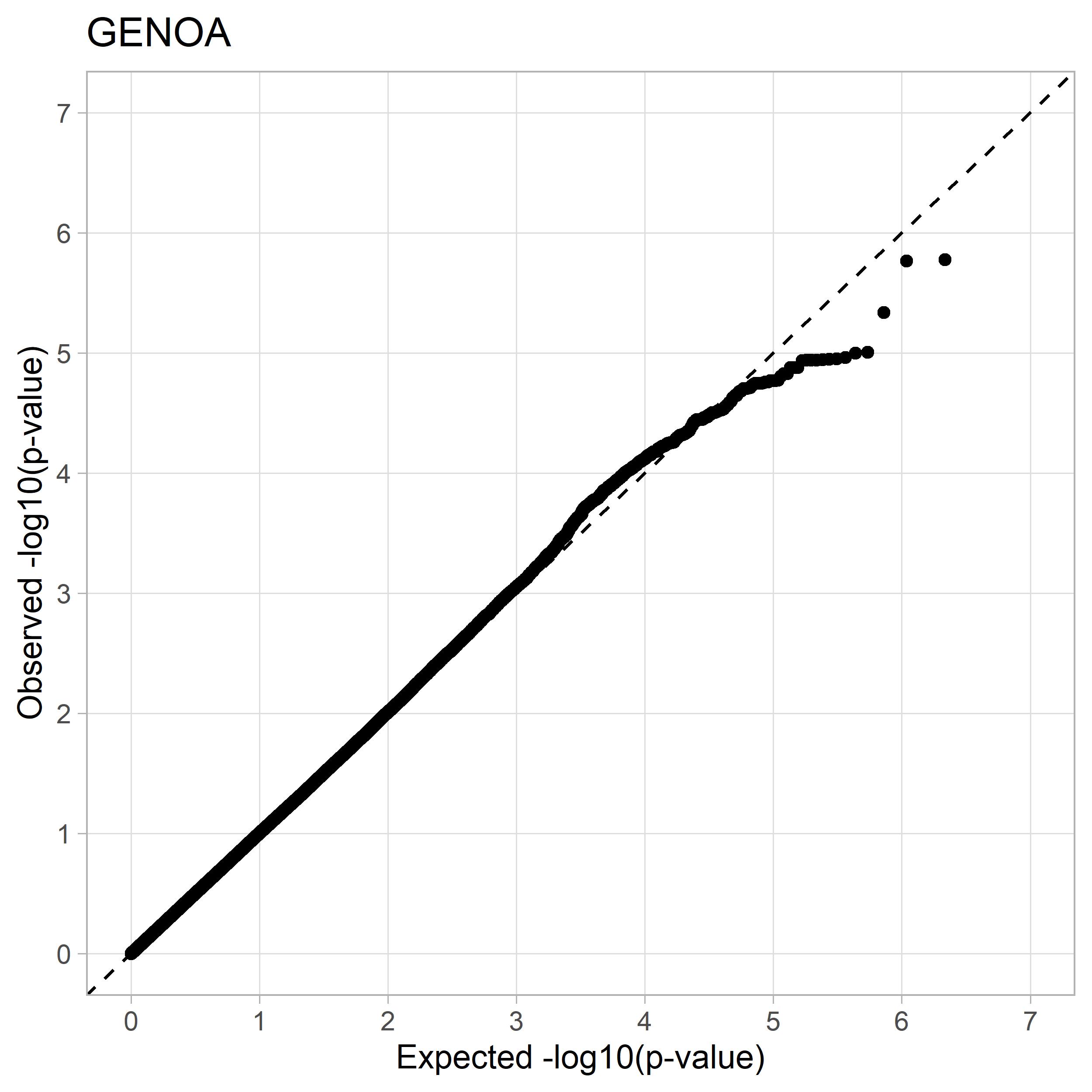


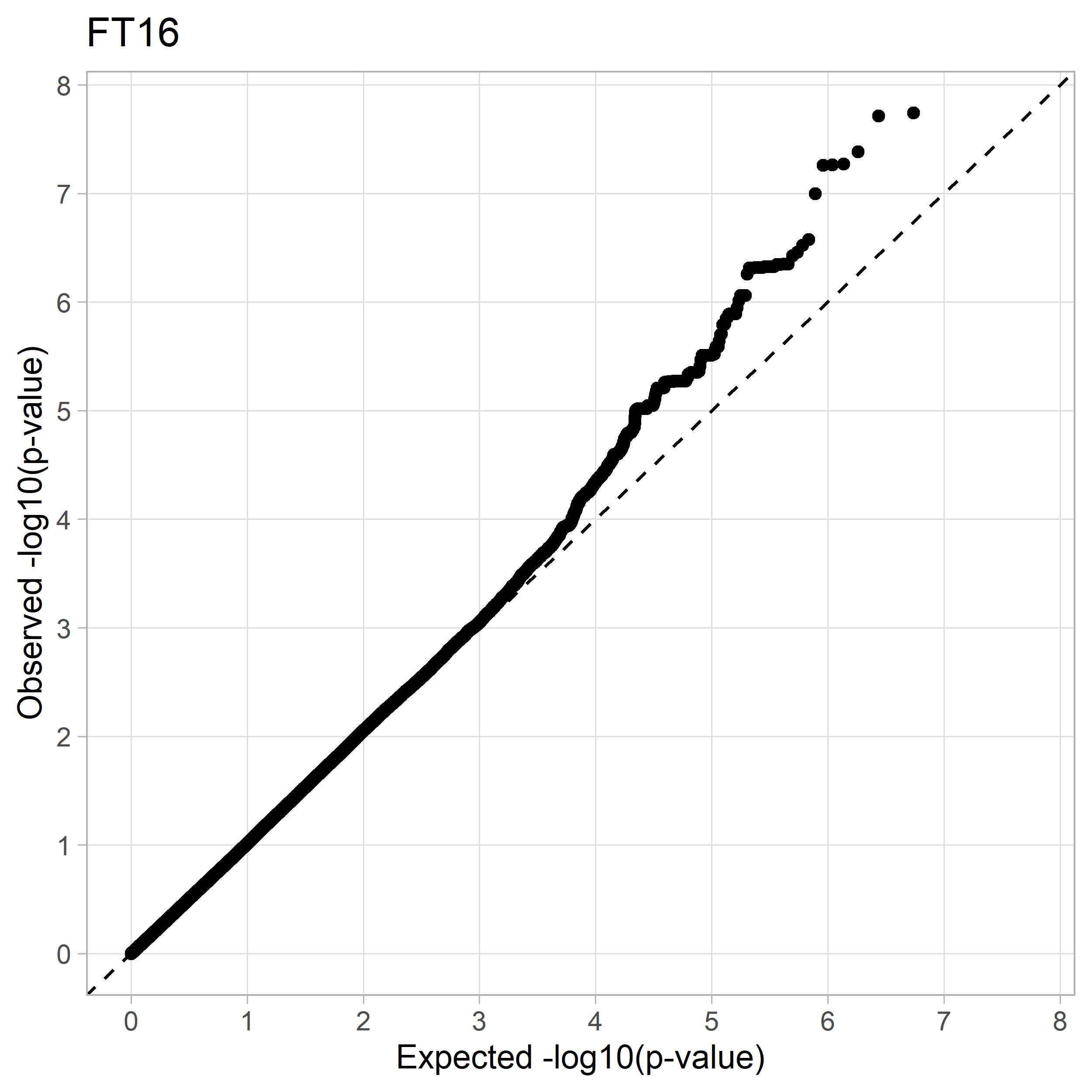


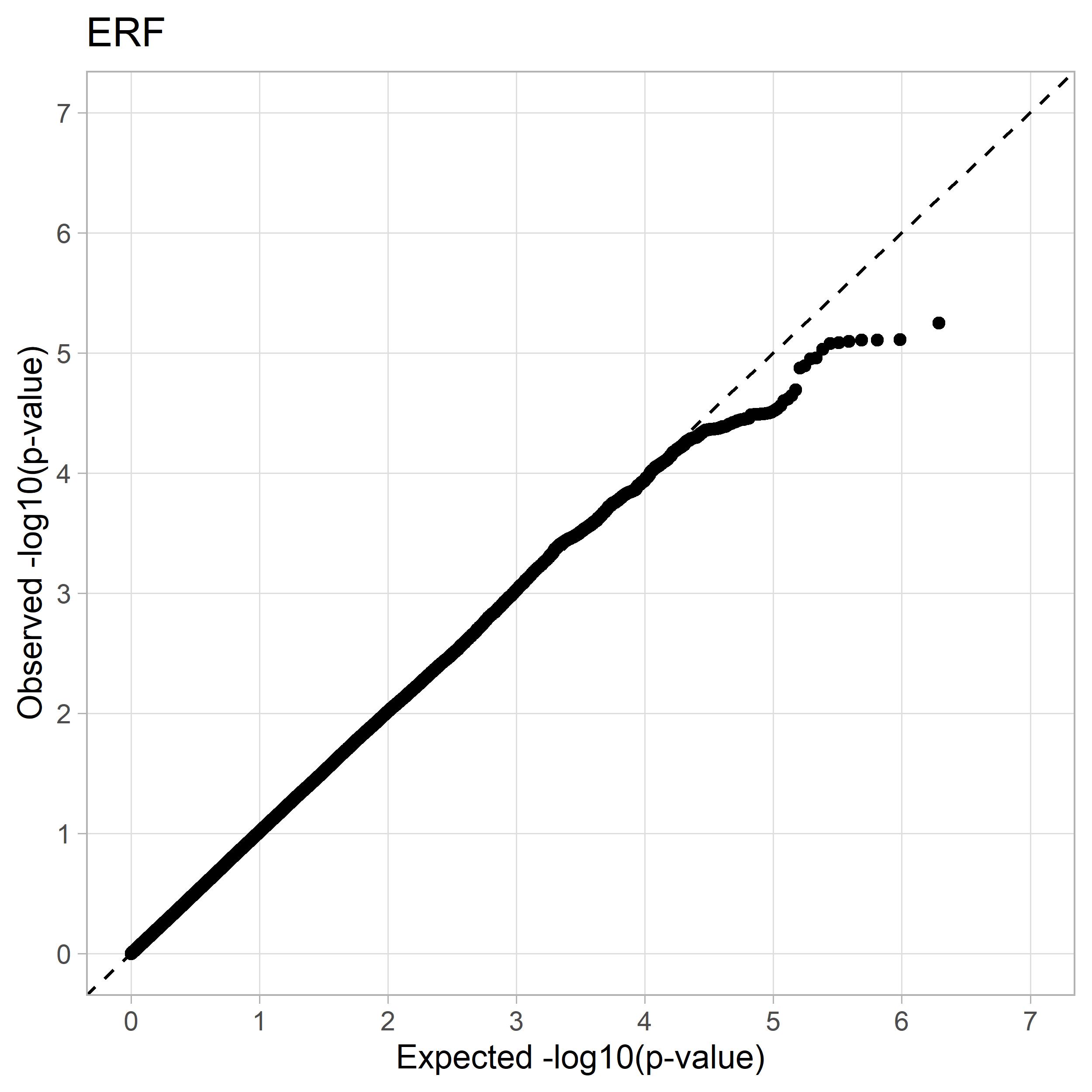


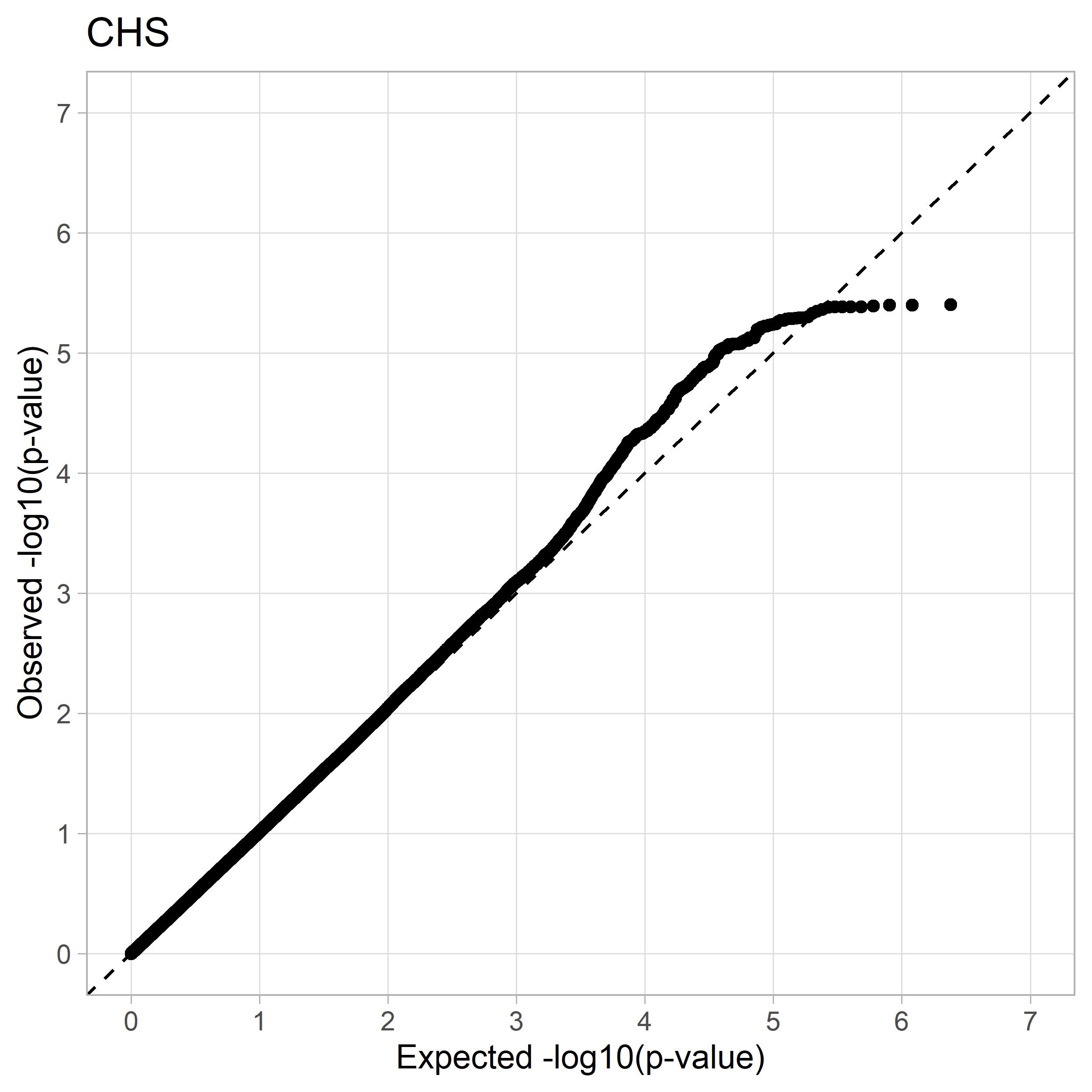


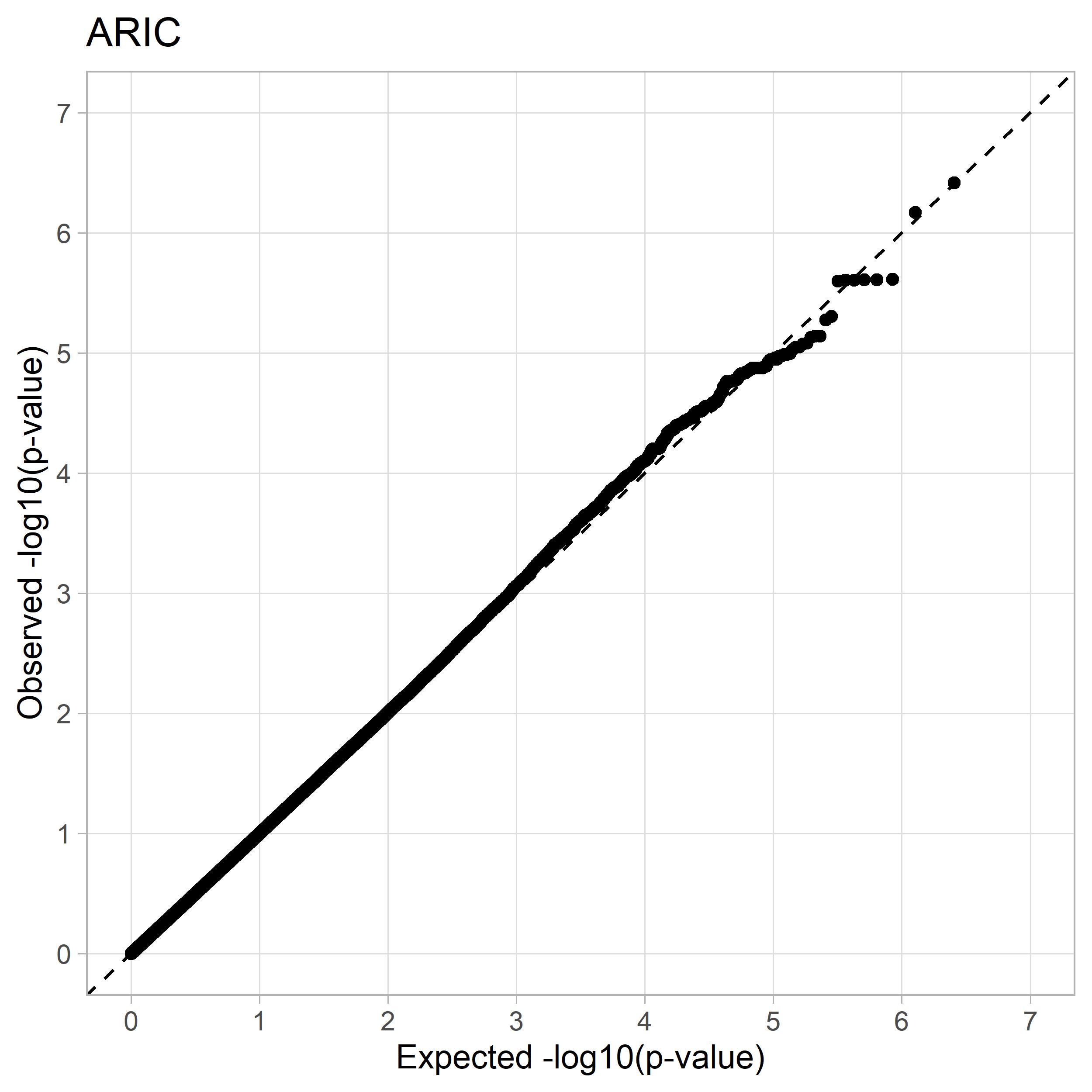


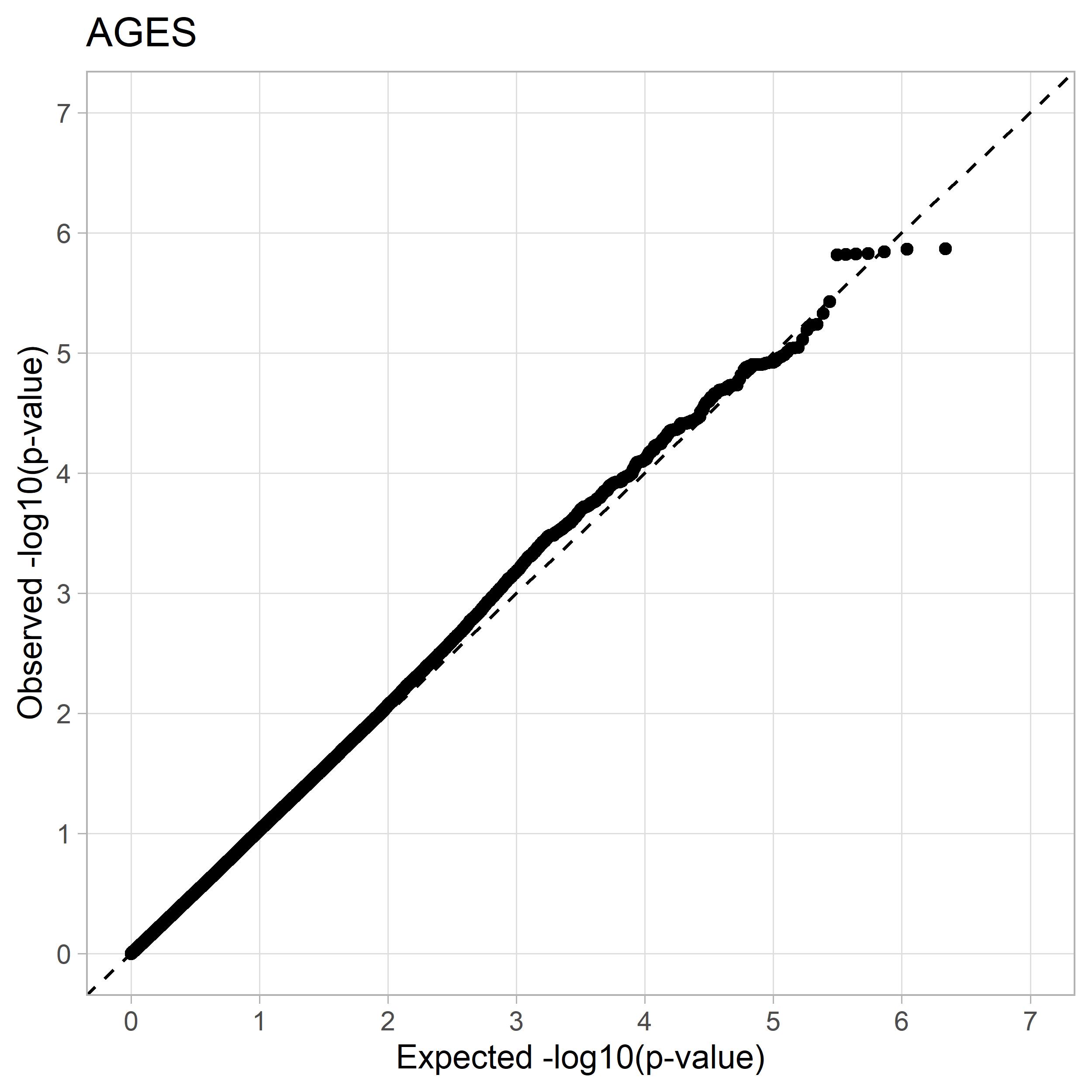


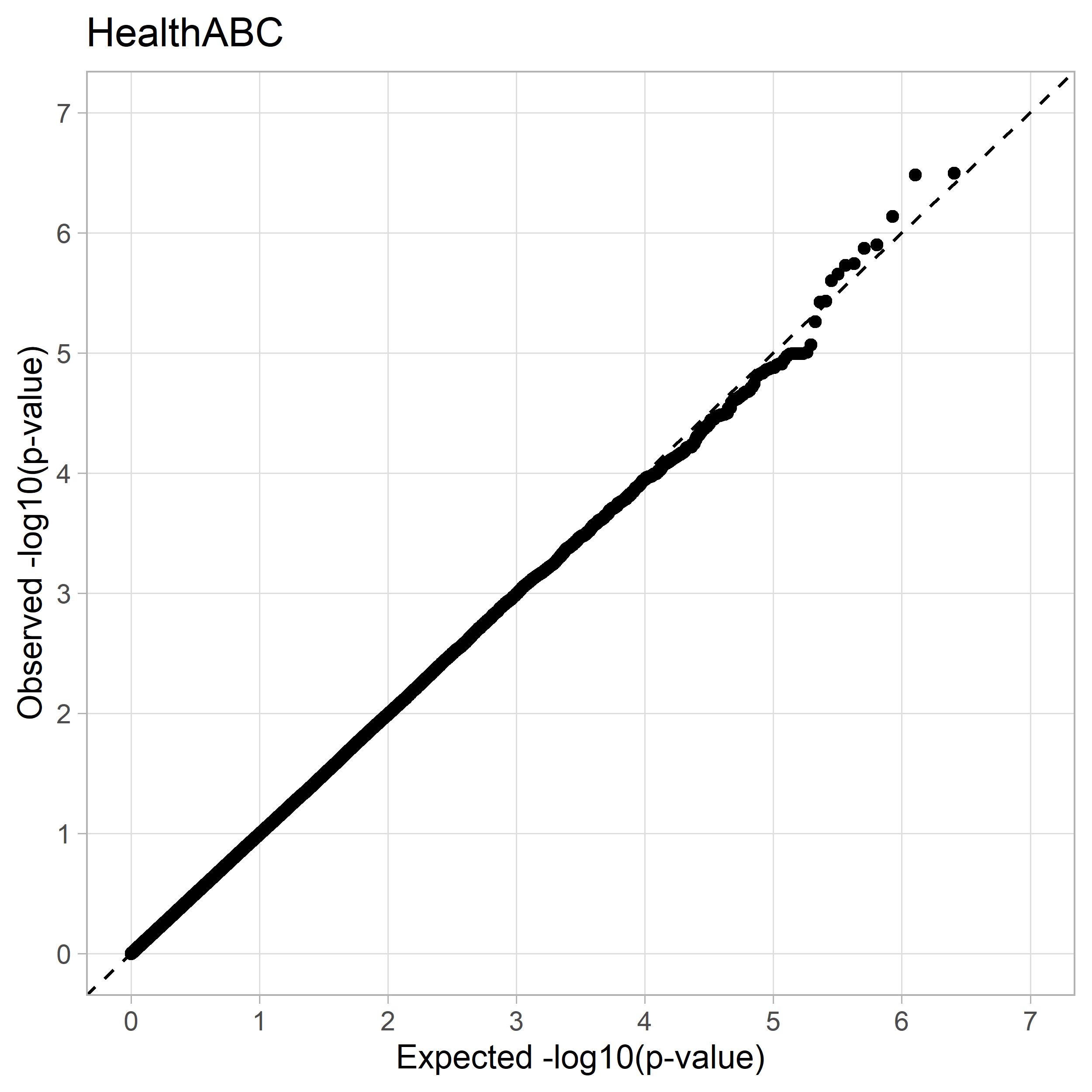


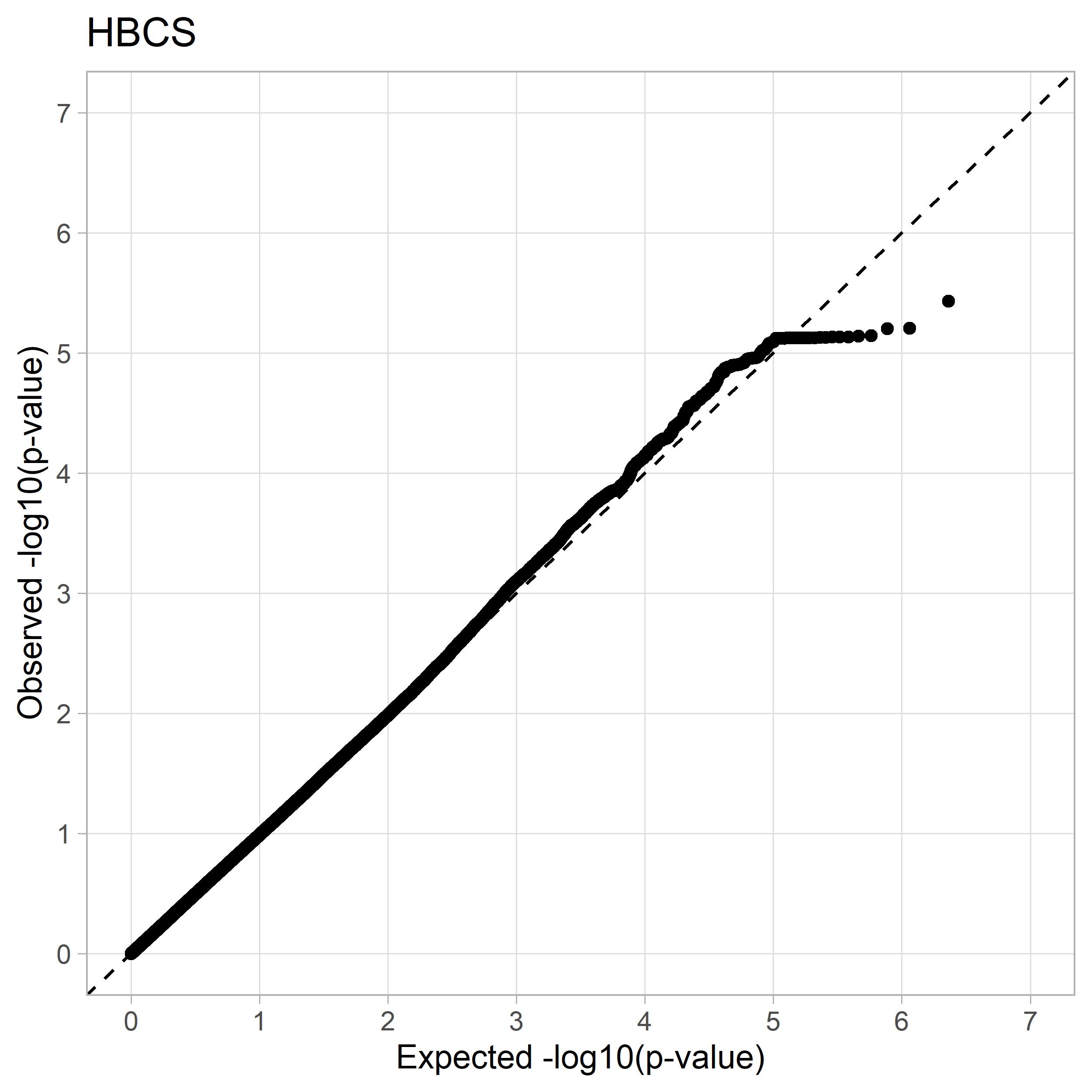


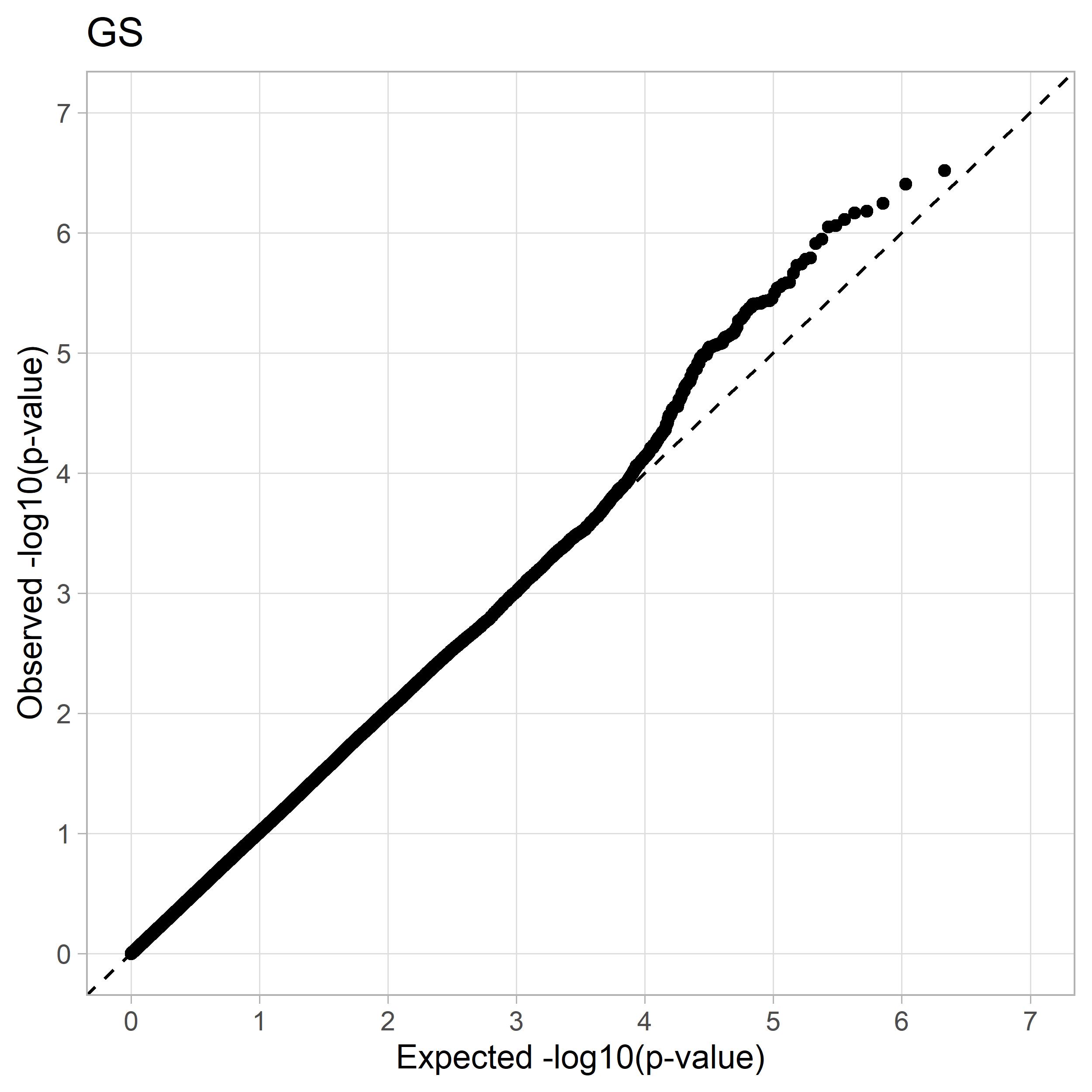


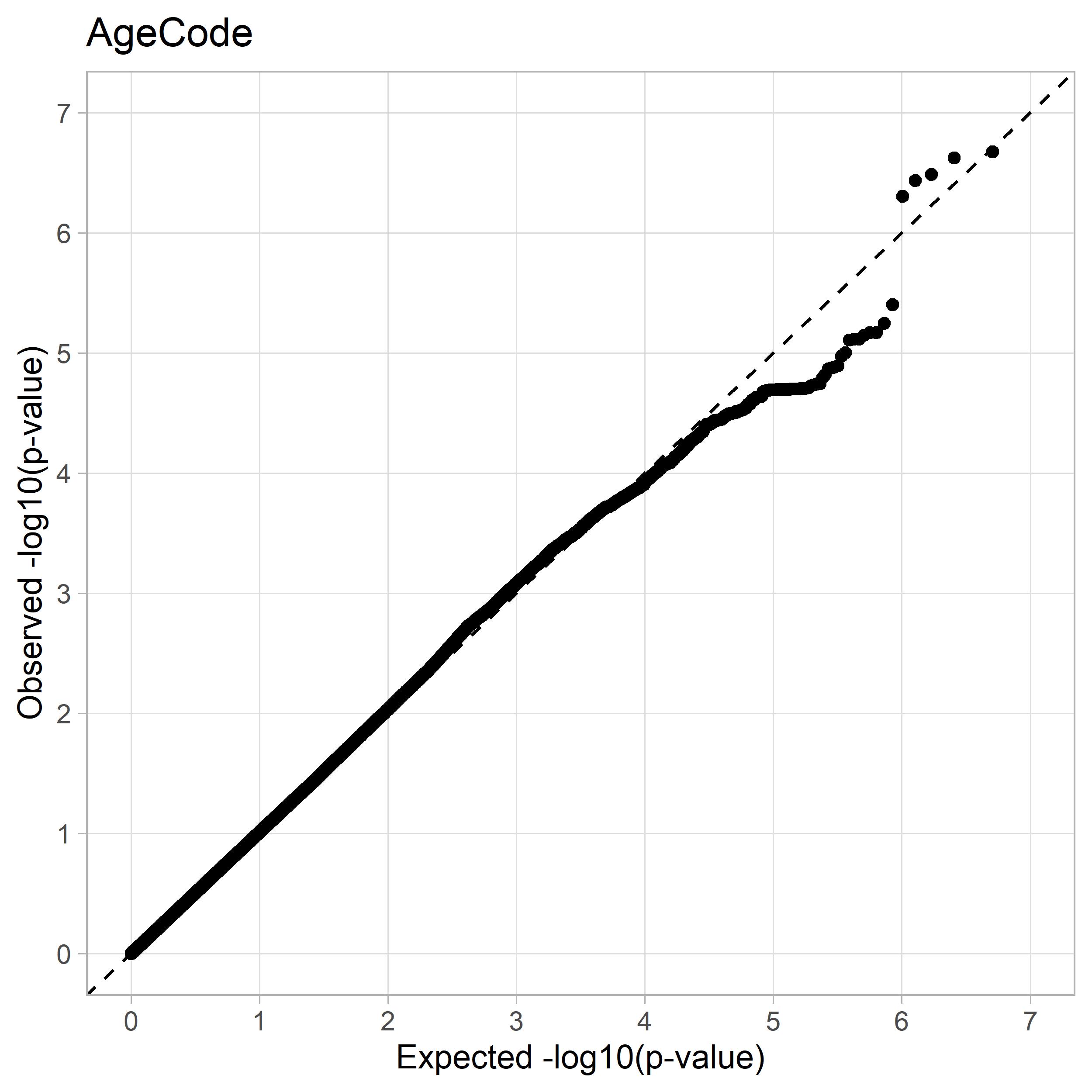


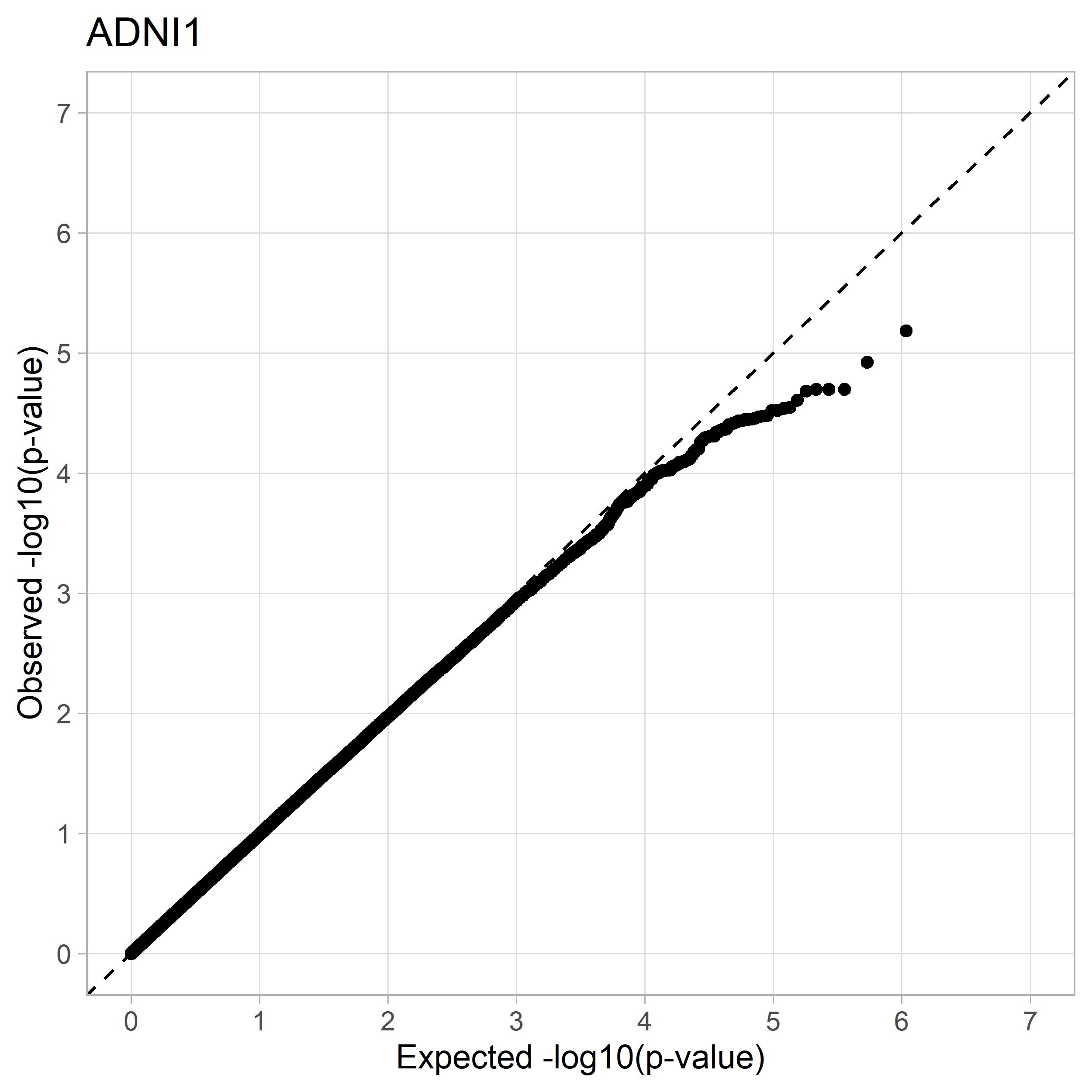


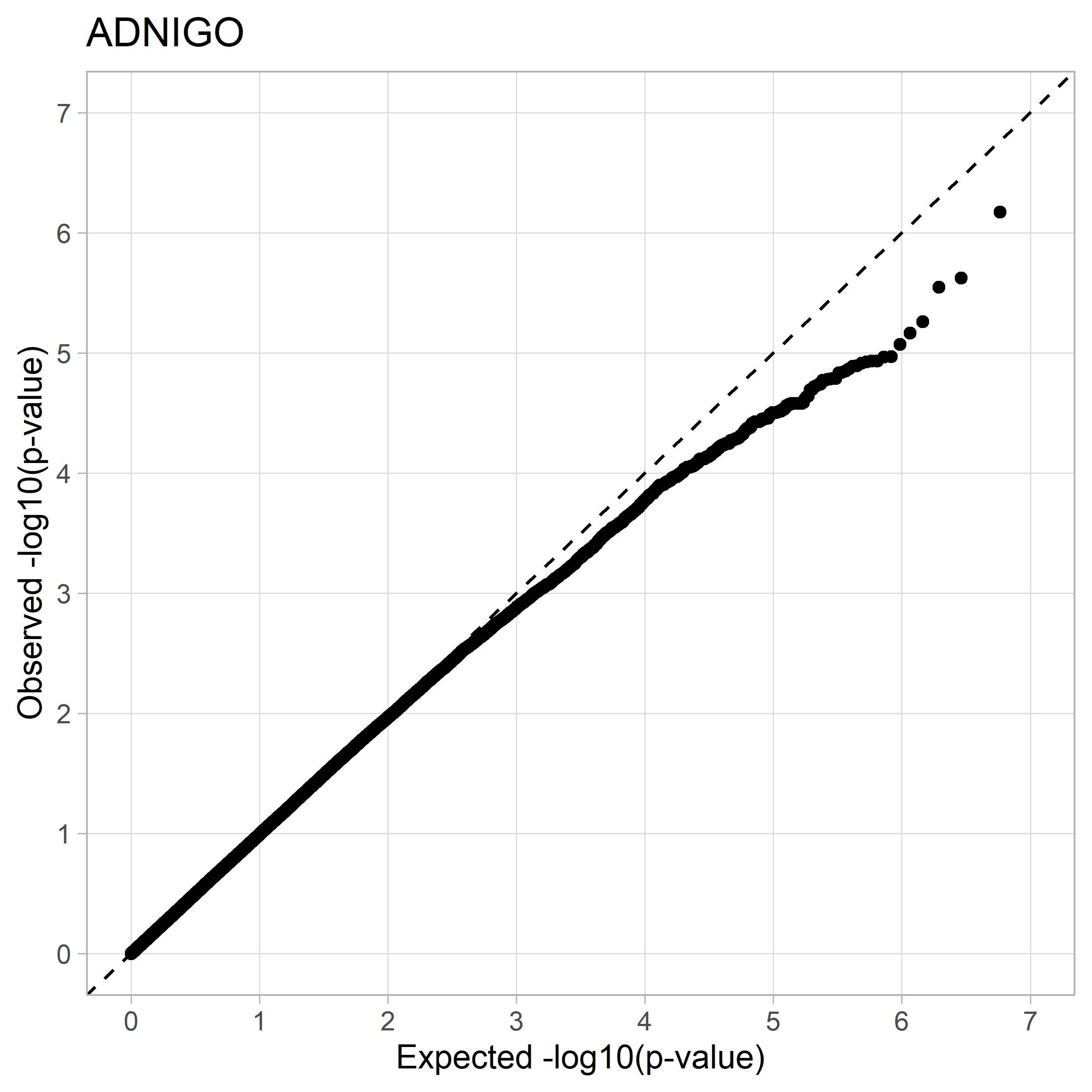


**Supplementary Figure 24:** QQ plots of cohort-level VSTM GWAS (Model 1) results (ADNI1 to Health ABC).


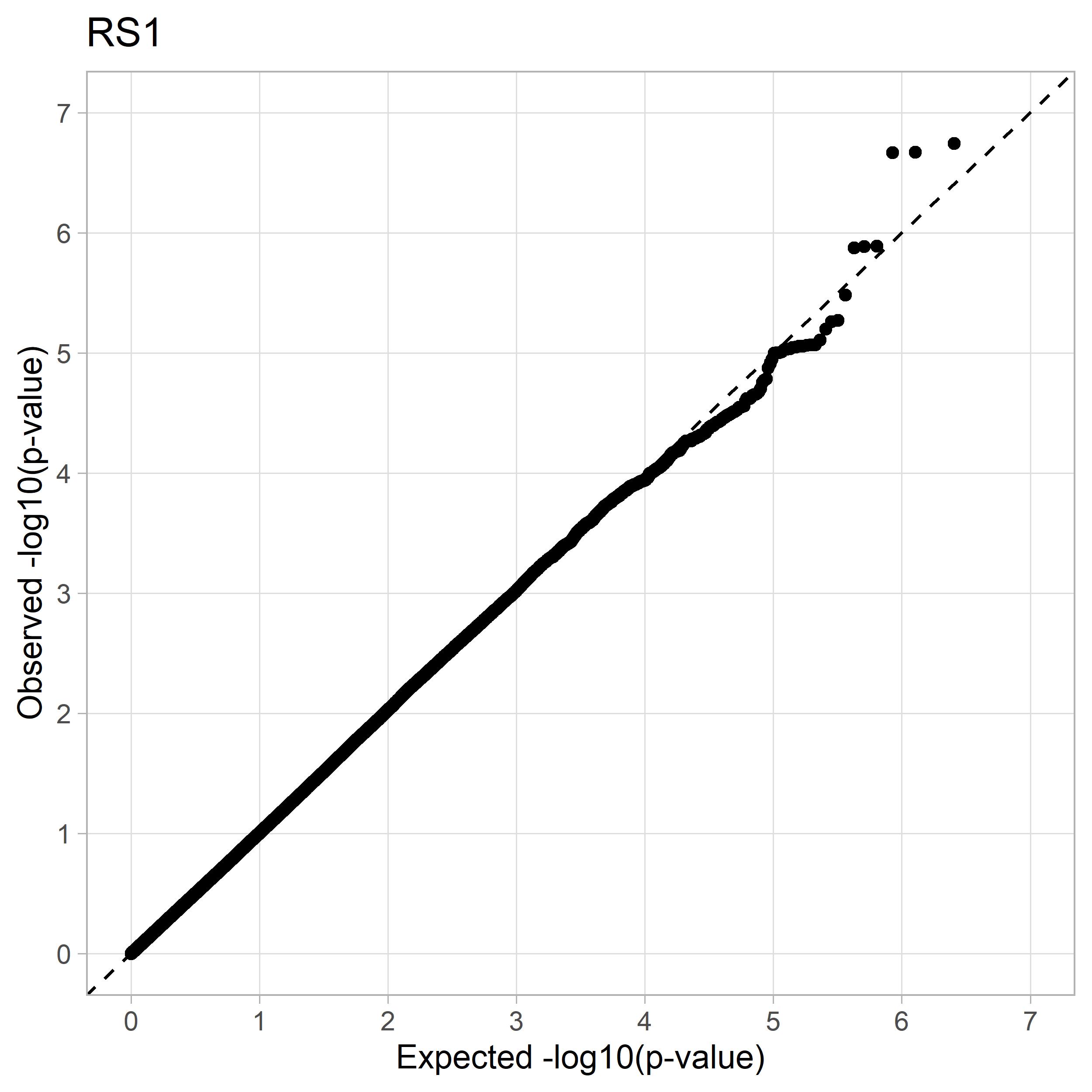


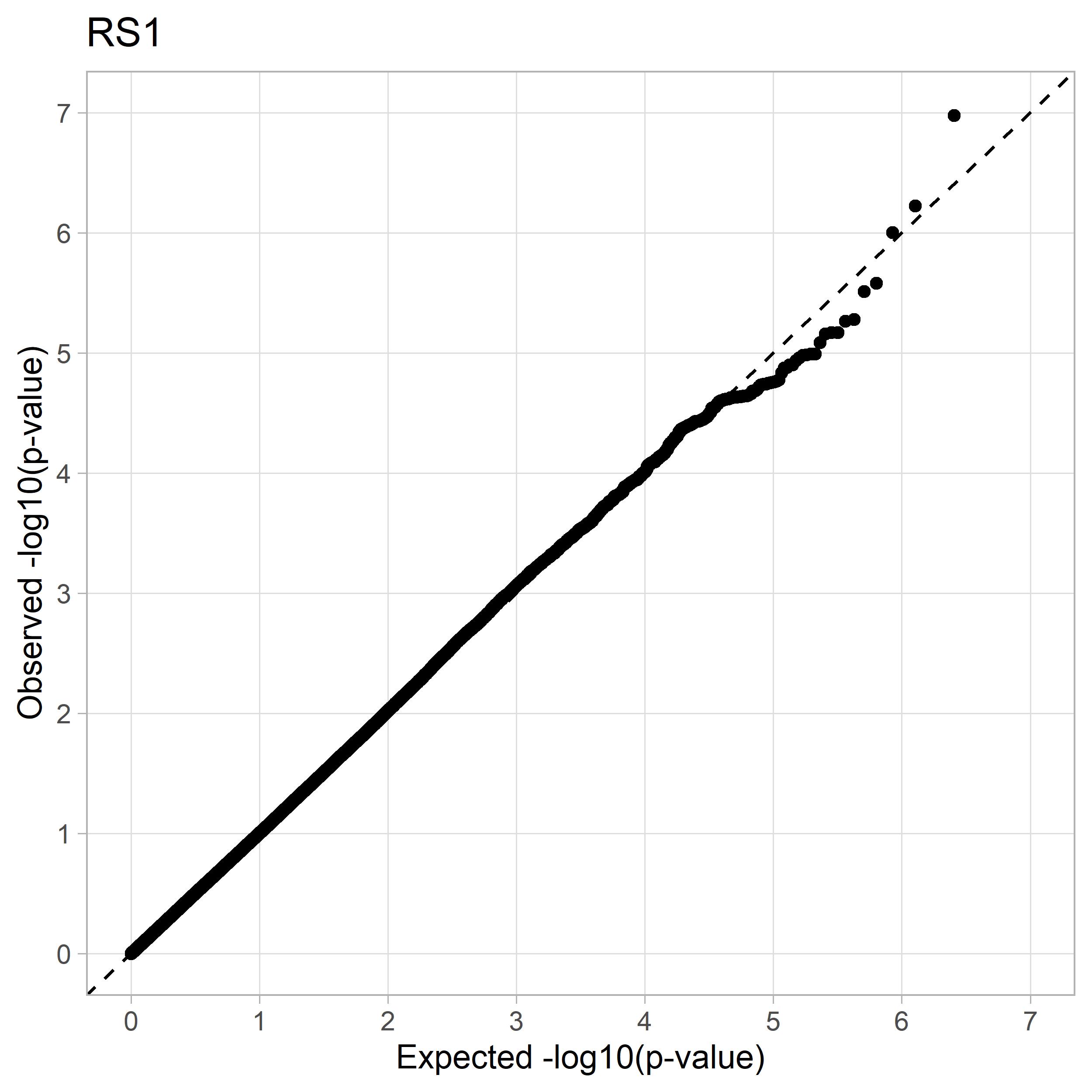


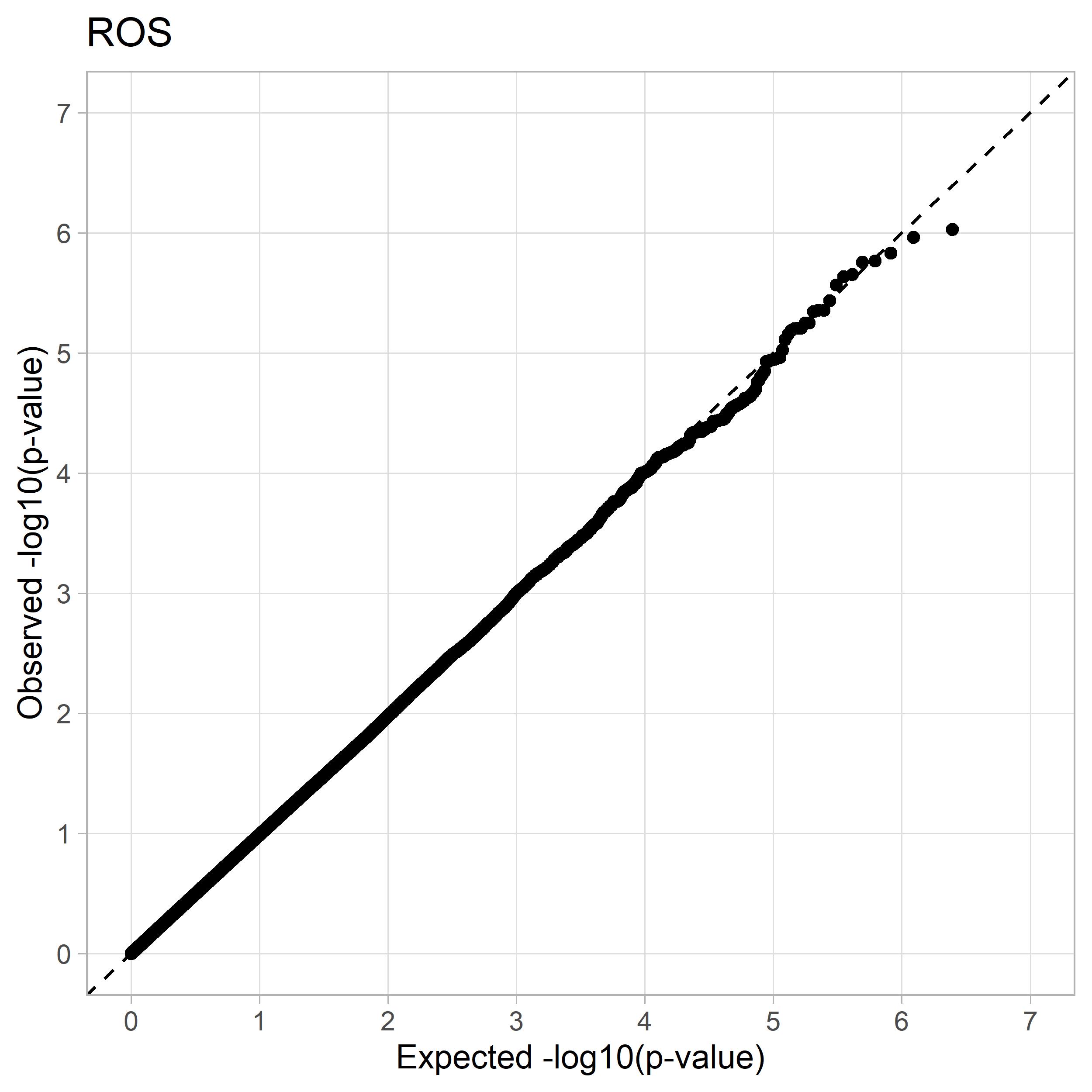


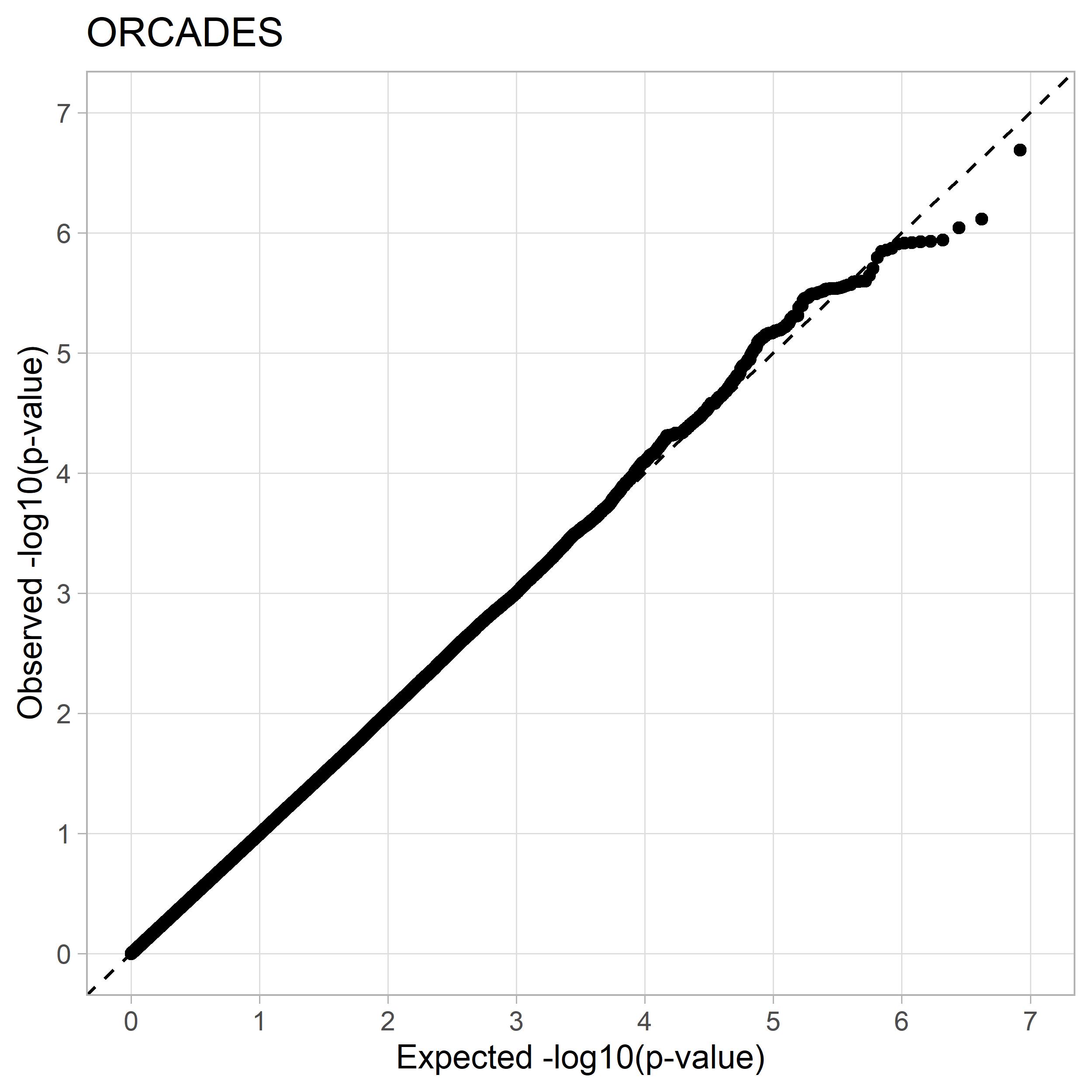


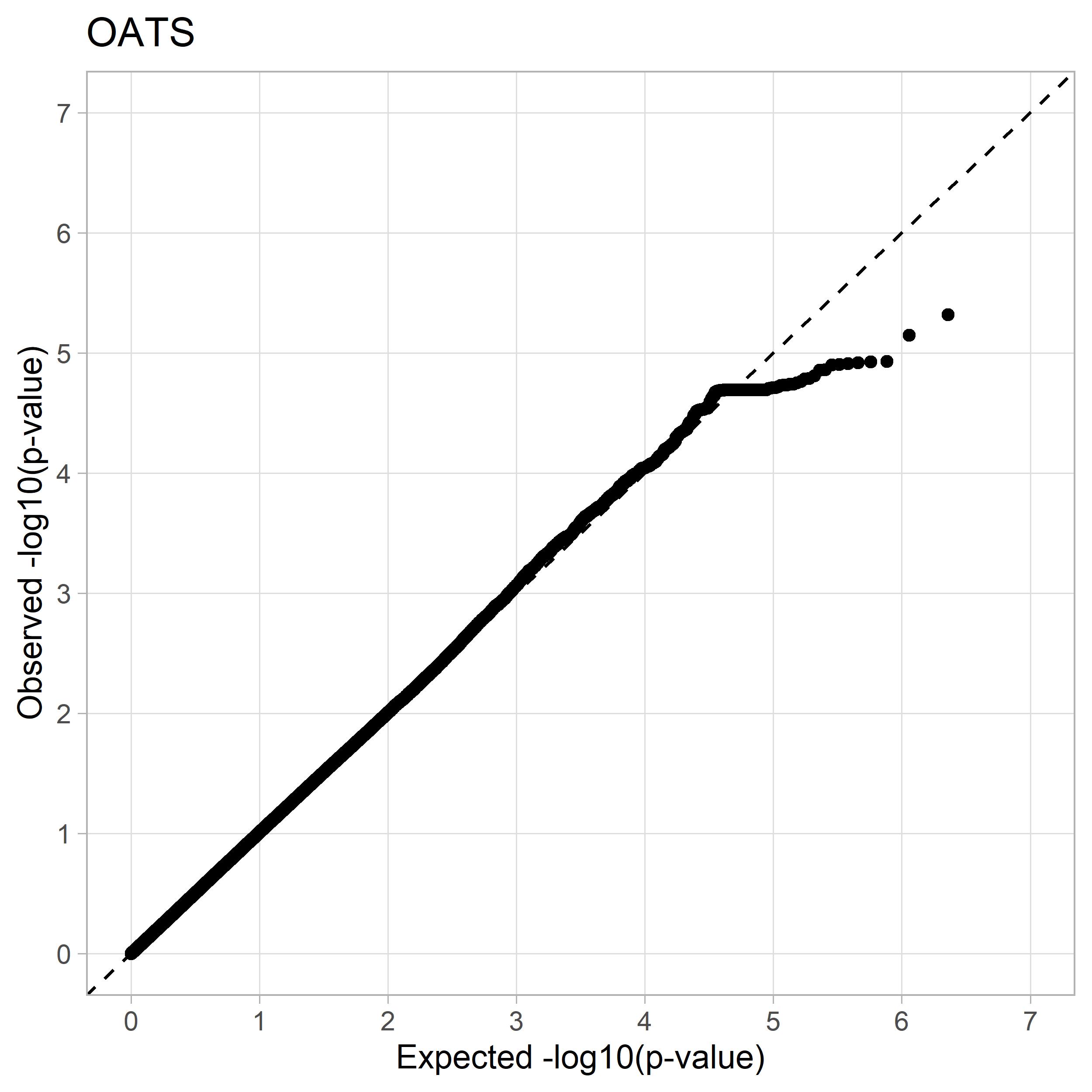


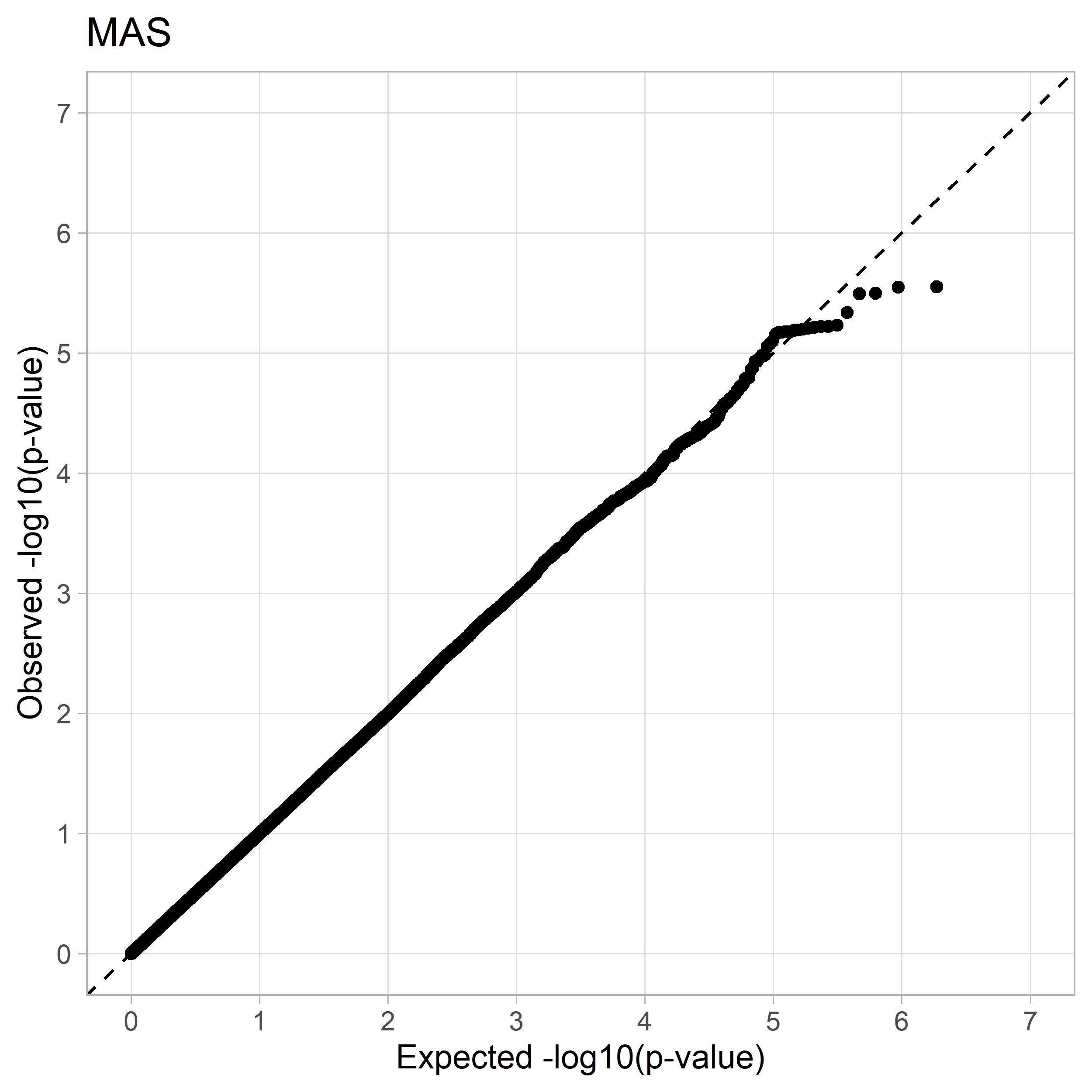


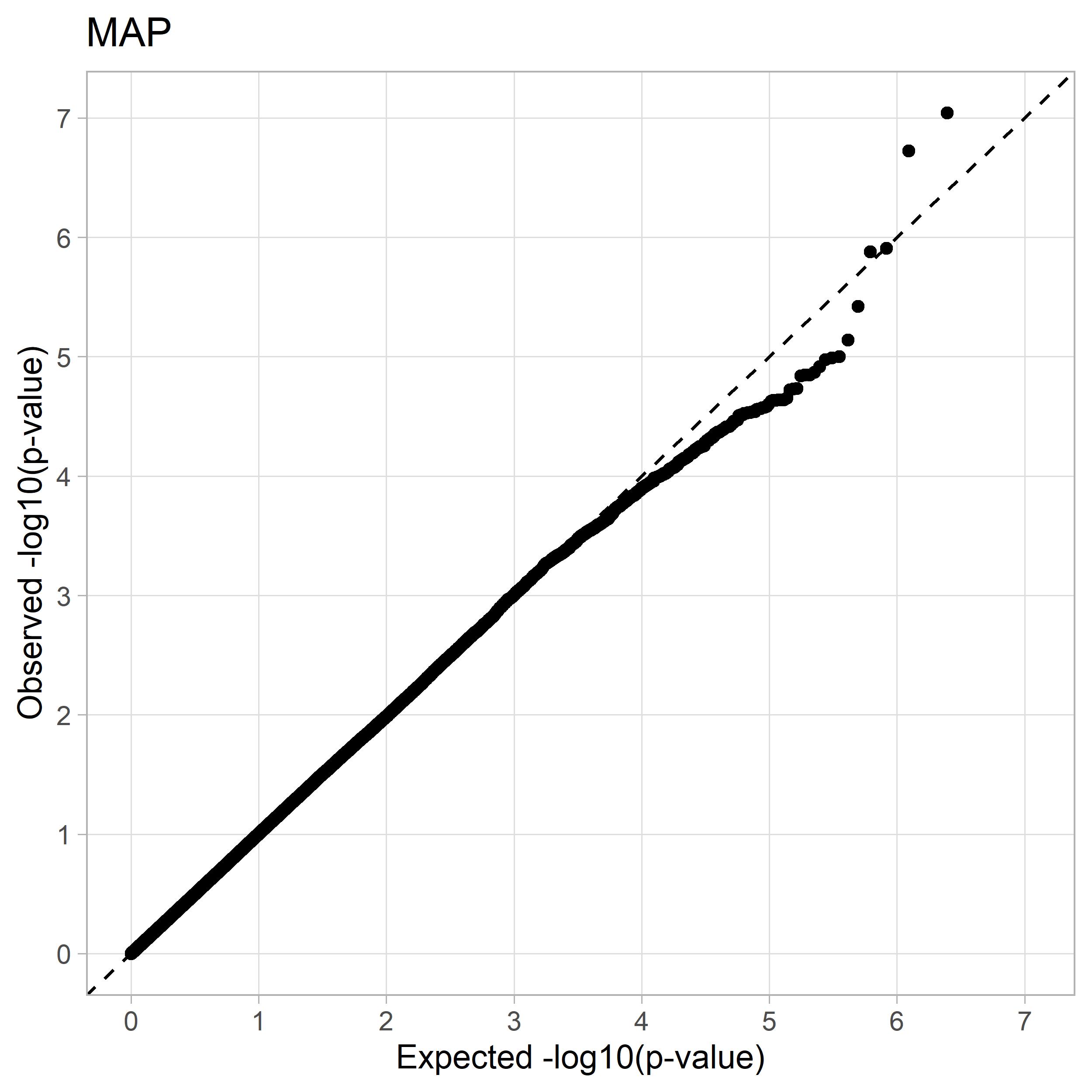


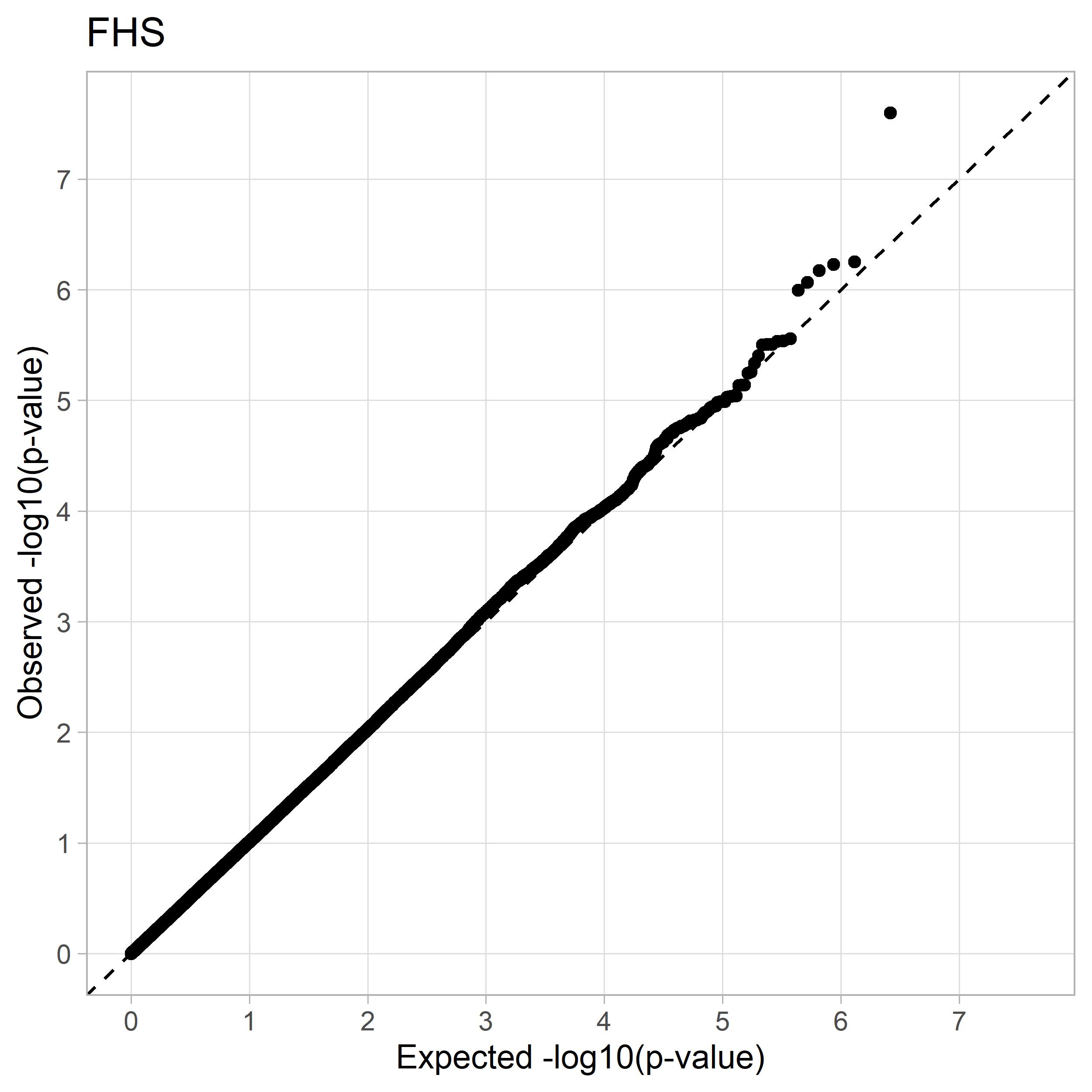


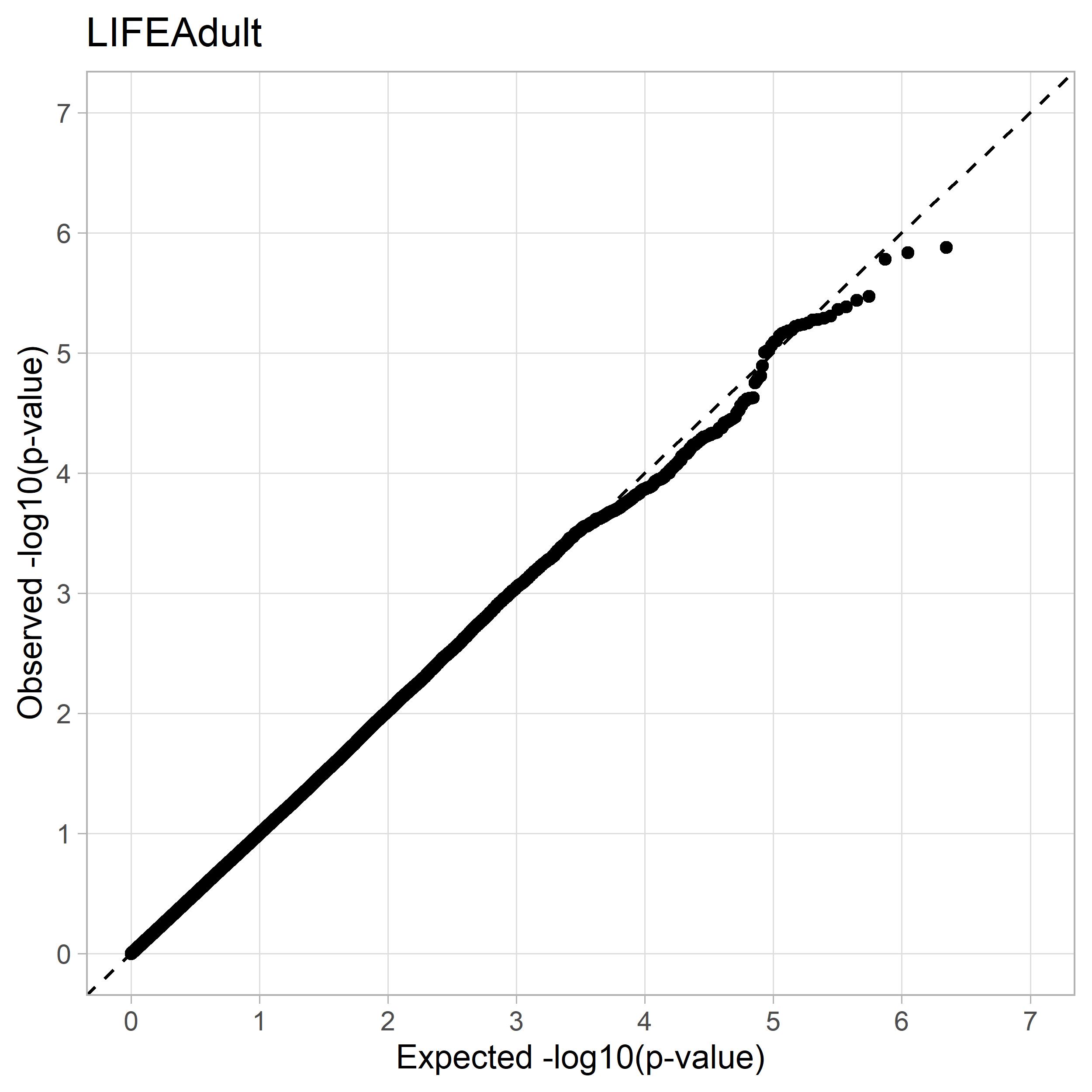


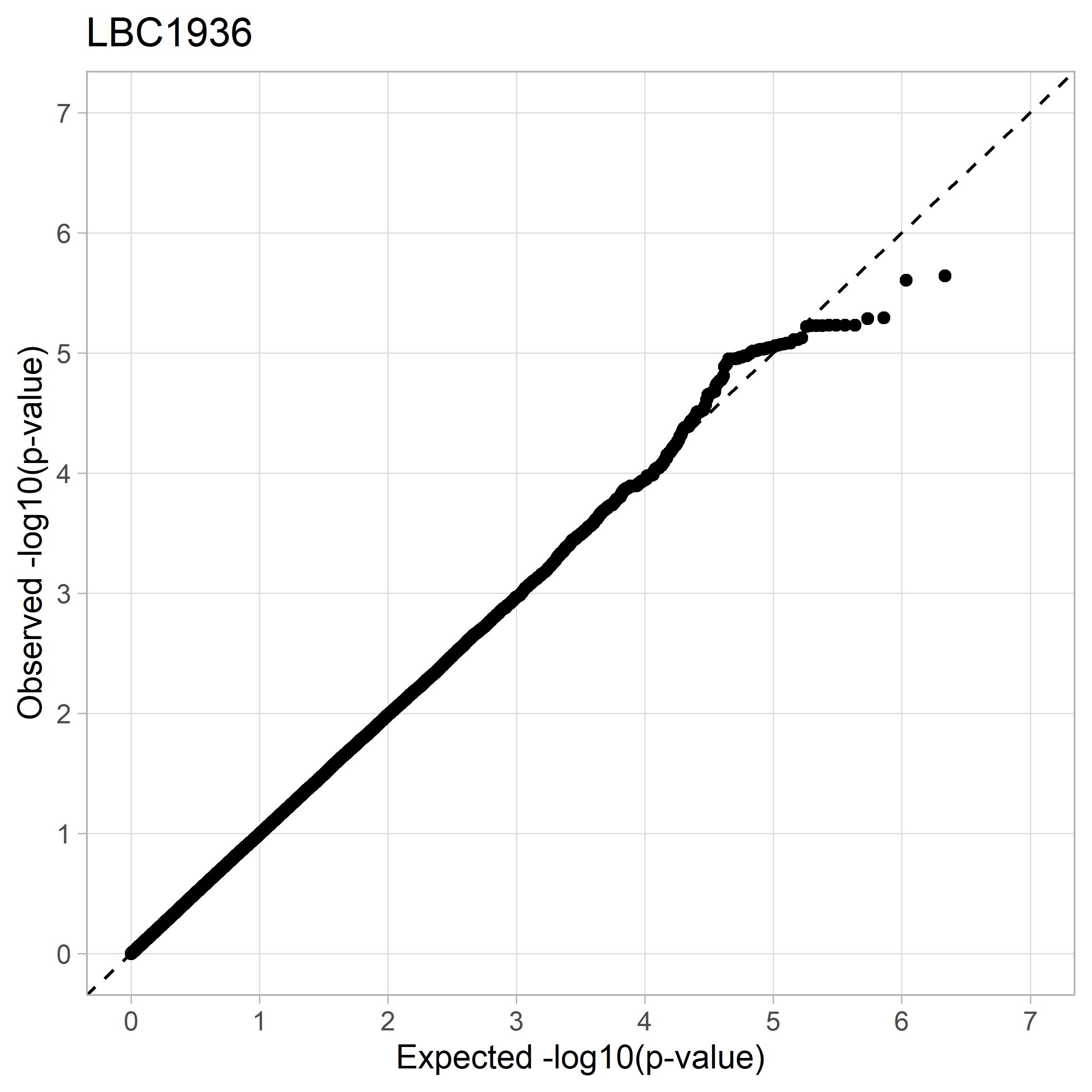

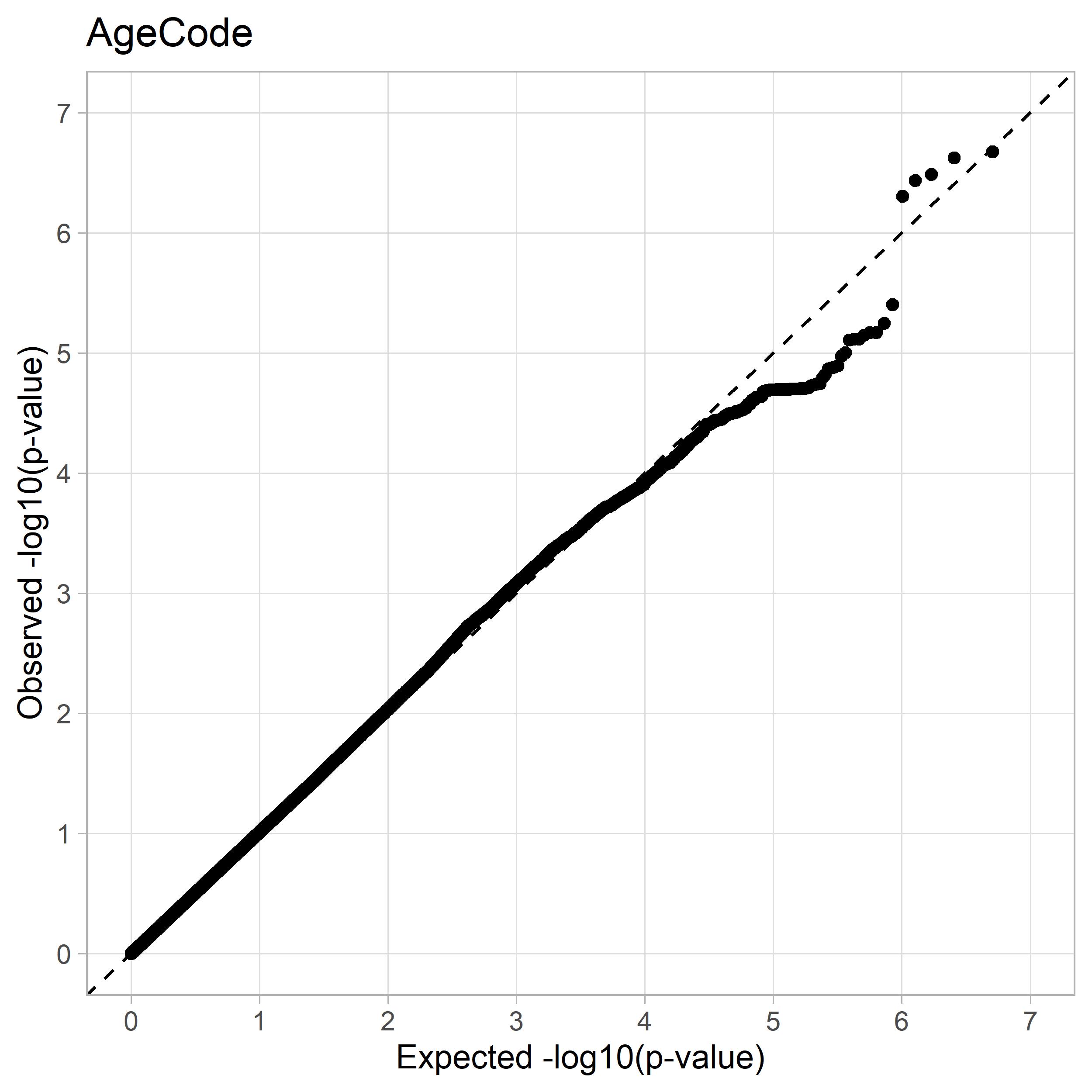


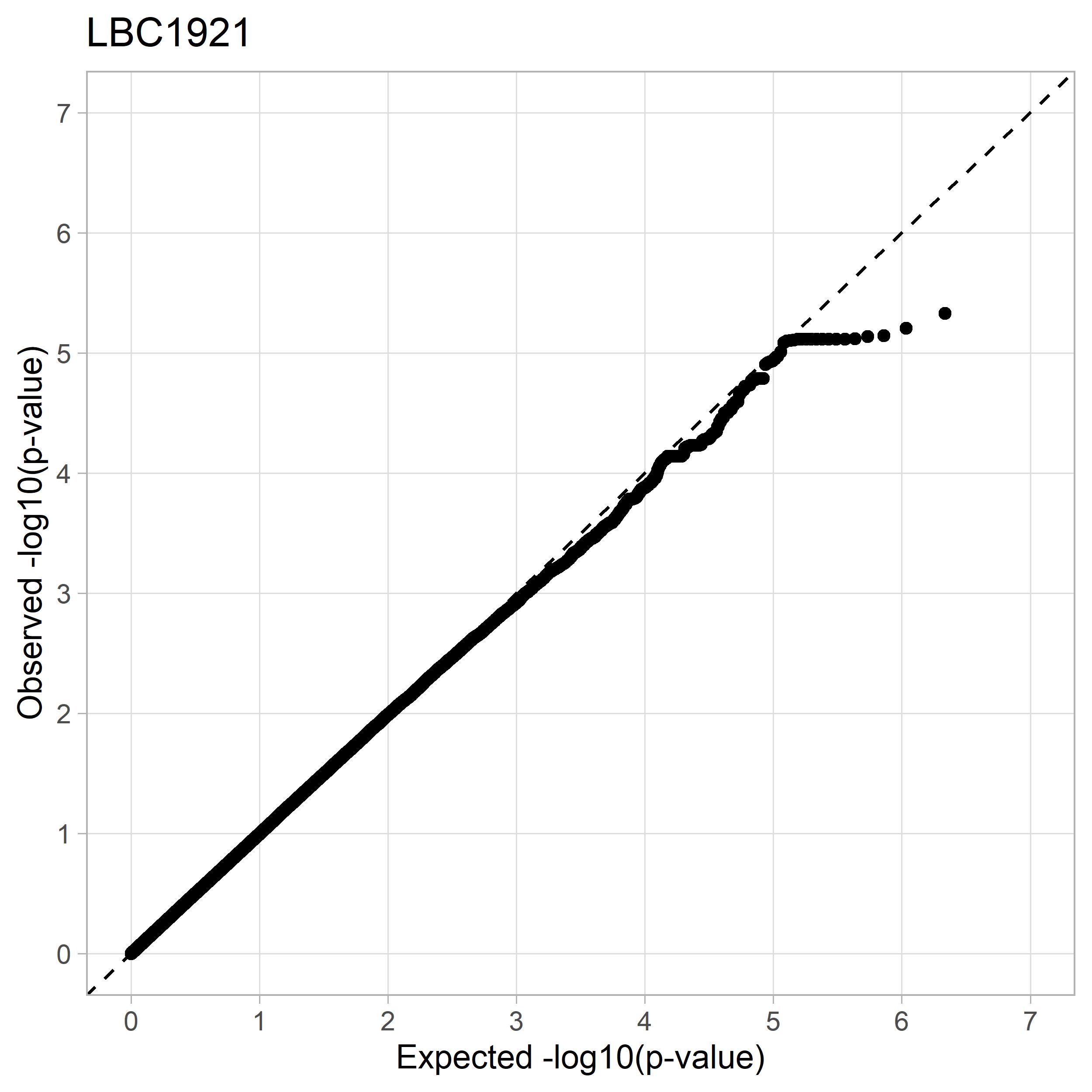

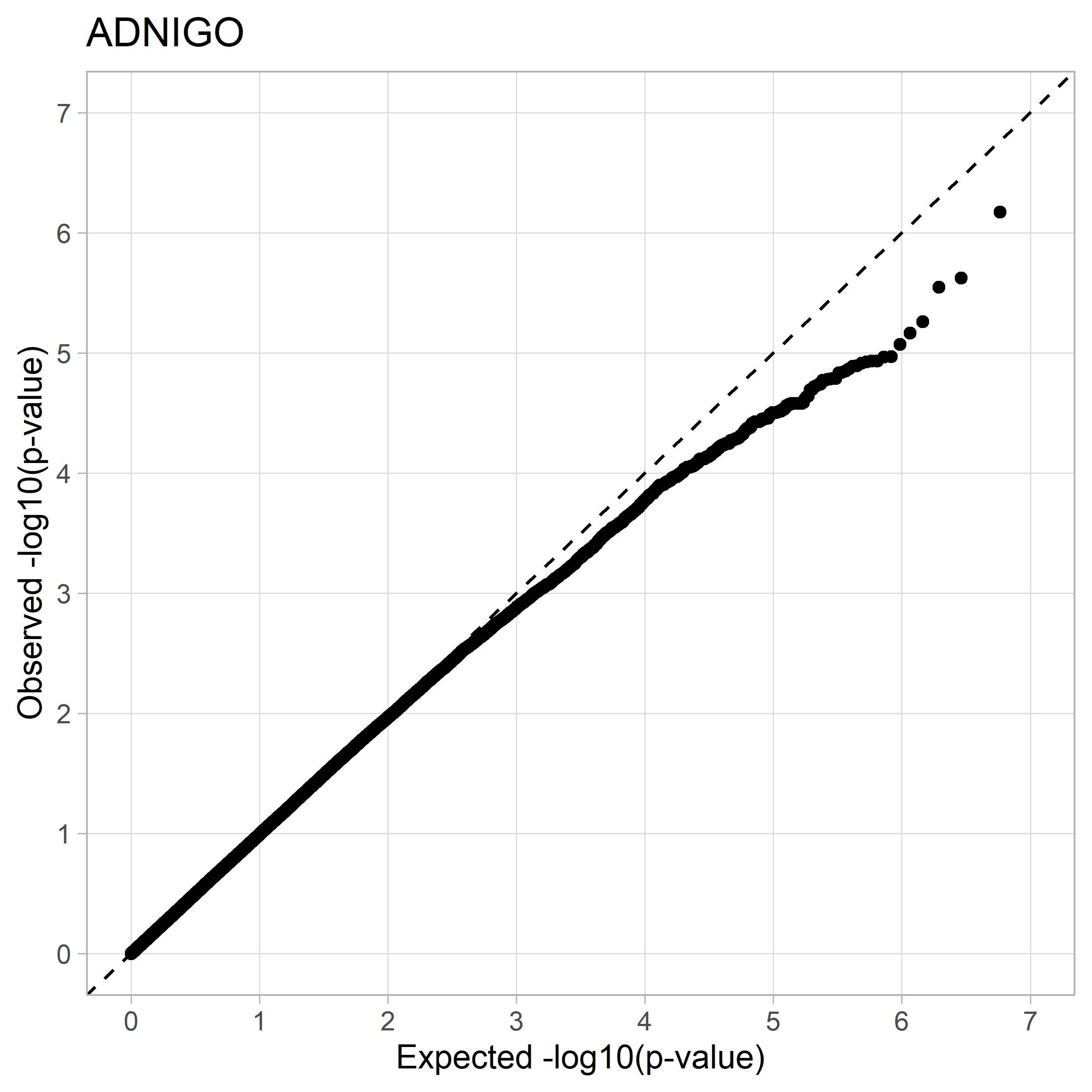


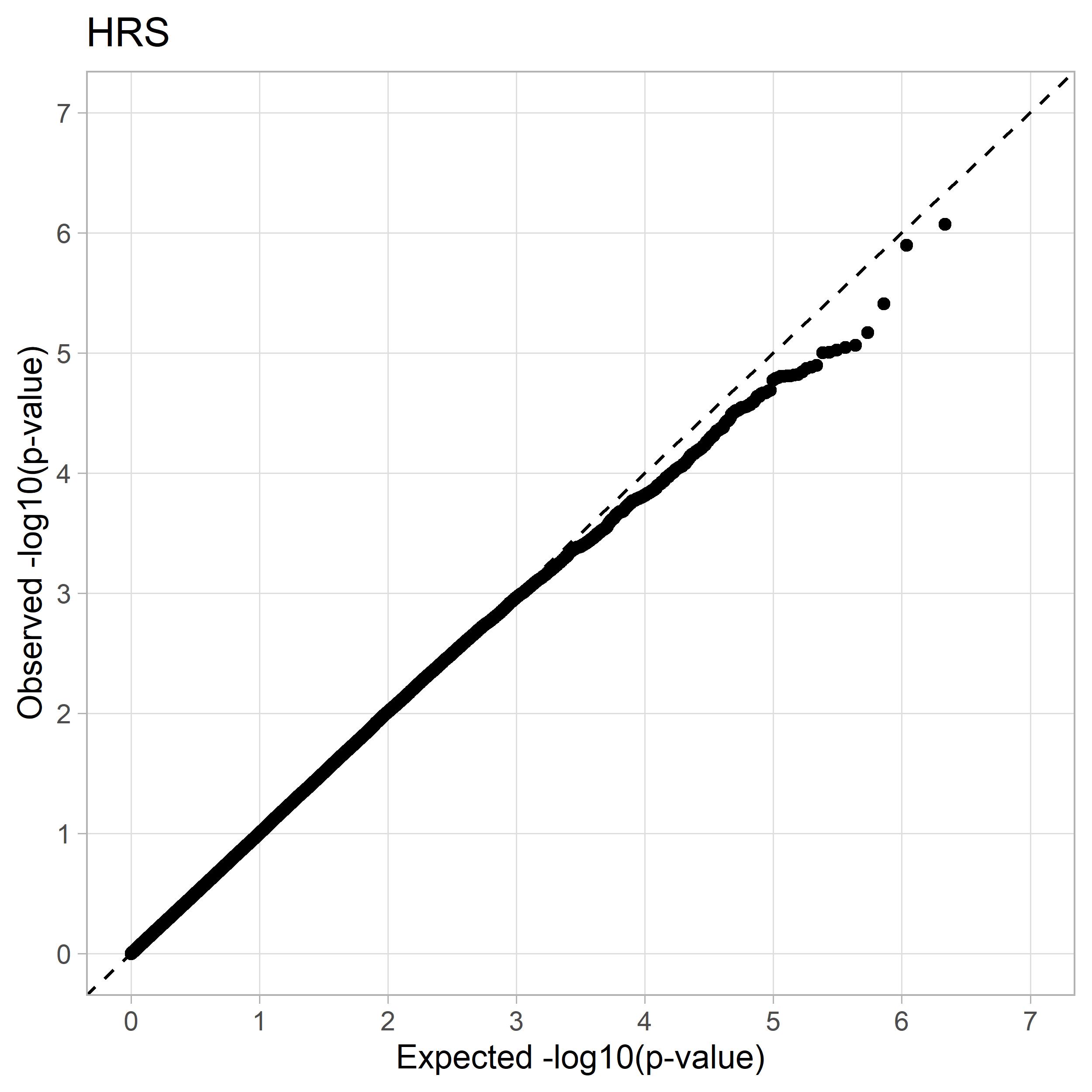

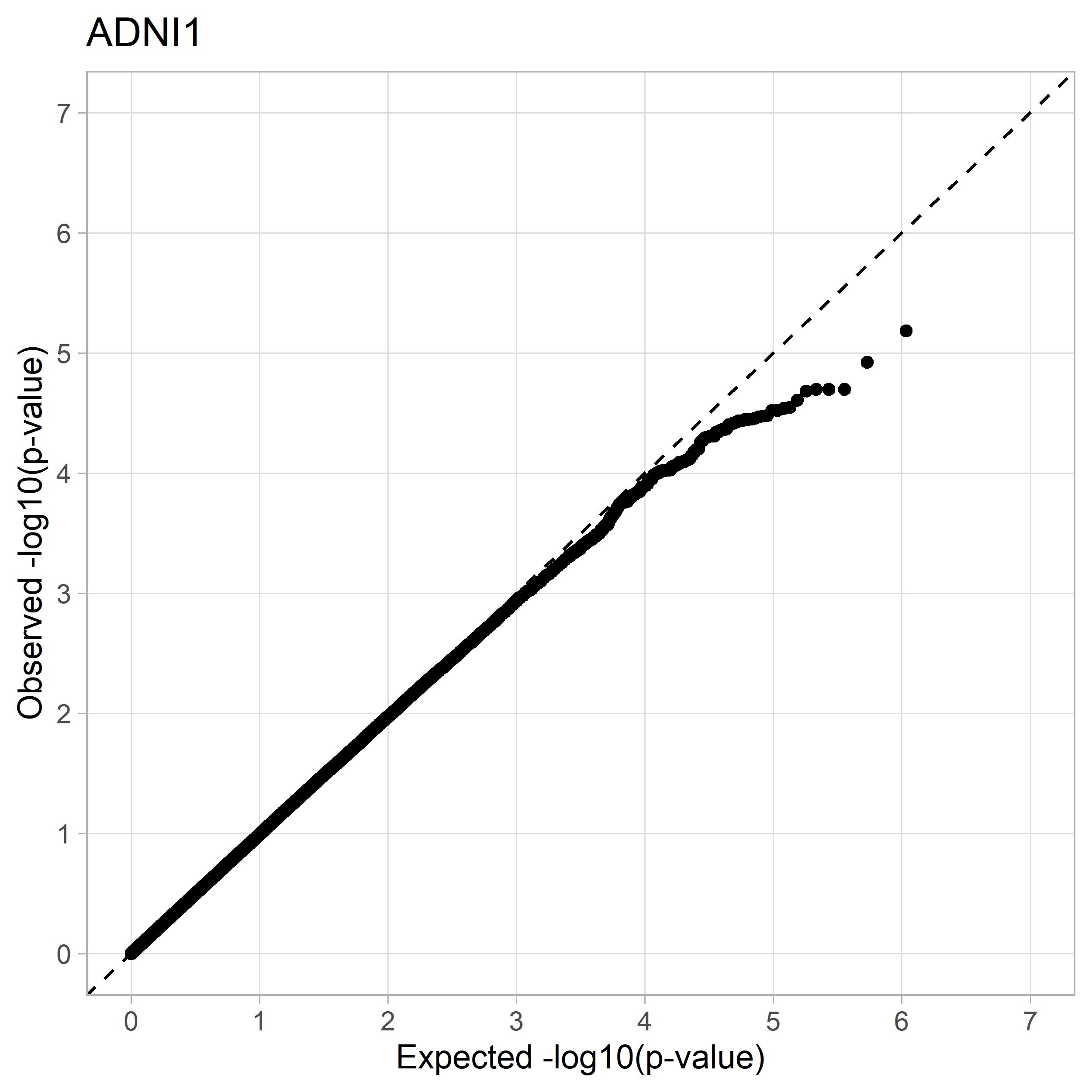


**Supplementary Figure 25:** QQ plots of cohort-level VSTM GWAS (Model 1) results (HRS to RS2).


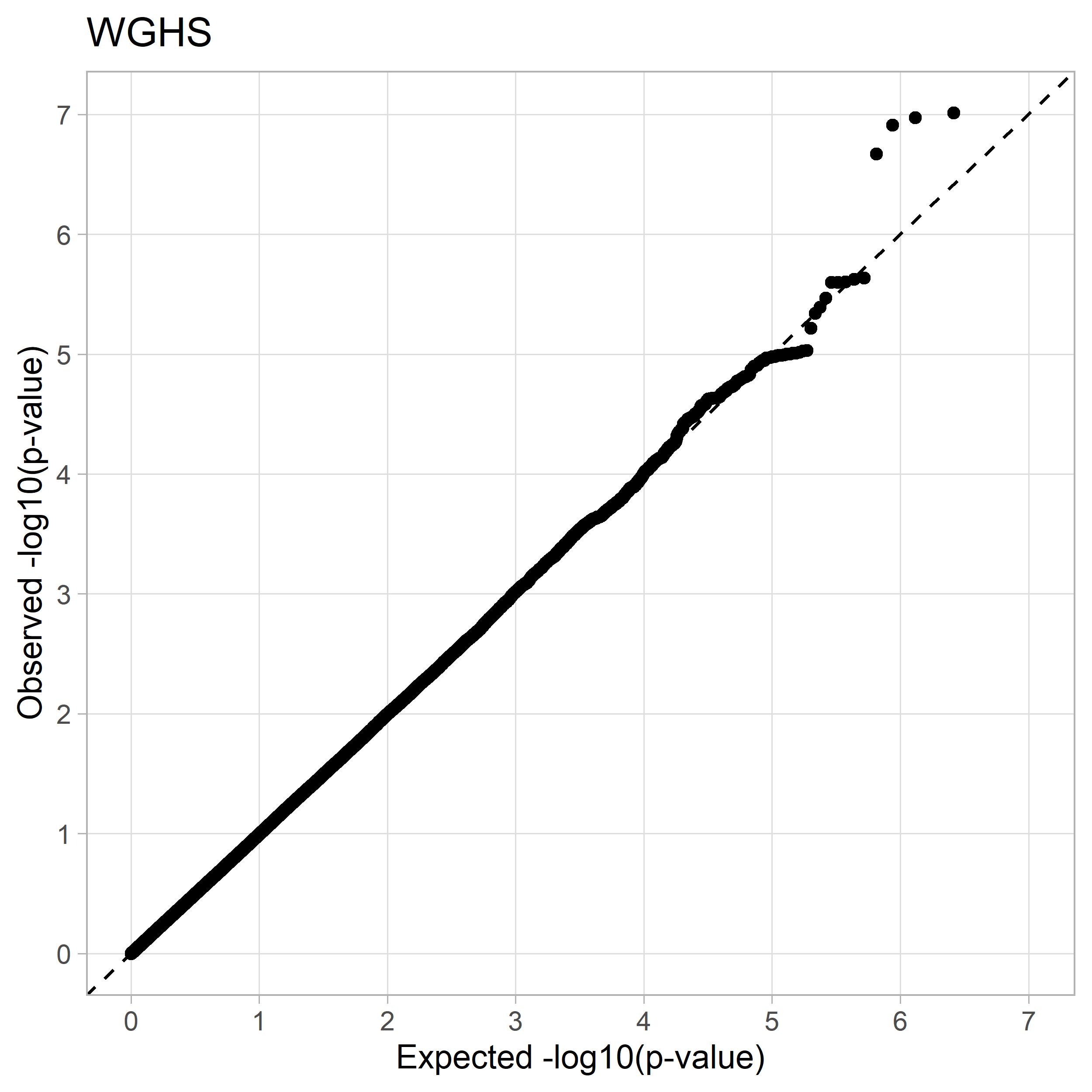

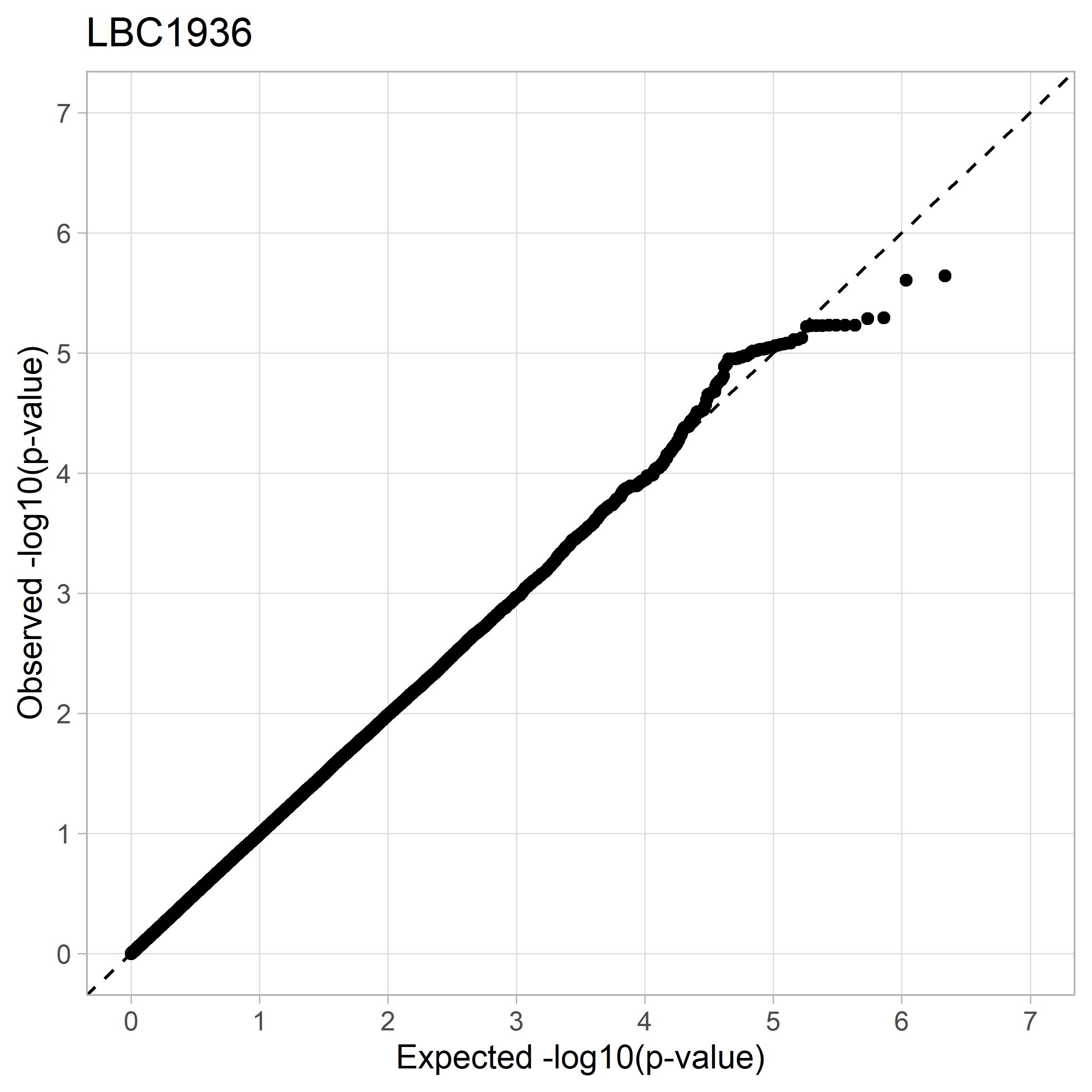

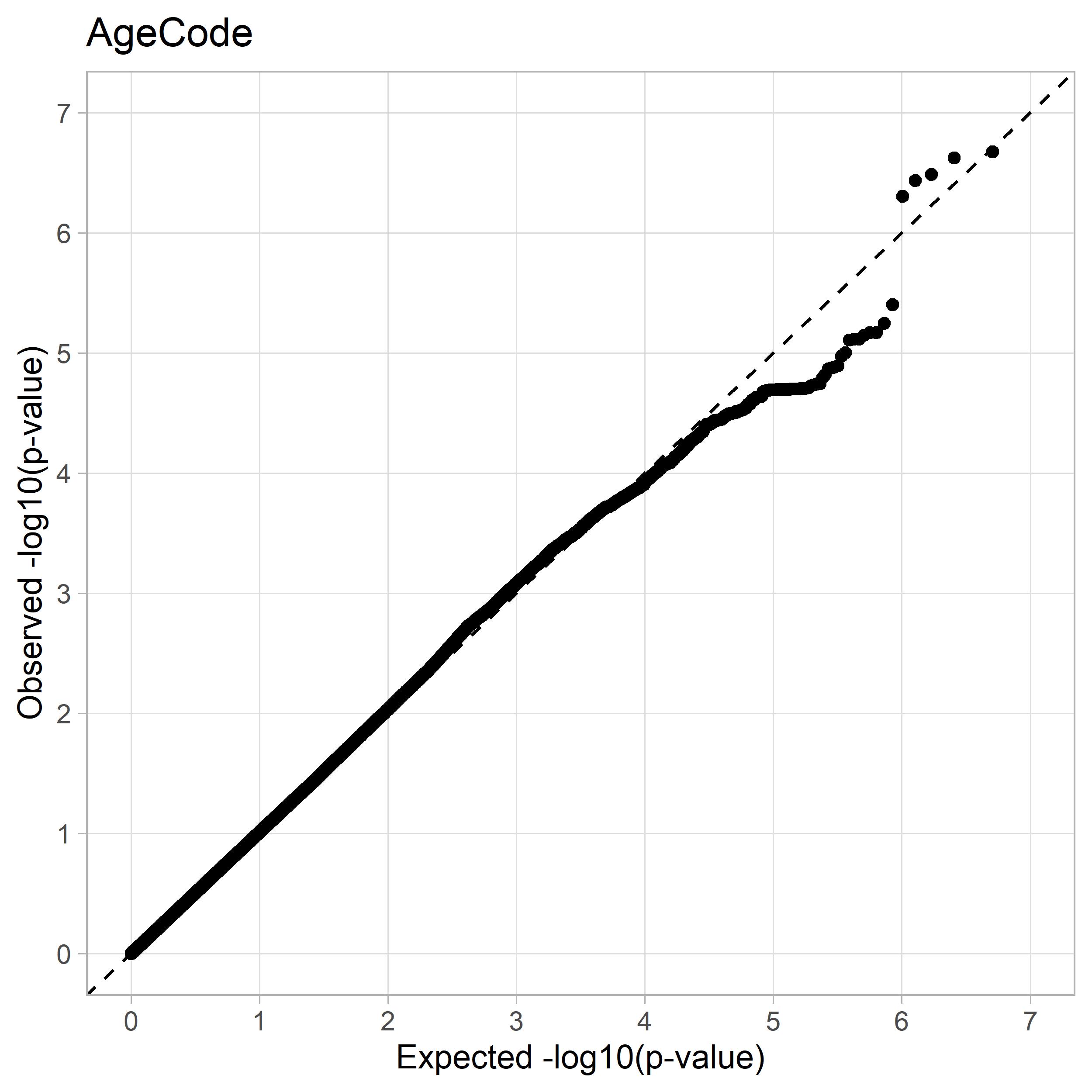


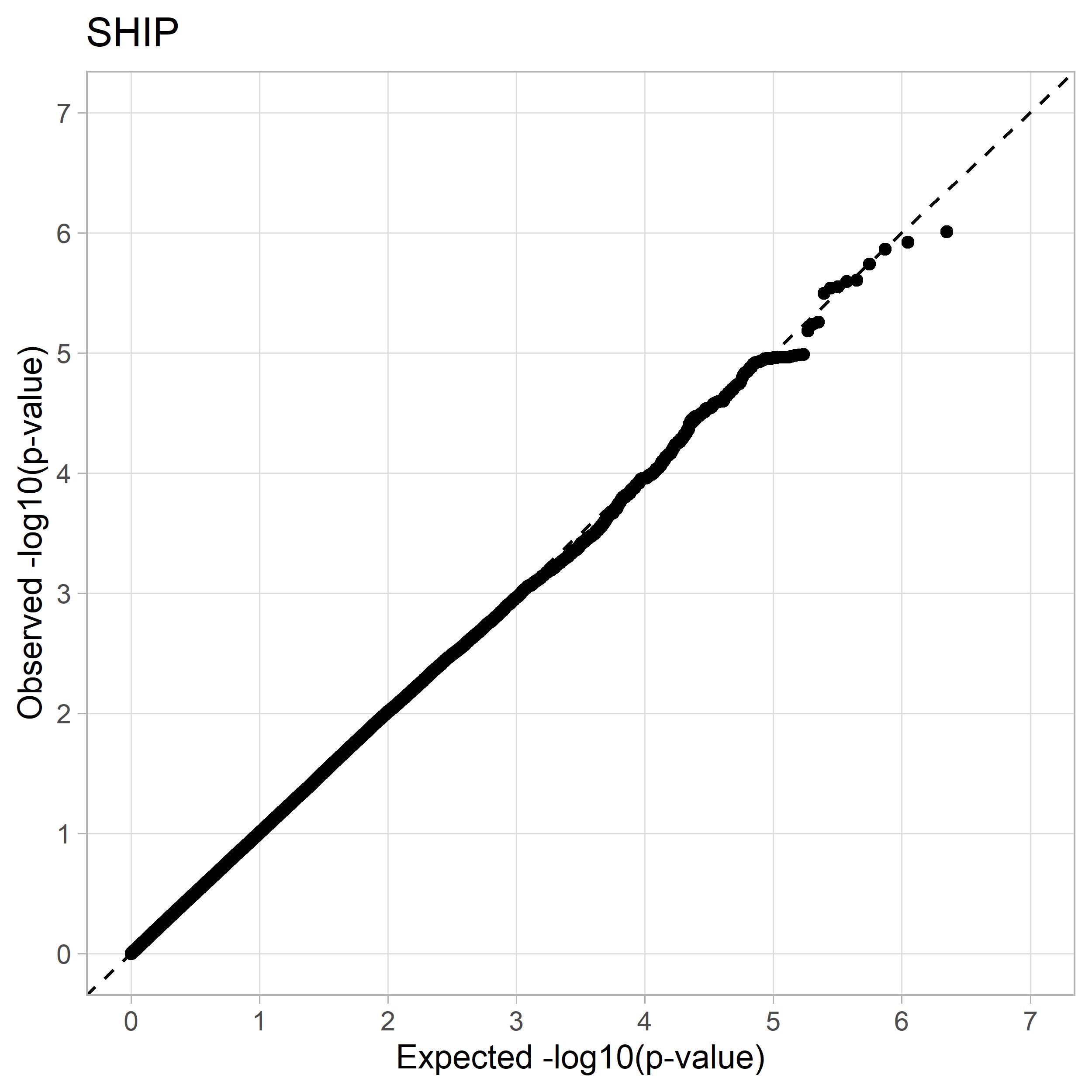

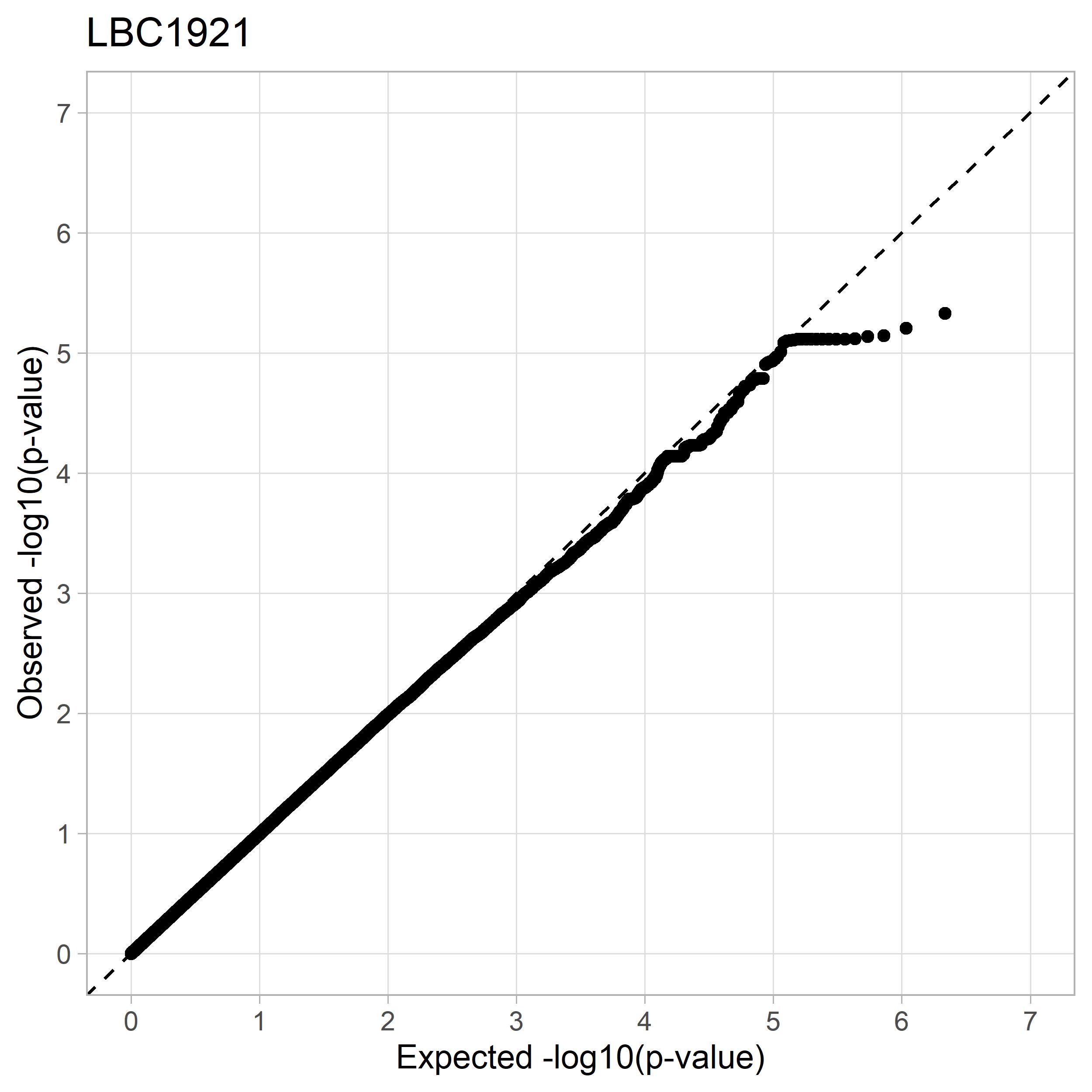

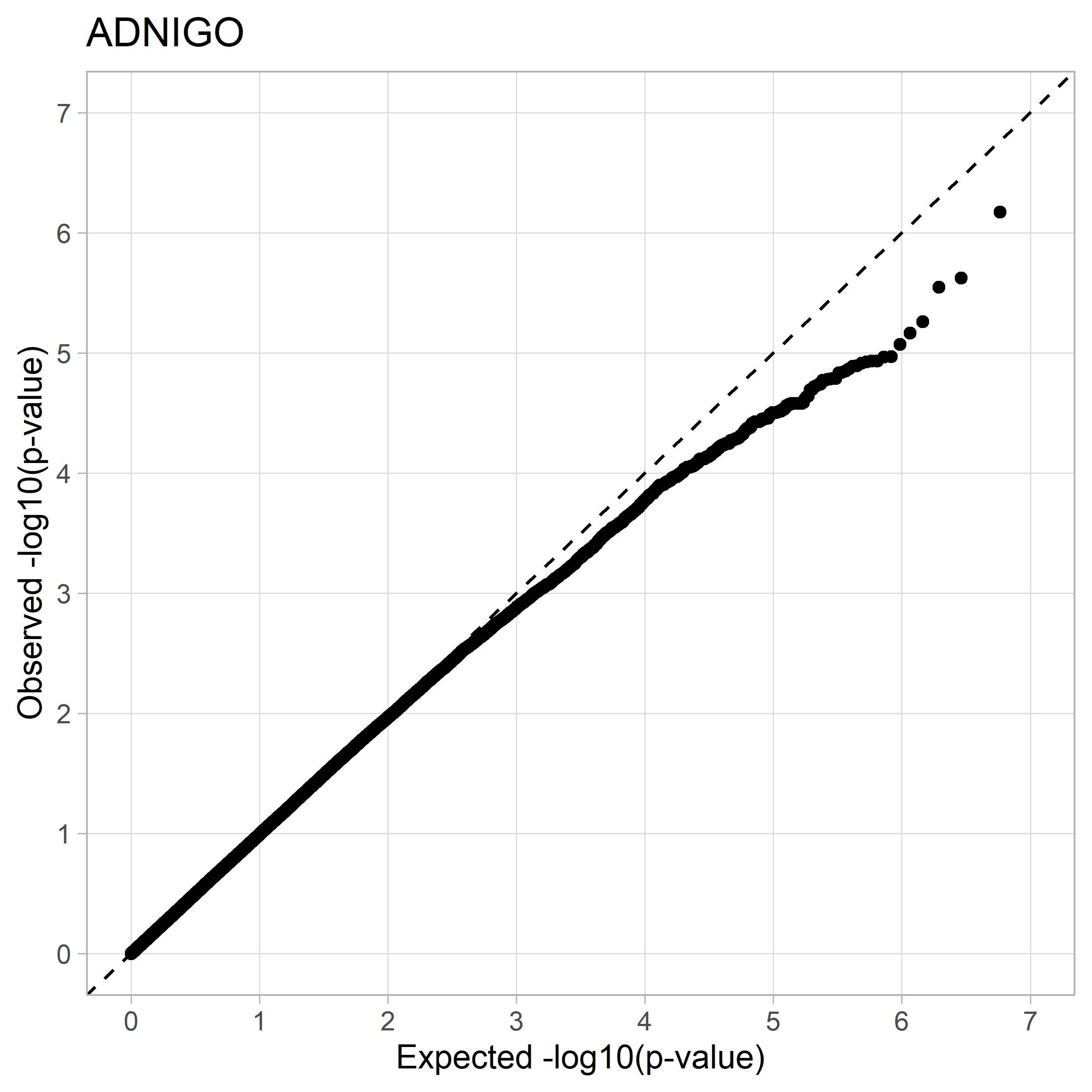


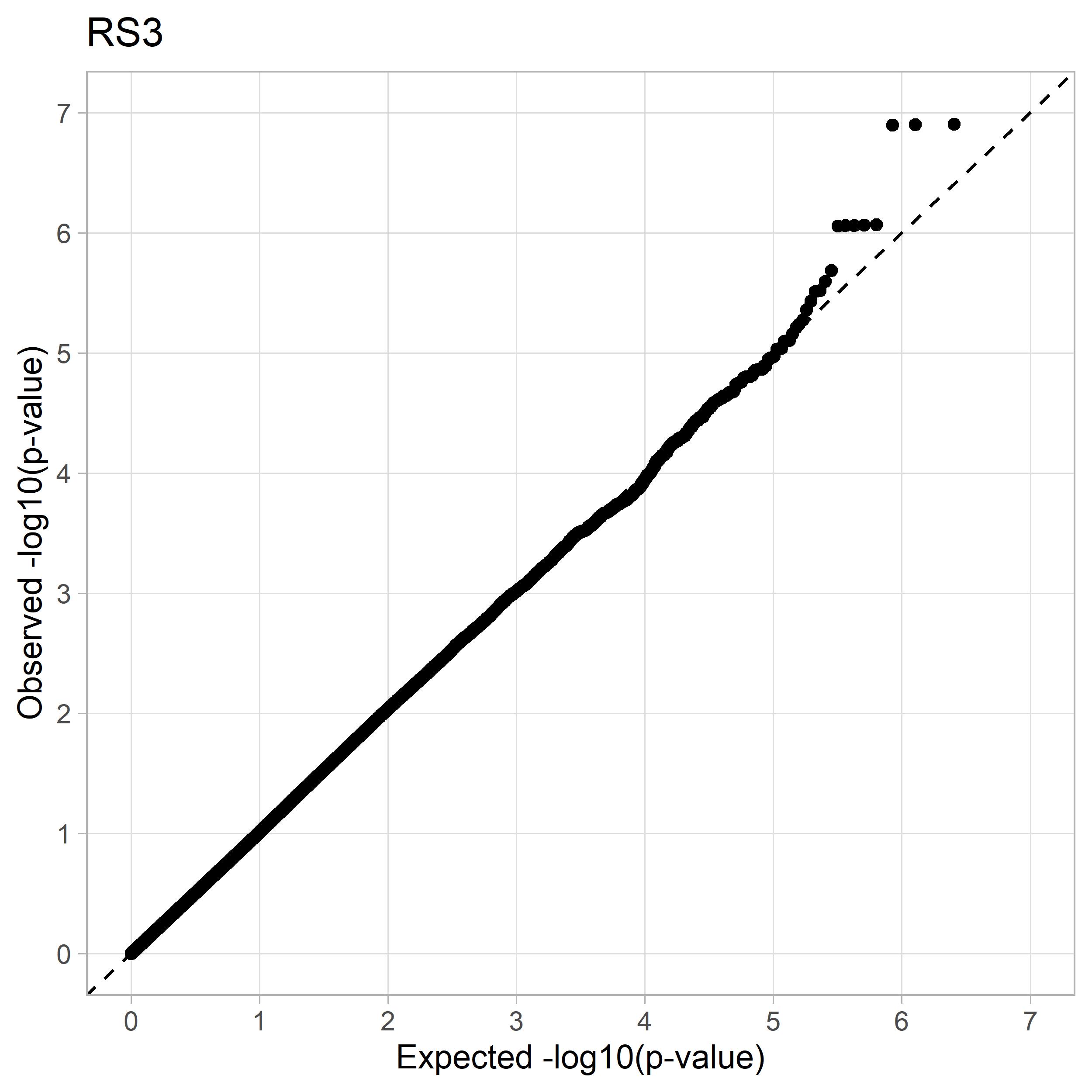

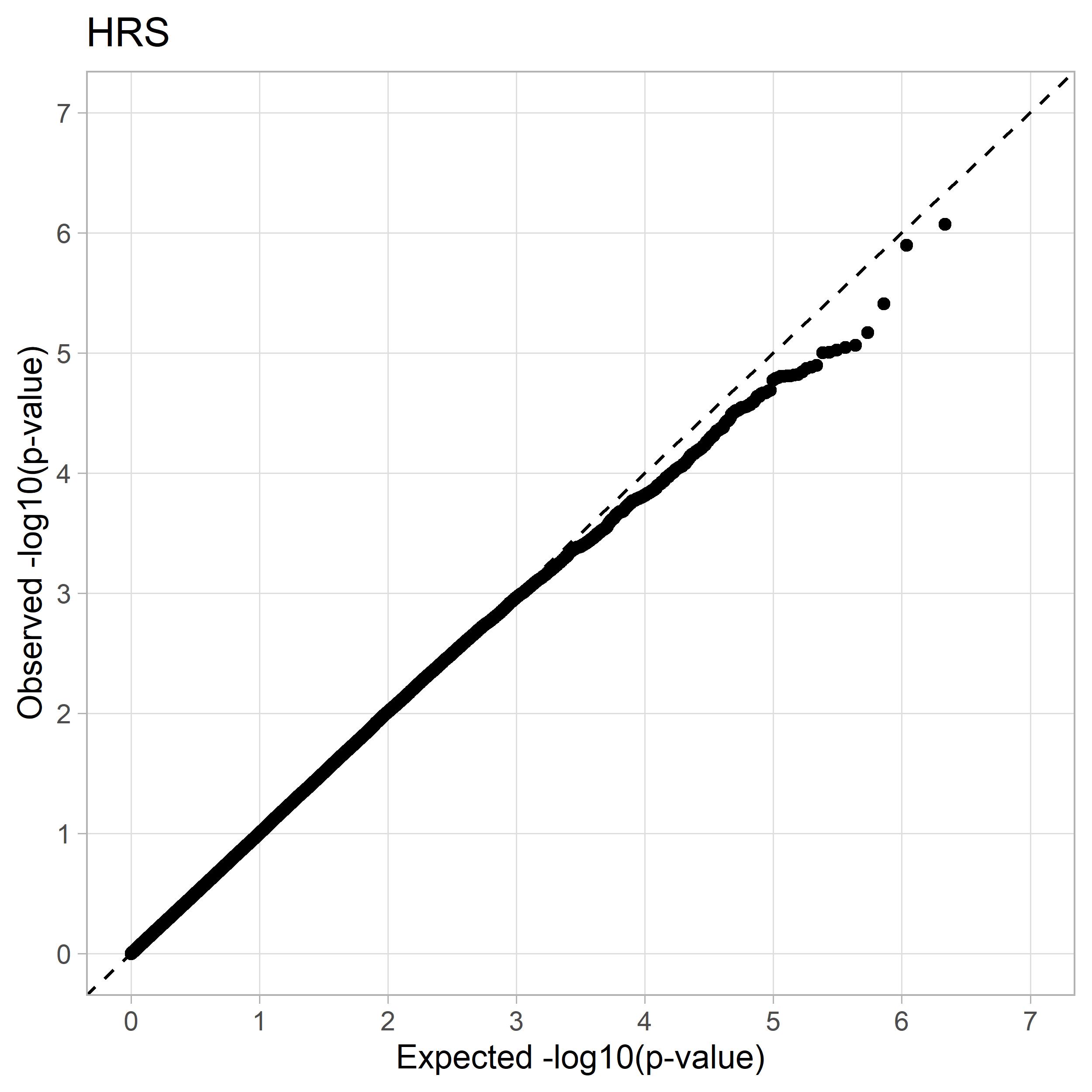

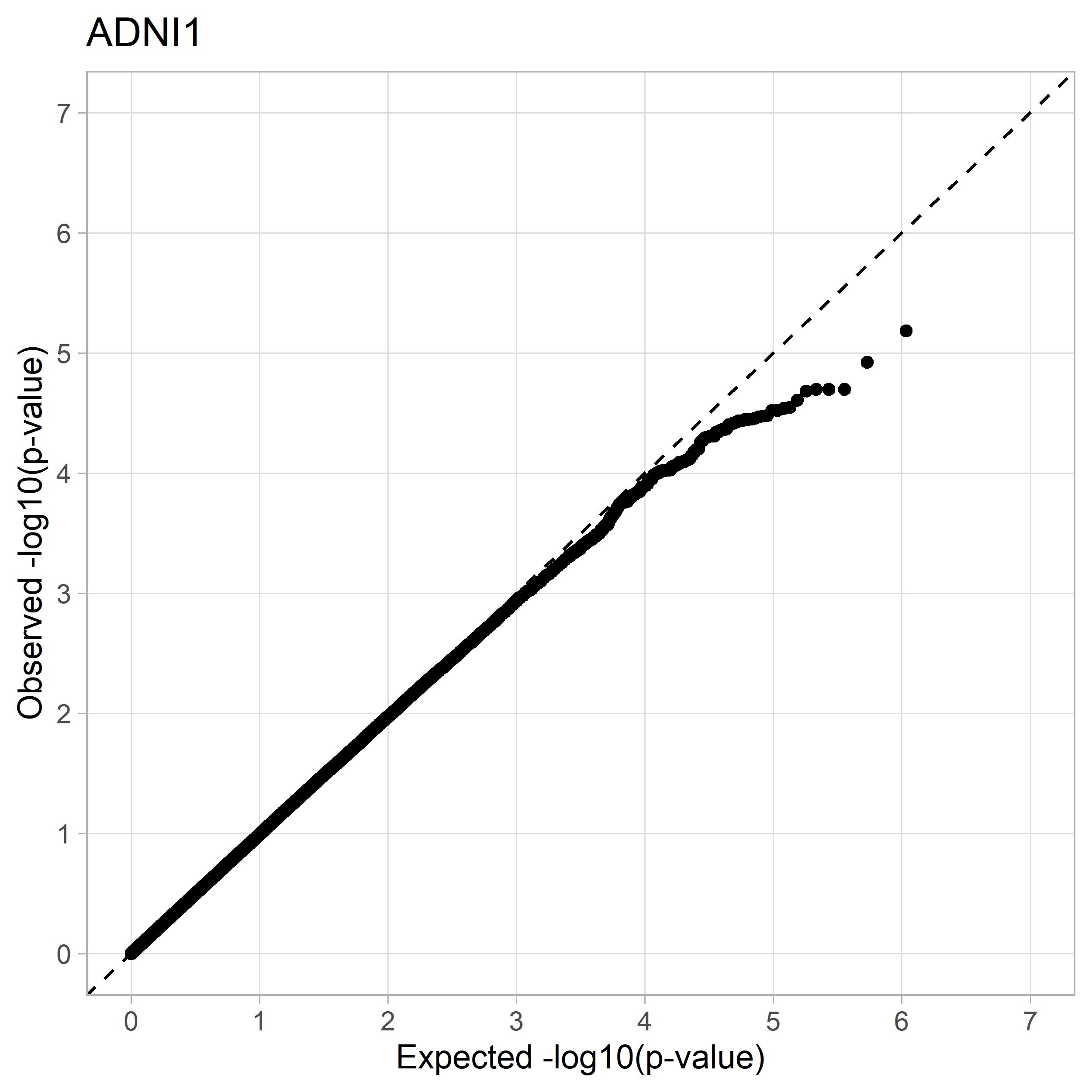


**Supplementary Figure 26:** QQ plots of cohort-level VSTM GWAS (Model 1) results (RS3 to WGHS).

**Supplementary Figure 27:** QQ plots of cohort-level VL GWAS (Model 1) results (ADNI to LIFEAdult).


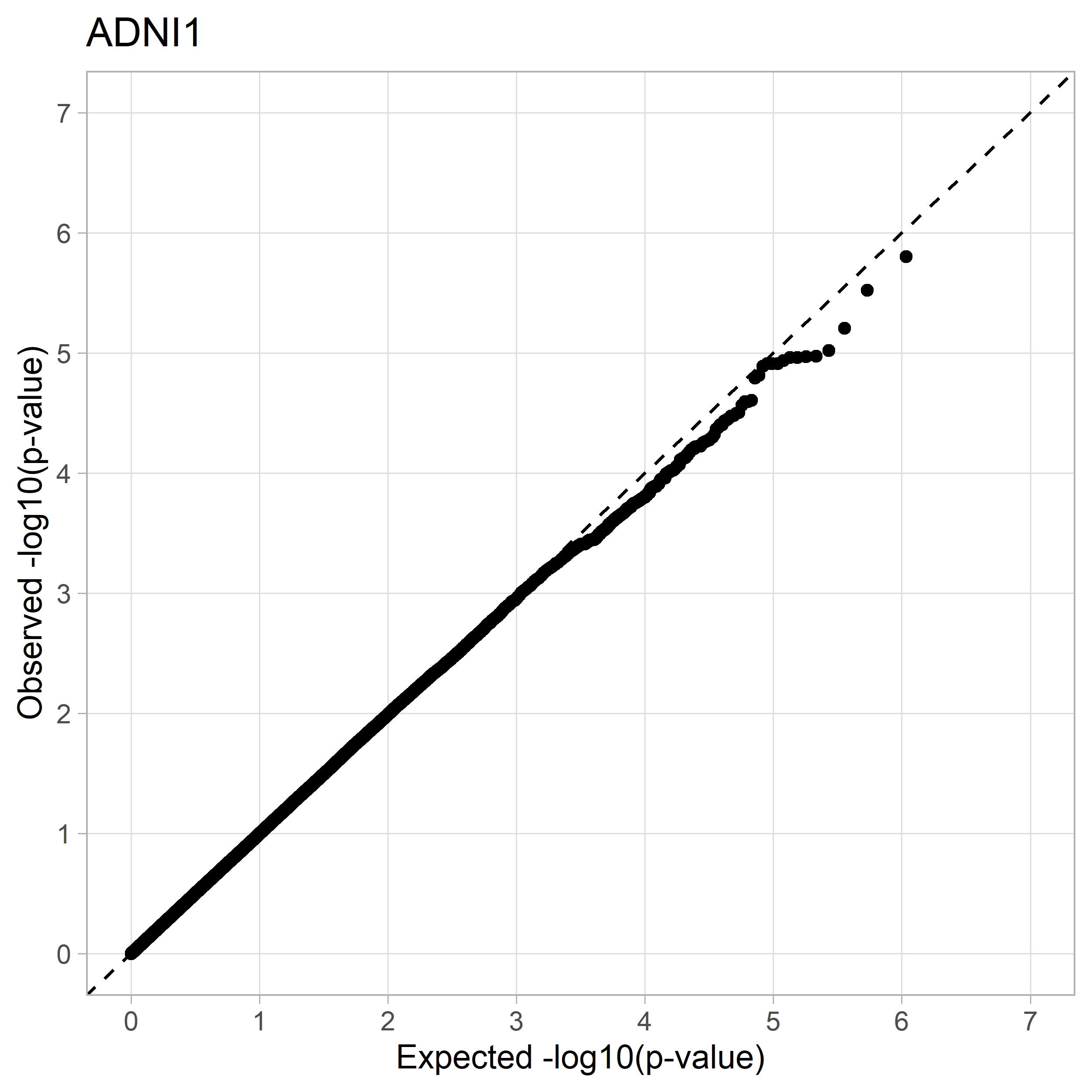

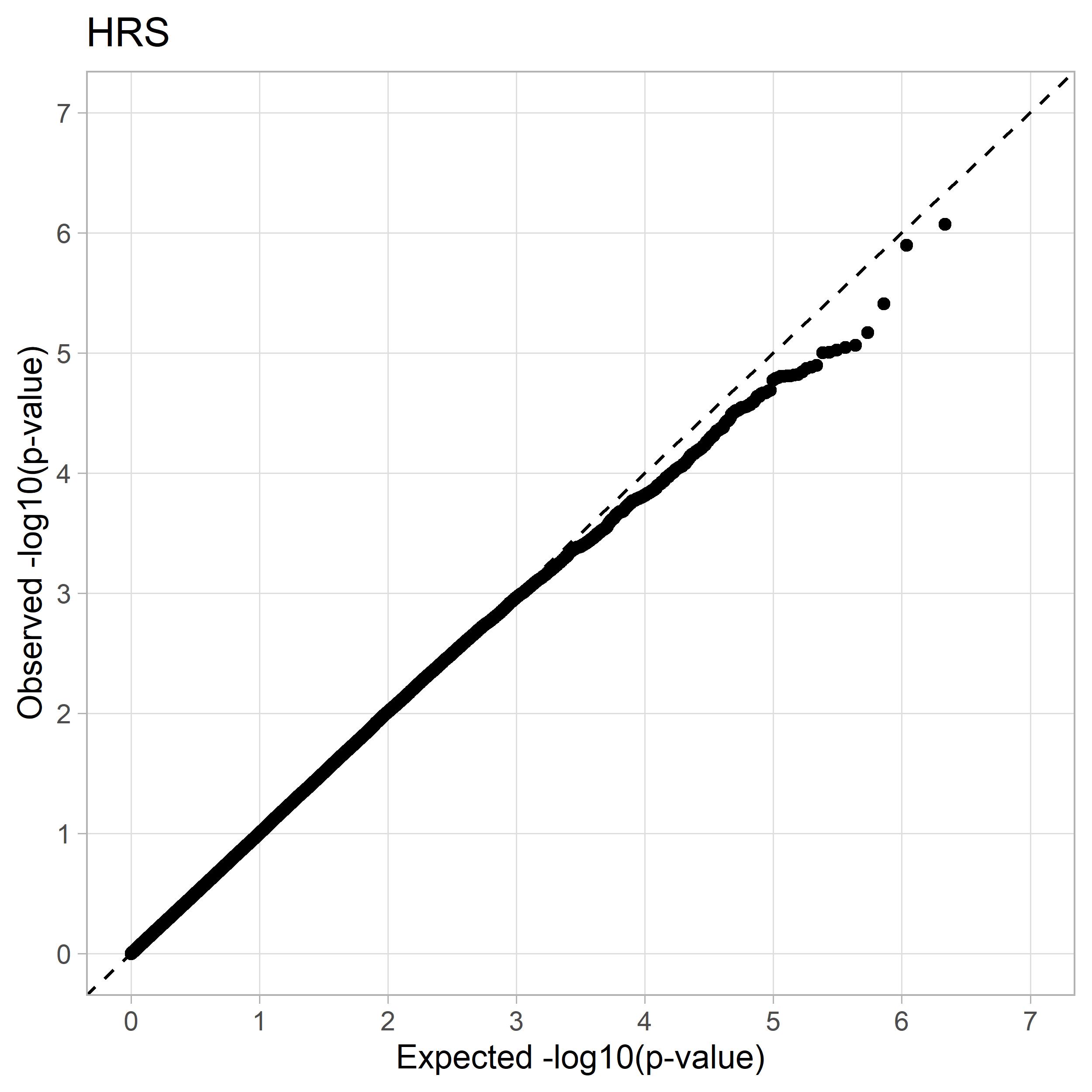

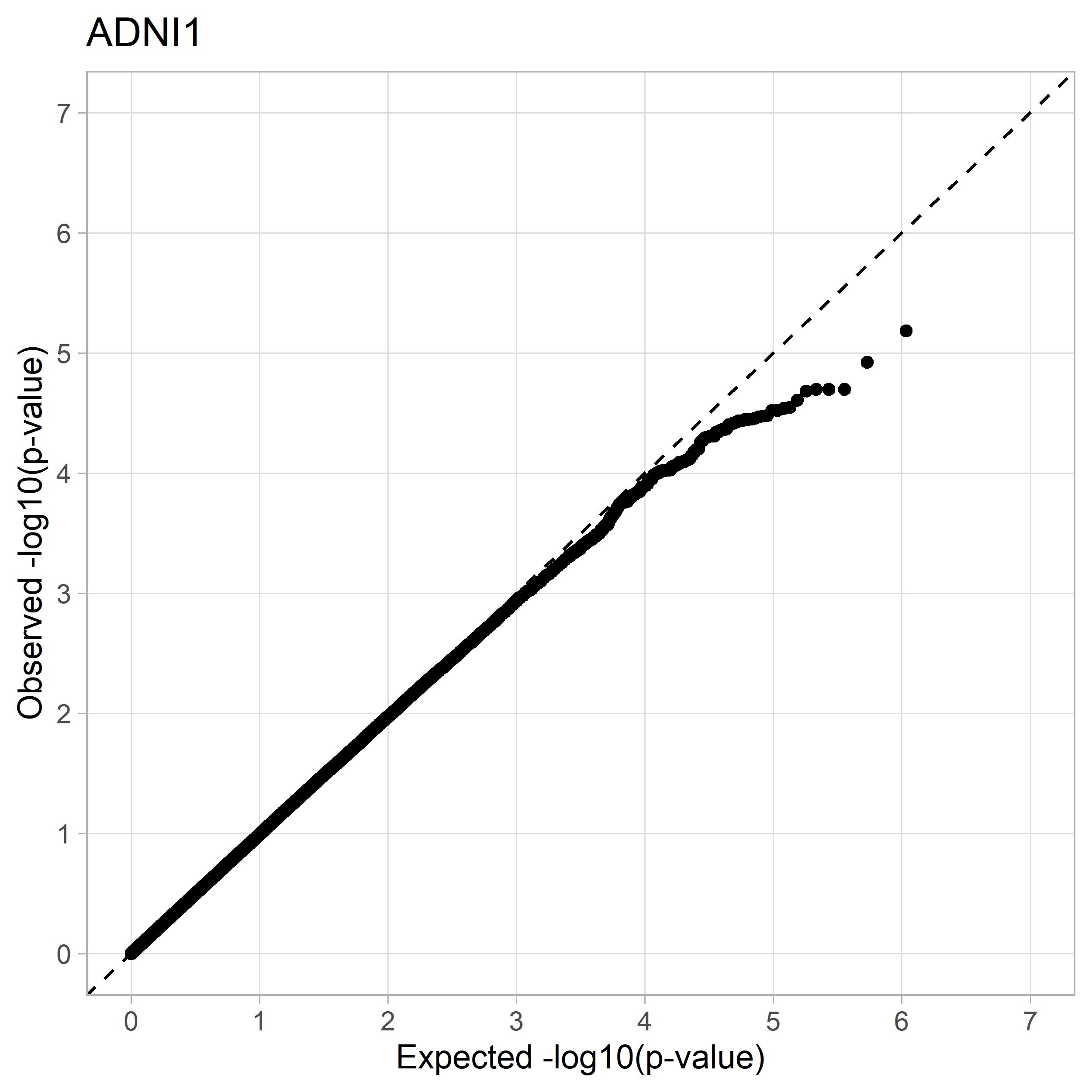


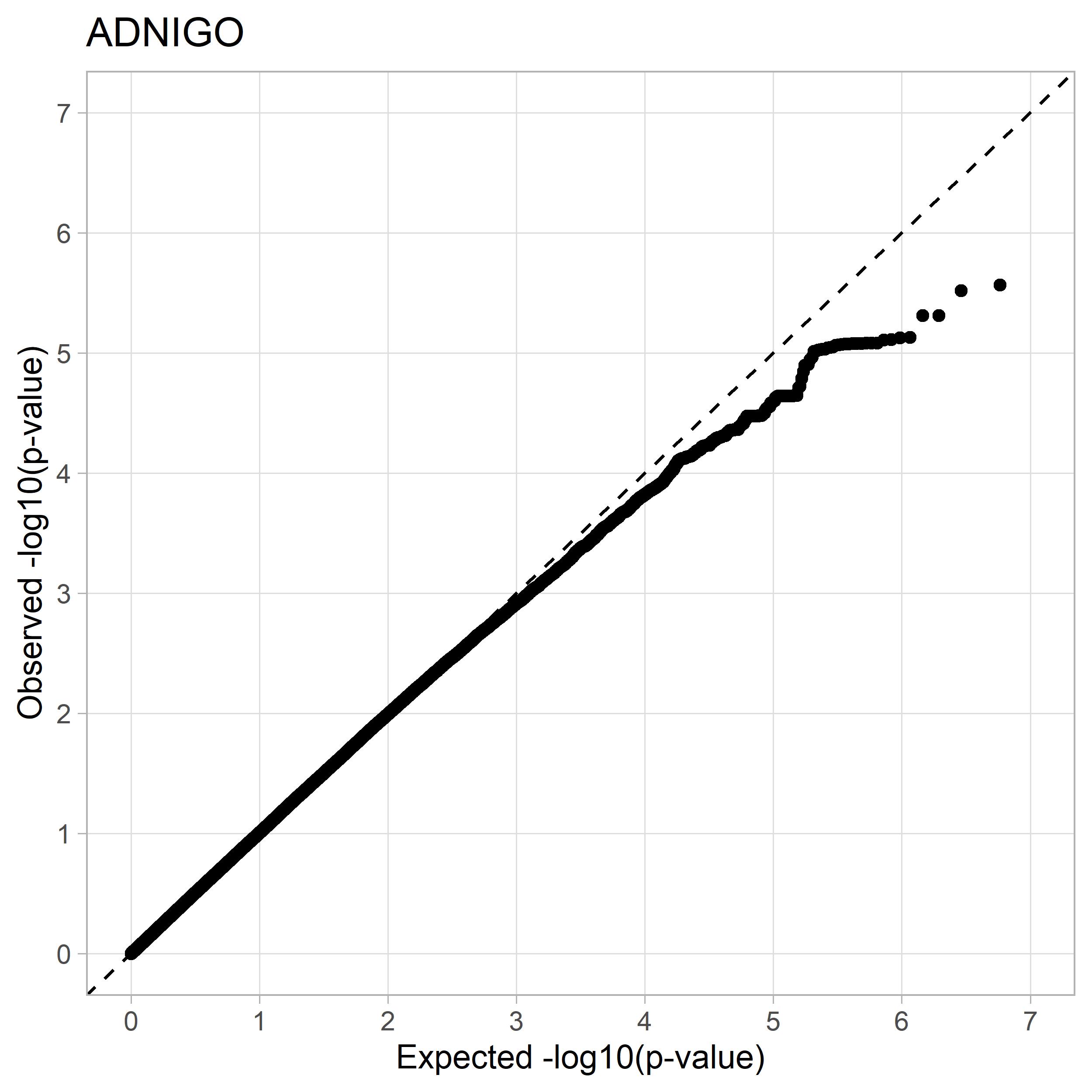

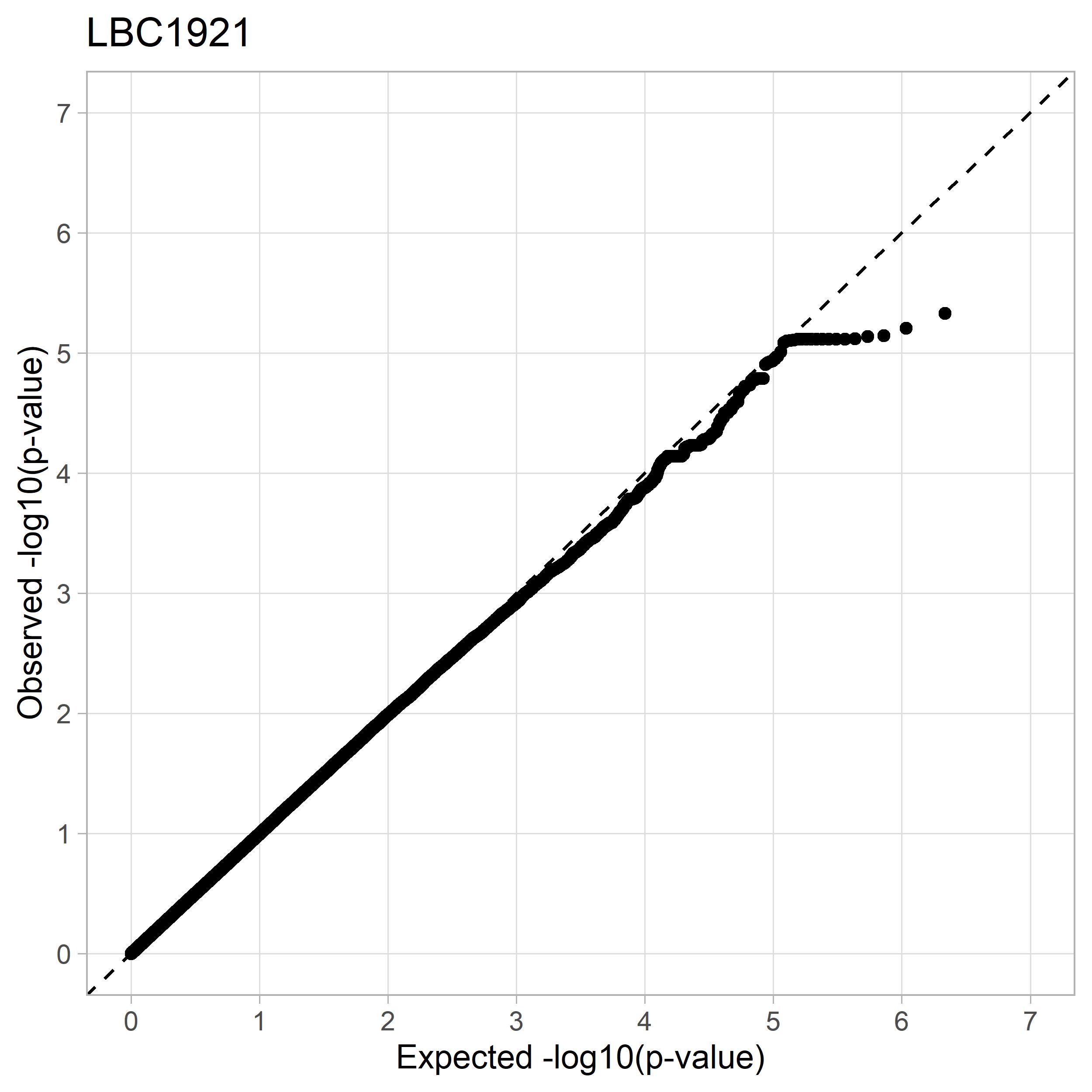

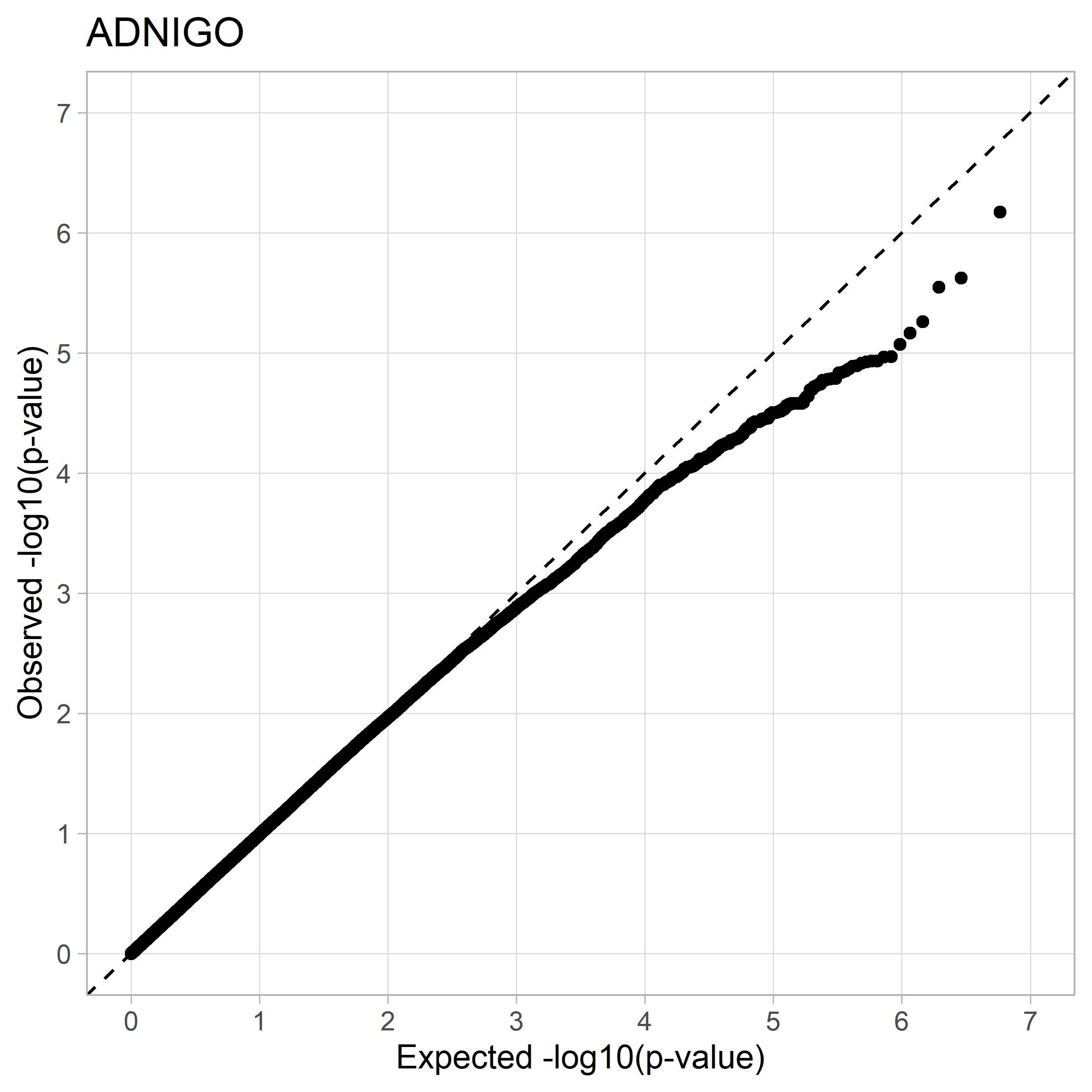


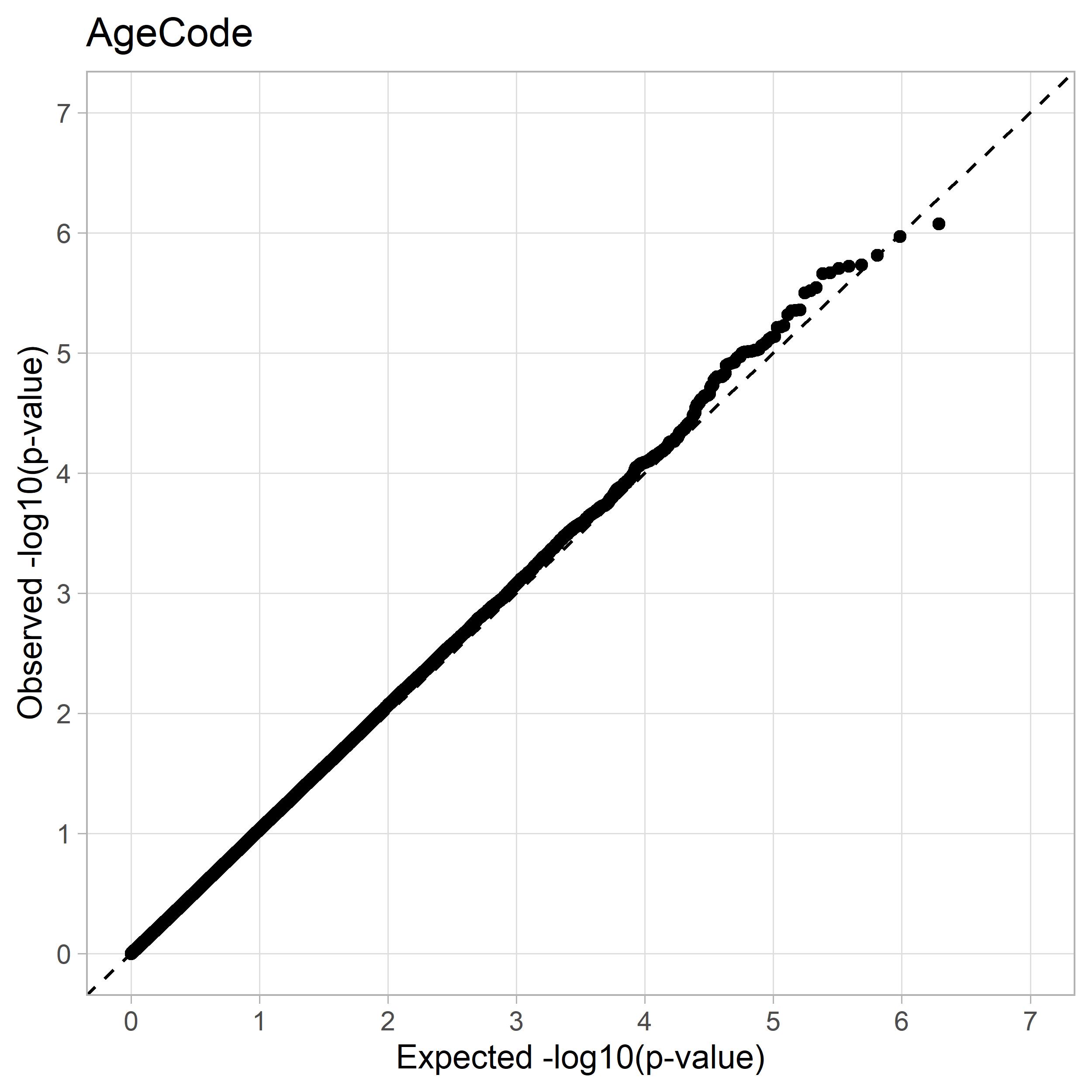

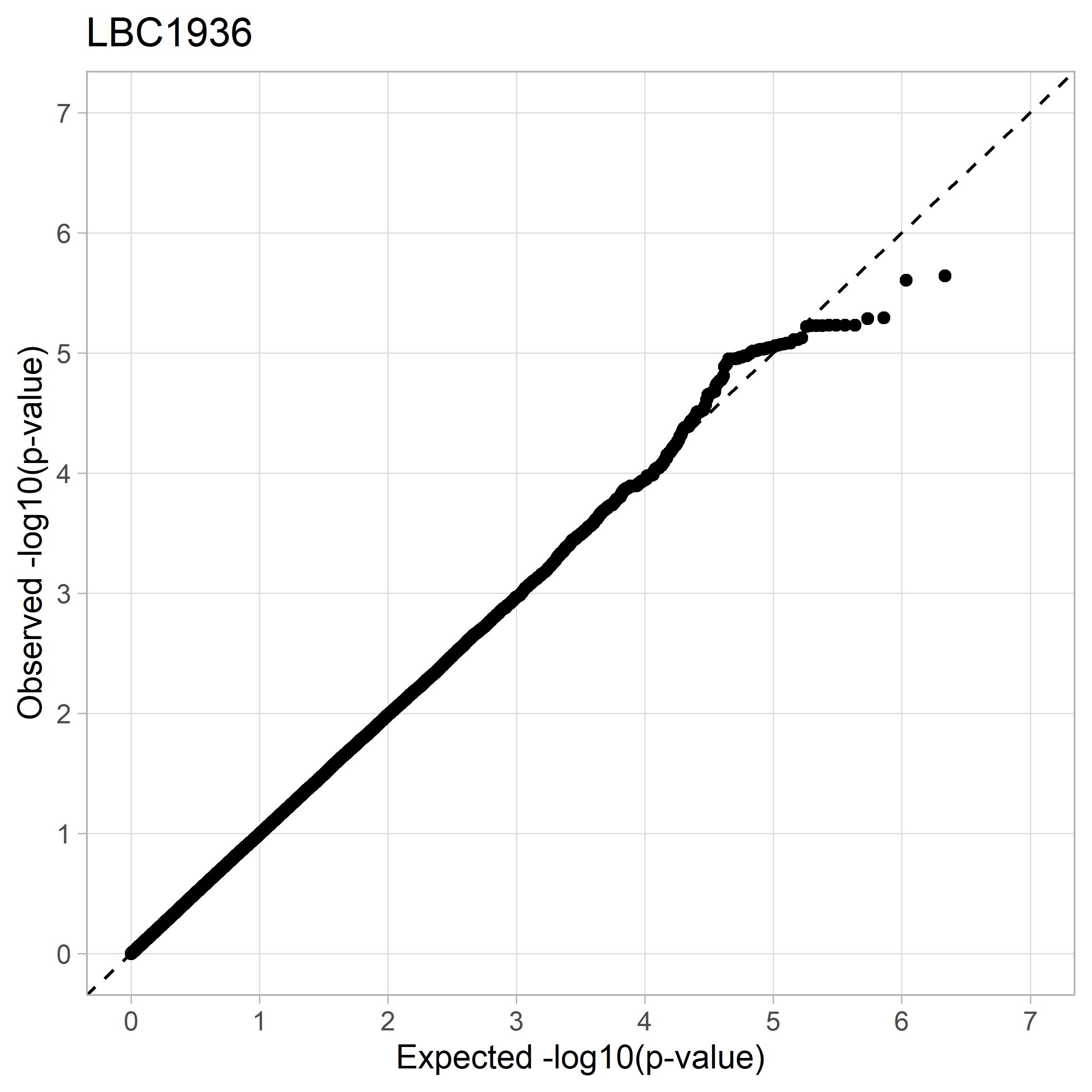

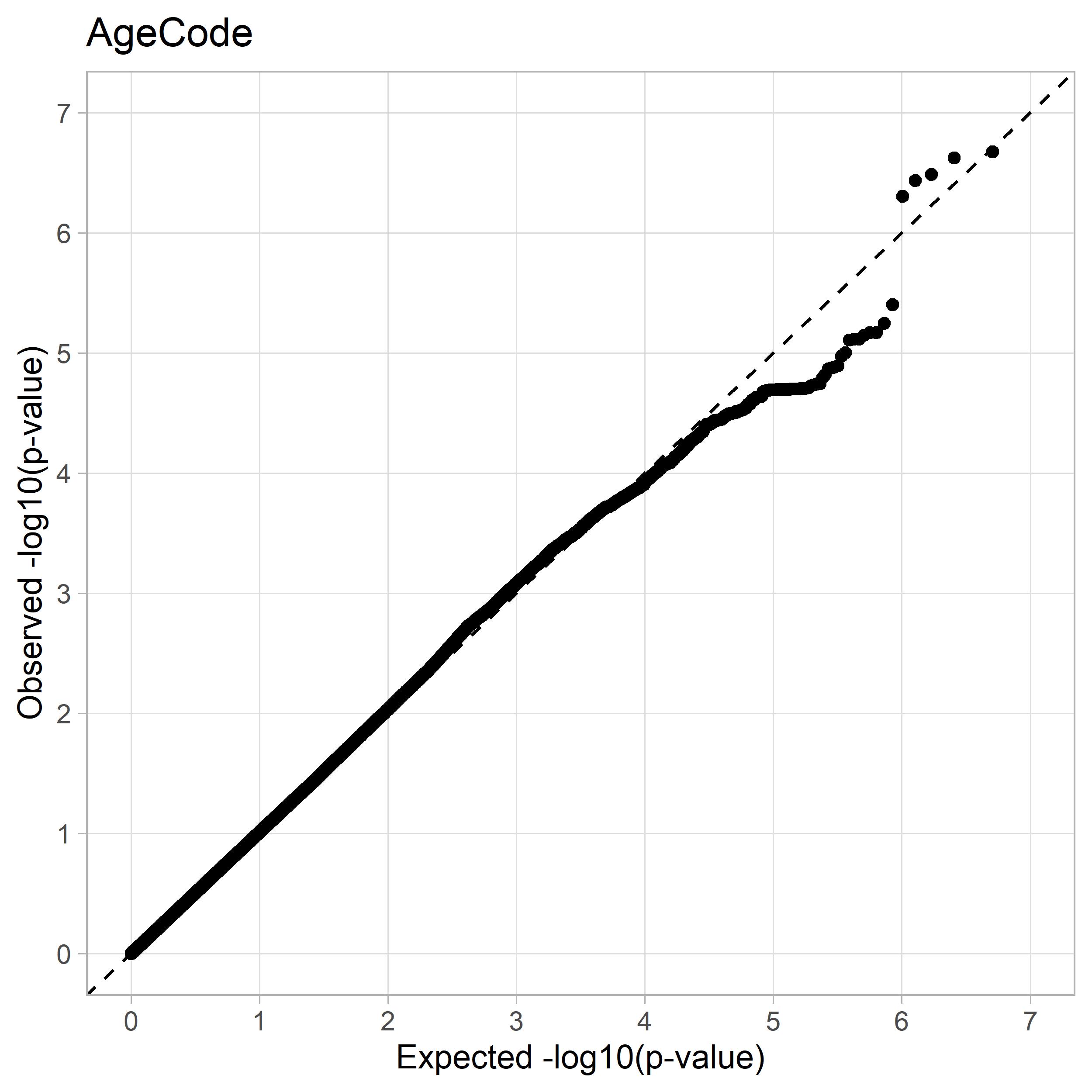


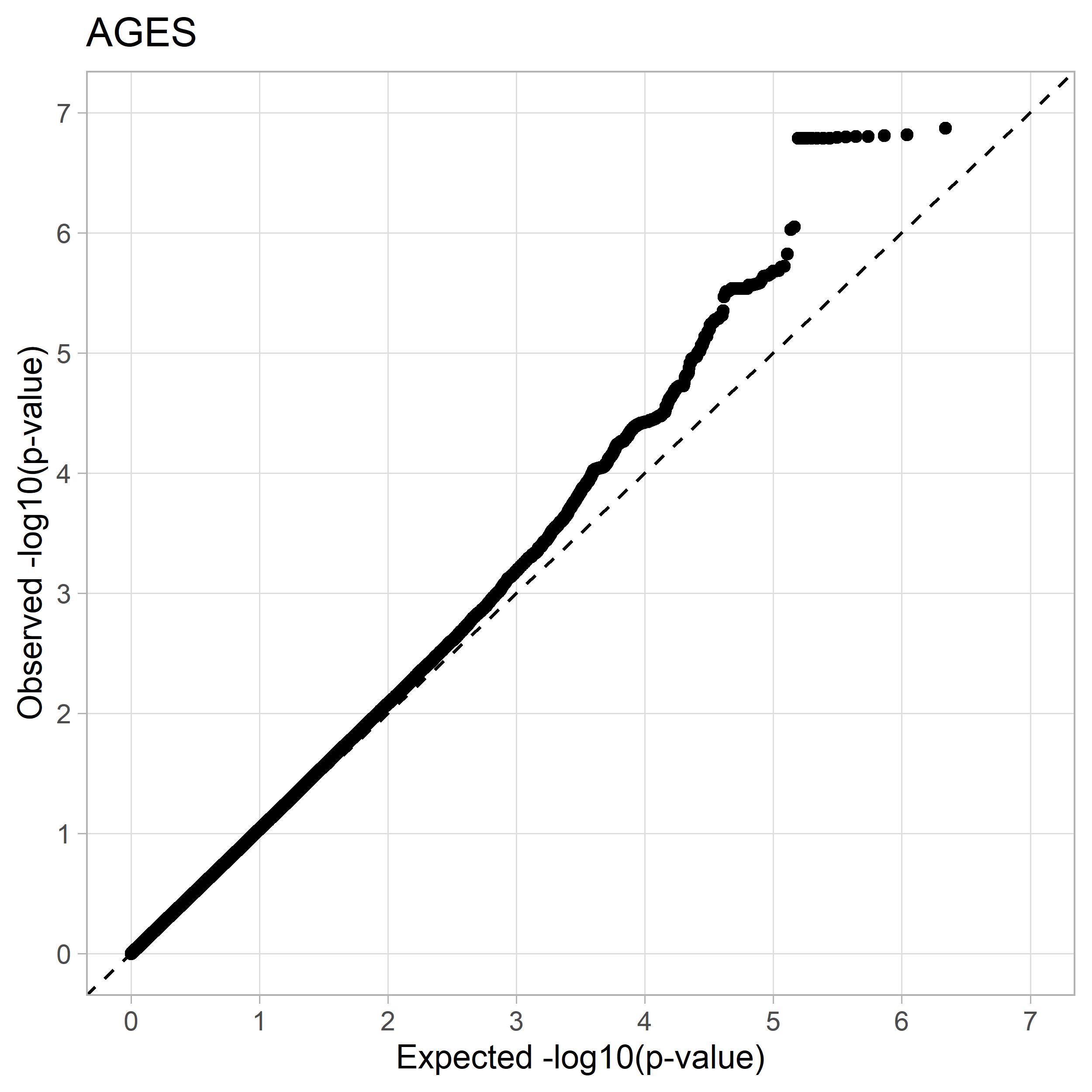


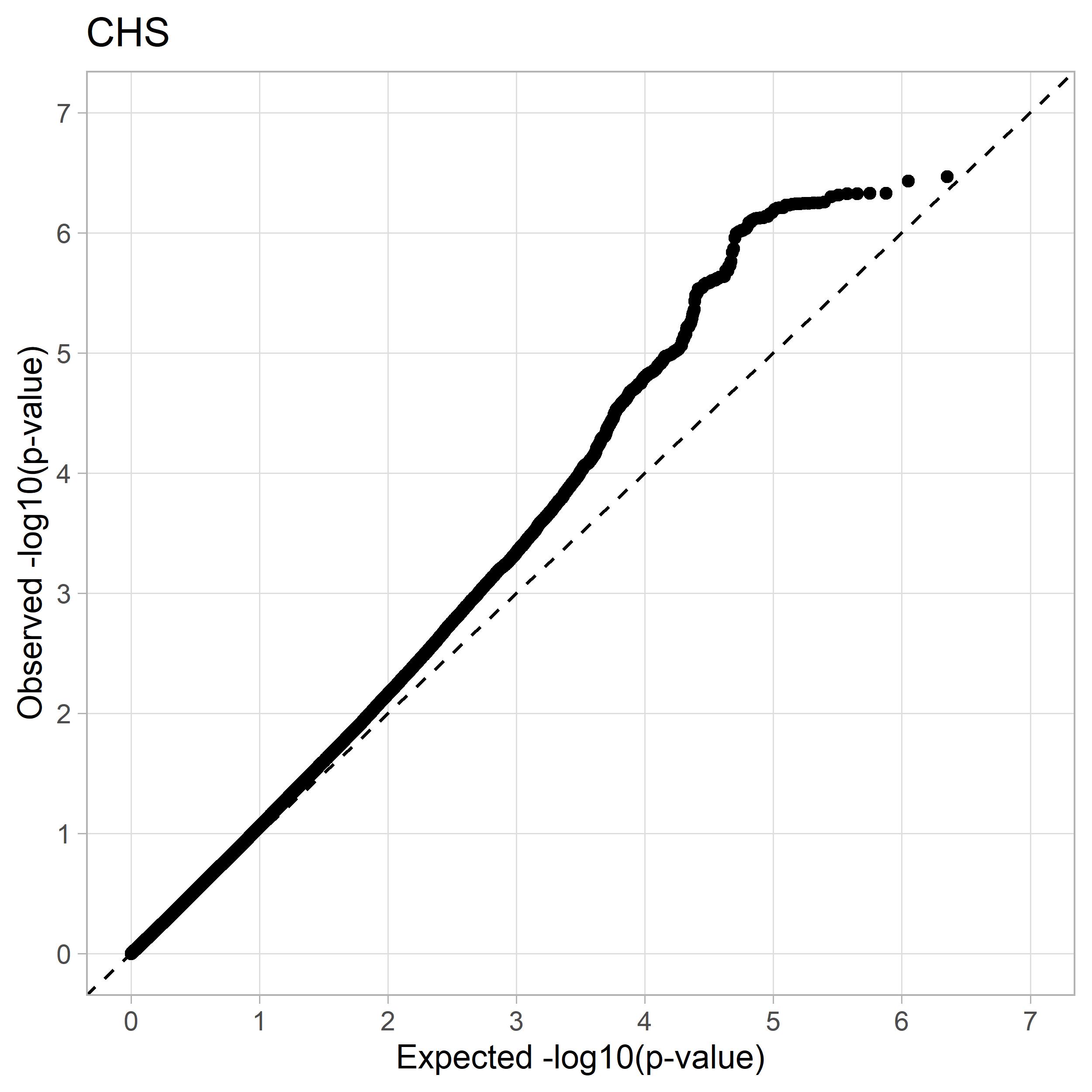


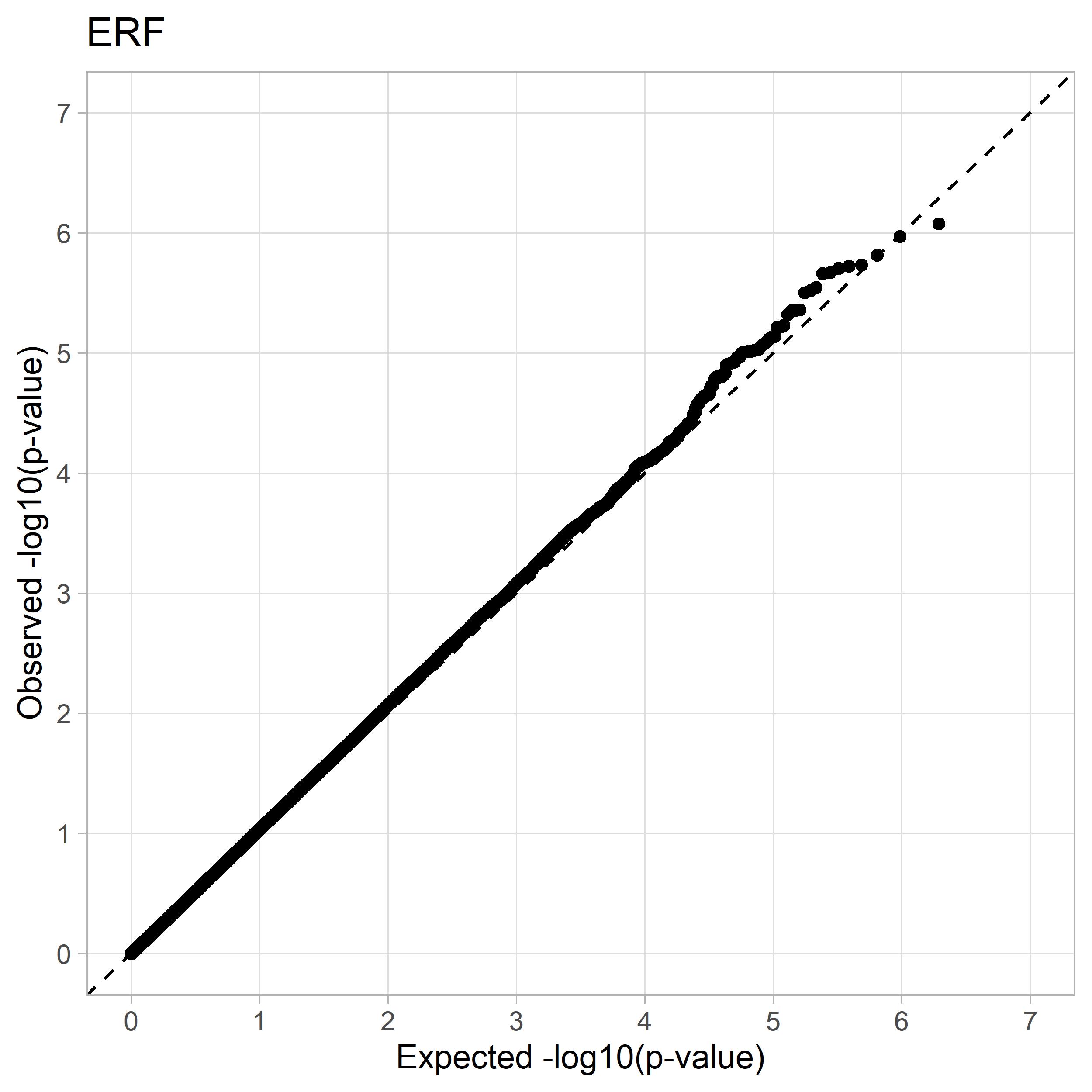


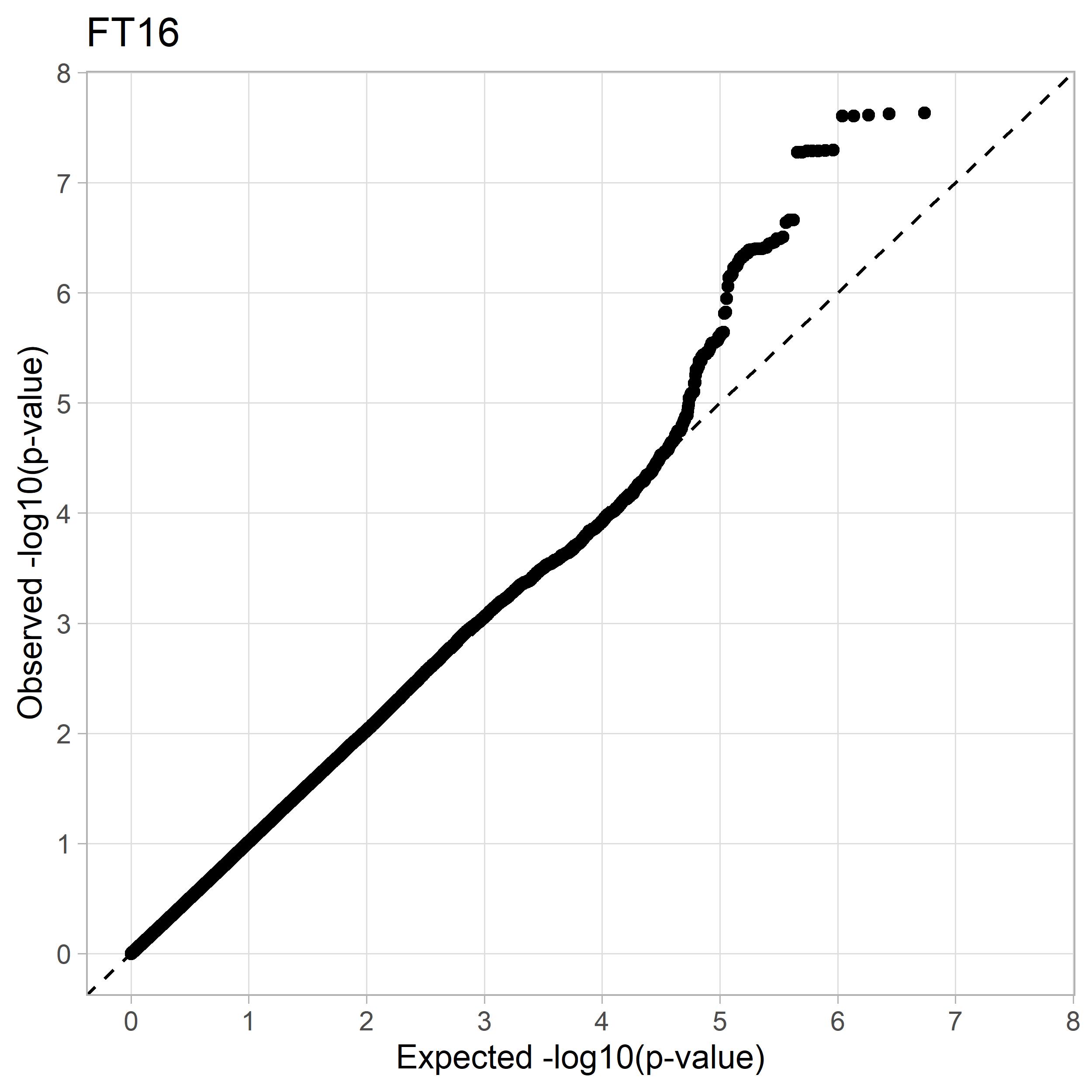


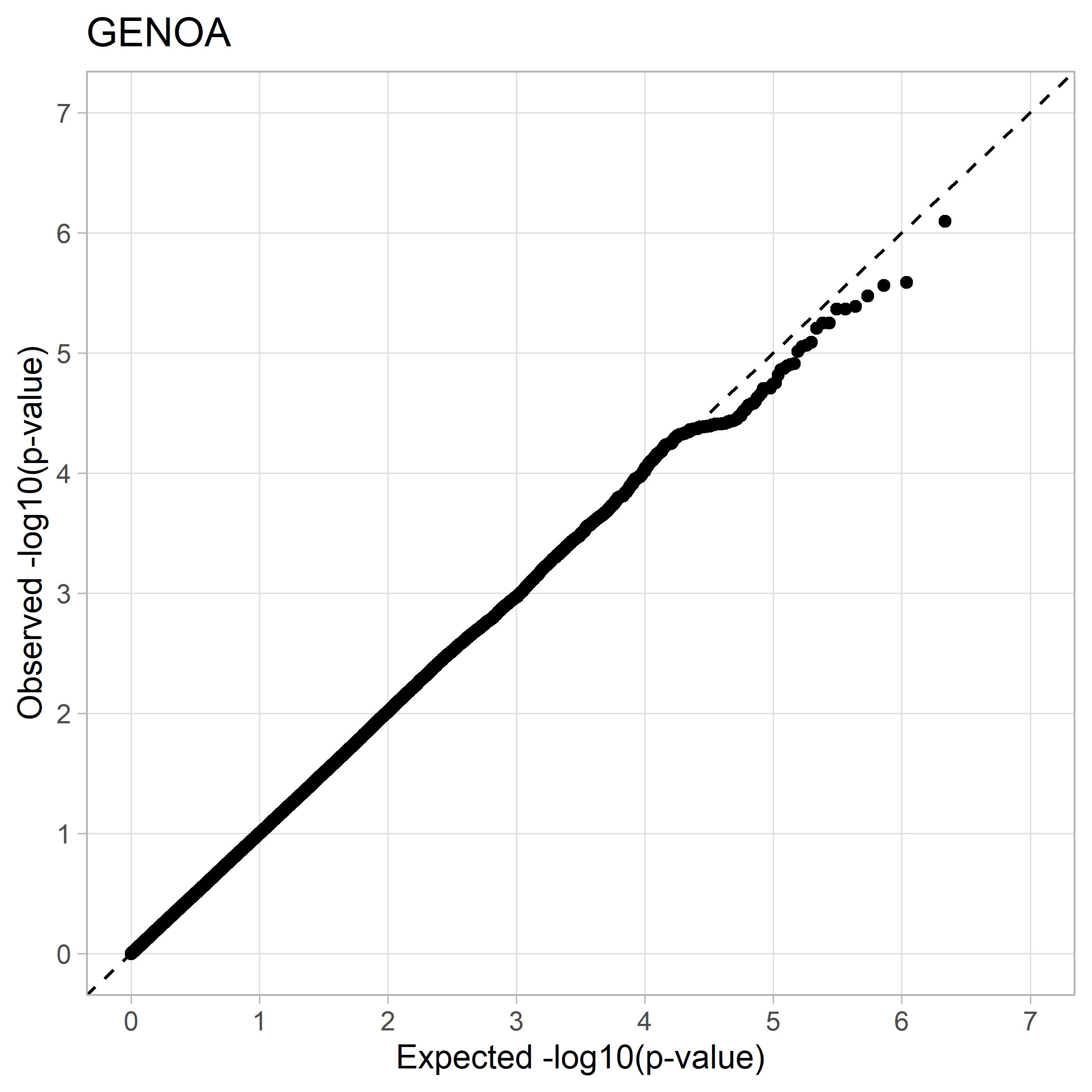


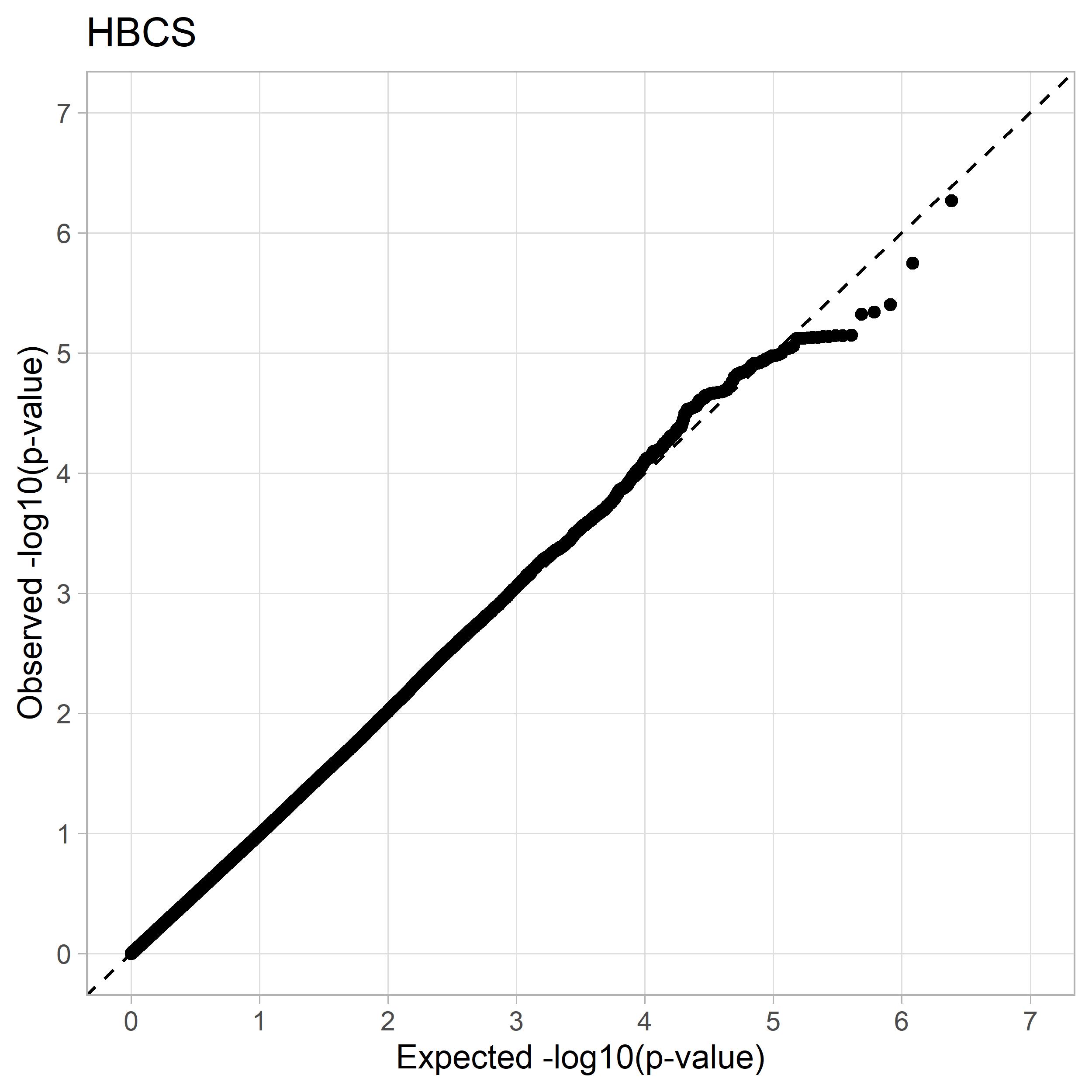


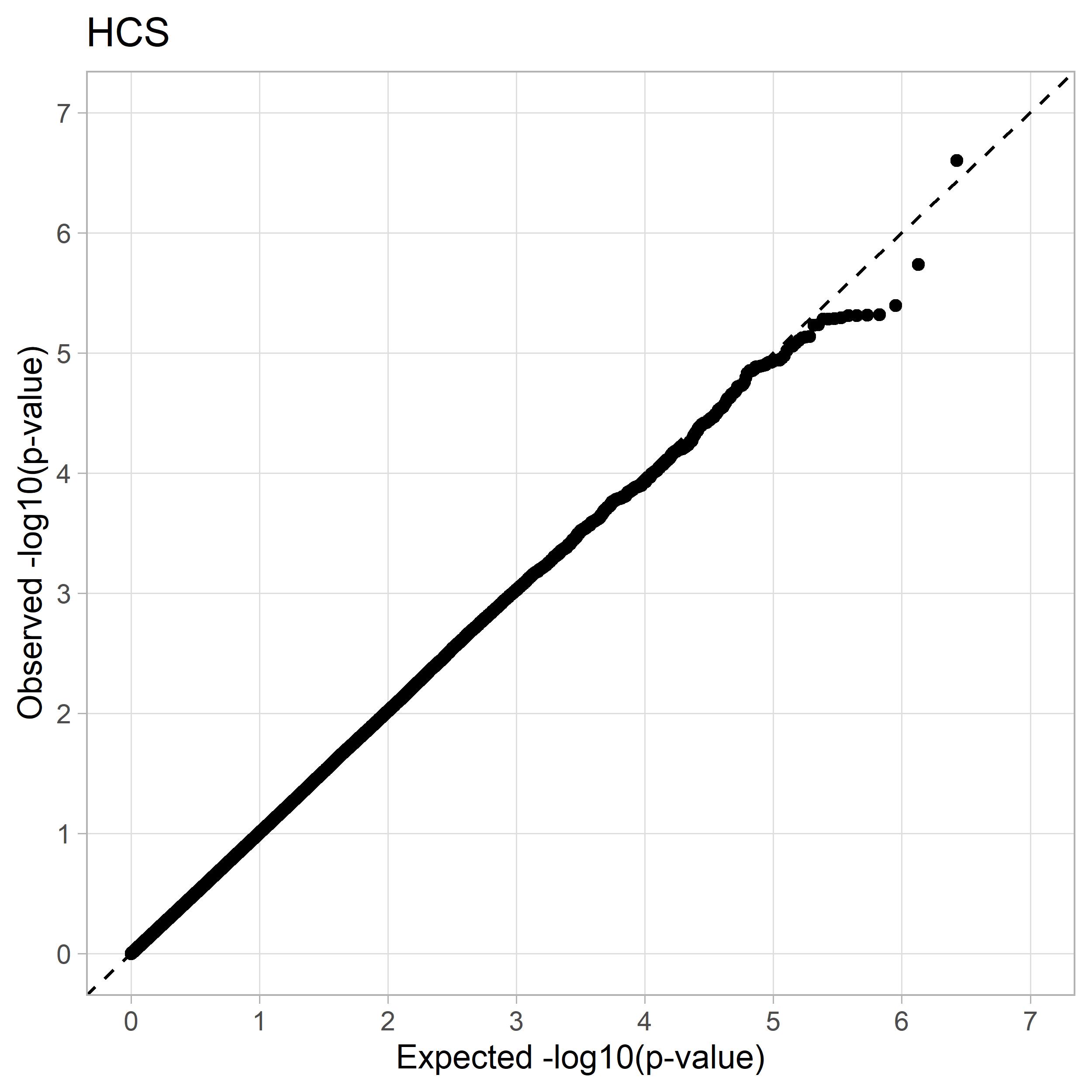


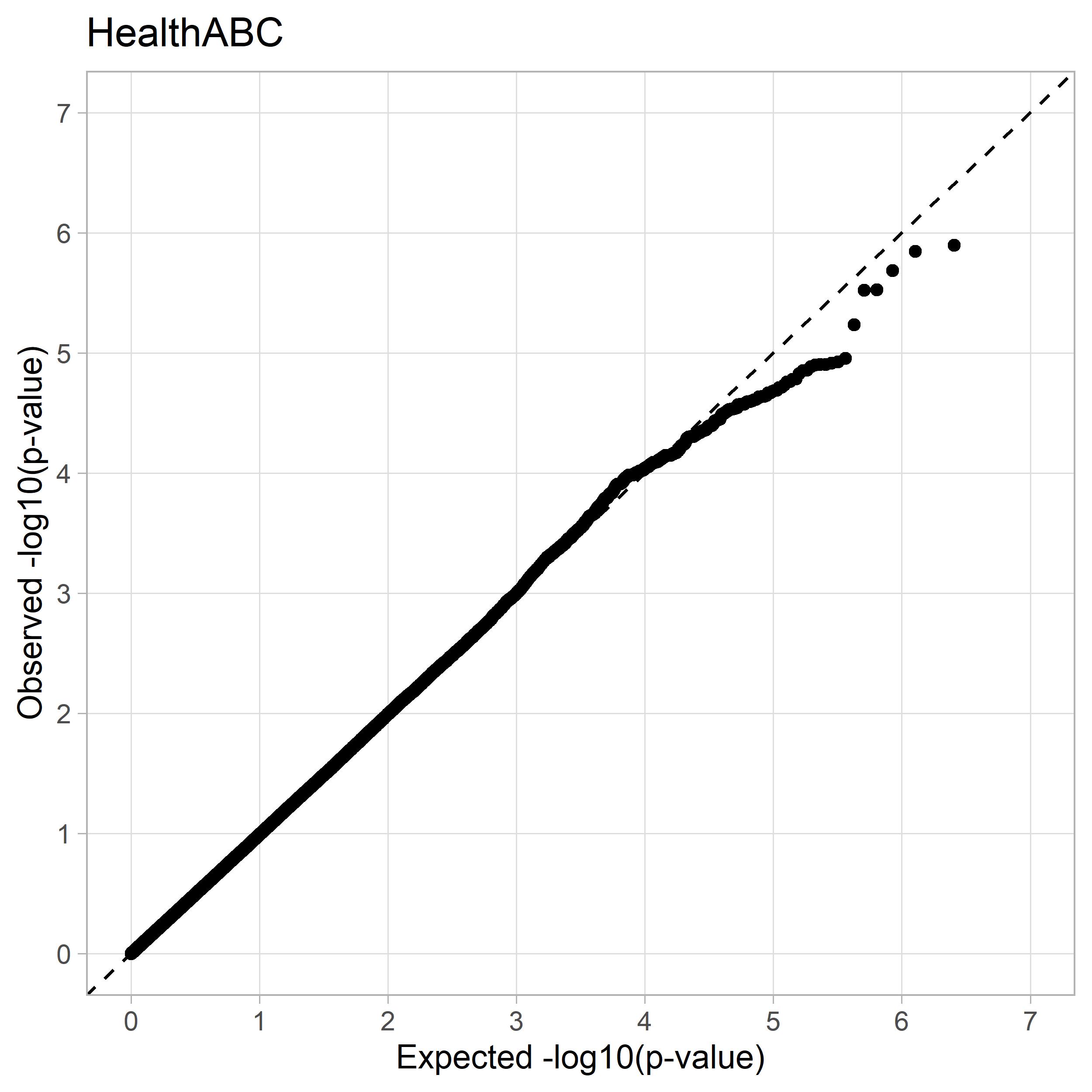


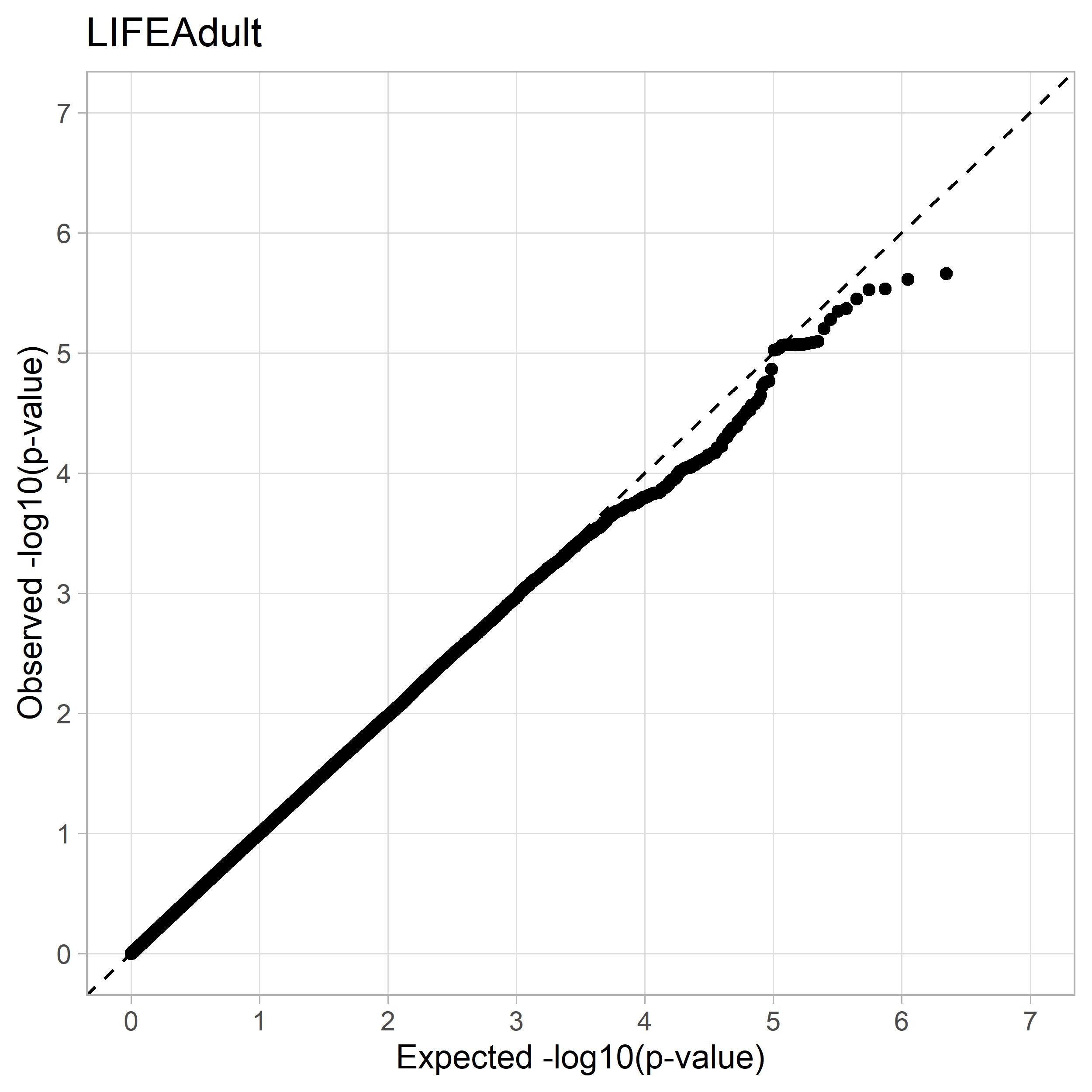


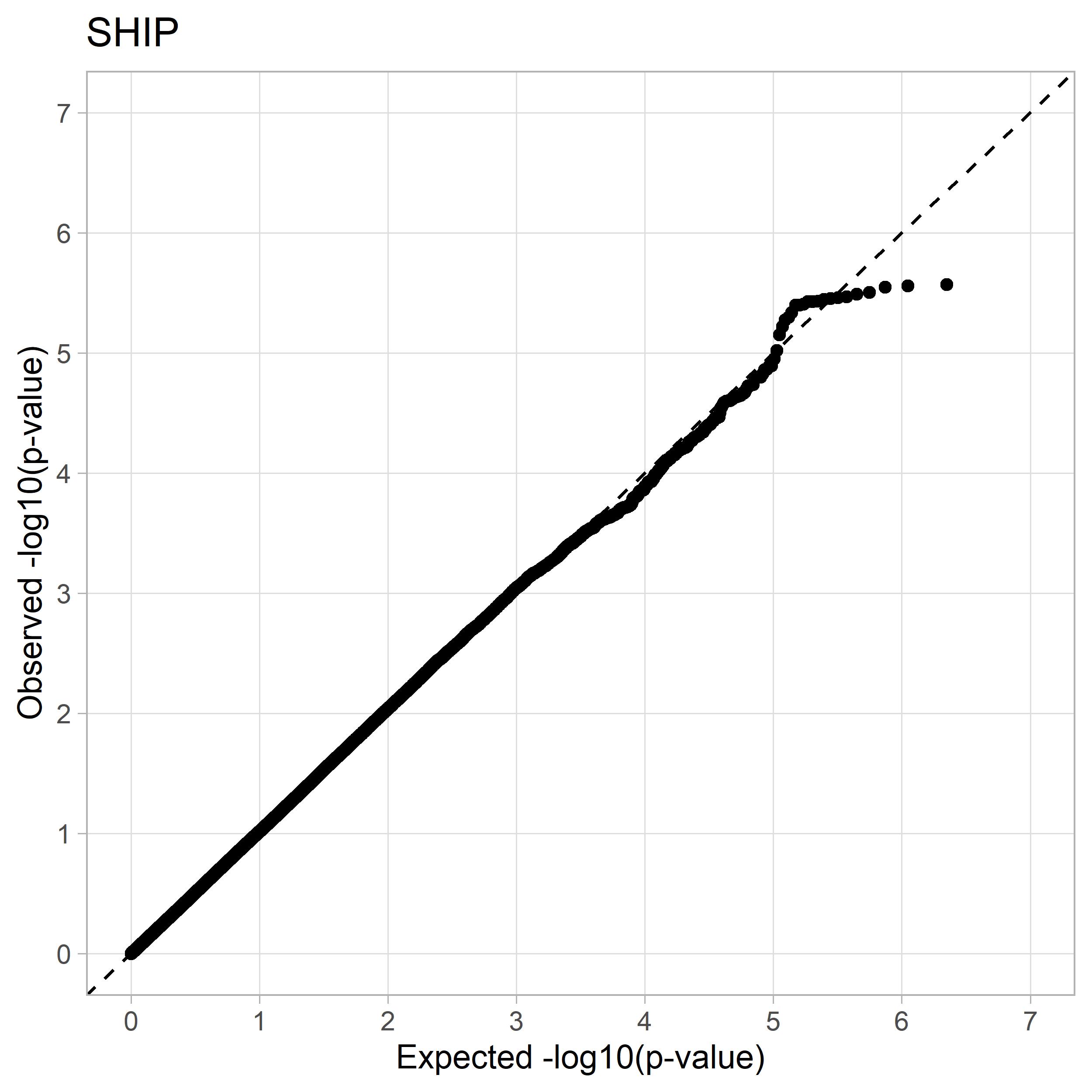


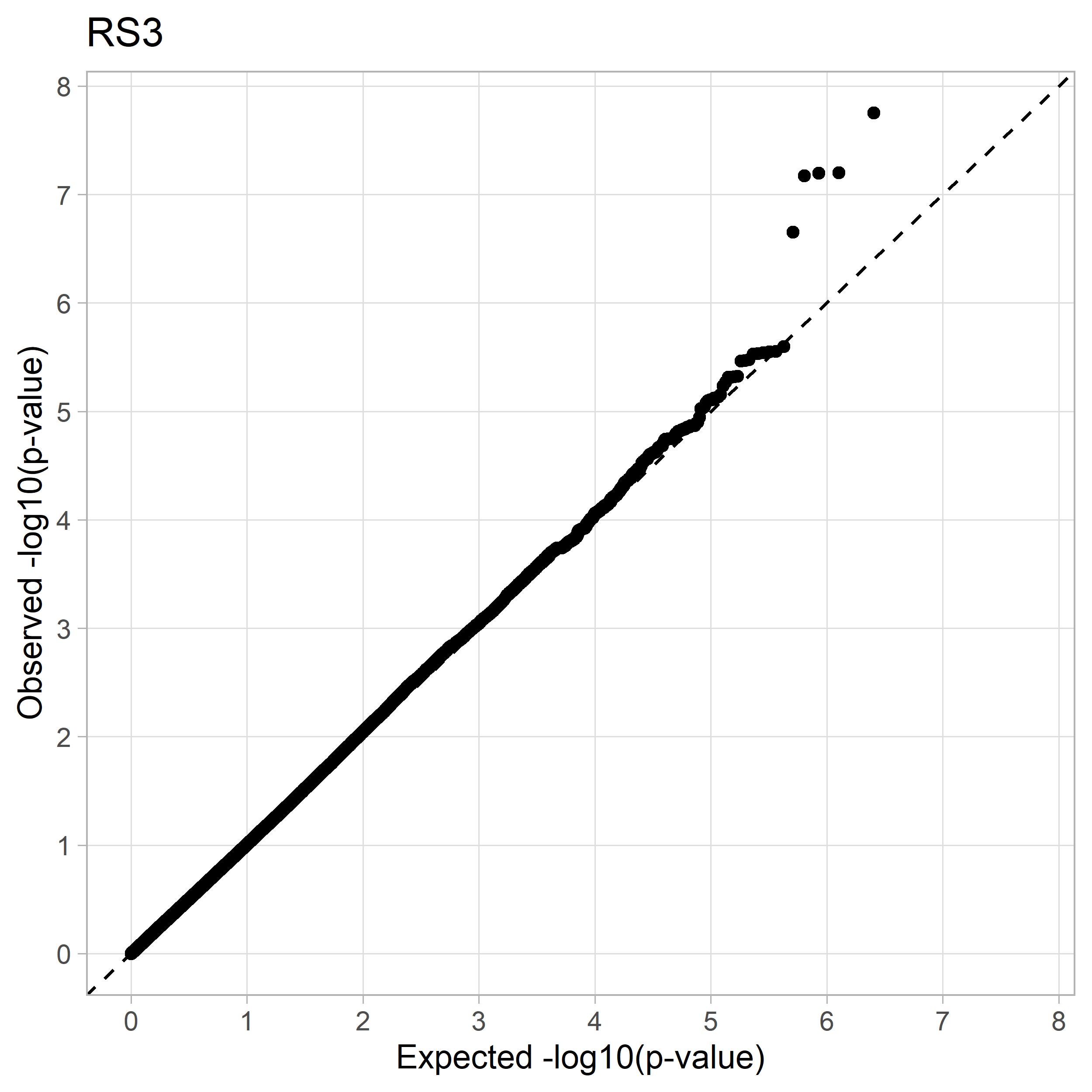


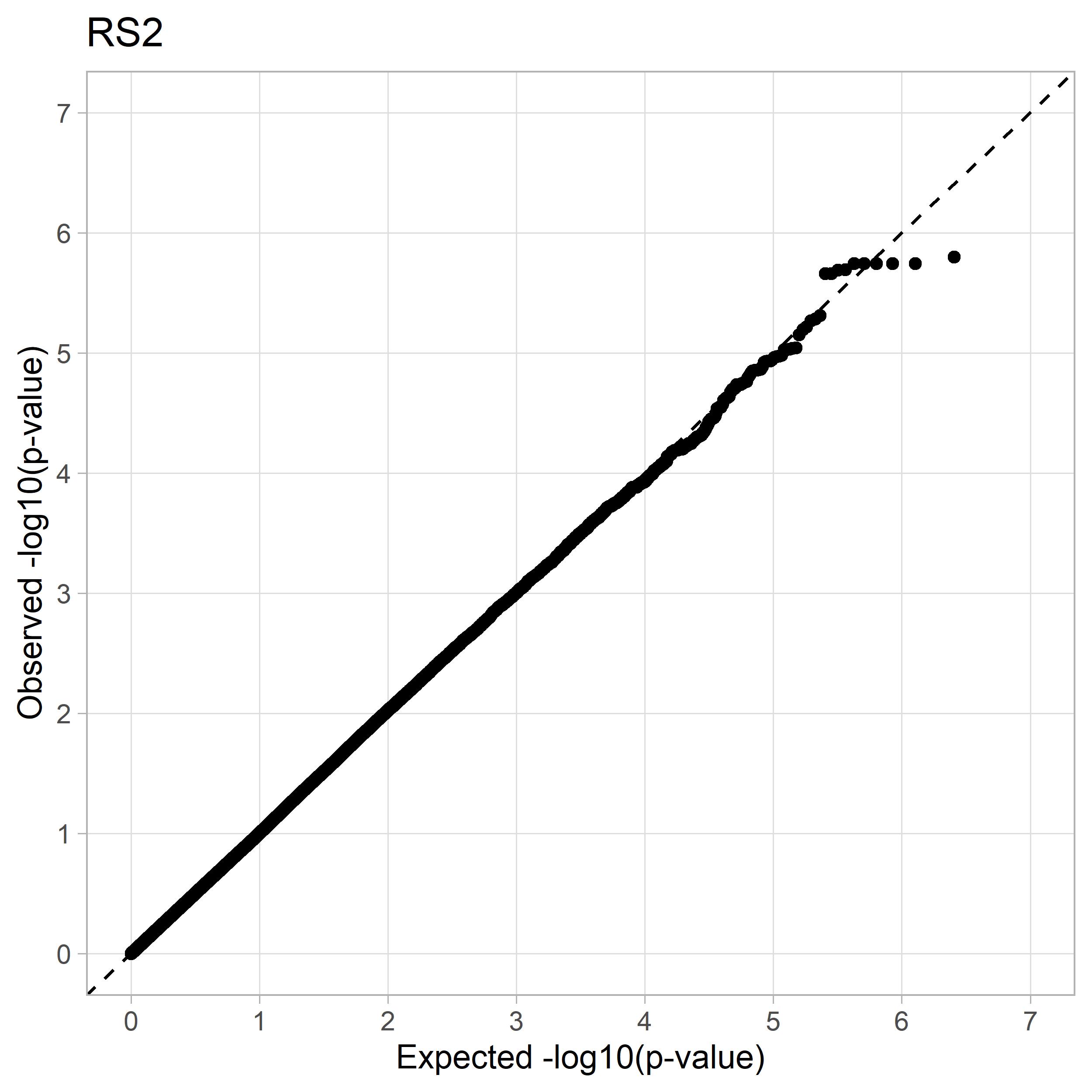


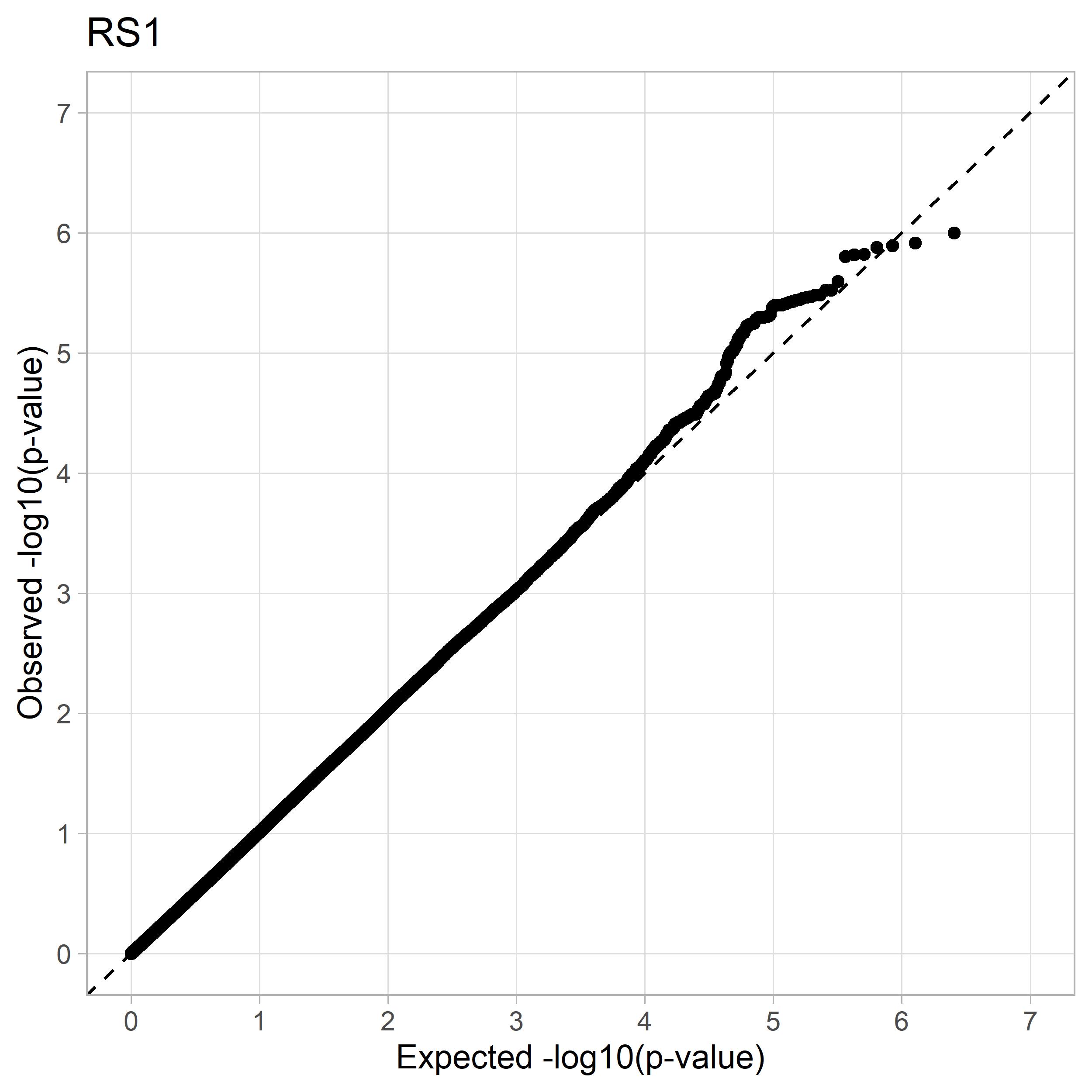


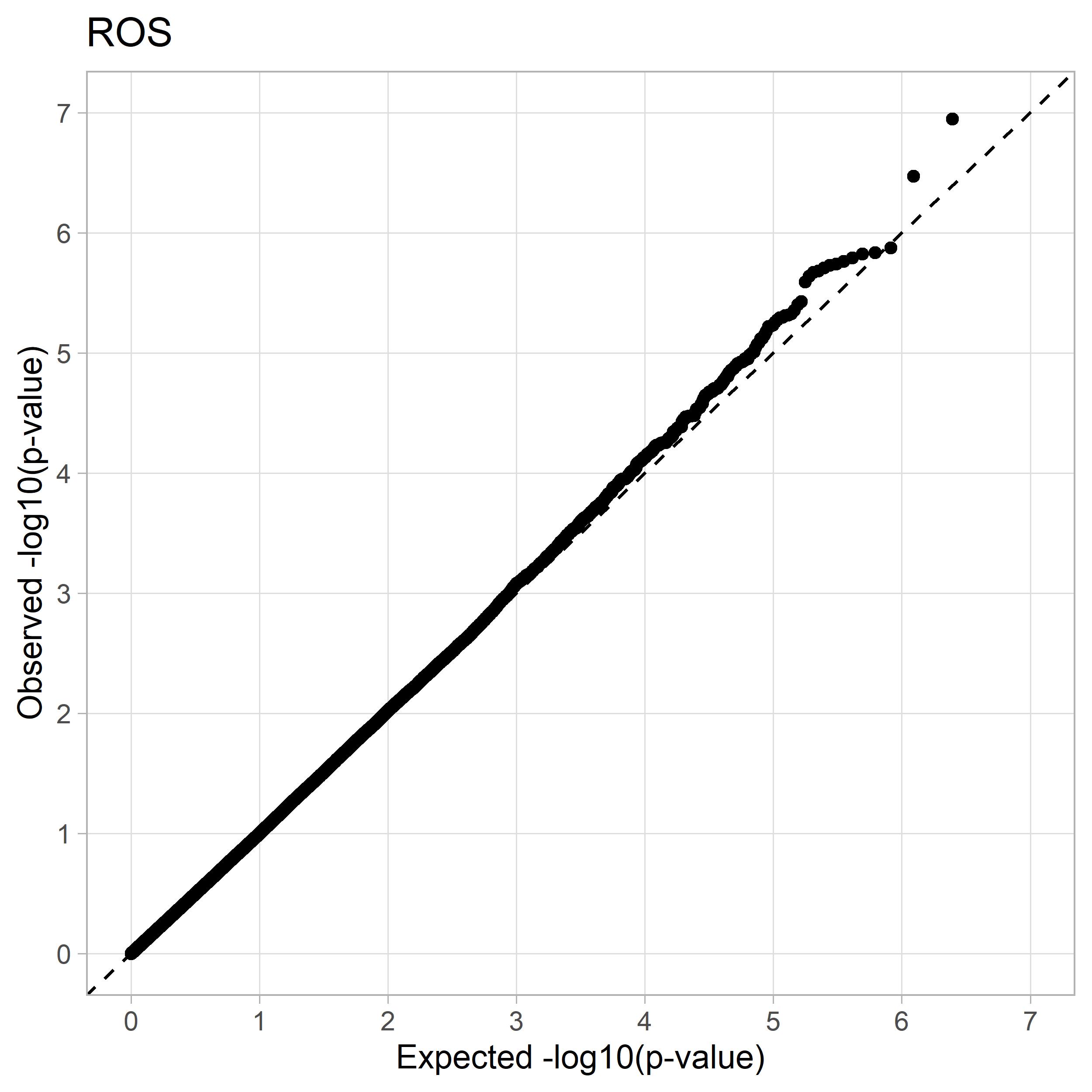


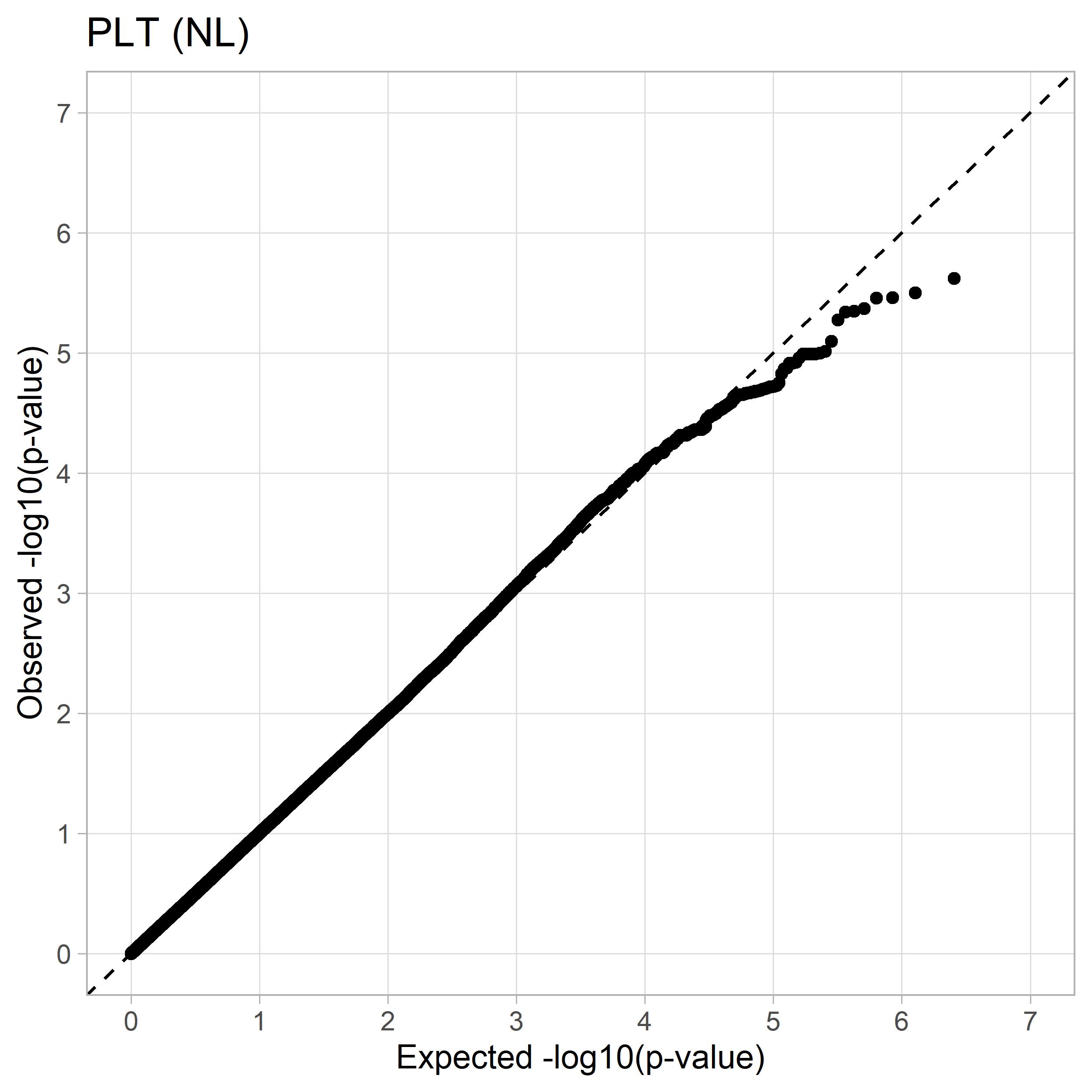


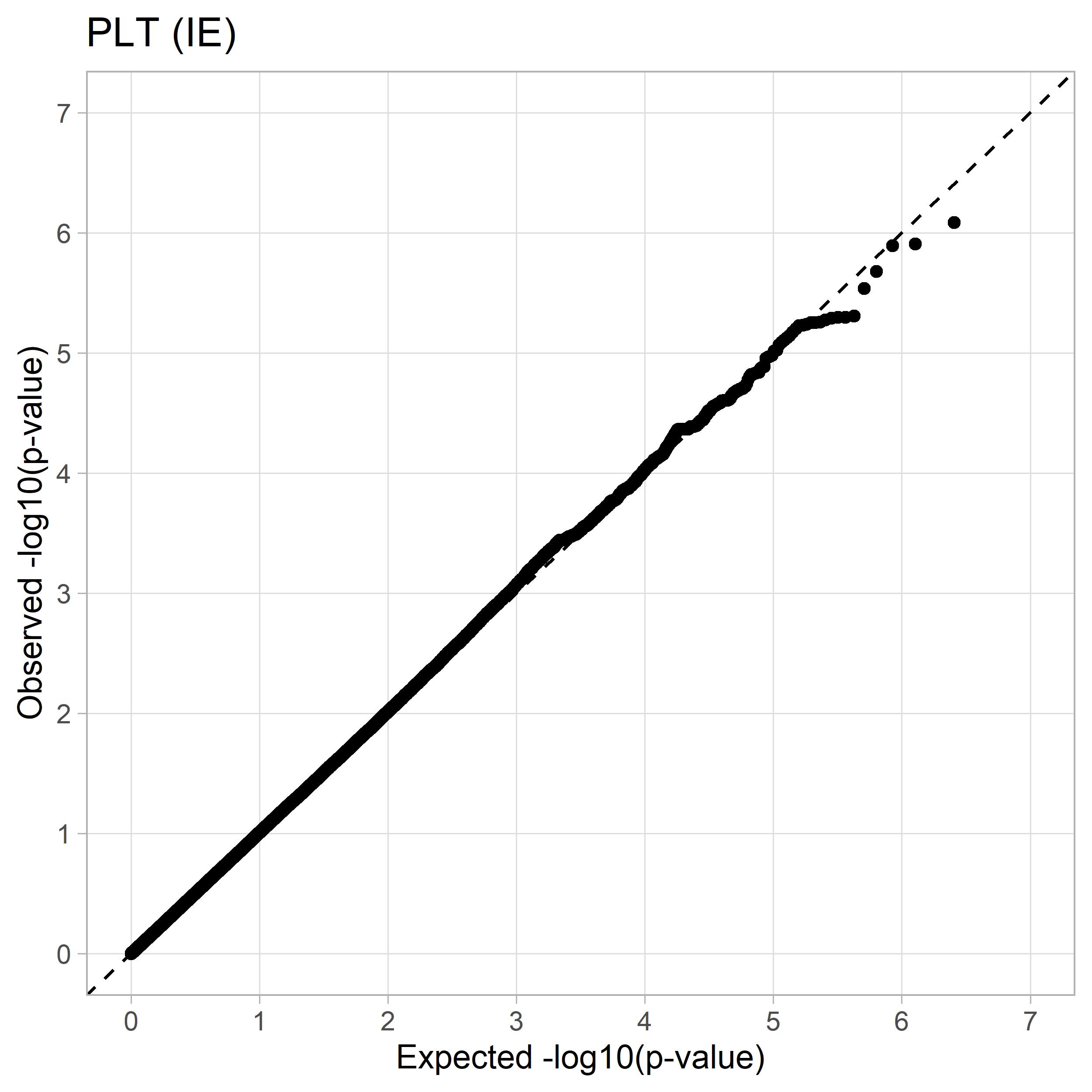


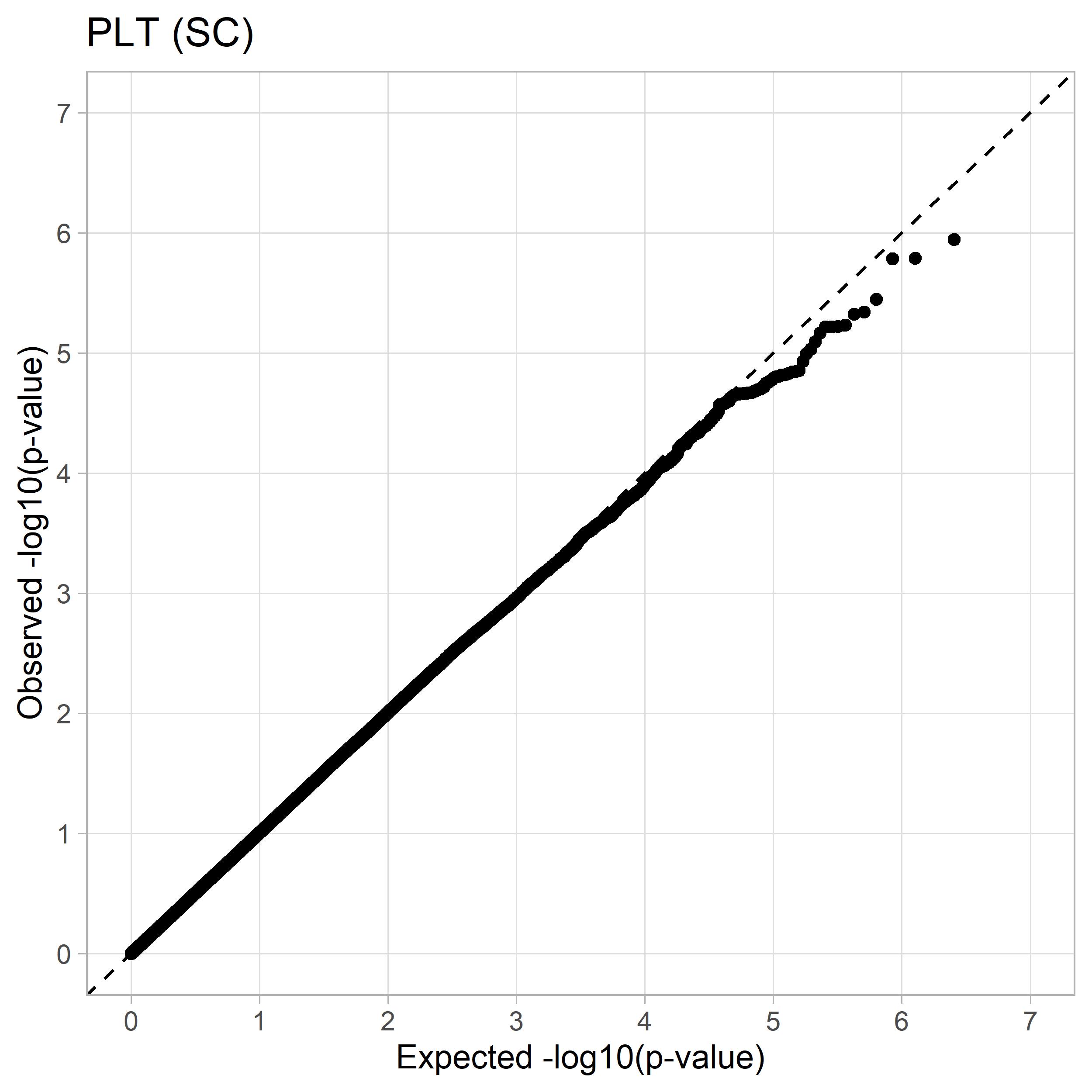

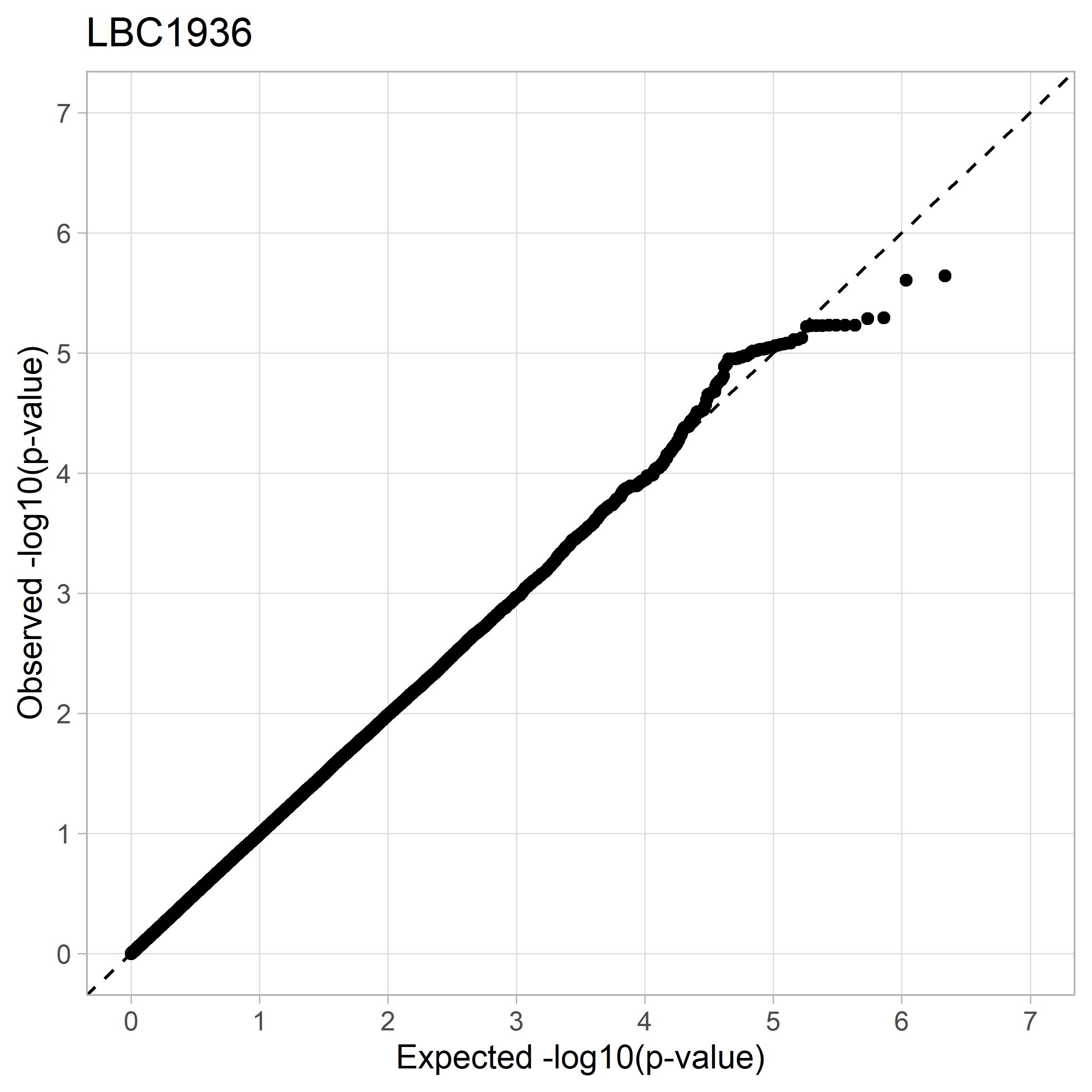

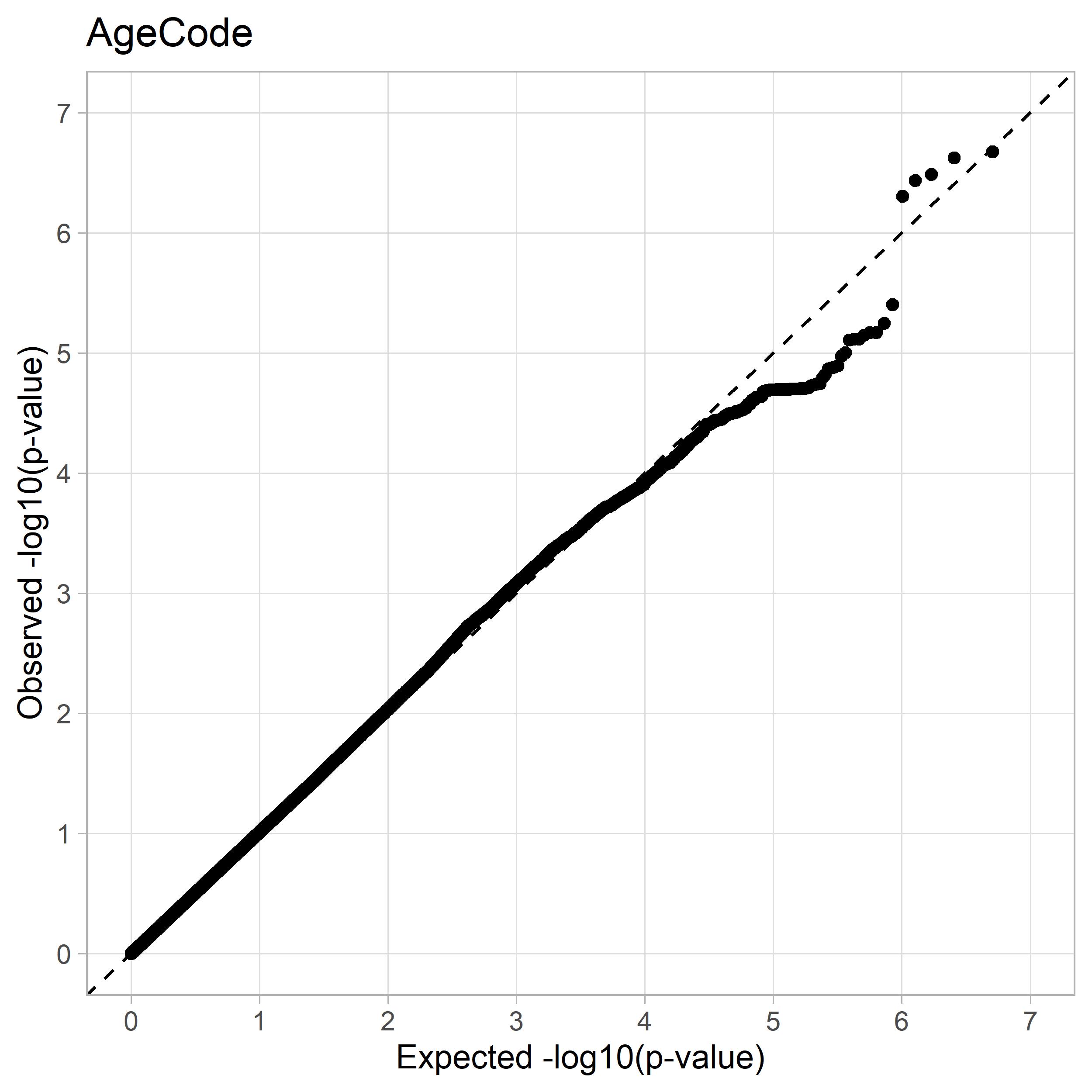


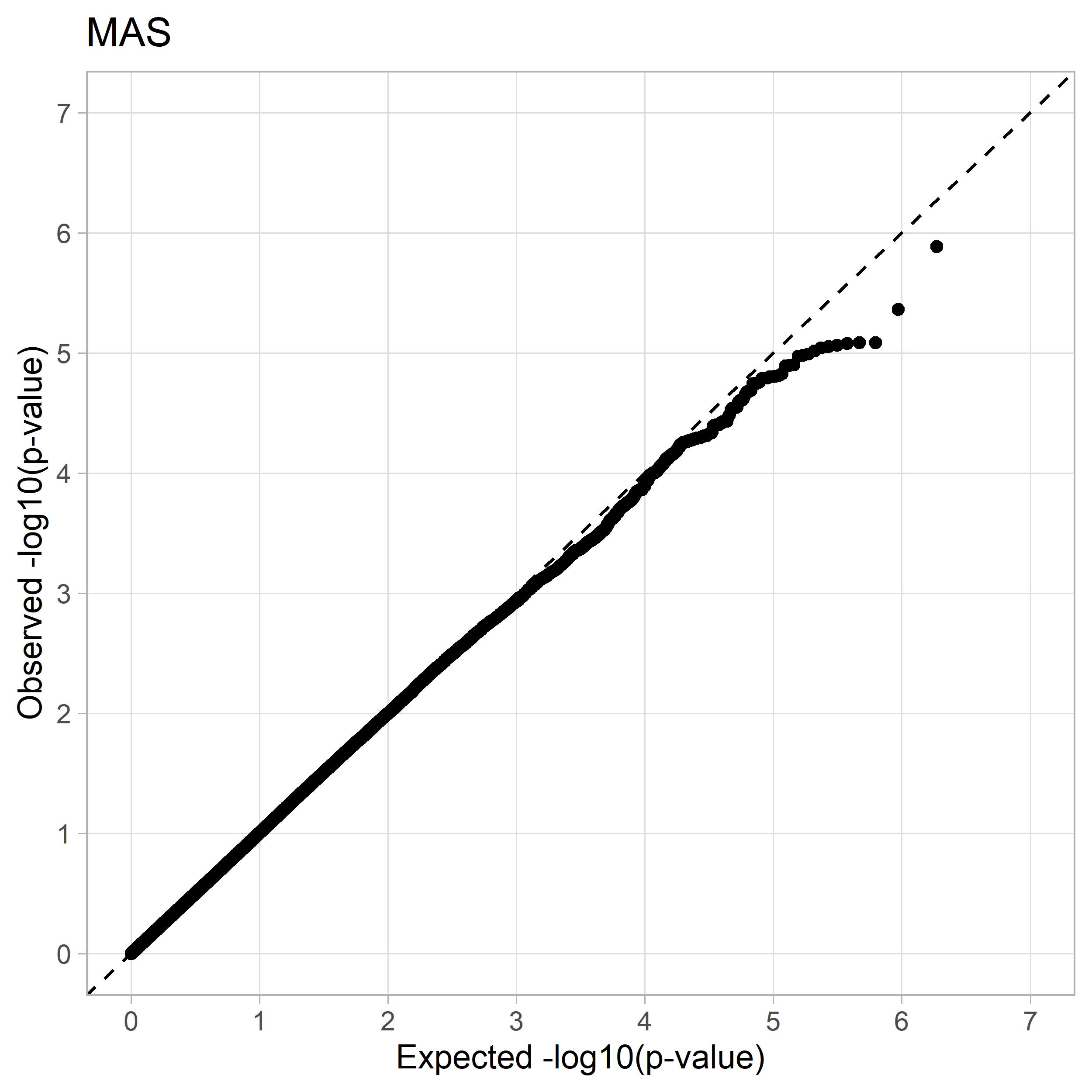

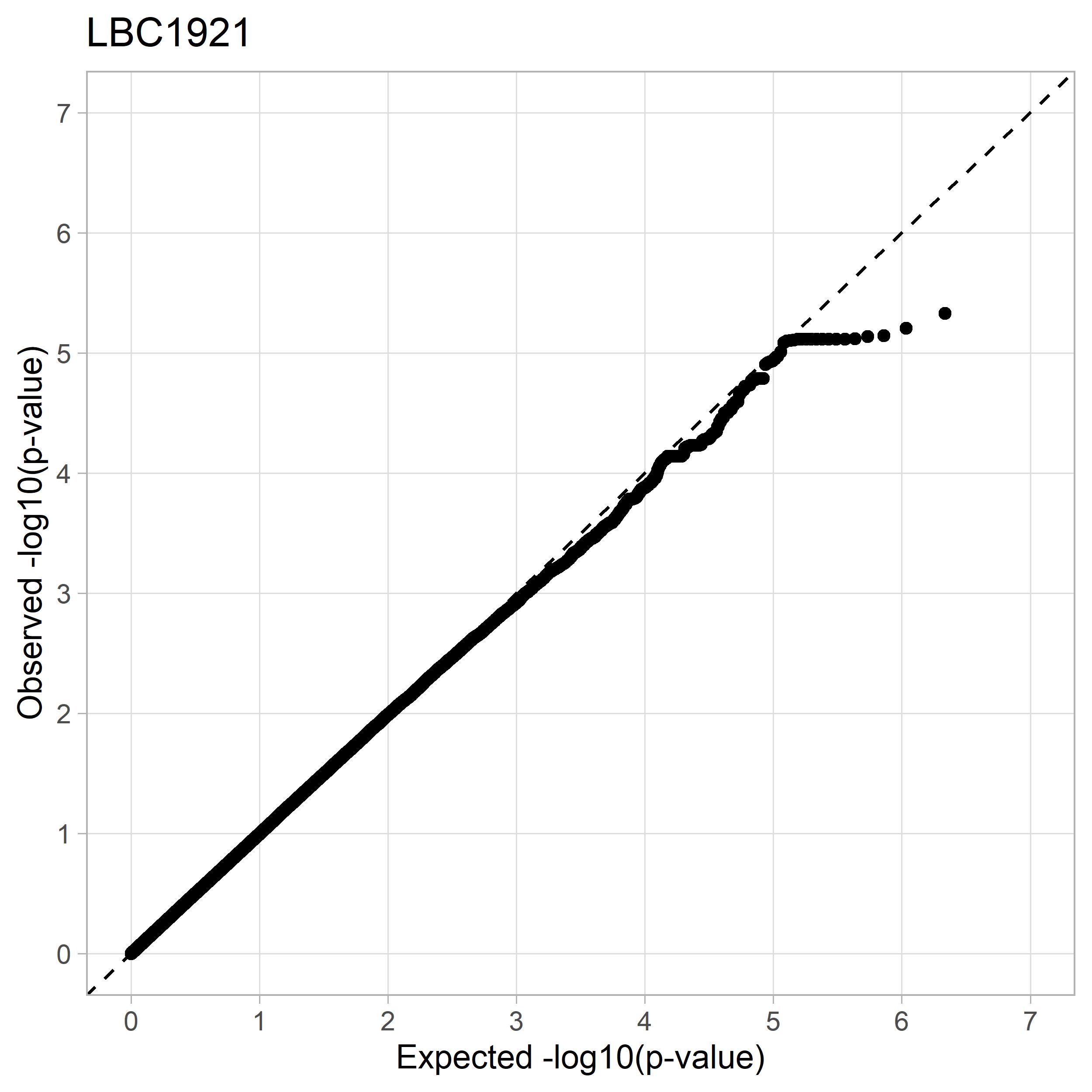

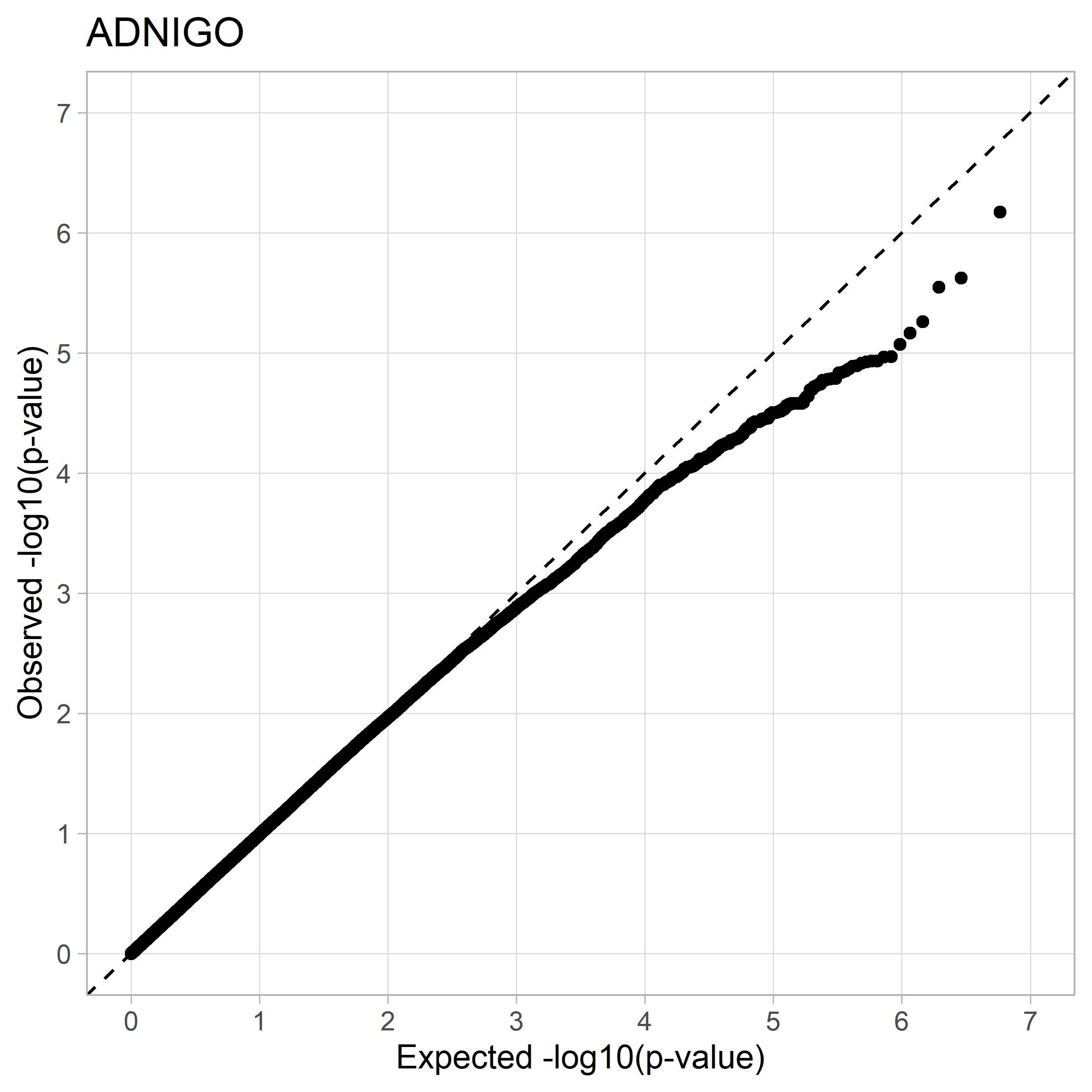


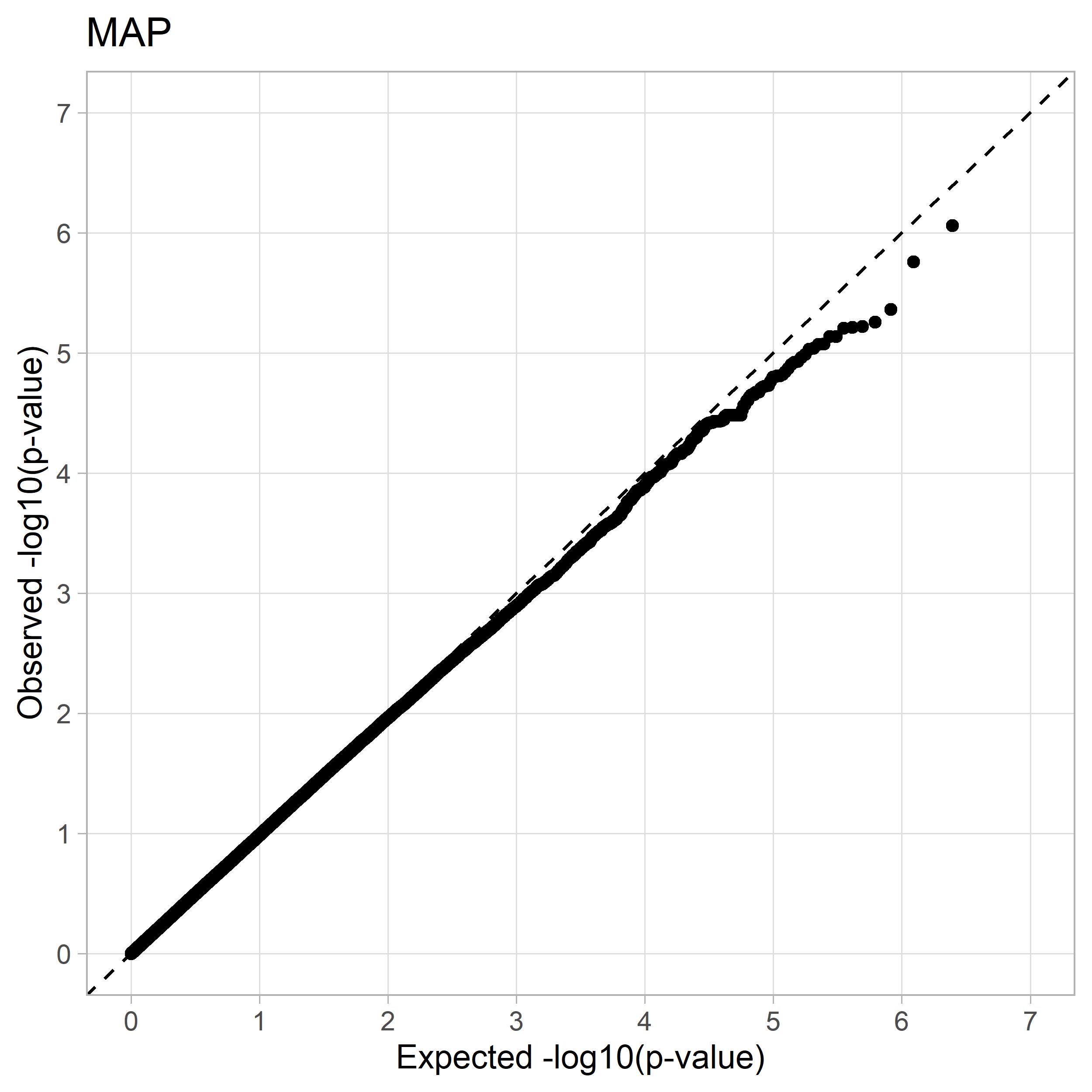

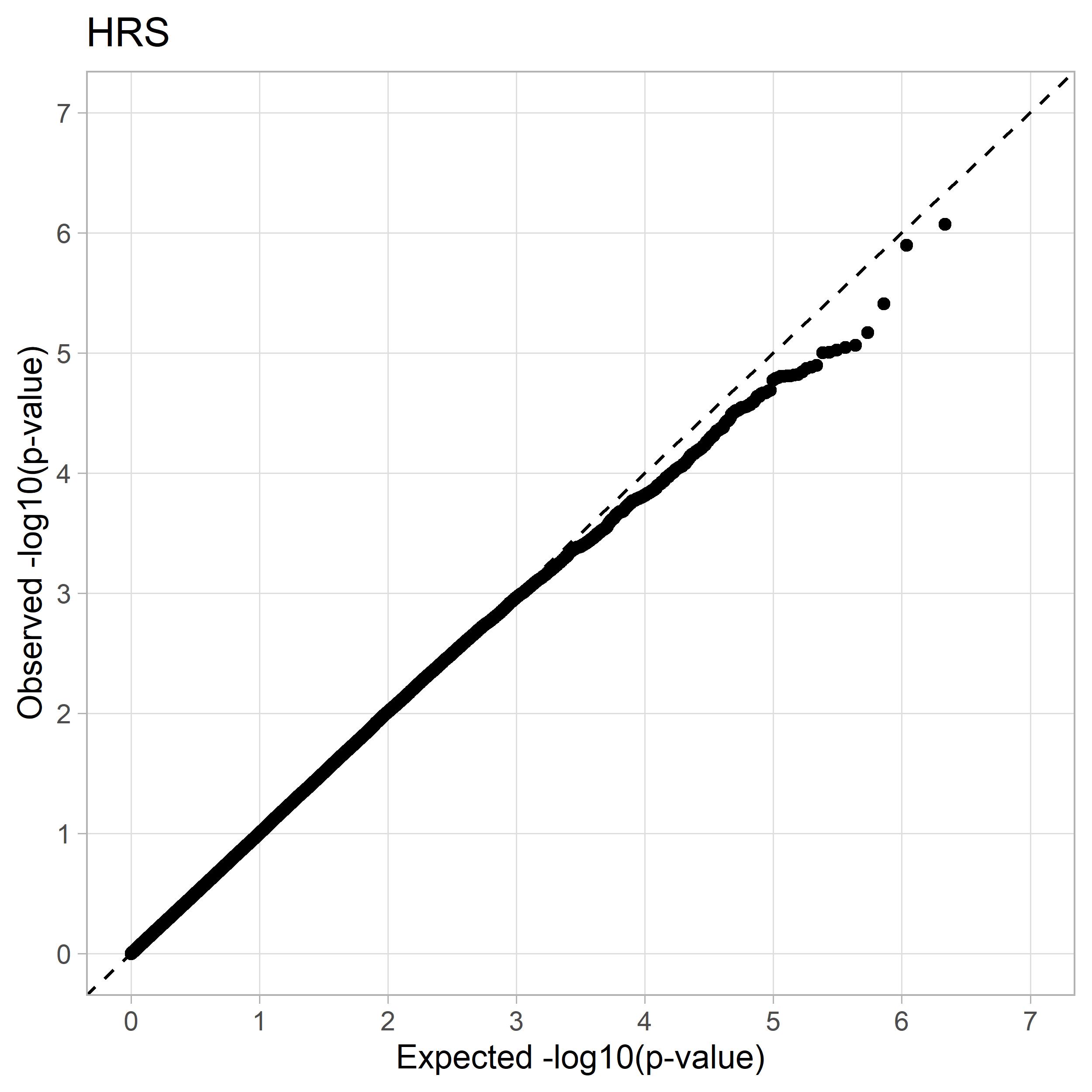

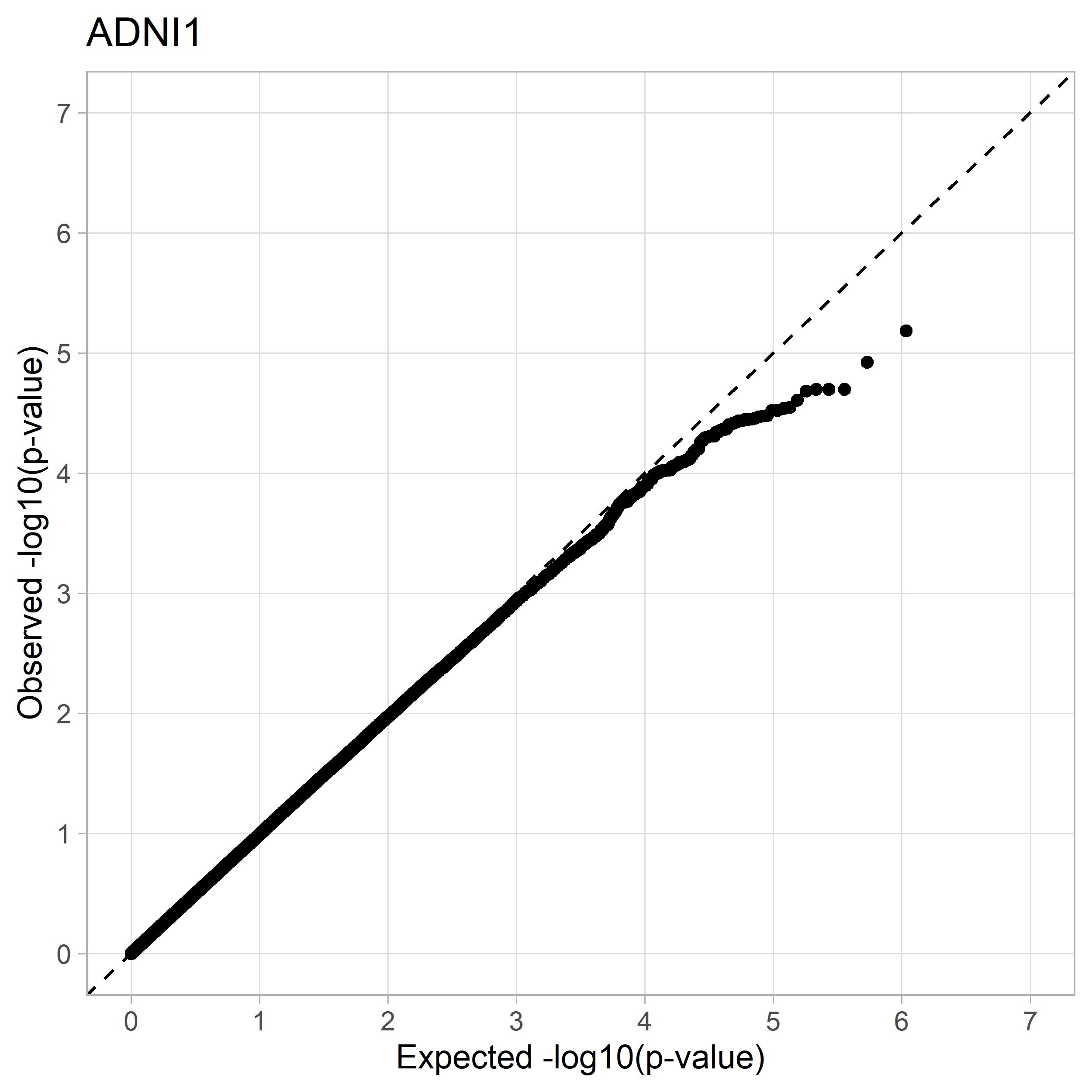


**Supplementary Figure 28:** QQ plots of cohort-level VL GWAS (Model 1) results (MAP to SHIP).
