# Supplementary material for: Genome-wide meta-analyses reveal novel loci for verbal short-term memory and learning
Source: Mol Psychiatry. 2022 Aug 16;27(11):4419–31. doi: 10.1038/s41380-022-01710-8 (PMC9734053; doi:10.1038/s41380-022-01710-8)
Supplement: Supplementary file 2 [file 41380_2022_1710_MOESM4_ESM.docx]

**Supplement 1: Earlier findings on genes implicated in this study, description of the cohorts, memory tests, and fMRI study procedures**

**Table of Contents**

EARLIER FINDINGS ON GENES IMPLICATED IN THIS STUDY	1

COHORT DESCRIPTIONS	3

ACKNOWLEDGEMENTS	16

VERBAL MEMORY TESTS	23

Word list tests with oral presentation	23

Word list tests with visual presentation	23

Paragraph recall tests	24

Visuo-spatial memory test in the UK Biobank	25

METHODS IN THE FUNCTIONAL ANALYSES	25

Dorsolateral prefrontal cortex methylation QTL analyses	25

In-vivo brain amyloid and Tau burden analyses	26

fMRI study sample and methods	27

REFERENCES	31

# EARLIER FINDINGS ON GENES IMPLICATED IN THIS STUDY

| **SNPs implicated in this study** | **Locus / genes** | **Role of the SNP/Gene in earlier studies** |
| --- | --- | --- |
| NA | 2p16.3 /*NRXN1* | *NRXN1* encodes for a cell adhesion molecule that regulates synapse formation and it has been implicated in range of neuropsychiatric outcomes^1,2^, general cognitive ability^3^, and sensorimotor performance^4^. In the PheWAS of UK Biobank samples (N~337.000), among the top phenotypes that are associated with *NRXN1* are death due to Neoplasm of uncertain behavior of brain (ICD10 D43.2 ; p=6.7x10-38), Malignant neoplasm of cerebrum (ICD10 C71.0 ; p=4.5x10-25), and Alzheimer’s disease (ICD10 G30.9 ; p=4.2x10-19)^5^. |
| rs4687625, rs2276816, rs2015971 | 3p21 / *POC1A,* *SMIM4,* *STAB1,* *PBRM1,* *NEK4,* *NT5DC2,* *ITIH4,* *GNL3,* *ITIH1,* *MUSTN1,* *GLT8D1, and* *ITIH3* | The locus has been implicated in General cognitive ability^6^ in the combined sample consisting of UK biobank, CHARGE cohorts and COGENT consortium cohorts (N=300,486) as well as schizophrenia^7,8^, bipolar disorder (BPD)^9^, major mood disorders^10,11^, and cross-disorder analyses ^12^ within the Psychiatric Genomics Consortium (PGC) and other samples. The top SNP in the cross-disorder GWAMA^12^, intronic *ITIH3* variant rs2535629, is in perfect LD with rs2240920 (D’ 1.0, r2: 0.91 in European populations) that was suggestively associated with VL (p=1.83^-6^) in our study. Intronic *ITIH4* variant rs4687658 was recently implicated as a shared risk factor for schizophrenia and smaller intracranial volume^13^. *ITIH4* and *GNL3* were also associated with amplitude of synchronous brain activity as assessed by electroencephalography (EEG) ^14^. Moreover, upregulation of the ITIH4 has been shown in Alzheimer’s disease^15^ A recent study with polyevidence scoring analyses integrated several lines of evidence based on genomic and expression data and the results pointed towards a role of *GLT8D1* in schizophrenia^16^. Another study identified *ITIH3/ITIH4* variant rs2535627, that was associated with schizophrenia^7^, as a sQTL that was significantly associated with an alternative exon skipping of *NEK4*^*17*^. Yet another study implicated *STAB1* as a primary candidate for BPD based on PGC BPD GWAMA findings and differences in gene-expression between euthymic and manic states^9^, whereas cross-disorder meta-analyses with combined BPD and ADHD found that 74 nominally significant variants were eQTLs for *NT5DC2*^18^. Interestingly, *TREX1* at 3p21.3 close to our locus has been implicated in rare vascular diseases characterized with neuropathology and cognitive impairment^19^.  In the PheWAS of UK Biobank samples (N~337.000), the top two phenotypes that are associated with synonymous *ITIH4* variant rs2276816 are Worrier/anxious feelings (p=8.8x10-6) and visuo-spatial memory (p=4.4x10-5). For both rs4687625 and rs2015971 top two phenotypes are standing height (p<2.5x10-10) and impedance of the whole body (p<8.2x10-10). Both SNPs also show suggestive association with fluid intelligence (p<2.6x10-5; N=108,818)^5^. |
| NA | 5p13.2 / AGXT2 | *AGXT2* may play a role in the regulation of the autonomic nervous system^20^. |
| rs425724 | 5p14.3 / *CDH18 and other cadherin genes* | Recent small family study implicated cell adhesion pathway, including *CDH18*, in familial early-onset dementia^21^. Several studies link cadherin genes in general with variety of neuropsychiatric disorders, such as ADHD, autism and intellectual disability^22^ and hippocampal volume loss over time^23^.  *CDH18* in particular has been implicated in trait depression^24^ and anecdotal evidence links deletion near *CDH18* with schizophrenia^25^. In the PheWAS of UK Biobank samples (N~337.000), the top phenotype that associated with *CDH18* is death due to Neoplasm of uncertain behavior of brain (ICD10 D43.2 ; p=3.3x10-53)^5^. |
| NA | 6q16.3 / *GRIK2* | *GRIK2* encodes the glutamate receptor 6, and deletions in this gene have been linked to intellectual disability^26^. In the PheWAS of UK Biobank samples (N~337.000), the top phenotype that is associated with *GRIK2*  is death due to Heartfailure (ICD10 I50.9 ; p=7.5x10-43)^5^ |
| NA | 7q11.22 / *CALN1* | *CALN1* has been linked with schizophrenia in a Han Chinese sample^8^. |
| rs9528369 | 13q21 | Linkage studies have identified 13q21 as a major susceptibility locus for language impairment^27^, language delay in autism^28^, and performance in nonword repetition test, a VSTM task with pseudowords, and endophenotype for verbal trait disorders ^29^. Moreover, recent GWAMA linked rs4497562 in this region with educational attainment (p=1.1x10-12)^30^. rs4497562 is ~396kB from rs9528369 and these SNPs are in LD (D’ 0.68, r2: 0.36 in European populations). In the PheWAS of UK Biobank samples (N=334.070), the top phenotype that showed association with rs9528369 is College/University degree (p=3.1x10-5)^5^. |
| rs4420638, rs6857 | 19q13.3 / *APOE, APOC1, TOMM40, PVRL2* | This locus encompasses all SNPs that were associated with verbal *long-term* memory after accounting for short-term memory and decline in verbal memory score after age 60 in the HRS cohort^31^. Functional analyses of 19q13.3 region showed that several *APOE* locus cis-regulatory elements influence both *APOE* and *TOMM40* promoter activity^32^. This locus has been repeatedly linked with general cognitive ability^33^, non-pathological cognitive decline^34-36^, and dementias^37,38^. Moreover, the top SNP in the most recent GWAMA of AD in this locus (rs42938)^37^ is in perfect LD with one of the lead SNPs of this study rs4420638  (D’: 1.0, r2: 0.7 in European populations). In the PheWAS of UK Biobank samples (N~337.000), the top two phenotypes that are associated with both rs4420638 and rs6857 are Alzheimer’s disease in the mother or in the father (p’s<2.2x10-171)^5^. |

# COHORT DESCRIPTIONS

**Alzheimer's Disease Neuroimaging Initiative (ADNI)**

All individuals included in these analyses were participants from the Alzheimer’s Disease Neuroimaging Initiative (ADNI)^23,39^. The initial phase (ADNI-1) was launched in 2003 to test whether serial magnetic resonance imaging (MRI), position emission tomography (PET), other biological markers, and clinical and neuropsychological assessment could be combined to measure the progression of MCI (mild cognitive impairment) and early Alzheimer’s disease (AD). The ADNI-1 participants were recruited from 59 sites across the U.S. and Canada and included approximately 200 cognitively normal older individuals (CN), 400 patients diagnosed with MCI, and 200 patients diagnosed with early probable AD aged 55-90 years. The ADNI-1 study has been extended to its subsequent phases (ADNI-GO/2) for follow-up for existing participants and additional new enrollments. Inclusion and exclusion criteria, clinical protocol, and other information about ADNI can be found at [www.adni-info.org](http://www.adni-info.org).  Demographic information, *APOE* and whole genome-wide genotypes, neuropsychological test scores, and diagnostic information are available from the ADNI data repository ([http://www.loni.usc.edu/ADNI/](http://www.loni.usc.edu/ADNI/)). ADNI samples were genotyped using Human 610-Quad (ADNI-1) and HumanOmni Express (ADNI-GO/2). After a standard quality control procedure for genetic markers and participants, imputation of un-genotyped SNPs was performed using MaCH (Markov Chain Haplotyping) software ^40^. Only non-Hispanic Caucasian participants were selected for this analysis by genetic clustering with CEU (Utah residents with Northern and Western European ancestry from the CEPH collection) and TSI (Tuscans in Italy) populations using HapMap 3 genotype data and multidimensional scaling (MDS) analysis ^41,42^. Among cognitively normal older participants with available cognitive tests and GWAS genotypes, 207 (ADNI-1) and 121 (ADNI-GO/2) non-Hispanic Caucasian participants were available for our GWAS analysis. Written informed consent was obtained at the time of enrollment that included permission for analysis and data sharing and consent forms were approved by each participating sites’ Institutional Review Board (IRB).

**Ageing, Cognition and Dementia in Primary Care Patients (AgeCoDe)**

The AgeCoDe study consists of 3.327 non-demented elderly subjects over 75 years which were randomly selected from the general-practice registry in six German cities (Bonn, Dusseldorf, Hamburg, Leipzig, Mannheim, and Munich) that were recruited from 2003 to 2004 ^43,44^. Inclusion criteria for patients were age 75 years and over, absence of dementia in the GP’s view and at least one contact with the GP within the last 12 months. Exclusion criteria were consultations only by home visits, residence in a nursing home, severe illness the GP would deem fatal within 3 months, insufficient facility in German, deafness or blindness, lacking ability to consent and not being a regular patient of the participating practice. The ethics committees of the participating centers approved the study.

Genotyping was performed in 782 patients in the Division of Molecular and Cognitive Neuroscience, Faculty of Psychology and Faculty of Medicine, University of Basel, using an Affymetrix 6.0 genotyping platform ^45^.
Among participants with genome-wide data, after exclusion of 9 participants with missing phenotype data and 13 participants with dementia, 760 participants were available for GWAS.

**Age, Gene/Environment Susceptibility (AGES)**

The AGES-Reykjavik Study is a single center prospective cohort study based on the Reykjavik Study. The Reykjavik Study was initiated in 1967 by the Icelandic Heart Association to study cardiovascular disease and risk factors. The cohort included men and women born between 1907 and 1935 who lived in Reykjavik at the 1967 baseline examination. Re-examination of surviving members of the cohort was initiated in 2002 as part of the AGES-Reykjavik Study. The AGES-Reykjavik Study is designed to investigate aging using a multifaceted comprehensive approach that includes detailed measures of brain function and structure. All cohort members were European Caucasians. Briefly, as part of a comprehensive examination, all participants answered a questionnaire, underwent a clinical examination and had blood drawn ^46^. All consenting participants were offered to take a neuropsychological test battery,^47^ including the California Verbal Learning Test (CVLT),^48^ to assess memory performance. Among participants with genome-wide data, after exclusion of 126 participants with dementia and 266 participants with a history of stroke, 2697 participants were available for a GWAS of word list learning (CVLT). 

**The Atherosclerosis Risk in Communities Study (ARIC)** 

     The ARIC study is a prospective population-based study of atherosclerosis and clinical atherosclerotic diseases in 15,792 men and women, including 11,478 white participants, drawn from 4 United States communities (Suburban Minneapolis, Minnesota; Washington County, Maryland; Forsyth County, North Carolina; and Jackson, Mississippi). In the first 3 communities, the sample reflects the demographic composition of the community. In Jackson, only black residents were enrolled.  Participants were between age 45 and 64 years at their baseline examination in 1987-1989 when blood was drawn for DNA extraction and participants consented to genetic testing ^49^.  Vascular risk factors and outcomes, including transient ischemic attack and stroke, were determined in a standard fashion ^50^. The Logical Memory I Test from the revised version of the Wechsler Memory Scale, (WMS-R)^51^ a test of immediate verbal memory, was administered to participants in the ARIC Brain MRI study (2004-2006)^52^ and the Atherosclerosis Risk in Communities Neurocognitive Study (ARIC-NCS) (2011-2013)^53^. There were 440 individuals in the discovery sample with no history of stroke whose Logical Memory I scores were obtained in the ARIC Brain MRI study.  There were 3,528 individuals from the ARIC-NCS study whose test scores were available for analysis in the replication sample after excluding participants in the discovery sample, as well as those with prevalent stroke and possible prevalent dementia based on a score < 21 on the Mini Mental State Examination^54^. 

**The Coronary Artery Risk Development in Young Adults Study (CARDIA)**

CARDIA is a longitudinal study of 5,115 African-Americans and white adults who were age 18-30 at the baseline examination in 1985-86 ^55^. Participants have been followed at 2-5 years intervals over 30 years. Cognitive function was first assessed at the year 25 examination (n=3393). The present analysis focuses on the Rey Auditory Verbal Learning Test (RAVLT), which assesses the ability to memorize and to retrieve words (verbal memory) ^56^.  The RAVLT consisted of 5 presentations of a 15‐item word list (List A) with recall, one presentation of a 15‐item Interference List (List B) with recall, a Short Delay Free Recall of List A (Trial 6) and a Long Delay Free Recall of list A (Trial 7). The 15 words were read slowly to the subject, requesting him/her to repeat them after the reading, independently from the order they were said. The same procedure was repeated in steps A2 (i.e. second recall of list A), A3, A4 and A5, pointing out that the subject had to remember all the words, including those said previously. Then a second list of words (List B) was read as a distracter, and the subject was asked to recall these new words. After that, the words of the first list were asked (Trial 6, Recall after Interference), without being exposed, in a task of immediate recall from episodic memory. Twenty-five minutes after this stage the subject was asked to recall the words of the first list (Trial 7, Delayed Recall) in order to assess the delayed recall of episodic memory. The average score in the first learning steps (Steps A1 to A5) was computed to give the Immediate Rey score (sum of all words recalled in the five tests divided by five), the score of trial 6 provided the score for Recall after Interference and the score of Trial 7 provided the score for Delayed Recall.  The Immediate Recall and Delayed Recall scores were used in the analyses.

Genotyping was performed at the Broad Institute using the Affymetrix Genome-Wide Human SNP Array 6.0 (Santa Clara, California). Participants of African descent were genotype as part of the Candidate Gene Association Resource (CARe) project; those of European descent were genotyped as part of the Gene Environment Association Studies Initiative (GENEVA). Genotyping was completed for 903 African-American and 1720 white individuals with a sample call rate ≥ 98%. A total of 578,568 SNPs passed quality control (MAF ≥ 2%, call rate ≥ 95%, HWE ≥ 10-4) and were used for imputation to the 1000 Genomes reference panel. Individuals with both phenotype and genotype data included in these analyses are N=709 African-Americans and N=1403 whites.

**The Cardiovascular Health Study (CHS)**

The Cardiovascular Health Study (CHS) is a population-based cohort study of risk factors for coronary heart disease and stroke in adults ≥65 years conducted across four field centers ^57^. The original predominantly European ancestry cohort of 5,201 persons was recruited in 1989-1990 from random samples of the Medicare eligibility lists; subsequently, an additional predominantly African-American cohort of 687 persons was enrolled for a total sample of 5,888. DNA was extracted from blood samples drawn on all participants at their baseline examination in 1989-90. In 2007-2008, genotyping was performed at the General Clinical Research Center's Phenotyping/Genotyping Laboratory at Cedars-Sinai using the Illumina 370CNV BeadChip system on 3980 CHS participants who were free of CVD at baseline, consented to genetic testing, and had DNA available for genotyping.

Vascular risk factors and outcomes, including transient ischemic attack, stroke and dementia, were determined in a standard fashion ^58,59^. As part of the CHS Cognition Study,^59,60^ in 1992-94 (WMS-R) and again, in 1997-99 (CVLT), participants were invited to undergo and detailed neuropsychological assessment including the CVLT(4) and the logical memory test from the WMS-R^51,61^.

Because most other cohorts were predominantly European ancestry, the African American participants were excluded from this analysis to limit the potential for false positive associations due to population stratification. To date, genotyping has been successful among 3,271 of 3,373 European ancestry participants on whom genotyping was attempted. Among participants with genome-wide data, after exclusion of participants with dementia or a history of stroke, 330 participants were available a GWAS of word list learning (CVLT) and 618 participants were available for a GWAS of immediate paragraph recall (WMS-R).

**Duke Neurogenetics Study (DNS)**

Participants were recruited as part of the Duke Neurogenetics Study (DNS), an ongoing study investigating biological mechanisms of individual differences in brain function and behavior. Informed consent was obtained for all subjects as approved by the Duke University School of Medicine Institutional Review Board. All participants were healthy, young adult volunteers free of the following exclusion criteria included: (1) medical diagnoses of cancer, stroke, head injury with loss of consciousness, untreated migraine headaches, diabetes requiring insulin treatment, chronic kidney or liver disease, or lifetime history of psychotic symptoms; (2) use of psychotropic, glucocorticoid, or hypolipidemic medication; and (3) conditions affecting cerebral blood flow and metabolism (e.g. hypertension). The DNS seeks to establish broad variability in multiple behavioral phenotypes related to psychopathology, so participants were not excluded based on diagnosis of any past or current DSM-IV Axis I or Axis II disorder. No subjects were taking psychotropic medication at the time or at least 10 days prior to study participation. DNA was iolated from saliva derived from Oragene DNA self-collection kits (DNA Genotek) customized for 23andMe (www.23andme.com). DNA extraction and genotyping were performed through 23andMe by the National Genomics Institute (NGI), a CLIA-certified clinical laboratory and subsidiary of Laboratory Corporation of America. One of two different Illumina arrays with custom content was used to provide genome-wide SNP data: the HumanOmniExpress or HumanOmniExpress-24.

**Erasmus Rucphen Family study (ERF)**

The Erasmus Rucphen Family (ERF) study is a family-based cohort study in a genetically isolated population in the Netherlands,^62,63^ including 3,000 participants. Participants are all descendants of a limited number of founders living in the 19th century. Extensive genealogical data is available for this population. The study protocol included venous puncture for DNA isolation and chemistry, cognitive evaluation, cardiovascular examination, eye assessments and body composition measurements. All participants gave informed consent and the study was approved by the medical ethics committee at Erasmus MC University Medical Center. Genotyping was done at the Human Genotyping Facility, Genetic Laboratory Department of Internal Medicine, Erasmus MC, Rotterdam, and at the Genotyping Center of Leiden University, The Netherlands. In total, 2,385 samples from the ERF Study were available with good quality genotyping data. Participants were invited to undergo a neuropsychological evaluation,^64^ which included the Dutch version of Rey’s Auditory Verbal Learning Test (RAVLT)^65,66^. Among participants with genome-wide data, we excluded 21 individuals with a history of stroke. The remaining 2724 participants were available for performance GWAS of word list learning (RAVLT). 

**The Framingham Heart Study (FHS1, FHS2): Offspring Cohort**

The FHS is a community-based longitudinal cohort study that was initiated in 1948 with an enrollment of the Original cohort of 5209 participants in the town of Framingham, MA, USA. Biennial examinations including standardized interviews, physician examinations, and laboratory testing have been performed on these subjects since the enrollment. In 1971, 5124 adult children of the Original cohort and spouses of these children, the Offspring cohort, were enrolled and underwent examination approximately every 4 years. Beginning in 2002, the children of the Offspring cohort, Generation 3, were enrolled and examined every 7 years.    Rigorous tracking of cognitive function was added in 1975. Participants who attended an examination between 1997 and 2001(Original cohort examination 25 or 26, and Offspring cohort examination 7) were invited to return for a brain MRI study at which time they were also administered a 35-45 minute neuropsychological test battery. The study sample (FHS1) consisted of 2110 genotyped offspring participants who were free of dementia and stroke and who were administered the neuropsychological battery between 1999 and 2005. Logical Memory was assessed using the Wechsler Memory Scale III-UK (WMS-IIIUK)^67^. The study sample of FHS2 consists of 3067 genotyped Gen3 participants completed the second exam cycle in 2008-2010, which included a cognitive screening. The CERAD word list task and Victoria Stroop Test were administered using standard administration procedures. Written informed consent to genetic research has been obtained on all individuals included in this study. Ethics permission for FHS and genetic research in FHS was obtained from the Institutional Review Board of Boston University Medical Campus (IRB number H-32132, H-26671)

**FinnTwin16 (FT16)**

The FinnTwin16 study is a longitudinal study of twin pairs born 1975-1979, with five waves of data collection at ages 16, 17, 18.5, young adulthood (mid-twenties) and mid-thirties ^68^. After the young adult survey, twin pairs concordant and discordant for alcohol use were invited to an in-person testing; 602 twins participated. A blood sample was taken for DNA analyses, and genotyping was done on Illumina gwas chips. An extensive neuropsychological test battery was administered ^69^. Verbal memory was studied with the California Verbal Learning Test (CVLT)^48^. CVLT was administered to 560 participants. The study was approved by the Institutional Review Board of Indiana University and the ethical committee of the Helsinki and Uusimaa Hospital District. Written informed consent was provided by all participants.  Among participants with genome-wide data, after exclusion of no participants with dementia and no participants with a history of stroke, 452 participants were available for a GWAS, after removing one member of each monozygotic twin pair. 

**Genetic Epidemiology Network of Arteriopathy (GENOA)**

GENOA is a study of hypertensive sibships designed to investigate the genetic underpinnings of hypertension and target organ damage. In the initial phase of the GENOA study (Phase I: 1996-2001), all members of sibships containing ≥ 2 individuals with essential hypertension clinically diagnosed before age 60 were invited to participate, including both hypertensive and normotensive siblings (1,583 non-Hispanic whites from Rochester, MN, and 1,841 African Americans from Jackson, MS). The diagnosis of essential hypertension was established based on blood pressure levels measured at the study visit (>140 mmHg average systolic BP or >90 mmHg average diastolic BP) or a prior diagnosis of hypertension and current treatment with antihypertensive medications. Exclusion criteria were secondary hypertension, alcoholism or drug abuse, pregnancy, insulin-dependent diabetes mellitus, or active malignancy. In the second phase of the GENOA study (Phase II: 2000-2004), 1,241 white and 1,482 African American participants were successfully re-recruited to measure potential target organ damage due to hypertension. The Genetics of Microangiopathic Brain Injury (GMBI) study (2001-2006) is an ancillary study of GENOA undertaken to investigate susceptibility genes for ischemic brain injury. Phase II GENOA participants that had a sibling willing and eligible to participate in the GMBI study underwent a neurocognitive testing battery to assess several domains of cognitive function including Rey’s Auditory Verbal Learning Test (RAVLT)^65^ (967 whites and 1,010 African Americans). Genotyping was performed at the Mayo Clinic, Rochester (MN). GENOA white participants who had history of stroke (N=22) or dementia (N=11) were excluded from the analysis. Among participants with genome-wide genotype data, 842 GENOA white participants from 394 sibships were available for a GWAS on word list learning (RAVLT). 

**Generation Scotland (GS)**

Generation Scotland: the Scottish Family Health Study is a large family-based study with over 24,000 participants ^70,71^. Recruitment took place between 2006 and 2011 with probands (n=7,953) being aged between 35-65 years and registered with participating general practices (GPs) from 5 centres across Scotland. Their family members were also invited to participate in the study. The GWAS analyses were performed on 6,731 unrelated individuals, as defined by relatedness <0.025, which was calculated using GCTA-GREML ^72^. The sum of the immediate and delayed paragraph recall (one paragraph) of the Logical Memory test from the Wechsler Memory Scale III was the cognitive phenotype of interest ^67^. The scoring range was 0-50. DNA from blood (or saliva from a small number of postal and clinical participants) was collected on a GS sub-sample of 10,000 participants ^73^. Genotyping was conducted at the Clinical Research Facility Genetics Core, University of Edinburgh.

**The Hunter Community Study** (**HCS)**

The Hunter Community Study (HCS) is a population-based, prospective cohort study of older Australians from the Hunter Region, NSW Australia. Participants are community dwelling men and women aged 55-85, recruited from 2007-8. The purpose of the cohort was to assess factors important in the health, well-being, social functioning and economic consequences of ageing.

Participants attended a research clinic and had survey, clinical, genetic, biochemical, health services, economic, environmental as well as social and behavioural measures collected. The data collected included Mini Mental State Examination (MMSE), Audio-Recorded Cognitive Screen Neuropsychological battery and the Memory Complaint Questionnaire (MACQ). A full description of the cohort has been published previously ^74^.

During the clinic visit, participants also consented to blood sample collection and storage (DNA and whole blood) as well as consent for data linkage to Australian health databases. A total of 3,235 participants were enrolled into the study.

**The Health, Aging and Body Composition [Health ABC] Study**

The Health ABC study^75^ is a prospective cohort study investigating the associations between body composition, weight-related health conditions, and incident functional limitation in older adults.  Health ABC enrolled well-functioning, community-dwelling African-American (n=1,281) and white (n=1,794) men and women aged 70-79 years between April 1997 and June 1998.  Participants were recruited from a random sample of all Medicare eligible residents in the Pittsburgh, PA, and Memphis, TN, metropolitan areas.  Eligibility requirements included no difficulty with activities of daily living, walking a quarter of a mile, or climbing 10 steps without resting. Participants have undergone annual exams and semi-annual phone interviews. DNA extraction were carried out at baseline.

Among participants with genome-wide data, 432 participants were available for a GWAS after exclusion of participants with dementia and a history of stroke.

**Health and Retirement Study (HRS)**

The Health and Retirement Study (HRS) is a longitudinal survey of a representative sample of Americans over the age of 50.  The current sample includes over 26,000 persons in 17,000 households. Respondents are interviewed every two years about income and wealth, health and use of health services, work and retirement, and family connections.  DNA was extracted from saliva collected during a face-to-face interview in the respondents' homes.  These data represent respondents who provided DNA samples and signed consent forms in 2006 and 2008.  Respondents were removed if they were less than 18 years of age, had dementia, or had missing genotype or phenotype data. Among participants with genome-wide data, after exclusion of 195 participants with dementia and 560 participants with a history of stroke, 7904 participants were available for analysis.

**Helsinki Birth Cohort Study (HBCS)**

The source cohort for the HBCS comprised 4,130 women and 4,630 men born as singletons at Helsinki University Central Hospital during 1934-44, who had birth and child welfare records and were living in Finland in 1971 ^76^. To approach an intended sample size of N=2,000, a random subsample of 2,902 subjects was invited to participate in the study; 2,003 of them (1,075 women and 928 men) were finally included ^77^. Participants who could come to the examination center were invited to take a neuropsychological test battery, including the word list recall from the CERAD battery ^78^. 1,063 participants attended neuropsychological testing between February 2005 and February 2011. DNA was extracted from 1,728 randomly selected participants of the HBCS. Genotyping was conducted at the Welcome Trust Sanger Institute, Cambridge, UK. Among participants with available cognitive tests and genome-wide genotypes, after exclusion of 22 participants with a history of stroke, 888 individuals were available for a GWAS of word list learning (visually presented word list). The study was approved by the Institutional Review Board of the National Public Health Institute, and informed consent was obtained from all participants.

**Lothian Birth Cohort 1921 (LBC1921) and 1936 (LBC1936)**

The Lothian Birth Cohorts of 1921 and 1936 (LBC1921, LBC1936) include surviving participants from the Scottish Mental Surveys of 1932 or 1947 (SMS1932 and SMS1947), having been born, respectively in 1921 and 1936 ^79-81^. The LBC1921 cohort consists of 550 relatively healthy individuals, 316 females and 234 males, assessed on cognitive and medical traits at about 79 years of age. When tested, the sample had a mean age of 79.1 years (SD = 0.6). The LBC1936 consists of 1091 relatively healthy individuals assessed on cognitive and medical traits at about 70 years of age.  At baseline the sample of 548 men and 543 women had a mean age 69.6 years (SD = 0.8). They were all Caucasian and almost all lived independently in the Lothian region (Edinburgh city and surrounding area) of Scotland. Genotyping was performed at the Wellcome Trust Clinical Research Facility, Edinburgh. Quality control measures were applied; 468 and 953 participants remained for LBC1921 and LBC1936 respectively. Ethics permission for the study was obtained from the Multi-Centre Research Ethics Committee for Scotland (MREC/01/0/56) and from Lothian Research Ethics Committee (LBC1936: LREC/2003/2/29 and LBC1921: LREC/1998/4/183). The research was carried out in compliance with the Helsinki Declaration. All subjects gave written, informed consent.

**Leipzig Research Center for Civilization Diseases (*****LIFE-ADULT*****)**

LIFE-Adult is a population-based cohort study of 10,000 inhabitants of the city of Leipzig, Germany^82^. Recruitment was age- and sex-stratified with an age range 18-80 years. At the time of the present analysis N = 3,447 were successfully genotyped with Affymetrix-Axiom CEU SNP-Array.  After SNP quality control comprising default Affymetrix measures of cluster quality, callrate>=97%, p(HWE)>10^-6^, p-value of plate association >10^-7^, 527,847 SNPs remained. Imputation was performed using HapMap2 release 22 reference panel and the software IMPUTE Version 2.3.2. N=2,200 elderly participants (age range 60-80 years) received an in-depth neuropsychological assessment including CERADplus Neuropsychological test battery, Trail making test and structured interviews for the diagnosis of dementia.

Among participants with genome-wide data, after exclusion of 0 participants with dementia and 27 participants with a history of stroke, 1095 participants were available for a GWAS.

**LOGOS (Learning on Genetics of Schizophrenia Spectrum)**

The LOGOS project recruited 1540 randomly selected young male conscripts from the Greek Army (mean age 22.13; range 18–44) between June 2008 and July 2011 at the Military Training Camp of Candidate, Supply Army officers (SEAP) in Heraklion, Crete. Following public presentation of the study’s methods and goals in each consecutive series of new conscripts, all participants willing to volunteer received a detailed information sheet and gave written informed consent before screening. All subjects were thoroughly screened for past or current physical and mental health status by the army medical authorities, the study nurse and a trained research psychologist. They underwent a Mini-International Neuropsychiatric Interview,^36^ and were tested on a single occasion at some point during their 2 months military training in this establishment. Inclusion criteria were recent (last two months) conscript status in the camp and written informed consent. Exclusion criteria were left-handedness (n=150), personal history of head trauma, medical and neurological conditions (n=68), personal history of DSM-IV Axis I disorders (n=95), current use of prescribed drugs or a positive recreational drug screen (n=0) and a hearing test (n=53). On the basis of these criteria, and after 47 subjects who dropped out, cognitive and genetic data were available for 866 subjects. The LOGOS study was approved by the Ethics Committee of the University of Crete, the Executive Army Bureau, and the Bureau for the Protection of Personal and Sensitive Data of the Greek State. LOGOS was genotyped on Illumina HumanOmniExpress array.

**Older Australian Twins Study (OATS)**

Twins aged at least 65 years old were recruited from Twins Registry Australia and through a recruitment drive from the three eastern Australian states (N=623).  Exclusion criteria included a life-threatening illness or a current diagnosis of an acute psychiatric illness or insufficient English to complete the interview.  An extensive assessment was performed including medical history, and lifestyle factors and demographic information was collected.  A comprehensive cognitive battery was performed, including a test of verbal short-term memory (Story A of WMS-III Logical Memory, Weschler, 1999). Blood was collected for genetic analyses.  DNA was extracted using standard protocols and genome-wide genotyping was undertaken using the Illumina Omniexpress array at the Queensland Diamantina Institute, Australia. 

Ethics approval was granted by Twins Registry Australia, University of New South Wales, University of Melbourne, Queensland Institute of Medical Research and the South Eastern Sydney and Illawarra Area Health Service University of New South Wales and the Illawarra Area Health Service Human Research Ethics Committees.  All participants gave written informed consent.  Further details can be found in Sachdev et al. 2009^83^.

For this study, among participants with genome-wide genotyping data, after exclusion of 12 participants with dementia and 25 participants with a history of stroke, 467 participants were available for a GWAS with data for immediate paragraph recall (WMS-III)^84^.

**Orkney Complex Disease Study (ORCADES)**

The Orkney Complex Disease Study (ORCADES) is a family-based, cross-sectional study that seeks to identify genetic factors influencing cardiovascular and other disease risk in the isolated archipelago of the Orkney Isles in northern Scotland ^85^. Genetic diversity in this population is decreased compared to Mainland Scotland, consistent with the high levels of endogamy historically. 2078 participants aged 16-100 years were recruited between 2005 and 2011, most having three or four grandparents from Orkney, the remainder with two Orcadian grandparents. Fasting blood samples were collected and many health-related phenotypes and environmental exposures were measured in each individual. All participants gave written informed consent and the study was approved by Research Ethics Committees in Orkney and Aberdeen (North of Scotland REC). Participants were invited to take a neuropsychological test battery including the logical memory test (immediate paragraph recall) adapted from the Original Wechsler Memory Scale^86^. 

**PROspective Study of Pravastatin in the Elderly at Risk (Prosper IE/NL/SC)**

All data come from the PROspective Study of Pravastatin in the Elderly at Risk (PROSPER). A detailed description of the study has been published elsewhere. PROSPER was a prospective multicenter randomized placebo-controlled trial to assess whether treatment with pravastatin diminishes the risk of major vascular events in elderly. Between December 1997 and May 1999, we screened and enrolled subjects in Scotland (Glasgow), Ireland (Cork), and the Netherlands (Leiden). Men and women aged 70-82 years were recruited if they had pre-existing vascular disease or increased risk of such disease because of smoking, hypertension, or diabetes. A total number of 5,804 subjects were randomly assigned to pravastatin or placebo. A large number of prospective tests were performed including Biobank tests and cognitive function measurements. Memory was assessed with the 15-Picture Learning test (PLT) testing immediate and delayed recall. The main outcome parameters were the accumulated number of recalled pictures over the three learning trials and the number of pictures recalled after 20 minutes.  A whole genome wide screening has been performed in the sequential PHASE project. Of 5,763 subjects DNA was available for genotyping. Genotyping was performed with the Illumina 660K beadchip, after QC (call rate <95%) 5,244 subjects and 557,192 SNPs were left for analysis. These SNPs were imputed to 2.5 million SNPs based on the HAPMAP built 36 with MACH imputation software.  The study was approved by the institutional ethics review boards of centers of Cork University (Ireland), Glasgow University (Scotland) and Leiden University Medical Center (the Netherlands) and all participants gave written informed consent.

Among participants with cognitive function and genome-wide data (n=4933), after exclusion of 0 participants with dementia and 586 participants with a history of stroke, 4389 participants were available for a GWAS. ^87-89^

**Rotterdam Study (RS)**  

The Rotterdam Study is a population-based prospective cohort study among inhabitants of a district of Rotterdam (Ommoord), the Netherlands, and aims to examine the determinants of disease and health in the elderly with a focus on cardiovascular, endocrine, hepatic, neurological, ophthalmic, psychiatric, dermatological, otolaryngological, locomotor, and respiratory diseases.^90^ In 1990-1993, 7,983 persons 55 years of age or over participated and were re-examined every 3 to 4 years (Rotterdam Study-I). In 1999, 3,011 individuals who had become 55 years of age or moved into the study district since the start of the study were added to the cohort (Rotterdam Study-II), and in 2006 a further extension of the cohort was initiated in which 3,932 subjects aged 45–54 years and living in the same district were included (Rotterdam Study-III). All participants had DNA extracted at their first visit. Genotyping was attempted in participants with high-quality extracted DNA. Genotyping was done at the Human Genotyping Facility, Genetic Laboratory Department of Internal Medicine, Erasmus MC, Rotterdam, the Netherlands. Participants underwent several neuropsychological tests at the baseline and follow-up examinations,^91^ including a 15-word verbal learning test based on Rey’s recall of words.^92^ Participants are continuously monitored for major events, including dementia and stroke, by automated linkage of the general practitioners’ records and hospital discharge files with the study database.^93,94^ Among participants with good quality genome-wide data: after exclusion of participants with dementia (N=124 for the Rotterdam Study-I, N=56 for the Rotterdam Study-II and N=6 for the Rotterdam Study-III) and participants with a history of stroke (N=168 for the Rotterdam Study and N=102 for the Rotterdam Study-II), 2,138 participants from the Rotterdam Study, 1,368 participants from the Rotterdam Study-II and 1,916 participants from the Rotterdam Study-3 were available for a GWAS of verbal learning and immediate recall (visually presented word list). 

**Religious Orders Study and Rush Memory and Aging Project (ROSMAP)**

The ROS, started in 1994, enrolled Catholic priests, nuns, and brothers from about 40 groups in the United States ^95,96^. The MAP, started in 1997, enrolled older persons from about 40 continuous care retirement communities and subsidized housing facilities in the Chicago metropolitan area^97^. Both studies were approved by the institutional review board of Rush University Medical Center. The follow-up rate of survivors exceeds 90%. Participants were free of known dementia at enrollment, agreed to annual clinical evaluations, and signed both an informed consent and an Anatomic Gift Act for donating their brains at time of death ^98^. Participants were invited to take a neuropsychological test battery, including delayed recall of Story A from the logical memory subset of the Wechsler Memory Scale-Revised,^51,61^ and delayed word list recall from the Consortium to Establish a Registry for Alzheimer’s Disease (CERAD) battery ^78^. DNA was extracted from whole blood, lymphocytes, or frozen postmortem brain tissue. Genotyping was performed at the Broad Institute’s Center for Genotyping and the Translational Genomics Research Institute^99^. Among participants with available memory tests and genome-wide genotypes, after exclusion of 16 participants with dementia and a history of stroke, 798 ROS participants and 875 MAP participants were available for a GWAS of verbal learning (CERAD), and 801 ROS participants and 874 MAP participants were available for a GWAS of short term memory. ROSMAP data can be requested at www.radc.rush.edu.

**Study of Health in Pomerania (SHIP)**

The Study of Health in Pomerania is a population-based epidemiological study in the region of Western Pomerania, Germany ^100^. In brief, from the total population of West Pomerania comprising 213,057 inhabitants in 1996, a twostage stratified cluster sample of adults aged 20–79 years was drawn. The net sample (without migrated or deceased persons) comprised 6,265 eligible subjects, out of which 4,308 completed their baseline examinations. From July 2007 to October 2010 the Life-Events and Gene-Environment Interaction in Depression (LEGENDE) study was carried out in the SHIP cohort. A diagnostic interview for mental disorders was performed based on Diagnostic and Statistical Manual for Mental Disorders (IV edition) diagnostic criteria ^101^. As part of the SHIP-LEGENDE project,^102^ participants have been invited to take a cognitive test battery, including a German adaptation of Rey’s Auditory Verbal Learning Test (RAVLT)^103^. Genotyping was performed at Affymetrix (Santa Clara, CA). Among participants with available cognitive tests and genome-wide genotypes, we excluded 119 participants with a history of stroke, resulting in. 2,179 participants aged 20 years or older available for a GWAS of verbal learning and immediate word list recall (RAVLT).

**Sydney Memory and Ageing Study (Sydney MAS)**

This study began in 2005 to examine the clinical characteristics and prevalence of mild cognitive impairment and related syndromes, and to determine the rate of cognitive change with ageing in older adults ^104^. Participants aged 70-90 years of age were randomly recruited from the electoral roll in Sydney.  Exclusion criteria included a diagnosis of dementia.  A comprehensive assessment was undertaken, which included a neuropsychological test battery and blood samples for DNA testing.  Cognitive tests included the RAVLT^65^, a test of verbal learning, and a test of memory, Story A of the Wechsler Memory Scale (WMS-III) (Wechsler, 1999).  DNA was genotyped using the Affymetrix 6.0 array at the Ramaciotti Centre, UNSW Sydney, Australia. 

Ethics approval for the study was provided by the University of New South Wales and the Illawarra Area Health Service Human Research Ethics Committees.  All participants gave written informed consent to join the study.

Among the 925 participants with available genome-wide data, after exclusion of 41 participants with a history of stroke, 888 individuals were available for performing a GWAS of verbal learning (RAVLT) and 888 for immediate paragraph recall (WMS-III)^84^.

**Women's Genome Health Study (WGHS)**

The WGHS is a large cohort for genome-wide genetic analysis of a wide range of clinical phenotypes among >25,000 women, 45 years or older at baseline and with ongoing follow-up observation, now for approximately 18 years^105^. The population is derived from participants in the Women’s Health Study who provided a blood sample at baseline. By design, participants included in the WGHS were free from dementia and stroke at baseline. For the cognitive function substudy, women above 70 years of age were contacted for a baseline telephone cognitive assessment, including a measure of immediate paragraph recall using the East Boston Memory Test, and the Telephone Interview of Cognitive Status 10–item word list ^106^. Genome-wide genotyping in the WGHS was performed using the Illumina (San Diego, CA) HumanHap 300 Duo “+” platform including a total 339,596 SNPs passing quality control filters among 23,294 participants with verified European ancestry. Among participants with available memory tests and genome-wide genotypes, 3,542 individuals of verified European ancestry were available for genetic analysis of immediate word list and paragraph recall. WGHS HapMap II imputation: Among the final 23,294 individuals of verified European ancestry, genotypes for a total of 2,608,508 SNPs were imputed from the experimental genotypes for 340,349 SNPs and LD relationships implicit in the HapMap r. 22 CEU samples.

# ACKNOWLEDGEMENTS

**Alzheimer's Disease Neuroimaging Initiative (ADNI)**

Data used in the preparation of this article were obtained from the Alzheimer's Disease Neuroimaging Initiative (ADNI) database ([adni.loni.usc.edu](http://adni.loni.ucla.edu/)). As such, the investigators within the ADNI contributed to the design and implementation of ADNI and/or provided data but did not participate in analysis or writing of this report. A complete listing of ADNI investigators can be found at [http://adni.loni.usc.edu/wp-content/uploads/how_to_apply/ADNI_Acknowledgement_List.pdf](http://adni.loni.usc.edu/wp-content/uploads/how_to_apply/ADNI_Acknowledgement_List.pdf).

Data collection and sharing for this project was funded by the Alzheimer's Disease Neuroimaging Initiative (ADNI) (National Institutes of Health Grant U01 AG024904) and DOD ADNI (Department of Defense award number W81XWH-12-2-0012). ADNI is funded by the National Institute on Aging, the National Institute of Biomedical Imaging and Bioengineering, and through generous contributions from the following: Alzheimer’s Association; Alzheimer’s Drug Discovery Foundation; BioClinica, Inc.; Biogen Idec Inc.; Bristol-Myers Squibb Company; Eisai Inc.; Elan Pharmaceuticals, Inc.; Eli Lilly and Company; F. Hoffmann-La Roche Ltd and its affiliated company Genentech, Inc.; GE Healthcare; Innogenetics, N.V.; IXICO Ltd.; Janssen Alzheimer Immunotherapy Research & Development, LLC.; Johnson & Johnson Pharmaceutical Research & Development LLC.; Medpace, Inc.; Merck & Co., Inc.; Meso Scale Diagnostics, LLC.; NeuroRx Research; Novartis Pharmaceuticals Corporation; Pfizer Inc.; Piramal Imaging; Servier; Synarc Inc.; and Takeda Pharmaceutical Company. The Canadian Institutes of Health Research is providing funds to support ADNI clinical sites in Canada. Private sector contributions are facilitated by the Foundation for the National Institutes of Health (www.fnih.org). The grantee organization is the Northern California Institute for Research and Education, and the study is coordinated by the Alzheimer's Disease Cooperative Study at the University of California, San Diego. ADNI data are disseminated by the Laboratory for Neuro Imaging at the University of Southern California. Samples from the National Cell Repository for AD (NCRAD), which receives government support under a cooperative agreement grant (U24 AG21886) awarded by the National Institute on Aging (AIG), were used in this study.

Additional support for data analysis was provided by NLM R01 LM012535, NIA grants R03 AG054936, P30 AG010133, R01 AG019771, and K01 AG049050, the Alzheimer’s Association, the Indiana Clinical and Translational Science Institute, and the IU Health-IU School of Medicine Strategic Neuroscience Research Initiative.

**Ageing, Cognition and Dementia in Primary Care Patients (AgeCoDe)**

The work described in the present publication was performed within the context of the German Research Network on Dementia (KND) and the German Research Network on Degenerative Dementia (KNDD), which are funded by the German Federal Ministry of Education and Research (grants KND: 01GI0102, 01GI0420, 01GI0422, 01GI0423, 01GI0429, 01GI0431, 01GI0433, 01GI0434; grants KNDD: 01GI1007A, 01GI0710, 01GI0711, 01GI0712, 01GI0713, 01GI0714, 01GI0715, 01GI0716, 01ET1006B). 

**Age, Gene/Environment Susceptibility (AGES)**

This study has been funded by NIH contracts N01-AG-1-2100 and 271201200022C, the NIA Intramural Research Program, Hjartavernd (the Icelandic Heart Association), and the Althingi (the Icelandic Parliament). The study is approved by the Icelandic National Bioethics Committee, VSN: 00-063. The researchers are indebted to the participants for their willingness to participate in the study.

**The Atherosclerosis Risk in Communities (ARIC)**

The Atherosclerosis Risk in Communities study has been funded in whole or in part with Federal funds from the National Heart, Lung, and Blood Institute, National Institutes of Health, Department of Health and Human Services (contract numbers HHSN268201700001I, HHSN268201700002I, HHSN268201700003I, HHSN268201700004I and HHSN268201700005I), R01HL087641, R01HL086694; National Human Genome Research Institute contract U01HG004402; and National Institutes of Health contract HHSN268200625226C. The authors thank the staff and participants of the ARIC study for their important contributions. Infrastructure was partly supported by Grant Number UL1RR025005, a component of the National Institutes of Health and NIH Roadmap for Medical Research. 

**The Coronary Artery Risk Development in Young Adults Study (CARDIA)**

The Coronary Artery Risk Development in Young Adults Study (CARDIA) is conducted and supported by the National Heart, Lung, and Blood Institute (NHLBI) in collaboration with the University of Alabama at Birmingham (HHSN268201800005I & HHSN268201800007I), Northwestern University (HHSN268201800003I), University of Minnesota (HHSN268201800006I), and Kaiser Foundation Research Institute (HHSN268201800004I). CARDIA was also partially supported by the Intramural Research Program of the National Institute on Aging (NIA) and an intra‐agency agreement between NIA and NHLBI (AG0005). Genotyping was funded as part of the NHLBI Candidate-gene Association Resource (N01-HC-65226) and the NHGRI Gene Environment Association Studies (GENEVA) (U01-HG004729, U01-HG04424, and U01-HG004446). Analyses were supported by NIH grants R01 HL122658 and R01-NS087541.

**The Cardiovascular Health Study (CHS)**

This CHS research was supported by NHLBI contracts HHSN268201200036C, HHSN268200800007C,  HHSN268201800001C, N01HC55222, N01HC85079, N01HC85080, N01HC85081, N01HC85082, N01HC85083, N01HC85086; and NHLBI grants U01HL080295, R01HL087652, R01HL105756, R01HL103612, R01HL120393, and U01HL130114 with additional contribution from the National Institute of Neurological Disorders and Stroke (NINDS). Additional support was provided through R01AG023629, R01AG15928, R01AG20098, and R01AG033193 from the National Institute on Aging (NIA). A full list of principal CHS investigators and institutions can be found at [CHS-NHLBI.org](http://chs-nhlbi.org/).

The provision of genotyping data was supported in part by the National Center for Advancing Translational Sciences, CTSI grant  UL1TR001881, and the National Institute of Diabetes and Digestive and Kidney Disease Diabetes Research Center (DRC) grant DK063491 to the Southern California Diabetes Endocrinology Research Center.

**Erasmus Rucphen Family study (ERF)**

Erasmus Rucphen Family (ERF) was supported by the Consortium for Systems Biology (NCSB), both within the framework of the Netherlands Genomics Initiative (NGI)/Netherlands Organisation for Scientific Research (NWO). ERF study as a part of EUROSPAN (European Special Populations Research Network) was supported by European Commission FP6 STRP grant number 018947 (LSHG-CT-2006-01947) and also received funding from the European Community’s Seventh Framework Programme (FP7/2007-2013)/grant agreement HEALTH-F4-2007-201413 by the European Commission under the programme “Quality of Life and Management of the Living Resources” of 5th Framework Programme (No. QLG2-CT-2002-01254) as well as FP7 project EUROHEADPAIN (nr 602633). High-throughput analysis of the ERF data was supported by joint grant from Netherlands Organisation for Scientific Research and the Russian Foundation for Basic Research (NWO-RFBR 047.017.043). High throughput metabolomics measurements of the ERF study has been supported by BBMRI-NL (Biobanking and Biomolecular Resources Research Infrastructure Netherlands).

**FinnTwin16 (FT16)**

We thank all study participants and all the staff of the twin study. Phenotype and genotype data collection in the twin cohort has been supported by  the Wellcome Trust Sanger Institute, the Broad Institute, ENGAGE – European Network for Genetic and Genomic Epidemiology, FP7-HEALTH-F4-2007, grant agreement number 201413, National Institute of Alcohol Abuse and Alcoholism (grants AA-12502, AA-00145, and AA-09203 to R J Rose and AA15416 and K02AA018755 to D M Dick) and the Academy of Finland (grants 100499, 205585, 118555, 141054, 264146, 308248,  and 312073 to JKaprio). JKaprio acknowledges support by the Academy of Finland (grants 265240, 263278). E Vuoksimaa acknowledges support by the Academy of Finland (grant 314639).

**Framingham Heart Study (FHS)**

We thank the Framingham Heart Study (FHS) participants, as well as the study team (especially the investigators and staff of the neurology team) for their contributions and dedication to the study. The authors are pleased to acknowledge that the computational work reported on in this paper was performed on the Shared Computing Cluster, which is administered by Boston University Research Computing Services. URL: [www.bu.edu/tech/support/research/](http://www.bu.edu/tech/support/research/).

This work was supported by the National Heart, Lung and Blood Institute’s Framingham Heart Study (Contract No. N01-HC-25195, No. HHSN268201500001I and No. 75N92019D00031). This study was also supported by grants from the National Institute of Aging (R01s AG054076, AG049607, AG059421, U01-AG049505, and AG052409), the National Institute of Neurological Disorders and Stroke (R01 NS017950) and the National Heart, Lung and Blood Institute (UH2 NS100605, R01 HL093029, HL096917). 

**Genetic Epidemiology Network of Arteriopathy (GENOA)**

Support for the Genetic Epidemiology Network of Arteriopathy (GENOA) was provided by the National Heart, Lung and Blood Institute (HL054464, HL054457, HL054481, HL087660, HL119443) and the National Institute of Neurological Disorders and Stroke (NS041558) of the National Institutes of Health. Genotyping was performed at the Mayo Clinic and was made possible by the University of Texas Health Sciences Center. We would also like to thank the families that participated in the GENOA study.

**Generation Scotland (GS)**

Generation Scotland has received core funding from the Chief Scientist Office of the Scottish Government Health Directorates CZD/16/6 and the Scottish Funding Council HR03006. Genotyping of the GS:SFHS samples was carried out by the Genetics Core Laboratory at the Clinical Research Facility, University of Edinburgh, Scotland and was funded by the UK Medical Research Council (MRC). The Quantitative Trait Locus team at the Human Genetics Unit are funded by the MRC. REM and IJD undertook the work within The University of Edinburgh Centre for Cognitive Ageing and Cognitive Epidemiology (MR/K026992/1), part of the cross council Lifelong Health and Wellbeing Initiative. Funding from the BBSRC and MRC is gratefully acknowledged. Genotyping of the GS:SFHS samples was carried out by the Genetics Core Laboratory at the Edinburgh Clinical Research Facility, University of Edinburgh, Scotland and was funded by the Medical Research Council UK and the Wellcome Trust (Wellcome Trust Strategic Award “STratifying Resilience and Depression Longitudinally” (STRADL) Reference 104036/Z/14/Z).

**The Health, Aging and Body Composition [Health ABC] Study**

The Health ABC Study research was supported by in part by the Intramural Research Program of the NIH, National Institute on Aging (NIA) and by NIA contracts N01AG62101, N01AG62103, and N01AG62106. The genome-wide association study was funded by NIA grant 1R01AG032098-01A1 to Wake Forest University Health Sciences and genotyping services were provided by the Center for Inherited Disease Research (CIDR). CIDR is fully funded through a federal contract from the National Institutes of Health to The Johns Hopkins University, contract number HHSN268200782096C. This work utilized the computational resources of the NIH HPC **Biowulf**cluster. (http://hpc.nih.gov).

**Health and Retirement Study (HRS)**

HRS is supported by the National Institute on Aging (NIA U01AG009740). The
genotyping was funded separately by the National Institute on Aging (RC2
AG036495, RC4 AG039029), and data analysis was funded in part by R03 AG048806. Our genotyping was conducted by the NIH Center for Inherited Disease Research (CIDR) at Johns Hopkins University. Genotyping quality control and final preparation of the data were performed by the Genetics Coordinating Center at the University of Washington. 

**The Hunter Community Study (HCS)**

The Hunter Community Study funding for the study was obtained from The University of Newcastle, who provided $300,000 from its Strategic Initiatives Fund, and $600,000 from a philanthropic fellowship fund, while a private philanthropic trust, the Vincent Fairfax Family Foundation, provided another $195,000. The Hunter Medical Research Institute provided media support during the initial recruitment of participants. Follow up funding of the cohort was obtained from the National Health and Medical Research Council of Australia (NH&MRC) and Beyond Blue.

**Helsinki Birth Cohort Study (HBCS)**

Helsinki Birth Cohort Study: We thank all study participants as well as everybody involved in the Helsinki Birth Cohort Study. The Helsinki Birth Cohort Study has been supported by Grants from the Academy of Finland, the Finnish Diabetes Research Society, Folkhälsan Research Foundation, Novo Nordisk Foundation, Finska Läkaresällskapet, Signe and Ane Gyllenberg Foundation, University of Helsinki, Ministry of Education, Ahokas Foundation, Emil Aaltonen Foundation, Juho Vainio Foundation, and Wellcome Trust (Grant number WT089062).

**Lothian Birth Cohort 1921 (LBC1921) and 1936 (LBC1936)**

We thank the cohort participants and team members who contributed to these studies. Phenotype collection in the Lothian Birth Cohort 1921 was supported by the UK Biotechnology and Biological Sciences Research Council (BBSRC), The Royal Society and The Chief Scientist Office of the Scottish Government. Phenotype collection in the Lothian Birth Cohort 1936 was supported by Research Into Ageing (continues as part of Age UK The Disconnected Mind project). Genotyping of the cohorts was funded by the BBSRC. The work was undertaken by The University of Edinburgh Centre for Cognitive Ageing and Cognitive Epidemiology (CCACE), part of the cross council Lifelong Health and Wellbeing Initiative (MR/K026992/1). Funding from the BBSRC and Medical Research Council (MRC) is gratefully acknowledged.

**Leipzig Research Center for Civilization Diseases (*****LIFE-ADULT*****)**

LIFE is funded by means of the European Union, by the European Regional Development Fund (ERDF) and by funds of the Free State of Saxony within the framework of the excellence initiative (project numbers 713-241202, 713-241202, 14505/2470, 14575/2470).

**Older Australian Twins Study (OATS)**

We would like to gratefully acknowledge and thank all OATS participants and their supporters for their generosity.  We also gratefully acknowledge the OATS Research Team (former and current staff and students) for their contributions (see (https:// cheba.unsw.edu.au/project/older-australian-twins-study).  This study is supported by the Australian National Health and Medical Research Council (NHMRC)/Australian Research Council Strategic Award (Grant 401162) and the NHMRC Project grant 1405325.  This research was facilitated through Twins Research Australia, a national resource in part supported by a Centre for Research Excellence from the NHMRC.

**Orkney Complex Disease Study (ORCADES)**

The Orkney Complex Disease Study (ORCADES) was supported by the Chief Scientist Office of the Scottish Government (CZB/4/276, CZB/4/710), a Royal Society URF to J.F.W., the MRC Human Genetics Unit quinquennial programme “QTL in Health and Disease”, Arthritis Research UK and the European Union framework program 6 EUROSPAN project (contract no. LSHG-CT-2006-018947). DNA extractions were performed at the Wellcome Trust Clinical Research Facility in Edinburgh. We would like to acknowledge the invaluable contributions of the research nurses in Orkney, the administrative team in Edinburgh and the people of Orkney.

**PROspective Study of Pravastatin in the Elderly at Risk (Prosper IE/NL/SC)**

The PROSPER study was supported by an investigator initiated grant obtained from Bristol-Myers Squibb. Prof. Dr. J. W. Jukema is an Established Clinical Investigator of the Netherlands Heart Foundation (grant 2001 D 032). Support for genotyping was provided by the seventh framework program of the European commission (grant 223004) and by the Netherlands Genomics Initiative (Netherlands Consortium for Healthy Aging grant 050-060-810). 

**Religious Orders Study and Rush Memory and Aging Project (ROSMAP)**

The Religious Orders Study and Rush Memory and Aging Project are supported by National Institute on Aging Grants R01AG36042, U01AG61356, P30AG10161, R01AG17917, R01AG15819, and the Translational Genomics Research Institute. We thank the study participants and the staff of the Rush Alzheimer’s Disease Center.

**Rotterdam Study (RS I/II/III)**

The generation and management of GWAS genotype data for the Rotterdam Study (RS I, RS II, RS III) was executed by the Human Genotyping Facility of the Genetic Laboratory of the Department of Internal Medicine, Erasmus MC, Rotterdam, The Netherlands. The GWAS datasets are supported by the Netherlands Organisation of Scientific Research NWO Investments (nr. 175.010.2005.011, 911-03-012), the Genetic Laboratory of the Department of Internal Medicine, Erasmus MC, the Research Institute for Diseases in the Elderly (014-93-015; RIDE2), the Netherlands Genomics Initiative (NGI)/Netherlands Organisation for Scientific Research (NWO) Netherlands Consortium for Healthy Aging (NCHA), project nr. 050-060-810. We thank Pascal Arp, Mila Jhamai, Marijn Verkerk, Lizbeth Herrera and Marjolein Peters, MSc, and Carolina Medina-Gomez, MSc, for their help in creating the GWAS database, and Karol Estrada, PhD, Yurii Aulchenko, PhD, and Carolina Medina-Gomez, MSc, for the creation and analysis of imputed data. 

The Rotterdam Study is funded by Erasmus Medical Center and Erasmus University, Rotterdam, Netherlands Organization for the Health Research and Development (ZonMw), the Research Institute for Diseases in the Elderly (RIDE), the Ministry of Education, Culture and Science, the Ministry for Health, Welfare and Sports, the European Commission (DG XII), and the Municipality of Rotterdam. The authors are grateful to the study participants, the staff from the Rotterdam Study and the participating general practitioners and pharmacists. 

The Rotterdam Study has been approved by the Medical Ethics Committee of the Erasmus MC (registration number MEC 02.1015) and by the Dutch Ministry of Health, Welfare and Sport (Population Screening Act WBO, license number 1071272-159521-PG). The Rotterdam Study has been entered into the Netherlands National Trial Register (NTR; [www.trialregister.nl](http://www.trialregister.nl/)) and into the WHO International Clinical Trials Registry Platform (ICTRP; [www.who.int/ictrp/network/primary/en/](http://www.who.int/ictrp/network/primary/en/)) under shared catalogue number NTR6831. All participants provided written informed consent to participate in the study and to have their information obtained from treating physicians.

**Study of Health in Pomerania (SHIP)**

SHIP is part of the Community Medicine Research net of the University of Greifswald, Germany, which is funded by the Federal Ministry of Education and Research (grants no. 01ZZ9603, 01ZZ0103, and 01ZZ0403), the Ministry of Cultural Affairs as well as the Social Ministry of the Federal State of Mecklenburg-West Pomerania, and the network ‘Greifswald Approach to Individualized Medicine (GANI_MED)’ funded by the Federal Ministry of Education and Research (grant 03IS2061A). Genome-wide data have been supported by the Federal Ministry of Education and Research (grant no. 03ZIK012) and a joint grant from Siemens Healthineers, Erlangen, Germany and the Federal State of Mecklenburg- West Pomerania. The University of Greifswald is a member of the Caché Campus program of the InterSystems GmbH.

**Sydney Memory and Ageing Study (Sydney MAS)**

We thank the Sydney MAS participants and their supporters for their contributions to the study.  We also acknowledge the Sydney MAS Research Team (former and current staff and students).  This study is supported by the Australian National Health and Medical Research Council (NHMRC) Program Grants 350833, 568969 and 109308.

**Women's Genome Health Study (WGHS)**

The WGHS is supported by the National Heart, Lung, and Blood Institute (HL043851 and HL080467) and the National Cancer Institute (CA047988 and UM1CA182913), with funding for genotyping provided by Amgen. Collection of cognitive function in the Women’s Health Study, parent cohort to the WGHS, was funded by the National Institute for Aging (AG15933).

# VERBAL MEMORY TESTS

## Word list tests with oral presentation

Rey’s Auditory Verbal learning test (RAVLT): used by ADNI1, ADNIGO2, CARDIA, ERF, GENOA, Sydney MAS, and SHIP: In the first part of the test a first list of 15 semantically unrelated common words is read to the participant followed by an immediate recall test where the participant is asked to say the words he/she can recall. There are 5 such exposure trials (3 trials for SHIP). In all cohorts using RAVLT^65^, verbal learning variable is the sum of words correctly recalled in all the trials. In ADNIGO2, CARDIA, ERF, GENOA, and SHIP verbal short-term memory phenotype is the total number of words correctly recalled in the first trial of the RAVLT.

California Verbal Learning Test (CVLT) used by AGES, CHS and FT: The CVLT^48^ consists of lists of 16 items, belonging to one of four semantic categories of "shopping list" items: for example, the first-"Monday's" list-contains four names of fruits, of herbs and spices, of articles of clothing, and of tools. There were 4 repeated trials in a different order for AGES and 5 in the same order for CHS and FT. In all cohorts using CVLT, verbal learning variable is the sum of words correctly recalled in all the trials. In FT verbal short-term memory phenotype is the total number of words correctly recalled in the first trial of the CVLT.

Hopkins Verbal Learning Test (HVLT) used by the HCS: The HVLT^107^ involves presenting 12 words (4 words from 3 semantic categories) verbally. All 12 words are presented for 3 trials. After each trial participants are to recall as many words from the list as they can remember. Verbal learning variable is the sum of words correctly recalled in all the trials. In the HCS three alternative 12 word lists have been generated and for the baseline exam used in the present analyses, one of these three lists was chosen randomly. The three lists were shown to be equivalent.^108^

Buschke selective reminding test (Buschke SRT)^109^ used by the Health ABC: The test consists of an immediate recall of a list of 12 written words. After the recall only those words not recalled on the trial are presented. There were three repeats in the test. Verbal learning variable is the sum of words correctly recalled in all the trials and verbal short-term memory phenotype is the total number of words correctly recalled in the first trial of the test. 

10-word list used by HRS^110^: 10-word list is adapted from the Telephone Interview of the Cognitive Status (TICS). The test is administered over the phone and consists of 10-item non-semantically related word list, which the participant is asked to recall immediately.

## Word list tests with visual presentation

Rey’s recall of words,^111^ used by the Rotterdam Study, Rotterdam Study-II and Rotterdam Study-III: In this test participants are shown 15 different semantically unrelated words (from list A of the RAVLT) and then asked to recall as many as possible. This is done 3 consecutive times. In all cohorts using Rey’s recall of words, verbal learning variable is the sum of words correctly recalled in all the trials and verbal short-term memory phenotype is the total number of words correctly recalled in the first trial of the test.

Consortium to Establish a Registry for Alzheimer’s Disease (CERAD) used by the AgeCoDe, HBCS, LIFE-Adult, ROS, MAP, and FHS2: The list learning test is a part of a larger set of tests from CERAD^78^. The word list learning task consists of learning a list of 10 semantically unrelated written words, read aloud by the participant. This list is repeated 3 times (in a different order) and after each presentation, the participant recalls as many words as possible. In all cohorts using CERAD, verbal learning variable is the sum of words correctly recalled in all the trials. In AgeCoDe, FHS, HBCS, and LIFE-Adult verbal short-term memory phenotype is the total number of words correctly recalled in the first trial of the CERAD.

PLT^111^: Used by the Prosper(IE), Prosper (NL), and Prosper (SC): Memory was tested using the Picture-Word Recall test based on the Groningen-Fifteen Words test. This measures immediate recall of 15 pictures. Prosper was included in the analysis despite that the recalled material was presented as pictures due to the fact that the same words are used in the RAVLT and the Rey’s recall of words and there were no differences between verbal presentation and visual presentation^111^. The outcome variable is the mean number of correctly recalled pictures over three immediate trials.

CANTAB verbal recall^112^ used by DUKE1 and DUKE2: The participant is shown a sequence of words on screen one by one. The participant is then tasked with recalling the words, whilst a rater marks which ones they remembered. Outcome measures is the number of distinct words for the free-recall phase. 

Word list test in the Wechsler Memory Scale III^84^: Used by the LOGOS 

In the word list learning task, the participant is presented a list of 12 words followed by immediate recall of the words. This list is repeated 4 times and after each presentation, the participant recalls as many words as possible. In LOGOS, verbal learning variable is the sum of words correctly recalled in all the trials.

## Paragraph recall tests

All tests with paragraph recall used verbal presentation except CANTAB verbal recall.

Logical Memory adapted from the Original Wechsler Memory Scale (WMS)^86^ used by ADNI1 and ORCADES: Story A (Anna Thompson) from the original WMS version is read to the participant and followed by an immediate free recall. The total score is the number of memory units or "ideas" recalled from story A. Story A contains 25 memory units, hence the maximum score is 25.

Logical Memory from the Wechsler Memory Scale Revised (WMS-R)^61^ used by ARIC, CHS, LBC1921, ORCADES, ROS and MAP: The examiner reads two stories, stopping after each reading for an immediate free recall. Compared to the original version of the WMS the stories have been changed to make them more contemporary in content and language. The total score is the average number of ideas recalled from story A and B. As each story contains 25 scoring units, the maximum score is 25 (25+25/2), with the exception of the ARIC study for which the maximum score is 50 (25 + 25). Of note, in ROS and MAP only story A is used.

Logical Memory from the Wechsler Memory Scale-IIIUK (WMS-IIIUK)^67^ used by FHS1, GS:SFHS, LBC1936, and OATS. This version is very similar to the logical memory test from the WMS-R, but the second story is read twice (except in OATS).

Logical Memory from the Wechsler Memory Scale (3rd edition, WMS-III)^84^ used by Sydney MAS: This version is very similar to the logical memory test from the WMS-R but only the first story was presented.

East Boston Memory Test (EBMT) used by WGHS: The EBMT^113^ consists of a short story read to participants and the score is based on participant's ability to immediately repeat 12 key items in the story, for a range in scores of 0-12.

## Visuo-spatial memory test in the UK Biobank

In this test, participants were given five seconds to memorize the position of as many matching pairs of symbols as possible (maximum of 6 pairs) on the computer screen. The cards were then turned face down on the screen and the participant was asked to touch as many pairs as possible in the fewest tries. We used “Number of incorrect matches in round (#399)” as the memory test score. The test is described in detail here: [https://biobank.ctsu.ox.ac.uk/crystal/refer.cgi?id=100239](https://biobank.ctsu.ox.ac.uk/crystal/refer.cgi?id=100239). The scores ranged from 0 to 12 with higher score indicating more incorrect matches. Mean = 2.3 (SD: 3.1) in the full sample of N=498.787. 

# METHODS IN THE FUNCTIONAL ANALYSES

## Dorsolateral prefrontal cortex methylation QTL analyses 

We downloaded the ROSMAP data from the Accelerating Medicines Partnership – Alzheimer’s Disease (AMP-AD) Knowledge Portal using the synapseClient R package v1.15-0 ([http://docs.synapse.org/articles/getting_started.html](http://docs.synapse.org/articles/getting_started.html))^114^. In the ROSMAP cohort, methylation levels were measured using the Illumina HumanMethylation450 BeadChip in 708 individuals^115^(syn3157275), and 322 of these individuals have genomic data (syn3157329)^99^. We excluded the loci that had too many missing values, or did not pass the quality control criteria (RnBeads Version 1.10.8)^116^. We computed M-values using the lumi R package (Version 2.36.0)^117^. We used the Matrix_eQTL_engine from the MatrixEQTL package (Version 2.2) to determine associations between the SNPs and DNA methylation M-values based on a linear additive model and a t-test. We considered gender and age as covariates in this analysis, and identified mQTLs with an FDR <0.01. We used the minfi (Version 1.30.0) and IlluminaHumanMethylation450kanno.ilmn12.hg19 (Version 0.6.0) packages to determine the genes that correspond to the identified mQTLs.

## In-vivo brain amyloid and Tau burden analyses

Brain Amyloid and Tau PET imaging was carried out on 183 FHS Generation 3 participants who had attended the 2nd quadrennial examination. All participants gave written informed consent. Consent protocols were approved by the Institutional Review Boards of the Boston Medical Center and the Massachusetts General Hospital. Prior to PET imaging, participants completed computer tomography for attenuation correction and structural brain MRI for co-registration. Structural T1-weighted data were acquired using a Philips 3 Tesla Achieva (Philips, Best, Netherlands; 6,800 ms, echo time = 3.1ms, flip angle = 9 degrees, and a voxel size = .98 x .98 x 1.2mm). Images were processed with FreeSurfer (FS) v6.0 ([http://surfer.nmr.mgh.harvard.edu](http://surfer.nmr.mgh.harvard.edu/)) to identify white matter and pial surfaces, and standard cortical regions of interest (ROI) for PET sampling, using manual correction of automated segmentation where required^118,119^.

^11^C-Pittsburgh Compound B (PiB) and ^18^F-Flortaucipir (FTP) were prepared according to previously published protocols (*12*).  All PET data were acquired on a Siemens ECAT HR+ (3D mode; 63 image planes; 15.2-cm axial field of view; 5.6-mm transaxial resolution; and 2.4-mm slice interval). PiB PET images were acquired with a 10 to 15 mCi bolus injection followed by a 60-minute dynamic acquisition and PiB retention expressed as the distribution volume ratio (DVR), compared to a cerebellar cortex reference region^120^. FTP PET was acquired from 80 to 100 minutes (min) in 4 x 5-min frames, and FTP retention expressed as the standardized uptake value ratio (SUVr) with cerebral white matter reference. PET data were evaluated without partial volume effect correction (PVC) in this relatively young sample with minimal atrophy. PET images were co-registered to the corresponding baseline T1 image for each subject (SPM8), and FS-derived ROIs were sampled for all PET data sets. PET data were reconstructed, attenuation corrected, scatter corrected, and evaluated frame by frame for excessive head motion. 

Global amyloid burden was represented using PiB DVR in a large neocortical target region that included superior frontal, rostral middle frontal, rostral anterior cingulate, medial orbitofrontal, inferior and middle temporal, inferior parietal, and precuneus regions (FS-defined frontal, lateral and retrosplenial [FLR] region) compared to a cerebellar cortical reference region^120^.

FTP retention was assessed using vertex-wise surface mapping of the cortical ribbon at the midpoint of the grey matter (surface smoothing kernel: 6mm) (*12*). Based on previous knowledge regarding cortical ROI vulnerability to early tau deposition we examined association in the hippocampal (HC), entorhinal (EC), inferior temporal (ITC) and precuneus (PC) regions^121^.

Association of allelic variation at each locus of interest with the global amyloid and regional tau burden was examined using additive genetic models and linear regressions adjusted for age- at PET imaging, and sex.  

## fMRI study sample and methods

**Participants**

We included data from 435 Caucasian neurotypical controls of European ancestry (mean age ± standard deviation 29 ± 8.8, range 18-54; 257 Female; mean IQ ± standard deviation 109.9 ± 9.1, range 84-132; Handedness [Edinburgh inventory] > 60) recruited in the framework of the Clinical Brain Disorders Branch Sibling Study of schizophrenia at the National Institute of Mental Health (Daniel R. Weinberger, Principal Investigator). The study was approved by the Institutional Review Board of the Intramural Program of the National Institute of Mental Health. Participants signed an informed consent complying with the Declaration of Helsinki after full explanation of all procedures approved by the local ethics committee. All participants provided written informed consent for a protocol approved by the NIMH Institutional Review Board. Participants were assessed in person with a Structured Clinical Interview for DSM-IV^122^. Exclusion criteria for healthy participants included: presence of a psychiatric disorder at the time of the study and by history; having a first-degree relative with a psychiatric disorder; IQ>80; recent drug or alcohol abuse (within 1 year) or 45 years of previous abuse; substantial medical or neurological conditions; and current psychotropic pharmacological treatment. Inclusion/exclusion criteria have been described in detail elsewhere^123^.

**Working memory task** 

The N-Back task consisted of one run with two conditions (0-back and 2-back). Participants did not exit the scanner during the breaks. The stimuli were arranged in a block design, consisting of eight 30-s blocks: four blocks of the control condition alternating with four blocks of 2-back. Each block began with task instructions (2 s) and included 14 task trials (duration: 0.5 s, inter-trial interval: 1.5 s). Stimuli were presented via a back-projection system and behavioral responses were recorded through an optic fiber response box which allowed assessment of accuracy (percentage of correct responses) and reaction time for each trial. Numbers appeared at a fixed location, which was highlighted by a circle within the diamond-shaped box. Participants responded with their right hand with a four-button MRI-compatible pad within the scanner. Participants scoring below 70% in the 2-back task were excluded by protocol. 

fMRI data acquisition and analysis

BOLD fMRI data were acquired on a 3T GE Signa Scanner (Milwaukee, WI) using a gradient-echo echo planar imaging sequence (TR=2000 ms, TE=30 ms, flip angle=90°, field of view=24 cm, matrix=64x64, 24 slices 6 mm thick). Individual linear contrast images of the 2-back > 0-back conditions were entered in a second-level group analysis. The fMRI images were processed following standard procedures in SPM12 (http://www.fil.ion.ucl.ac.uk/spm). The fMRI images were first co-registered to high-resolution anatomical images. Data were corrected for head motion artifacts, excluded if motion exceeded 2 mm in translation or 1.5° in rotation (with motion parameters used as covariates of no interest in first level analysis), spatially normalized to a 3 × 3 × 3 mm^3^ voxel size into a standard stereotactic space (MNI template) using affine and nonlinear transformation, and then smoothed with an 8 mm full-width at half-maximum Gaussian filter. The processed images were analyzed in a two-level procedure. At the first level, separate general linear models were specified for each subject by modeling the alternating task conditions as a box car reference vector that was convolved with the standard hemodynamic response function at each voxel. Movement parameters were added as nuisance variables. Data quality was further assessed based on time series signal-to-noise ratio, signal variance, and artifacts like ghosting at each stage of initial analysis. Second-level results were masked for activity at 2-back. 

Participants took part in a separate session aimed to collect socio-demographic and neuropsychological information by means of semi-structured interviews. We used the Edinburgh Handedness Inventory to assess hand preference and only included participants scoring at least 60 in favor of right-hand use. The final sample included participants aged 18 to 54 years (mean ± standard deviation: 29 ± 8.8; 257 Female), with IQ assessed via Wechsler Adult Intelligence Scale within the normal range (84-132; mean ± standard deviation: 109 ± 9).

**Genotyping** 

Participants in the imaging study underwent blood withdrawal for subsequent DNA extraction from peripheral blood mononuclear cells. Standard methods to extract DNA with the Puregene purification kit (Gentra Systems; Minneapolis, MN, USA) were used. Genotypes were obtained using several different Illumina BeadChips (550K/610K/660K/2.5M), which were designed, manufactured and completed by Illumina (San Diego, CA, USA). Briefly, each sample was whole-genome amplified, fragmented, precipitated and resuspended in appropriate concentrations of hybridization buffer. After hybridization, the Bead Chip oligonucleotides were extended by a single labeled base, which was detected by fluorescence imaging with an Illumina Bead Array Reader. Normalized bead intensity data obtained for each sample were loaded into the Illumina GenomeStudio with cluster position files provided by Illumina, and fluorescence intensities were converted into SNP genotypes. 

Genotyped SNPs used for imputation were required to have missing rate < .02, Hardy-Weinberg equilibrium P > 10^-6^, and minor allele frequency (MAF) > .01. Sample duplications and cryptic relatedness were ruled out through identity-by-state (IBS) analysis of genotype data. Pre-phasing was done before imputation with SHAPEIT^124^, and imputation was done with IMPUTE2^125^ using 1000 genome phase 3 as reference panel^124,126^. After imputation, imputed dosage data for each SNP with imputation quality (INFO)>0.1 were used for calculation of polygenic scores. After genotypes were called and the pedigree file was assembled, we removed SNPs showing minor allele frequency <5%, genotype missing rate >2%, or deviation from Hardy-Weinberg equilibrium (p<.001). Individuals were also removed if their overall genotyping rate was below 98%. To control for population stratification in the association analysis, the first 10 principal components of the whole genome data were calculated using EIGENSOFT v5.01 (EIGENSOFT,

http://www.hsph.harvard.edu/alkes-price/software/).

**Polygenic score computation**

Polygenic scores for verbal short-term memory (PGS_VSTM_) and verbal learning (PGS_VL_) were computed for each individual as a measure of predicted performance based on the GWAS weights. We obtained betas associating allele dose with performance for 115,414 and 57,689, respectively, linkage disequilibrium-independent (R^2 ^< .1) index SNPs spanning across the whole genome. We then computed a weighted sum of the cumulative SNP effects by summing the imputation probability for the reference allele of the index SNP, weighted by the effect size of association with performance, at each independent locus across the whole genome, as described elsewhere^127^. 

We used three different p-value thresholds of association with short-term and verbal memory for SNP inclusion in the score: 5 × 10^-8^ (GWAS significance), 10^-4^ (intermediate significance), 0.05 (nominal significance). SNPs in sets with lower P-values were also in sets with higher P-values.

Supplementary Table 1. Number of independent single-nucleotide polymorphisms included in the Polygenic scores for verbal memory (PGS_VL_) and short-term memory (PGS_VSTM_).

| **P threshold** | **PGS**_**VSTM**_ | **PGS**_**VL**_ |
| --- | --- | --- |
| **P<5e-08** | 2 | 1 |
| **P<1e-04** | 104 | 61 |
| **P<0.05** | 19,115 | 7,488 |

**Association of the verbal learning polygenic score with brain activity and behavior during working memory performance** 

Individual contrast images were used for regression analysis at the group level. Before the analyses, we screened the association of PGSs with demographic (age, sex, education, IQ) and behavioral (2-back accuracy and reaction times) variables using linear models. Females had lower intermediate PGS_VL_ (SNP inclusion p = 10^-4^) than males (p = .046); greater intermediate PGS_VSTM_ was associated with shorter 2-back reaction times (p = .035); we also found a marginally significant positive association of the whole-genome significant PGS_STM_ with education (p = .091). Hence, we included sex and education as nuisance variables in the fMRI models. We also included Age because of the large range of variation in our sample.

# REFERENCES

1.	Kasem, E., Kurihara, T. & Tabuchi, K. Neurexins and neuropsychiatric disorders. *Neurosci Res* **127**, 53-60 (2018).

2.	Hu, Z., Xiao, X., Zhang, Z. & Li, M. Genetic insights and neurobiological implications from NRXN1 in neuropsychiatric disorders. *Mol Psychiatry* **24**, 1400-1414 (2019).

3.	Need, A.C. *et al.* A genome-wide study of common SNPs and CNVs in cognitive performance in the CANTAB. *Hum Mol Genet* **18**, 4650-61 (2009).

4.	Voineskos, A.N. *et al.* Neurexin-1 and frontal lobe white matter: an overlapping intermediate phenotype for schizophrenia and autism spectrum disorders. *PLoS One* **6**, e20982 (2011).

5.	UKBiobank PheWeb ([http://pheweb.sph.umich.edu](http://pheweb.sph.umich.edu) ) / accessed 18.9.2019. (2019).

6.	Davies, G. *et al.* Study of 300,486 individuals identifies 148 independent genetic loci influencing general cognitive function. *Nat Commun* **9**, 2098 (2018).

7.	Biological insights from 108 schizophrenia-associated genetic loci. *Nature* **511**, 421-7 (2014).

8.	Li, Z. *et al.* Loci with genome-wide associations with schizophrenia in the Han Chinese population. *Br J Psychiatry* **207**, 490-4 (2015).

9.	Witt, S.H. *et al.* Investigation of manic and euthymic episodes identifies state- and trait-specific gene expression and STAB1 as a new candidate gene for bipolar disorder. *Transl Psychiatry* **4**, e426 (2014).

10.	McMahon, F.J. *et al.* Meta-analysis of genome-wide association data identifies a risk locus for major mood disorders on 3p21.1. *Nat Genet* **42**, 128-31 (2010).

11.	Ripke, S. *et al.* A mega-analysis of genome-wide association studies for major depressive disorder. *Mol Psychiatry* **18**, 497-511 (2013).

12.	Identification of risk loci with shared effects on five major psychiatric disorders: a genome-wide analysis. *Lancet* **381**, 1371-1379 (2013).

13.	Smeland, O.B. *et al.* Genetic Overlap Between Schizophrenia and Volumes of Hippocampus, Putamen, and Intracranial Volume Indicates Shared Molecular Genetic Mechanisms. *Schizophr Bull* **44**, 854-864 (2018).

14.	Smit, D.J.A. *et al.* Genome-wide association analysis links multiple psychiatric liability genes to oscillatory brain activity. *Hum Brain Mapp* **39**, 4183-4195 (2018).

15.	Yang, M.H. *et al.* Activity-dependent neuroprotector homeobox protein: A candidate protein identified in serum as diagnostic biomarker for Alzheimer's disease. *J Proteomics* **75**, 3617-29 (2012).

16.	Yang, C.P. *et al.* Comprehensive integrative analyses identify GLT8D1 and CSNK2B as schizophrenia risk genes. *Nat Commun* **9**, 838 (2018).

17.	Takata, A., Matsumoto, N. & Kato, T. Genome-wide identification of splicing QTLs in the human brain and their enrichment among schizophrenia-associated loci. *Nat Commun* **8**, 14519 (2017).

18.	van Hulzen, K.J.E. *et al.* Genetic Overlap Between Attention-Deficit/Hyperactivity Disorder and Bipolar Disorder: Evidence From Genome-wide Association Study Meta-analysis. *Biol Psychiatry* **82**, 634-641 (2017).

19.	Ferrer, I. Cognitive impairment of vascular origin: neuropathology of cognitive impairment of vascular origin. *J Neurol Sci* **299**, 139-49 (2010).

20.	Seppala, I. *et al.* Genome-wide association study on dimethylarginines reveals novel AGXT2 variants associated with heart rate variability but not with overall mortality. *Eur Heart J* **35**, 524-31 (2014).

21.	Alexander, J. *et al.* Familial early-onset dementia with complex neuropathologic phenotype and genomic background. *Neurobiol Aging* **42**, 199-204 (2016).

22.	Redies, C., Hertel, N. & Hubner, C.A. Cadherins and neuropsychiatric disorders. *Brain Res* **1470**, 130-44 (2012).

23.	Saykin, A.J. *et al.* Alzheimer's Disease Neuroimaging Initiative biomarkers as quantitative phenotypes: Genetics core aims, progress, and plans. *Alzheimers Dement* **6**, 265-73 (2010).

24.	Terracciano, A. *et al.* Genome-wide association scan of trait depression. *Biol Psychiatry* **68**, 811-7 (2010).

25.	Singh, S.M., Castellani, C. & O'Reilly, R. Autism meets schizophrenia via cadherin pathway. *Schizophr Res* **116**, 293-4 (2010).

26.	Motazacker, M.M. *et al.* A defect in the ionotropic glutamate receptor 6 gene (GRIK2) is associated with autosomal recessive mental retardation. *Am J Hum Genet* **81**, 792-8 (2007).

27.	Bartlett, C.W. *et al.* A major susceptibility locus for specific language impairment is located on 13q21. *Am J Hum Genet* **71**, 45-55 (2002).

28.	Bradford, Y. *et al.* Incorporating language phenotypes strengthens evidence of linkage to autism. *Am J Med Genet* **105**, 539-47 (2001).

29.	Truong, D.T. *et al.* Multipoint genome-wide linkage scan for nonword repetition in a multigenerational family further supports chromosome 13q as a locus for verbal trait disorders. *Hum Genet* **135**, 1329-1341 (2016).

30.	Lee, J.J. *et al.* Gene discovery and polygenic prediction from a genome-wide association study of educational attainment in 1.1 million individuals. *Nat Genet* **50**, 1112-1121 (2018).

31.	Arpawong, T.E. *et al.* Genetic variants specific to aging-related verbal memory: Insights from GWASs in a population-based cohort. *PLoS One* **12**, e0182448 (2017).

32.	Bekris, L.M., Lutz, F. & Yu, C.E. Functional analysis of APOE locus genetic variation implicates regional enhancers in the regulation of both TOMM40 and APOE. *J Hum Genet* **57**, 18-25 (2012).

33.	Davies, G. *et al.* Genetic contributions to variation in general cognitive function: a meta-analysis of genome-wide association studies in the CHARGE consortium (N=53949). *Mol Psychiatry* **20**, 183-92 (2015).

34.	Rantalainen, V. *et al.* APOE and aging-related cognitive change in a longitudinal cohort of men. *Neurobiol Aging* **44**, 151-158 (2016).

35.	Zhang, C. & Pierce, B.L. Genetic susceptibility to accelerated cognitive decline in the US Health and Retirement Study. *Neurobiol Aging* **35**, 1512.e11-8 (2014).

36.	Deary, I.J. *et al.* Cognitive change and the APOE epsilon 4 allele. *Nature* **418**, 932 (2002).

37.	Kunkle, B.W. *et al.* Genetic meta-analysis of diagnosed Alzheimer's disease identifies new risk loci and implicates Abeta, tau, immunity and lipid processing. *Nat Genet* **51**, 414-430 (2019).

38.	Rantalainen, V. *et al.* APOE varepsilon4, rs405509, and rs440446 promoter and intron-1 polymorphisms and dementia risk in a cohort of elderly Finns-Helsinki Birth Cohort Study. *Neurobiol Aging* **73**, 230.e5-230.e8 (2019).

39.	Saykin, A.J. *et al.* Genetic studies of quantitative MCI and AD phenotypes in ADNI: Progress, opportunities, and plans. *Alzheimers Dement* **11**, 792-814 (2015).

40.	Nho, K. *et al.* The effect of reference panels and software tools on genotype imputation. *AMIA Annu Symp Proc* **2011**, 1013-8 (2011).

41.	Nho, K. *et al.* Whole-exome sequencing and imaging genetics identify functional variants for rate of change in hippocampal volume in mild cognitive impairment. *Mol Psychiatry* **18**, 781-7 (2013).

42.	Nho, K. *et al.* Protective variant for hippocampal atrophy identified by whole exome sequencing. *Ann Neurol* **77**, 547-52 (2015).

43.	Ramirez, A. *et al.* Elevated HbA1c is associated with increased risk of incident dementia in primary care patients. *J Alzheimers Dis* **44**, 1203-12 (2015).

44.	Luck, T. *et al.* A hierarchy of predictors for dementia-free survival in old-age: results of the AgeCoDe study. *Acta Psychiatr Scand* **129**, 63-72 (2014).

45.	Papassotiropoulos, A. *et al.* A genome-wide survey of human short-term memory. *Mol Psychiatry* **16**, 184-92 (2011).

46.	Harris, T.B. *et al.* Age, Gene/Environment Susceptibility-Reykjavik Study: multidisciplinary applied phenomics. *Am J Epidemiol* **165**, 1076-87 (2007).

47.	Palm, W.M. *et al.* Ventricular dilation: association with gait and cognition. *Ann Neurol* **66**, 485-93 (2009).

48.	Delis, D., Kramer, J. & Kaplan, E. *The California Verbal Learning Test—Research Edition*, (Psychological Corporation, New York, NY, 1987).

49.	The Atherosclerosis Risk in Communities (ARIC) Study: design and objectives. The ARIC investigators. *Am J Epidemiol* **129**, 687-702 (1989).

50.	Rosamond, W.D. *et al.* Stroke incidence and survival among middle-aged adults: 9-year follow-up of the Atherosclerosis Risk in Communities (ARIC) cohort. *Stroke* **30**, 736-43 (1999).

51.	Wechsler, D. *Wechsler memory scale-revised (manual)*, (Psychological Corporation, New York, NY, 1987).

52.	Knopman, D.S., Mosley, T.H., Catellier, D.J., Coker, L.H. & Atherosclerosis Risk in Communities Study Brain, M.R.I.S. Fourteen-year longitudinal study of vascular risk factors, APOE genotype, and cognition: the ARIC MRI Study. *Alzheimers Dement* **5**, 207-14 (2009).

53.	Knopman, D.S. *et al.* Mild Cognitive Impairment and Dementia Prevalence: The Atherosclerosis Risk in Communities Neurocognitive Study (ARIC-NCS). *Alzheimers Dement (Amst)* **2**, 1-11 (2016).

54.	Folstein, M.F., Folstein, S.E. & McHugh, P.R. "Mini-mental state". A practical method for grading the cognitive state of patients for the clinician. *J Psychiatr Res* **12**, 189-98 (1975).

55.	Friedman, G.D. *et al.* CARDIA: study design, recruitment, and some characteristics of the examined subjects. *J Clin Epidemiol* **41**, 1105-16 (1988).

56.	Rosenberg, S.J., Ryan, J.J. & Prifitera, A. Rey Auditory-Verbal Learning Test performance of patients with and without memory impairment. *J Clin Psychol* **40**, 785-7 (1984).

57.	Fried, L.P. *et al.* The Cardiovascular Health Study: design and rationale. *Ann Epidemiol* **1**, 263-76 (1991).

58.	Longstreth, W.T., Jr. *et al.* Frequency and predictors of stroke death in 5,888 participants in the Cardiovascular Health Study. *Neurology* **56**, 368-75 (2001).

59.	Lopez, O.L. *et al.* Evaluation of dementia in the cardiovascular health cognition study. *Neuroepidemiology* **22**, 1-12 (2003).

60.	Lopez, O.L. *et al.* Neuropsychological characteristics of mild cognitive impairment subgroups. *J Neurol Neurosurg Psychiatry* **77**, 159-65 (2006).

61.	Wechsler, D. *Wechsler memory-scale-revised (manual)*, (Psychological Corporation, New York, 1987).

62.	Service, S. *et al.* Magnitude and distribution of linkage disequilibrium in population isolates and implications for genome-wide association studies. *Nat Genet* **38**, 556-60 (2006).

63.	Aulchenko, Y.S. *et al.* Linkage disequilibrium in young genetically isolated Dutch population. *Eur J Hum Genet* **12**, 527-34 (2004).

64.	van Koolwijk, L.M. *et al.* Association of cognitive functioning with retinal nerve fiber layer thickness. *Invest Ophthalmol Vis Sci* **50**, 4576-80 (2009).

65.	Rey, A. *L’Examen Clinique en Psychologie*, (Presses Universitaires de France, Paris, 1964).

66.	Saan, R. & Deelman, B. *De 15-woordentest A en B: Een Voorlopige Handleiding [Internal Report]*, (University Hospital Groningen, Department of Neuropsychology, Groningen, The Netherlands, 1986).

67.	Wechsler, D. *WMS-IIIUK administration and scoring manual*, (Psychological Corporation, London, UK, 1998).

68.	Kaprio, J., Pulkkinen, L. & Rose, R.J. Genetic and environmental factors in health-related behaviors: studies on Finnish twins and twin families. *Twin Res* **5**, 366-71 (2002).

69.	Latvala, A. *et al.* Genetic origins of the association between verbal ability and alcohol dependence symptoms in young adulthood. *Psychol Med* **41**, 641-51 (2011).

70.	Smith, B.H. *et al.* Cohort Profile: Generation Scotland: Scottish Family Health Study (GS:SFHS). The study, its participants and their potential for genetic research on health and illness. *Int J Epidemiol* **42**, 689-700 (2013).

71.	Smith, B.H. *et al.* Generation Scotland: the Scottish Family Health Study; a new resource for researching genes and heritability. *BMC Med Genet* **7**, 74 (2006).

72.	Yang, J., Lee, S.H., Goddard, M.E. & Visscher, P.M. GCTA: a tool for genome-wide complex trait analysis. *Am J Hum Genet* **88**, 76-82 (2011).

73.	Kerr, S.M. *et al.* Pedigree and genotyping quality analyses of over 10,000 DNA samples from the Generation Scotland: Scottish Family Health Study. *BMC Med Genet* **14**, 38 (2013).

74.	McEvoy, M. *et al.* Cohort profile: The Hunter Community Study. *Int J Epidemiol* **39**, 1452-63 (2010).

75.	Cesari, M. *et al.* Inflammatory markers and cardiovascular disease (The Health, Aging and Body Composition [Health ABC] Study). *Am J Cardiol* **92**, 522-8 (2003).

76.	Lahti, J. *et al.* Glucocorticoid receptor gene haplotype predicts increased risk of hospital admission for depressive disorders in the Helsinki birth cohort study. *Journal of Psychiatric Research* **45**, 1160-1164 (2011).

77.	Barker, D.J., Osmond, C., Forsen, T.J., Kajantie, E. & Eriksson, J.G. Trajectories of growth among children who have coronary events as adults. *N Engl J Med* **353**, 1802-9 (2005).

78.	Morris, J.C. *et al.* The Consortium to Establish a Registry for Alzheimer's Disease (CERAD). Part I. Clinical and neuropsychological assessment of Alzheimer's disease. *Neurology* **39**, 1159-65 (1989).

79.	Deary, I.J., Gow, A.J., Pattie, A. & Starr, J.M. Cohort profile: the Lothian Birth Cohorts of 1921 and 1936. *Int J Epidemiol* **41**, 1576-84 (2012).

80.	Deary, I.J. *et al.* The Lothian Birth Cohort 1936: a study to examine influences on cognitive ageing from age 11 to age 70 and beyond. *BMC Geriatr* **7**, 28 (2007).

81.	Deary, I.J., Whiteman, M.C., Starr, J.M., Whalley, L.J. & Fox, H.C. The impact of childhood intelligence on later life: following up the Scottish mental surveys of 1932 and 1947. *J Pers Soc Psychol* **86**, 130-47 (2004).

82.	Loeffler, M. *et al.* The LIFE-Adult-Study: objectives and design of a population-based cohort study with 10,000 deeply phenotyped adults in Germany. *BMC Public Health* **15**, 691 (2015).

83.	Sachdev, P.S. *et al.* A comprehensive neuropsychiatric study of elderly twins: the Older Australian Twins Study. *Twin Res Hum Genet* **12**, 573-82 (2009).

84.	Wechsler, D. *Wechsler Memory Scale - Third edition*, (The Psychological Corporation, San Antonio TX, 1997).

85.	McQuillan, R. *et al.* Runs of homozygosity in European populations. *Am J Hum Genet* **83**, 359-72 (2008).

86.	Wechsler, D. *Wechsler memory scale*, (Psychological Corporation, San Antonio, TX, US, 1945).

87.	Shepherd, J. *et al.* Pravastatin in elderly individuals at risk of vascular disease (PROSPER): a randomised controlled trial. *Lancet* **360**, 1623-30 (2002).

88.	Shepherd, J. *et al.* The design of a prospective study of Pravastatin in the Elderly at Risk (PROSPER). PROSPER Study Group. PROspective Study of Pravastatin in the Elderly at Risk. *Am J Cardiol* **84**, 1192-7 (1999).

89.	Trompet, S. *et al.* Replication of LDL GWAs hits in PROSPER/PHASE as validation for future (pharmaco)genetic analyses. *BMC Med Genet* **12**, 131 (2011).

90.	Ikram, M.A. *et al.* The Rotterdam Study: 2018 update on objectives, design and main results. *Eur J Epidemiol* **32**, 807-850 (2017).

91.	Hoogendam, Y.Y., Hofman, A., van der Geest, J.N., van der Lugt, A. & Ikram, M.A. Patterns of cognitive function in aging: the Rotterdam Study. *Eur J Epidemiol* **29**, 133-40 (2014).

92.	Bleecker, M.L., Bolla-Wilson, K., Agnew, J. & Meyers, D.A. Age-related sex differences in verbal memory. *J Clin Psychol* **44**, 403-11 (1988).

93.	de Bruijn, R.F.A.G. *et al.* The potential for prevention of dementia across two decades: the prospective, population-based Rotterdam Study. *BMC Medicine* **13**, 132 (2015).

94.	Wieberdink, R.G., Ikram, M.A., Hofman, A., Koudstaal, P.J. & Breteler, M.M. Trends in stroke incidence rates and stroke risk factors in Rotterdam, the Netherlands from 1990 to 2008. *Eur J Epidemiol* **27**, 287-95 (2012).

95.	Bennett, D.A., Schneider, J.A., Arvanitakis, Z. & Wilson, R.S. Overview and findings from the religious orders study. *Curr Alzheimer Res* **9**, 628-45 (2012).

96.	Bennett, D.A. *et al.* Overview and findings from the rush Memory and Aging Project. *Curr Alzheimer Res* **9**, 646-63 (2012).

97.	Bennett, D.A. *et al.* Religious Orders Study and Rush Memory and Aging Project. *J Alzheimers Dis* **64**, S161-S189 (2018).

98.	Bennett, D.A. *et al.* Neuropathology of older persons without cognitive impairment from two community-based studies. *Neurology* **66**, 1837-44 (2006).

99.	Tasaki, S., Gaiteri, C., Mostafavi, S., De Jager, P.L. & Bennett, D.A. The Molecular and Neuropathological Consequences of Genetic Risk for Alzheimer's Dementia. *Front Neurosci* **12**, 699 (2018).

100.	Volzke, H. *et al.* Cohort profile: the study of health in Pomerania. *Int J Epidemiol* **40**, 294-307 (2011).

101.	Wittchen, H.U., Lachner, G., Wunderlich, U. & Pfister, H. Test-retest reliability of the computerized DSM-IV version of the Munich-Composite International Diagnostic Interview (M-CIDI). *Soc Psychiatry Psychiatr Epidemiol* **33**, 568-78 (1998).

102.	Appel, K. *et al.* Moderation of adult depression by a polymorphism in the FKBP5 gene and childhood physical abuse in the general population. *Neuropsychopharmacology* **36**, 1982-91 (2011).

103.	Oswald, W.D. & Fleischmann, U.M. *Nürnberger-Alters-Inventar NAI. Testkasten und Kurzmanual*, (Universität Erlangen-Nürnberg, Nürnberg, 1986).

104.	Sachdev, P.S. *et al.* The Sydney Memory and Ageing Study (MAS): methodology and baseline medical and neuropsychiatric characteristics of an elderly epidemiological non-demented cohort of Australians aged 70-90 years. *Int Psychogeriatr* **22**, 1248-64 (2010).

105.	Ridker, P.M. *et al.* Rationale, design, and methodology of the Women's Genome Health Study: a genome-wide association study of more than 25,000 initially healthy american women. *Clin Chem* **54**, 249-55 (2008).

106.	Kang, J.H., Cook, N., Manson, J., Buring, J.E. & Grodstein, F. Low dose aspirin and cognitive function in the women's health study cognitive cohort. *Bmj* **334**, 987 (2007).

107.	Lezak, M. *Neuropsychological testing*, (Oxford University Press, Oxford, UK, 2004).

108.	Schofield, P.W. *et al.* The Audio Recorded Cognitive Screen (ARCS): a flexible hybrid cognitive test instrument. *J Neurol Neurosurg Psychiatry* **81**, 602-7 (2010).

109.	Grober, E., Ocepek-Welikson, K. & Teresi, J.A. The Free and Cued Selective Reminding Test: evidence of psychometric adequacy. **51**, 266-282 (2009).

110.	Brandt, J., Spencer, M. & Folstein, M. The Telephone Interview for Cognitive Status. *Cognitive and Behavioral Neurology* **1**, 111-118 (1988).

111.	Brand, N. & Jolles, J. Learning and retrieval rate of words presented auditorily and visually. *J Gen Psychol* **112**, 201-10 (1985).

112.	CANTAB® [Cognitive assessment software]. All rights reserved. [www.cantab.com](www.cantab.com). (Cambridge Cognition, 2019 ).

113.	Albert, M. *et al.* Use of brief cognitive tests to identify individuals in the community with clinically diagnosed Alzheimer's disease. *Int J Neurosci* **57**, 167-78 (1991).

114.	Hodes, R.J. & Buckholtz, N. Accelerating Medicines Partnership: Alzheimer's Disease (AMP-AD) Knowledge Portal Aids Alzheimer's Drug Discovery through Open Data Sharing. *Expert Opin Ther Targets* **20**, 389-91 (2016).

115.	De Jager, P.L. *et al.* Alzheimer's disease: early alterations in brain DNA methylation at ANK1, BIN1, RHBDF2 and other loci. *Nat Neurosci* **17**, 1156-63 (2014).

116.	Assenov, Y. *et al.* Comprehensive analysis of DNA methylation data with RnBeads. *Nat Methods* **11**, 1138-1140 (2014).

117.	Du, P., Kibbe, W.A. & Lin, S.M. lumi: a pipeline for processing Illumina microarray. *Bioinformatics* **24**, 1547-8 (2008).

118.	Desikan, R.S. *et al.* An automated labeling system for subdividing the human cerebral cortex on MRI scans into gyral based regions of interest. *Neuroimage* **31**, 968-80 (2006).

119.	Greve, D.N. *et al.* Cortical surface-based analysis reduces bias and variance in kinetic modeling of brain PET data. *Neuroimage* **92**, 225-36 (2014).

120.	Hanseeuw, B.J. *et al.* PET staging of amyloidosis using striatum. *Alzheimers Dement* **14**, 1281-1292 (2018).

121.	Johnson, K.A. *et al.* Tau positron emission tomographic imaging in aging and early Alzheimer disease. *Ann Neurol* **79**, 110-9 (2016).

122.	Association, A.P. *Structured Clinical Interview for DSM Disorders*, (American Psychiatric Association, Washington DC, 1994).

123.	Pergola, G. *et al.* DRD2 co-expression network and a related polygenic index predict imaging, behavioral and clinical phenotypes linked to schizophrenia. *Transl Psychiatry* **7**, e1006 (2017).

124.	Delaneau, O., Marchini, J. & Zagury, J.F. A linear complexity phasing method for thousands of genomes. *Nat Methods* **9**, 179-81 (2011).

125.	Howie, B.N., Donnelly, P. & Marchini, J. A flexible and accurate genotype imputation method for the next generation of genome-wide association studies. *PLoS Genet* **5**, e1000529 (2009).

126.	Howie, B., Marchini, J. & Stephens, M. Genotype imputation with thousands of genomes. *G3 (Bethesda)* **1**, 457-70 (2011).

127.	Chen, Q. *et al.* Schizophrenia polygenic risk score predicts mnemonic hippocampal activity. *Brain* **141**, 1218-1228 (2018).
